# A non-photosynthetic green alga illuminates the reductive evolution of plastid electron transport systems

**DOI:** 10.1186/s12915-020-00853-w

**Published:** 2020-09-16

**Authors:** Motoki Kayama, Jun-Feng Chen, Takashi Nakada, Yoshiki Nishimura, Toshiharu Shikanai, Tomonori Azuma, Hideaki Miyashita, Shinichi Takaichi, Yuichiro Kashiyama, Ryoma Kamikawa

**Affiliations:** 1 Graduate School of Human and Environmental Studies, Kyoto University, Yoshida nihonmatsu cho, Sakyo ku, Kyoto, Kyoto 606-8501 Japan; 2 Institute for Advanced Biosciences, Keio University, Tsuruoka, Japan; 3 Graduate School of Science, Kyoto University, Kyoto, Japan; 4 Department of Molecular Microbiology, Tokyo University of Agriculture, Tokyo, Japan; 5 Graduate School of Engineering, Fukui University of Technology, Fukui, Japan; 6 Graduate School of Agriculture, Kyoto University, Kitashirakawa oiwake cho, Sakyo ku, Kyoto, Kyoto 606-8502 Japan

**Keywords:** Carotenoid, NADPH, Non-photosynthetic plastid, Plastoquinol, Plastid terminal oxidase, Transient RNA interference knockdown

## Abstract

**Background:**

Plastid electron transport systems are essential not only for photosynthesis but also for dissipating excess reducing power and sinking excess electrons generated by various redox reactions. Although numerous organisms with plastids have lost their photoautotrophic lifestyles, there is a spectrum of known functions of remnant plastids in non-photosynthetic algal/plant lineages; some of non-photosynthetic plastids still retain diverse metabolic pathways involving redox reactions while others, such as apicoplasts of apicomplexan parasites, possess highly reduced sets of functions. However, little is known about underlying mechanisms for redox homeostasis in functionally versatile non-photosynthetic plastids and thus about the reductive evolution of plastid electron transport systems.

**Results:**

Here we demonstrated that the central component for plastid electron transport systems, plastoquinone/plastoquinol pool, is still retained in a novel strain of an obligate heterotrophic green alga lacking the photosynthesis-related thylakoid membrane complexes. Microscopic and genome analyses revealed that the Volvocales green alga, chlamydomonad sp. strain NrCl902, has non-photosynthetic plastids and a plastid DNA that carries no genes for the photosynthetic electron transport system. Transcriptome-based in silico prediction of the metabolic map followed by liquid chromatography analyses demonstrated carotenoid and plastoquinol synthesis, but no trace of chlorophyll pigments in the non-photosynthetic green alga. Transient RNA interference knockdown leads to suppression of plastoquinone/plastoquinol synthesis. The alga appears to possess genes for an electron sink system mediated by plastid terminal oxidase, plastoquinone/plastoquinol, and type II NADH dehydrogenase. Other non-photosynthetic algae/land plants also possess key genes for this system, suggesting a broad distribution of an electron sink system in non-photosynthetic plastids.

**Conclusion:**

The plastoquinone/plastoquinol pool and thus the involved electron transport systems reported herein might be retained for redox homeostasis and might represent an intermediate step towards a more reduced set of the electron transport system in many non-photosynthetic plastids. Our findings illuminate a broadly distributed but previously hidden step of reductive evolution of plastid electron transport systems after the loss of photosynthesis.

## Background

Multiple lineages possess plastids acquired through independent endosymbioses in eukaryotes [1, 2]. An engulfed cyanobacterium by a heterotrophic eukaryote evolved to form the first plastid, an evolutionary event that occurred in the last common ancestor of Archaeplastida, which comprises land plants, green algae, red algae, and glaucophytes. Other algal lineages possess red alga-derived or green alga-derived complex plastids acquired by secondary or tertiary endosymbiotic events. The fact that plastids have been laterally transferred multiple times through eukaryote-eukaryote endosymbioses has spawned much interest in untangling the evolutionary events around the diversity of extant plastids.

Photosynthesis provides algae and plants with biochemical energy in forms of ATP and NADPH by conversion of solar energy through the plastid ATP synthase complex following photosynthetic electron transport accomplished by the cytochrome *b*_6_/*f* complex, plastoquinone/plastoquinol (PQ/PQH_2_), plastocyanin, ferredoxin (Fd), Fd:NADP^+^ reductase (FNR), and photosynthetic pigment systems, i.e., photosystems (PS) I and II [3–6]. The generated NADPH and ATP are utilized for carbon fixation through the Calvin Benson cycle as well as for various other metabolic pathways that are essential in the photosynthetic plastid. Regardless of the beneficial aspects of photosynthesis that allows autotrophic lifestyles, some species of algal and plant lineages have lost their photoautotrophic lifestyles secondarily. Almost all photosynthetic lineages appear to include such secondary heterotrophs, indicating multiple losses of photoautotrophic lifestyles independently in eukaryotic evolution [7].

The most well-studied species among such secondary heterotrophs is the malaria parasite *Plasmodium falciparum* (Apicomplexa), which bears a non-photosynthetic plastid called an apicoplast, with only a few metabolic functions such as biosynthesis of heme, Fe-S cluster, fatty acids, and isopentenyl pyrophosphates [8]. It also retains the most reduced system of photosynthetic electron transport comprising only Fd and FNR [9, 10]. However, recent investigations have greatly expanded the spectrum of known functions of remnant plastids in non-photosynthetic algal/plant lineages [11–19] and have been reviewed in recent papers [20, 21]. For example, the non-photosynthetic diatom plastid in *Nitzschia* sp. NIES-3581 still retains multiple redox reactions for glycolysis, pentose phosphate pathway, and biosynthesis of a variety of amino acids, in addition to the functions found in the apicoplast, except for isopentenyl pyrophosphate synthesis [16]. The volvocales green algae *Polytomella* spp. and the trebouxiophycean green alga *Helicosporidium* sp. also possess similarly complex metabolisms in non-photosynthetic plastids [11, 12]. In contrast, biosynthetic pathways for certain amino acids and fatty acids are reported to be lost in non-photosynthetic plastids of the chrysophycean “*Spumella*” sp. NIES-1846 bearing only glycolysis and biosynthesis of heme and Fe-S cluster [17]. The newly discovered sister lineage of red algae, Rhodelphidia, possesses a non-photosynthetic plastid metabolically functioning only for the synthesis of heme and Fe-S clusters [18]. Nuclear-encoded proteins imported across plastid membranes after translation in the cytosol are responsible for all the functions introduced above [8–19].

In contrast to the metabolic functions, few studies have focused on the evolution of the plastid electron transport system in non-photosynthetic plastids. In addition to photosynthetic linear electron transport, multiple branched pathways of electron transport mediated by PQ/PQH_2_ and plastid terminal oxidase (PTOX) are equipped in photosynthetic plastids as particular components to avoid the fatal photodamage caused by inefficient linear electron transport due to an imbalanced ratio of reducing power and ATP [22–25]. In particular, chlororespiration is a system in which type II NADH dehydrogenase (NDH2) re-oxidizes the reducing power NADPH from which electrons are discarded through PQ/PQH_2_ and PTOX in photosynthetic green algae [22–25]. Chlororespiration is also likely to contribute to redox homeostasis as a safety valve when excess NADPH is generated by plastid biochemical reactions [26]. The system mediated by PQ/PQH_2_ and PTOX also contributes as a sink for electrons generated through the carotenoid biosynthetic pathway [22–25].

It is noteworthy that some non-photosynthetic plastids still retain numerous biochemical functions involved in redox reactions using NADP^+^/NADPH and involved in the carotenoid biosynthetic pathway (e.g., [13, 16]). In turn, this suggests that the electron transport systems for redox homeostasis that dissipate excess reducing power and sink excess electrons might also be dedicated to certain functions of non-photosynthetic plastids. However, underlying mechanisms by which metabolically versatile non-photosynthetic plastids still conduct numerous redox reactions remain to be elucidated.

In this study, we demonstrated that a novel strain of a heterotrophic green alga, chlamydomonad sp. NrCl902, retains biosynthesis of PQ/PQH_2_. A transient RNA interference (RNAi) knockdown experiment strongly suggests that the product of homogentisate transferase gene detected in this study is involved in the synthesis of the PQ/PQH_2_ pool of this alga. In addition to sequences for Fd-FNR, the transcriptome analysis detected sequences for the plastidal, PQ/PQH_2_-mediated electron sink systems such as PTOX, which are likely to be required for carotenoid biosynthesis and redox homeostasis in the non-photosynthetic plastid. Our in-depth survey further indicates a broad distribution of the gene set for plastid electron transport systems that are simpler than that of photosynthetic plastids but more complex than that of apicoplasts. Our study thus unveiled a previously hidden step for reductive evolution of photosynthetic electron transport systems, along with the evolution of photoautotrophic algae to heterotrophic protists.

## Results and discussion

### A novel lineage of plastid-bearing, non-photosynthetic Volvocales species

**Fig. 1.**
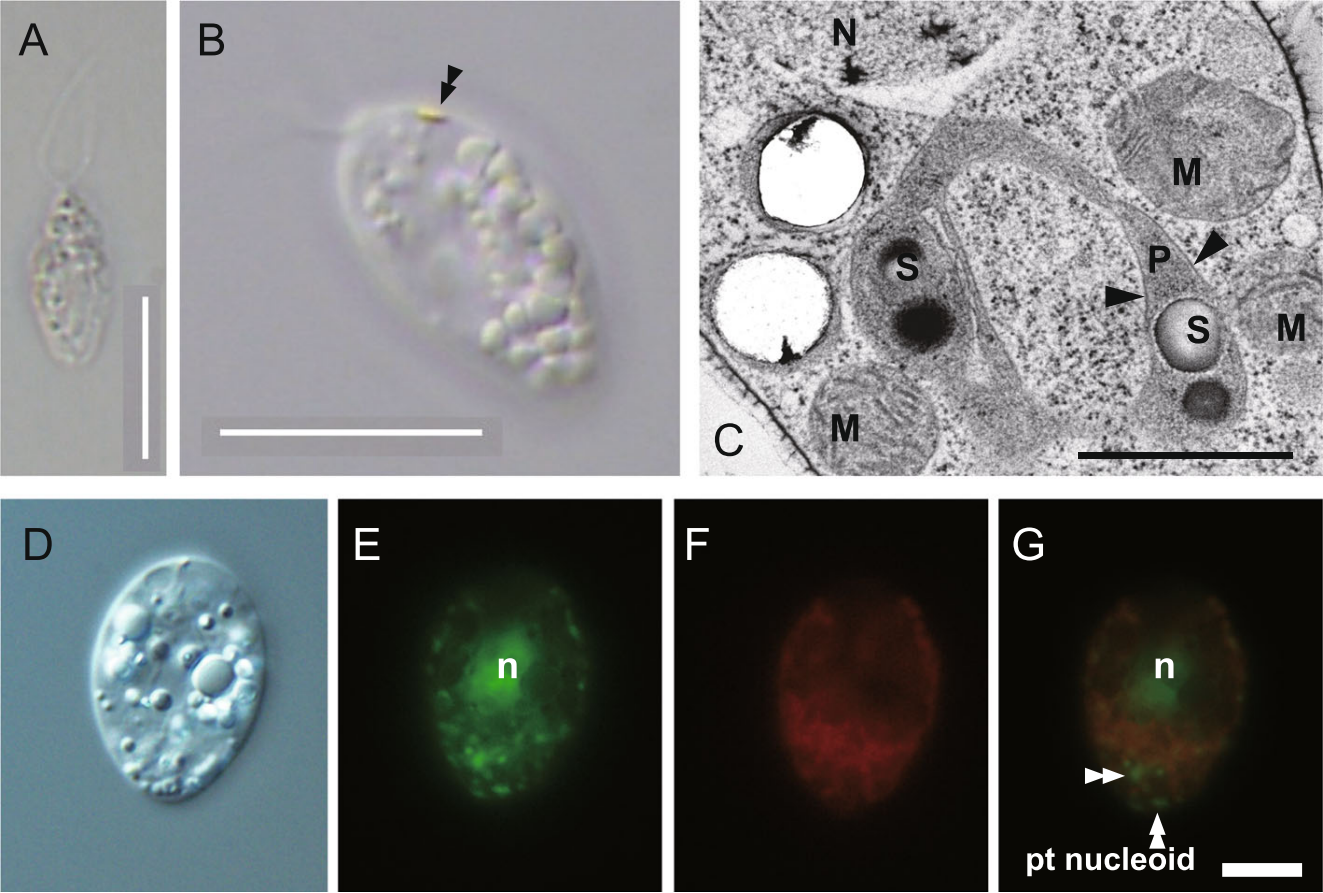
A novel strain of non-photosynthetic Volvocales green algae, chlamydomonad sp. NrCl902. **a** Light microscopic observation of a cell. Bar = 10 μm. **b** Light microscopic observation of the orange eyespot, indicated by a closed double arrowhead, at the anterior part of a cell. Bar = 10 μm. **c** Transmission electron microscopy of non-photosynthetic plastids containing starch granules. Bar = 1 μm. Arrowheads show the membranes enclosing starch granules. S: starch granule, N: nucleus, M: mitochondrion, P: plastid. **d** DIC, **e** SYBR Green, **f** Mitotracker, **g** Merge. n: nuclear DNA. SYBR Green fluorescence signals other than the nuclear DNA are highly likely organellar DNAs. Bar = 5 μm. Double arrowheads indicate SYBR Green signal not overlapped with Mitotracker signal, suggesting presence of plastid DNA (pl nucleoid)

We established and maintained the axenic, clonal culture of chlamydomonad sp. strain NrCl902, a colorless Volvocales green alga (Fig. 1a), in a medium containing sodium acetate as the sole carbon/energy source (see “Methods” section for details). The cell possesses an orange eyespot (Fig. 1b), located in plastids of photosynthetic Volvocales green algae [27]. Indeed, a membrane-bound structure containing starch granules but lacking accumulation of thylakoid membranes was observed via transmission electron microscopy (Fig. 1c). In light of these findings, we conclude that the chlamydomonad sp. possesses a non-photosynthetic plastid that is capable of synthesizing starch granules.

After SYBR Green staining, we could observe organellar DNAs in addition to the nuclear DNA (Fig. 1d, e). Most of organellar DNA signals were derived from mitochondrial DNAs as they colocalized with the Mitotracker fluorescence (Fig. 1d–g). However, some organellar DNA signals were outside the Mitotracker fluorescence (Fig. 1g), strongly suggesting some of them are derived from a plastid DNA in the new non-photosynthetic green alga.

**Fig. 2.**
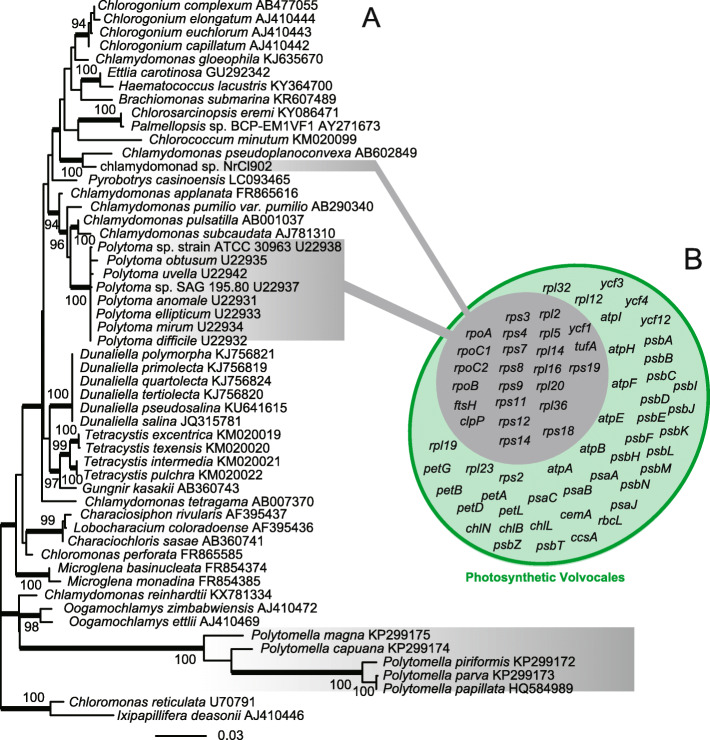
Loss of photosynthesis and convergent evolution of plastid-encoded gene repertoires in Volvocales. **a** Phylogenetic position of the chlamydomonad sp. NrCl902 in the 18S rRNA gene tree of Volvocales green algae. Non-photosynthetic lineages are highlighted in gray. Numbers at each branch show ML bootstrap values > 90%, whereas thick branches show the PhyloBayes posterior probabilities > 0.95. **b** Comparison of gene repertoires in plastid genomes of photosynthetic and non-photosynthetic Volvocales green algae. Genes highlighted in gray are those shared by plastid genomes of photosynthetic volvocales green algae and non-photosynthetic species, *Polytoma* and chlamydomonad sp. NrCl902, while genes highlighted in green are those present in plastid genomes of photosynthetic volvocales green algae but absent from those of the non-photosynthetic species. The plastid genome of *Polytoma* is represented by *P. uvella*. Protein-coding gene repertoires of the plastid genomes of *P. uvella* and chlamydomonad sp. NrCl902 are found to be identical. Note that *Polytomella* spp. are known to lack plastid DNAs completely [12]

In 18S rRNA gene phylogeny, chlamydomonad sp. NrCl902 was branched with *Chlamydomonas pseudoplanoconvexa* (Genbank accession number: AB602849), a photosynthetic species, with the highest bootstrap support and the highest PhyloBayes posterior probability (Fig. 2a). Other non-photosynthetic Volvocales green algae, *Polytoma* spp. and *Polytomella* spp., were distantly related to chlamydomonad sp. NrCl902. We removed *Polytoma oviformis* U22936 [28] from our analysis as the nucleotide sequence had been reported to be a chimera [29]. Thus, the chlamydomonad sp. NrCl902 is the third independent lineage of non-photosynthetic Volvocales species.

For the first time, we succeeded in assembling the complete plastid genome in non-photosynthetic Volvocales green algae, given a fragmented assembly of the previously published plastid genome of the non-photosynthetic Volvocales green alga *Polytomella uvella* [30]. The 176-kb-long, circularly mapping plastid genome of chlamydomonad sp. NrCl902 does not carry the genes or pseudogenes for photosynthesis-related thylakoid membrane complexes, the carbon fixation pathway, and chlorophyll biosynthesis (Additional file 1: Fig. S1). The proteins encoded in the chlamydomonad sp. NrCl902 plastid are related to proteolysis, transcription, translation, protein transport, and plastid division. Notably, the protein-coding gene repertoire of NrCl902 is completely identical with that of *P. uvella* (highlighted by gray back in Fig. 2b; Additional file 1: Fig. S1). As *P. uvella* and our strain NrCl902 have lost photosynthesis independently (Fig. 2a), the shared gene repertoire represents the convergent reductive plastid genome evolution after loss of photosynthesis.

### Plastoquinone/plastoquinol pool in the functionally versatile non-photosynthetic plastid

**Fig. 3.**
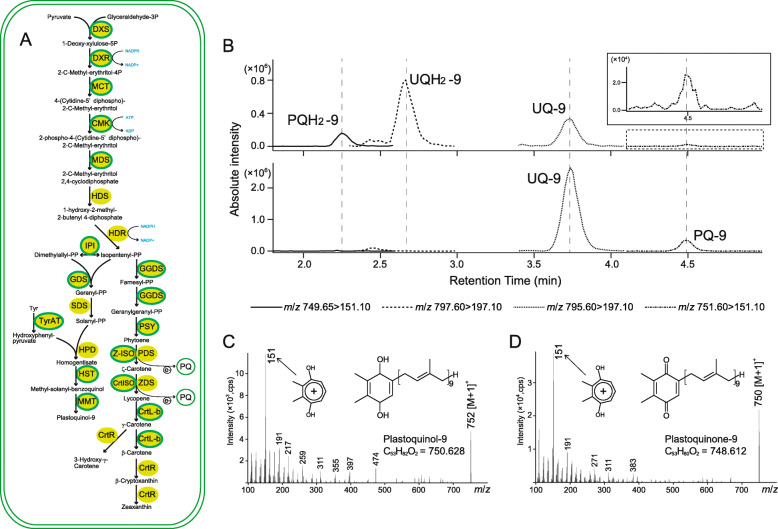
Carotenoid and plastoquinone/plastoquinol in chlamydomonad sp. NrCl902. **a** Predicted biosynthetic pathway of isoprenoids, carotenoids, and plastoquinol. Sequences in which plastid-targeting transit peptides were detected are shown by green circles with solid lines, whereas those lacking explicit plastid-targeting transit peptides are shown by green circles with no line. DXS: 1-deoxy-d-xylulose 5-phosphate synthase, DXR: 1-deoxy-d-xylulose 5-phosphate reductase, MCT: 2-C-methyl-d-erythritol 4-phosphate cytidylyltransferase, CMK: 4-diphosphocytidyl-2-C-methyl-d-erythritol kinase, MDS: 2-C-methyl-d-erythritol 2,4-cyclodiphosphate synthase, HDS: (E)-4-hydroxy-3-methyl-but-2-enyl pyrophosphate (HMB-PP) synthase, HDR: (E)-4-hydroxy-3-methyl-but-2-enyl pyrophosphate (HMB-PP) reductase, IPI: isopentenyl-diphosphate delta-isomerase, GDS: geranyl diphosphate synthase, SDS: solanesyl diphosphate synthase, TyrAT: tyrosine aminotransferase, HPD: 4-hydroxyphenylpyruvate dioxygenase, HST: homogentisate solanesyltransferase, MMT: MPBQ/MSBQ methyltransferase, GGDS: geranylgeranyl diphosphate synthase, PSY:, Z-ISO: 15-cis-ζ-carotene isomerase, PDS: phytoene desaturase, CrtISO: prolycopene isomerase, ZDS: ζ-carotene desaturase, CrtL-b: lycopene β cyclase, and CrtR: β-carotene hydroxylase (see also Table S1). **b** LC-MS/MS chromatograms (multiple reaction monitoring mode) of the directly analyzed acetone extract from chlamydomonad sp. NrCl902 (top) and the extract after oxidative treatment with ferric chloride (bottom), identifying plastoquinol-9 (PQH_2_-9), ubiquinol-9 (UQH_2_-9), plastoquinone-9 (PQ-9), and ubiquinone-9 (UQ-9). While a prominent peak at ca. 2.25 min nearly disappeared, another prominent peak appeared at ca. 4.50 min after oxidative treatment, suggesting that the latter compound was the oxidized product of the former; these were assigned as PQH_2_-9 and PQ-9, respectively, by comparing with the extract of cyanobacteria based on their retention times. Note that a trace amount of PQ-9 was present even before the oxidative treatment. **c**, **d** LC-MS/MS spectra (product ion scan mode) of compounds at ca. 2.25 and 4.50 min, confirming each as PQH_2_-9 and PQ-9, respectively

Our prediction of potential plastid metabolic functions in the non-photosynthetic green alga reveals a previously undiscovered function of non-photosynthetic plastids in this organism. We reconstructed the metabolic pathways of plastids in chlamydomonad sp. by transcriptome analyses with *C. reinhardtii* chloroplast functions as references; these included nitrite assimilation, sulfur assimilation, biosynthesis of isoprenoids, starches, fatty acids, glycerolipids, heme, chlorophylls, carotenoids, quinones, Fe-S clusters, and various amino acids, as well as carbon fixation through the Calvin Benson cycle [31]. By reciprocal blast analyses, we detected contigs encoding plastid-targeted proteins that are involved in most of the above functions, except for photosynthesis-related functions such as photosynthetic thylakoid membrane complexes, chlorophyll biosynthesis, and carbon fixation (Additional file 2: Fig. S2; Additional file 3: Table S1). It is possible that the plastid pentose phosphate pathway found in chlamydomonad sp. NrCl902 works for supply of ATP, NADPH, and erythrose 4-phosphate, the latter of which is then utilized in the shikimate pathway for aromatic amino acid biosynthesis (Additional file 2: Fig. S2), as proposed in the non-photosynthetic diatom plastid [16]. These complex metabolic pathways in the non-photosynthetic plastid are most likely fueled by cytosolic/mitochondrial metabolisms through plastid transporters such as triose phosphate transporters and glucose phosphate transporters (Additional file 4: Fig. S3; Additional file 5: Fig. S4; Additional file 6: Table S2). In addition, the plastid triose phosphate transporter would play roles for not only import of sugar phosphates but also export of glycerate 3-phosphate (Additional file 4: Fig. S3). Interestingly, sequences for the PQ/PQH_2_ biosynthetic pathway that follows isoprenoid synthesis were present in the transcriptome data (Fig. 3a in detail). To support this finding, we conducted liquid chromatography (LC)-tandem mass spectrometry (MS/MS) analyses for the detection of PQ/PQH_2_. In addition to ubiquinone and ubiquinol, which are the electron acceptor and donor, respectively, in mitochondrial oxidative phosphorylation (Additional file 4: Fig. S3; Additional file 6: Table S2), LC analysis detected an explicit, candidate peak of PQH_2_ and a faint, candidate peak of PQ (Fig. 3b). The MS spectrum confirmed that the explicit peak was PQH_2_-9 (Fig. 3c). After treatment of cell extracts with FeCl_3_ for oxidization, the PQH_2_-9 peak disappeared and the candidate peak of PQ became explicit, indicating that the faint peak detected by the initial analysis was the oxidized form of PQH_2_-9, i.e., PQ-9 (Fig. 3b). Indeed, the MS/MS spectrum indicated that the peak was most likely PQ-9 (Fig. 3d). In line with the above analyses, the non-photosynthetic plastid of chlamydomonad sp. NrCl902 was demonstrated to possess PQ-9 as the electron carrier. This is the first report of PQ/PQH_2_ pool in an organism with non-photosynthetic plastids that lack photosynthesis-related thylakoid membrane complexes.

**Fig. 4.**
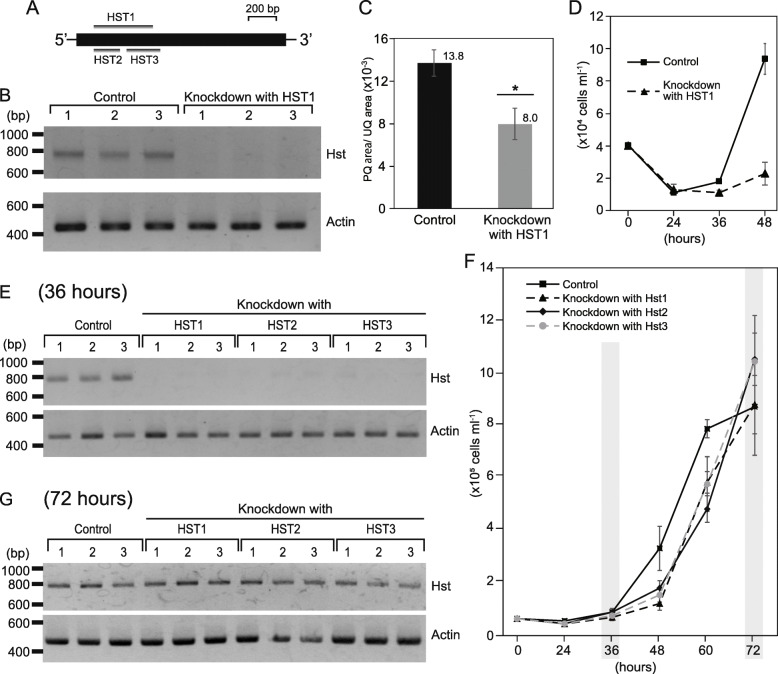
Suppression of plastoquinol synthesis. **a** Target regions of the RNAi experiment. A closed box shows the coding region of the homogentisate solanesyltransferase (HST) transcripts. Gray boxes show regions targeted by double-strand RNAs HST1-3. **b** RT-PCR assays for the transcripts of HST and actin. Lanes 1–3 (controls): RT-PCRs with total RNAs extracted from each of three cell suspensions for which electroporation was conducted without double-strand RNAs. Lanes 4–6: RT-PCRs with total RNAs extracted from each of three cell suspensions for which electroporation was conducted with HST1. RNA was extracted from cell suspensions 1.5 days after the electroporation. **c** Relative amount of plastoquinone (PQ) pool. Relative amount of quinones are evaluated as peak areas of MRM chromatogram in the LC-MS/MS analyses. Normalization of a PQ peak area was performed by a peak area of ubiquinone (UQ). Quinones were extracted from cells 2 days after the electroporation with or without HST1. All the reduced quinones, plastoquinol and ubiquinol, were oxidized with FeCl_3_ prior to the LC-MS/MS analyses, resulting in PQ and UQ. Error bar shows standard deviation. The numbers on the bars show mean values. *: *P* < 0.05 (Welch *t*-test). *N* = 3. **d** Growth of chlamydomonad sp. NrCl902 until 2 days after the electroporation. Error bar shows standard deviation. *N* = 3. **e** RT-PCR assays for the transcripts of HST and actin in RNAi experiments with additional double-strand RNAs. Lanes 1–3 (controls): RT-PCRs with total RNAs extracted from each of three cells suspensions for which electroporation was conducted without double-strand RNAs. Lanes 4–6: RT-PCRs with total RNAs extracted from each of three cells suspensions for which electroporation was conducted with HST1. Lanes 7–9: RT-PCRs with total RNAs extracted from each of three cells suspensions for which electroporation was conducted with HST2. Lanes 10–12: RT-PCRs with total RNAs extracted from each of three cell suspensions for which electroporation was conducted with HST3. RNA was extracted from cell suspensions 1.5 days after electroporation. **f** Growth of chlamydomonad sp. NrCl902 until 3 days after electroporations. Error bar shows standard deviation. *N* = 3. RT-PCRs were performed 36 and 72 hours after electroporation (highlighted in grey). **g** RT-PCR assays for the transcripts of HST and actin in RNAi experiments after 3 days from electroporation. Lanes 1–3 (controls): RT-PCRs with total RNAs extracted from each of three cell suspensions for which electroporation was conducted without double-strand RNAs. Lanes 4–6: RT-PCRs with total RNAs extracted from each of three cell suspensions for which electroporation was conducted with HST1. Lanes 7–9: RT-PCRs with total RNAs extracted from each of three cell suspensions for which electroporation was conducted with HST2. Lanes 10–12: RT-PCRs with total RNAs extracted from each of three cell suspensions for which electroporation was conducted with HST3. RNA was extracted from cell suspensions 3 days after electroporation

To further confirm involvement of the detected sequences, such as homogentisate solanesyltransferase (HST), in biosynthesis of the PQ/PQH_2_ pool of this alga, we performed a transient RNAi knockdown experiment with electroporation of the double-strand RNAs (HST1; Fig. 4a) for the gene. After the electroporation, HST transcripts were undetectable by the reverse transcriptase PCR (RT-PCR) assay while transcripts of actin, a housekeeping gene for Actin cytoskeleton, were detected as a positive control (Fig. 4b). This is in stark contrast to the control experiment for which the entire experimental procedure was followed but except for electroporation without double-strand RNAs; transcripts of both genes were detected by the RT-PCR assays (Fig. 4b). The plastid PQ/PQH_2_ pool size relative to the UQ/UQH_2_ pool size of presumably mitochondria was significantly decreased after the double-strand HST1 RNA electroporation to ca. 60% of that in the control experiments (Fig. 4c), which was confirmed by the LC-MS/MS analyses. Given these, the transient knockdown of the transcripts for HST likely caused suppression of PQH_2_ synthesis. Interestingly, cell growth has been suppressed in the knockdown samples during 2 days after the electroporation (Fig. 4d), suggesting PQ/PQH_2_ deficiency might affect cell viability. To investigate a possibility of off-target effects in the cell growth, two other distinct double-strand RNA molecules (HST2 and HST3; Fig. 4a) within the same target region of HST1 were designed for independent RNAi experiments, and suppression of both HST mRNA expression and cell growth was observed as well (Fig. 4e, f). It is noteworthy that the number of cells in the knockdown samples became comparable with that of the control cells by 3 days after the electroporation (Fig. 4f) and that the RT-PCR assays for cells 3 days after the electroporation detected both HST and actin transcripts (Fig. 4g). These observations indicate that the RNAi effect in the current condition has lasted during a few days after the electroporation.

### Possible roles of PQ/PQH_2_ pool in the non-photosynthetic plastid

PQ is known as the electron acceptor from the carotenoid biosynthetic pathway in which phytoene desaturase (PDS or CrtP) and ζ-carotene desaturase (ZDS or CrtQ) pull out electrons from carotenoid precursors [24]. Transcriptome analyses of chlamydomonad sp. NrCl902 revealed the presence of contigs encoding carotenoid biosynthesis proteins that could convert an isoprenoid to zeaxanthin (Fig. 2a; Additional file 7: Fig. S5). Indeed, high-performance liquid chromatography (HPLC) analyses demonstrated that the chlamydomonad sp. possesses several different carotenoids including β-carotene, γ-carotene, 3-hydroxy-γ-carotene, and zeaxanthin (Additional file 7: Fig. S5). Those carotenoids might be either components of the eyespot (Fig. 1b [27]) or scavengers of reactive oxygen species [32]. The non-photosynthetic green alga *Polytomella magna* (Fig. 2a) is known to have an eyespot and genes for the carotenoid biosynthesis pathway, although what carotenoids are synthesized remains unknown [13]. In addition, certain carotenoids were also detected and were thought to be antioxidants in the malaria *P. falciparum*, although the exact localization of the carotenoid biosynthesis remained unclear in the parasite [33].

**Fig. 5.**
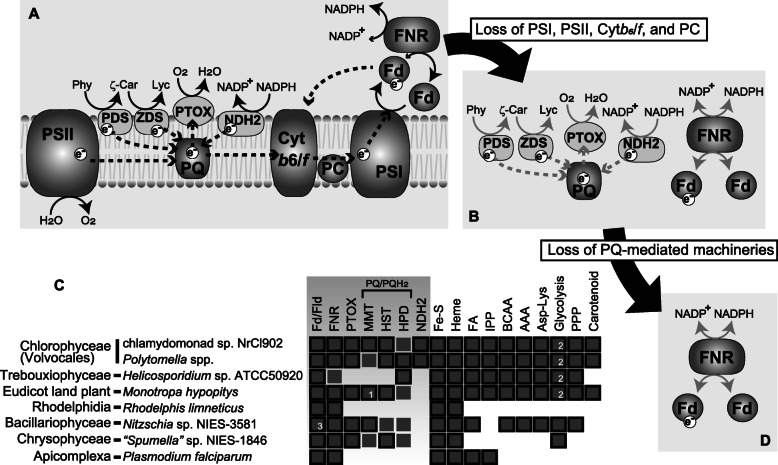
Evolution of plastid electron transport systems. **a** Photosynthetic plastid such as *Chlamydomonas reinhardtii*. The photosynthetic electron transport system comprises linear electron transport, plastid terminal oxidase (PTOX)-mediated electron sink, cyclic electron flow, and FNR-mediated NADPH-Fd electron transfer [3–6]. Solid arrows indicate the direction of reactions. Dotted arrows indicate the electron flow. **b** The non-photosynthetic plastid lacking the photosynthetic pigment system but retaining the PTOX-mediated sink of electrons and reducing power (NADPH). NADPH-Fd electron transfer was also performed. Gray arrows indicate the postulated direction and flow from the model in **a**, not experimentally confirmed. Chlamydomonad sp. NrCl902 is highly likely to possess this type of plastid. **c** Distribution of the plastid-targeted proteins/plastid functions in eukaryotes bearing non-photosynthetic plastids. Dark gray boxes enclosed by solid lines indicate the presence of corresponding proteins or functions localized in plastids, whereas those without solid lines indicate the presence of homologous sequence with no plastid-targeting sequence. Highlighted in light gray are the genes of the electron sink machineries. Data of *Polytomella* spp. (Volvocales), *Helicosporidium* sp. (Trebouxiophycea), *Monotropa hypopithys* (Eudicot land plant), *Nitzschia* sp. (Bacillariophyceae), “*Spumella*” sp. (Chrysophyceae), *Rhodelphis* spp. (Rhodelphidia), and *Plasmodium falciparum* (Apicomplexa) are derived from previous studies [8, 11–14, 16–18]. Data of *Polytomella*, *Monotropa*, *Nitzschia*, and *Spumella* are of transcriptome, while the others are of genome. **1** Only this is the plant-type while the others are the divergent-type [34]. **2** Enolase and phosphoglycerate mutase are absent. **3** Flavodoxin (Fld) is used instead of ferredoxin (Fd). **d** Non-photosynthetic plastid with only NADPH-Fd electron transfer, such as a non-photosynthetic plastid of *Helicosporidium* sp. AAA: aromatic amino acids, Asp-Lys: aspartate-lysine conversion, BCAA: branched chain amino acids, FA: fatty acids, Fe-S: iron sulfur cluster, FNR: Ferredoxin-NADP^+^ reductase, HPD: 4-hydroxyphenylpyruvate dioxygenase, HST: homogentisate solanesyltransferase, IPP: isopentenyl pyrophosphate, MMT: MPBQ/MSBQ methyltransferase, NDH2: type 2 NADH dehydrogenase, PSI: photosystem I, PSII: photosystem II, Cyt *b*6/*f*: cytochrome *b*6/*f*, PC: plastocyanin, PQ: plastoquinone, Phy: phytoene, PPP: pentose phosphate pathway, ζ-Car: ζ-Carotene, Lyc: lycopene, and e^−^: electron

If PQ is utilized as an electron acceptor in the biosynthesis of carotenoids in NrCl902, a certain electron sink system should be functional in the plastid to re-oxidize PQH_2_ to PQ. Plastid terminal oxidase (PTOX) functions in the oxidation of PQH_2_ as a key component of the plastid sink of excess electrons in photosynthetic plastids [22, 24, 25] (Fig. 5a), and the transcriptome data also contain a contig encoding PTOX with a plastid transit peptide (Additional file 3: Table S1). We thus inferred that PTOX in chlamydomonad sp. NrCl902 is involved in the oxidation of PQH_2_ generated in association with carotenoid biosynthesis (Fig. 5b).

The PQ pool is also reduced by cyclic electron transport and by chlororespiration mediated by type II NADH dehydrogenase (NDH2) in the photosynthetic model alga *C. reinhardtii* [23–25] (Fig. 5a), in addition to carotenoid biosynthesis. We further investigated the candidate roles of PQ/PQH_2_ in the non-photosynthetic green alga chlamydomonad sp. NrCl902. We detected a sequence for NDH2 with a plastid transit peptide (Additional file 3: Table S1). Because the presumably plastidal NDH2 likely catalyzes the redox exchange between NADPH and PQ [25], PQ/PQH_2_ pool may also be involved in a part of the electron transport system to the terminal electron sink PTOX from NADPH. If correct, NDH2 together with PQ/PQH_2_ may be a component of an electron transport system for chlororespiration in the non-photosynthetic plastid of our strain NrCl902 (Fig. 5b), as chlororespiration has been proposed to be involved in NADP^+^/NADPH homeostasis previously [26].

In addition to the possible roles of the PQ/PQH_2_ pool as the electron sink systems for redox homeostasis in the non-photosynthetic green algal plastid, it cannot be completely ruled out the PQ/PQH_2_ pool has other roles. For example, it is also possible that nuclear gene expression is regulated by monitoring the redox state of the PQ/PQH2 pool as proposed in photosynthetic organisms [35].

### Broad distribution of electron transport systems in eukaryotes with non-photosynthetic plastids

Intriguingly, the chlamydomonad sp. NrCl902 transcriptome data revealed two additional sequences related to the plastid electron transport system, plastid-targeted Fd and FNR. Although their exact roles in the non-photosynthetic green alga NrCl902 are unclear, the FNR of the non-photosynthetic plastid may reversely function to reduce Fd by NADPH-derived electrons as demonstrated in the cyanobacterial heterocyst where linear electron transport is absent [36] and in non-photosynthetic plastids of land plants with root-type FNR [37]. The reverse electron transport from NADPH to Fd could explain how the non-photosynthetic plastid forms the reduced Fd required for reduced Fd-dependent nitrite and sulfate assimilation, reduction of oxidized thioredoxin by ferredoxin:thioredoxin reductase, and for Fe-S cluster assembly (Additional file 2: Fig. S2; Additional file 3: Table S1) [8, 38–41]. Otherwise, the plastid Fd-FNR system might also contribute to NADP^+^/NADPH homeostasis by re-oxidizing excess NADPHs [26] in the non-photosynthetic green algal plastid.

It was unclear how broadly genes for the plastid redox homeostasis distribute in non-photosynthetic algae/land plants, while only the plastid Fd-FNR system in apicoplasts and Rhodelphidia [9, 10, 18] and PTOX in a non-photosynthetic chrysophyte [17] have been reported. Our additional in-depth survey suggests that the NADPH-Fd electron transport system and the PQ/PQH_2_-mediated electron transport system are still retained in various non-photosynthetic algae/plants. Plastid Fd and FNR are conserved in all the examined algae/land plants with non-photosynthetic plastids, though we detected plastid flavodoxin (Fld), a functional analog of ferredoxin, in a non-photosynthetic diatom [42] (Fig. 5c). Therefore, the electron transport between Fd/Fld and NADP(H) mediated by FNR is highly likely one of the indispensable functions for maintaining non-photosynthetic plastids. In contrast, the sequences for NDH2, PTOX, and PQH_2_ synthesis show a rather punctate distribution (Fig. 5c; Additional file 8: Table S3; Additional file 9: Table S4); NDH2 sequences are detected only from green algae, sequences for PTOX and PQH_2_ synthesis from a diatom, a chrysophyte, a land plant and green algae. The organisms used in this comparative analysis have lost photosynthesis independently [8–19], indicating convergent evolution towards retention of genes for NDH2, PTOX, and PQH_2_ synthesis. Note that some of the sequences for PQ/PQH_2_ biosynthesis obtained from the transcriptome data lack N-terminal plastid-targeting sequences (Fig. 5c; Additional file 8: Table S3). It remains unclear whether the lack of plastid-targeting sequence in some sequences is caused by their incomplete, 5′ truncate sequences in the transcriptome data or reflects their localization outside plastids. Phylogenetic analyses of the key proteins, FNR, PTOX, and methyl-6-solanyl-1,4-benzoquinol methyltransferase (MMT) for the last step of PQH_2_ synthesis, show no clear evidence of lateral transfer, but rather many of the sequences are suggested to be of vertical inheritance (Additional file 10: Fig S6, Additional file 11: Fig S7, Additional file 12: Fig. S8). In addition to chlamydomonad sp. NrCl902, through vertical inheritance, multiple distinct lineages with non-photosynthetic plastids might retain or might have retained the functional, abovementioned PTOX- and PQ/PQH_2_-mediated electron transport system until recently (Fig. 5c). Whether these species bearing the genes are indeed capable of synthesizing PQ/PQH_2_ should be investigated biochemically.

### Conclusion: evolutionary principle of the electron transport system

Plastids are widely known as light-dependent cellular powerhouse organelles supplying sugar through photosynthesis to the algal and plant cells [1–6]. However, research over the past decade has shown that plastids exist on a functional and evolutionary continuum that includes a variety of non-photosynthetically functioning organelles [7, 8, 11–21]. Nevertheless, the evolutionary transitions of the electron transport system and the branched pathways for the electron sink between photosynthetic and non-photosynthetic plastids have remained unclear. Our findings clearly demonstrate that the entire photosynthetic electron transport system (Fig. 5a) is not always straightforwardly evolved to the simplest form comprising only Fd and FNR, soon after loss of photosynthesis (Fig. 5d). Instead, some non-photosynthetic plastids have the PQ/PQH_2_-mediated electron transport system, and they might reflect an intermediate step for reductive evolution of the plastid electron transport system (Fig. 5a, b, and d). First, associated with loss of photosynthesis, components for the photosynthetic electron transport system are lost, i.e., PSI, PSII, cytochrome *b6*/*f*, and plastocyanin, while the PQ/PQH_2_-mediated electron transport system and the Fd-FNR system are retained (Fig. 5b). Subsequently, only Fd-FNR system is left after loss of PQ/PQH_2_-mediated electron transport system as seen in a non-photosynthetic plastid of the trebouxiophycean green alga *Helicosporidium* sp. (Fig. 5d). Given multiple non-photosynthetic plastid-bearing species with genes to synthesize PQH_2_ (Fig. 5c), plastids in the green alga NrCl902 and some other non-photosynthetic algae/plant might belong to the evolutionary stages shown in Fig. 5b. Metabolic pathways that require redox reactions such as glycolysis and/or the pentose phosphate pathway might be a key constraint against the evolutionary loss of PQ/PQH_2_ pool. In this point of view, the *Helicosporidium* plastid might have an alternative way for redox homeostasis as its genome lacks PTOX and PQ/PQH_2_ biosynthesis (Fig. 5c) regardless of plastid-targeted proteins for a variety of biochemical reactions [11]. Otherwise, the complex metabolic pathways retained in the *Helicosporidium* plastid might be no longer functional efficiently and thus might require no efficient system for redox homeostasis. In contrast, “*Spumella*” sp. likely lacks various pathways but possesses genes for PTOX and some homologs for PQ/PQH_2_ synthesis (Fig. 5c [17]), suggesting multiple evolutionary routes towards loss of PTOX and PQ/PQH_2_ biosynthesis. Additional systematic analyses exploring the links between reductive evolution of the electron transport system and metabolisms with redox reactions in the non-photosynthetic plastids of diverse eukaryotes may provide further insights into the principle that would govern the functional reduction of plastids.

## Methods

### Cultivation of strain

Chlamydomonad sp. NrCl902 was isolated by a single-cell isolation technique with a glass pipet, from a freshwater sediment sample collected from a paddy field in Niinomi, Sammu, Chiba, Japan (35° 37′ 01″ N, 140° 24′ 59″ E). The cell was grown and maintained in the AFAC medium [43] at 25 °C under the dark condition. The culture was deposited to Microbial Culture Collection of National Institute for Environmental Studies (NIES [44]), Japan, as NIES-4405.

### Cell observation

Cell shape was observed by the CCD Camera DP74-CU (Olympus) equipped with a light microscope (Zeiss). To stain DNA, SYBR Green I nucleic acid stain (Thermo Fisher Scientific) was added to the cell suspension to give a final dilution of 1:1000. To stain mitochondrial membranes, MitoTracker CMTM Ros (Thermo Fisher Scientific) were added to the cell culture to give a final concentration of 200 nmol L^-1^. After staining, cells were observed using BX51 fluorescence/differential interference microscope (Olympus) connected to DP72 charge-coupled device camera. Ultrastructure was observed by transmission electron microscopy as follows. Cells were frozen in liquid propane at − 175 °C and substituted with 2% glutaraldehyde, 0.5% tannic acid in acetone, and 2% distilled water at − 80 °C for 2 days. The sample was incubated at 4 °C for 2 h, following to incubation at − 20 °C for 2 h. The sample was rinsed with acetone 4 times for 15 min each and fixed with 2% osmium tetroxide (OsO_4_) in acetone at 4 °C for 60 min. Dehydration was performed with ethanol 3 times for 30 min each, followed by additional dehydration with ethanol at room temperature overnight. The sample was infiltrated with propylene oxide 2 times for 30 min each and put into a 70:30 mixture of propylene oxide and resin (Quetol-651; Nisshin EM Co.) for 1 h. After volatilization of propylene oxide, the sample was transferred into a fresh 100% resin, followed by polymerization at 60 °C for 48 h. The resin block was ultrathin-sectioned at 70 nm with a diamond knife using a ultramicrotome (ULTRACUT, Leica), and then the sections were placed on the copper grids. The sections were stained with 2% uranyl acetate at room temperature for 15 min, rinsed with distilled water, and secondarily stained with lead stain solution (Sigma-Aldrich Co.) at room temperature for 3 min. The sections were observed by a transmission electron microscope (JEN-1400Plus; JEOL Ltd.) at an acceleration voltage of 80 kV. Digital images (3296 × 2472 pixels) were taken with a CCD camera (EM-14830RUBY2; JEOL Ltd.).

### Phylogenetic position of chlamydomonad sp. NrCl902

Nuclear small subunit rRNA gene sequence of NrCl902 was retrieved from the nuclear DNA sequencing data obtained with Illumina (see below). The rRNA sequence of NrCl902 was aligned with those of Volvocales species by MAFFT [45] and ambiguously aligned sites were removed with Bioedit [46]. The dataset comprised of 54 taxa and 1726 sites was subjected to phylogenetic analyses with PhyloBayes 4.1 [47] and IQtree 1.6.5 [48] for Bayesian and maximum likelihood frameworks, respectively. The best-fitting available model based on the Bayesian Information Criterion was the TIM3e + I + Γ model, which was used for estimation of the maximum likelihood tree and for a bootstrap analysis with the 100 pseudoreplicates. PhyloBayes analyses were performed under the CAT-GTR + Γ model with two independent Markov chain Monte Carlo chains (MCMC) were run for 50,000 trees, sampling every 100 trees, with burnin of 12,500 trees. Two chains converged with maxdiff = 0.09. Subsequently, the consensus tree with branch lengths and Bayesian posterior probabilities (BPPs) were calculated from the rest of the sampled trees. The 18S rRNA gene sequence of NrCl902, and the dataset used for the analysis are attached in Additional file 13: Datasets.

### Organellar DNA sequencing

Total DNA was extracted with Plant DNA extraction kit (Jena Biosciences) according to the manufacturer’s instruction. Total DNA was sent to Hokkaido System Science Co. for subjecting to library construction with v2 / TruSeq DNA PCR-free Sample Prep Kit (Illumina) and HiSeq2500 sequencing, resulting in 45.9 million paired-end reads. Adapter trimming and quality filtering were performed with fastX toolkit [49]. In quality filtering, reads with quality scores > 20 for at least 75% of their length were retained, resulting in 36.9 million paired-end reads. The filtered short reads were subjected to KmerGenie 1.7044 [50] to predict an assembled genome size which is the required parameter for the following HGAP-based assembling. DNA was also sequenced by PacBio RSII, with SMRT cell 8Pac V3 and DNA Polymerase Binding Kit P6 v2, in Macrogen, and the resultant 1.6 Gb subreads were subjected to assembling by HGAP v3 [51] through DDBJ Pipeline [52]. By the tblastN search [53] with plastid-encoded protein sequences of *Chlamydomonas reinhardtii* (GenBank no. BK000554), a single contig derived from a plastid genome was detected. For error correction, we mapped the filtered Illumina short reads onto the contig with Bowtie2 [54] with default settings, and detected errors on the PacBio contig were manually corrected. By using PCR assay followed by the Sanger sequencing to fulfill the gap between the termini of the contig, we obtained the complete sequence of plastid genome with 176,432 bp in length. Protein-coding genes were identified with Mfannot [55] and blastX-based homology search to the nr database of GenBank [53]. Transfer RNA genes were identified with Mfannot [55] and tRNAscan [56]. Similarly, the mitochondrial contig was detected and annotated by comparison with the mitochondrial DNA sequence of *C. reinhardtii* (GenBank no. EU306622 [57]) and its close relative *C. leiostraca* (GenBank no. KP696389 [58]).

### Nuclear-encoded protein sequences

Total RNA was extracted with Trizol (Thermo Fisher Scientific) according to the manufacturer’s instruction. RNA was subjected to library construction with TruSeq RNA Sample Prep Kit v2 (Illumina) and HiSeq2500 sequencing, resulting in 43.7 million paired-end reads. Adapter trimming and quality filtering were performed with fastX toolkit [49]. In quality filtering, reads with quality scores > 20 for at least 75% of their length ≥ 50 bp long were retained, resulting in 21.8 million paired-end reads. Assembling was performed by trinity 2.4.0 with default settings. The *C. reinhardtii* chloroplast functions for photosynthesis; nitrite assimilation; sulfur assimilation; biosynthesis of isoprenoids, starches, fatty acids, glycerolipids, heme, chlorophylls, carotenoids, Fe-S cluster, and various amino acids; and carbon fixation through the Calvin Benson cycle were partly comprised of glycolysis and the pentose phosphate pathway [31]. By using tblastN [53] with the *C. reinhardtii* sequences involved in the above plastid functions as queries with a cut off criterion of e^− 10^, we searched for homologous sequences in the transcriptome data of chlamydomonad sp. NrCl902. We then confirmed that the detected sequences are not distant paralogues by using blastP with deduced amino acid sequences as queries against the non-redundant protein database [53]. To check whether encoded proteins in the detected sequences possess plastid-targeting transit peptides at the N-termini, ChloroP 1.1 [59] was subjected with a cutoff value of 0.5 (Additional file 3: Table S1). We also surveyed sequences for the cytosolic glycolysis/gluconeogenesis, the acetate metabolisms, and the mitochondrial tricarboxylic cycle as described above. Mitochondrial targeting sequences were surveyed by Mitofates [60].

### Survey of plastid-targeted proteins for electron transport systems in other non-photosynthetic algae/plant lineages

Plastid-targeted proteins for electron transport systems (Fig. 5c) were surveyed as described above for transcriptome data of *Polytomella* spp. [12, 13], *Monotropa hypopitys* [14], *Nitzschia* sp. [16], and “*Spumella*” sp. [17], and genome data of *Helicosporidium* sp. [11], *Rhodelphis limneticus* [18], and *Plasmodium falciparum* [8]. Before the analyses, we checked the quality of the transcriptome data with gVolante [61]. The BUSCO v2 “complete + partial” scores with 303 eukaryote conserved proteins by gVolante were 94.06% in NrCl902, 93.73% and 92.74% in *Polytomella parva* and *Polytomella magna*, respectively, and 93.4% in *Monotropa*. The scores for the transcriptome data of *Nitzschia* and *Spumella* used in this study were already reported to be higher than 80% [16, 17]. Detected sequences are shown in Additional file 8: Table S3 and 9: Tables S4, respectively. Plastid-targeting sequences of detected homologs were investigated as performed in previous studies [11–18]. Phylogenetic analyses of FNR, PTOX, and MMT for the last step of PQH_2_ synthesis were performed with IQtree 1.6.5 [48] with 100 bootstrap analyses. Details of the datasets and the used models are described in legends for Figs. S6-S8 (Additional file 10: Fig. S6, Additional file11: Fig. S7, Additional file 12: Fig. S8). Datasets used for the analyses are attached in Additional file 13: Datasets.

### Detection of carotenoids and quinones/quinols

The pigments including carotenoids were extracted with acetone/methanol (7:2, v/v) using an ultrasonicator. Each pigment was separated using a C8-HPLC column [62] equipped in the Separations Module Waters 2695 (Waters) and then detected by the Photodiode Array Detector Waters 2996 (Waters) and the Multi λ Fluorescence Detector Waters 2475 (Waters). Carotenoids were identified on the basis of their retention time and characteristic absorption spectra. We also confirmed the identification of each carotene with a method using a C18-HPLC column [63].

For quinone/quinol extraction, an aliquot of 2-propanol (LC-MS grade, Kanto Chemical Co., Inc., Tokyo, Japan) were added to a microtube containing the pelleted fresh cells of chlamydomonad sp. NrC1902 and then placed in an ice-cooled ultrasonication bath for extraction for 1 min. The supernatant was immediately separated from suspends by centrifugation. A half of the supernatant was directly injected into the HPLC apparatus for analysis. The other half was treated with ferric chloride (final concentration, 1.2 mM) before the analysis for oxidation of total quinones and quinols. The LC-MS/MS instrument was composed of a Shimadzu Nexera X2 liquid chromatography system, comprising a CBM-20A communication bus module, two DGU-20A3R/5R HPLC degassing units, three LC-30AD solvent delivery units constituting a ternary pumping system, an SIL-30 AC autosampler, a CTO-20 AC column oven, and a LC-MS-8030 triple quadrupole mass spectrometer connected through an atmospheric pressure chemical ionization (APCI) interface (Shimadzu, Kyoto, Japan). The system was coupled to a personal computer configured to run the Shimadzu LabSolution software. Reverse-phase HPLC was performed on a Zorbax Eclipse Plus C18 column (Rapid Resolution HT, 3.0 × 100 mm, 1.8 μm silica particle size; Agilent Technologies, Santa Clara, USA) with the binary gradient (flow rate of 0.5 mL min^-1^) of ethyl acetate (LC-MS grade, Honeywell, Seelze, Germany) with 0.1% [v/v] formic acid (LC-MS grade, Wako Pure Chemical Industries, Ltd., Osaka, Japan) in methanol (LC-MS grade, Kanto Chemical Co., Inc., Tokyo, Japan) with 0.1% [v/v] formic acid as follows (all v/v); 30% for 1.0 min, 30–80% in 4.0 min, 80% for 3 min, 80–30% in 0.1 min, and 30% for 3.9 min. All the mobile phases were degassed in vacuo with ultrasonication. The mobile-phase reservoir bottles were designed to prevent any contact between the mobile phases and air during analysis. The APCI was set as following conditions: nebulizer gas flow, 3.0 L min^− 1^; interface temperature, 350 °C; desolvation line temperature, 200 °C; heat block temperature, 200 °C; drying gas flow, 5 L min^− 1^. Parameters of the multiple reaction monitoring (MRM) in the positive ion mode of the mass spectrometer were summarized in Additional file 14 (Table S5). Parameters of the Q3 product ion scan in the positive ion mode of the mass spectrometer were summarized in Additional file 15 (Table S6).

### RNAi knockdown of homogentisate solanesyltransferase

In silico prediction of suitable regions in the homogentisate solanesyltransferase (HST) transcript for RNAi was performed with siDirect 2.0 [64], unveiling accumulation of the suitable regions at ca. 600 bp of the 5′ terminal region (data not shown). Therefore, we decided to prepare three double-strand RNA molecules for the 5′ terminal region (Fig. 4a) as follows. One microgram of total RNA extracted from cells of chlamydomonad sp. NrCl902 with Trizol (Thermo Fisher Scientific) was subjected to cDNA synthesis with 3′ RACE System for Rapid Amplification of cDNA Ends (Thermo Fisher Scientific) according to the manufacturer’s instructions. PCR assay with the primer set, 5′-AGCCTGAATAATGGCGCAAG-3′ and 5′-TGACGAAGGCGGTGATGAAG-3′, was performed in order to obtain DNA fragments of homogentisate solanesyltransferase (HST). Using the HST DNA fragment as the template, the nested PCR was performed with primers (HST1F: 5′-CTAATACGACTCACTATAGGGAGAATAATGGCGCAAGATCAGCTTC-3′ and HST1R: 5′-CTAATACGACTCACTATAGGGAGACTTGTTCACCACGTCAATGTCC-3′) in order to prepare the DNA fragment attached with the T7 promoter at the 5′ end, which is subsequently used as the template for double-strand RNA synthesis with MEGA script RNAi Kit (Thermo Fisher Scientific) equipped with T7 RNA polymerase. The double-strand RNA molecule prepared as describe above is called HST1. To reduce the possibility of off-target effect, we additionally prepared two distinct double-strand RNA molecules, called HST2 and HST3, targeting the HST transcript (Fig. 4a). RT-PCR assays were performed using the following primer sets: HST2F: 5′-CTAATACGACTCACTATAGGGAGAAGCCTGAATAATGGCGCAAGAT-3′ and HST2R: 5′-CTAATACGACTCACTATAGGGAGACACCTGGCTGTCATTTGTGGA-3′, and HST3F: 5′-CTAATACGACTCACTATAGGGAGAAAATTCAGCCATGCGTTTTGG-3′ and HST3R: 5′-CTAATACGACTCACTATAGGGAGACCACACCGGTTGACATCTCG-3′. The synthesized PCR products were used as the templates for double-strand RNA synthesis as described above.

Chlamydomonad sp. NrCl902 were preliminarily cultivated as described above for 4 days. The cultivated cells were further incubated in a fresh AFAC medium for 24 h. Cells were collected by centrifugation at 2500 rpm for 5 min and washed with the TAP medium (Thermo Fisher Scientific) containing 40 mM sucrose. The 8.0 × 10^5^ cells were suspended in 40 μL of the sucrose-containing TAP medium. The cell suspension was placed into an electroporation cuvette with a 2-mm gap (NEPAGENE). By using NEPA21 Super Electroporator (NEPAGENE), electroporation was performed with no double-strand RNAs or 4 μg of HST1 double-strand RNAs for nine cell suspension samples, each containing 8.0 × 10^5^ cells in the sucrose-containing TAP medium under the following electroporation conditions reported in Yamano et al. [65] with some modifications. For details, parameters of poring pulse were one polarity-exchange pulse of 300 V with 8 ms pulse length, 50 ms pulse interval, and a 40% decay rate, while those of transfer pulse were a ten polarity-exchanged pulse of 20 V with 50 ms pulse length, 50 ms pulse interval, and a 40% decay rate. In this case, the measured value of electrical impedance was within 440–500 Ω in the cell conditions described above. Of nine cell suspension samples, three were subjected to cell counting, three others to the RT-PCR assays, and the others to the PQ/PQH_2_ detection/quantification. Electroporated cells in the TAP medium were transferred to the AFAC medium and cultivated as described above, under the dark condition. Cells were counted every 12 h under the light microscopy until 48 h after electroporation. Total RNA was extracted from cells 1.5 days after the electroporation as described above. RT-PCR assays with the total RNA as the template were conducted with the primer set for HST (5′-AGCCTGAATAATGGCGCAAG-3′ and 5′-TGACGAAGGCGGTGATGAAG-3′) or with that for actin (5′-ACTCATACGTCGGTGATGAG-3′ and 5′-GCTCCATCAAGATCTTCATC-3′). Cells 2 days after electroporation were subjected to quinone/quinol extraction as described above. The quinols in the total extract was oxidized into quinones with ferric chloride before analyses described above.

To reduce the possibility of off-target effect, six cell suspensions were subjected to the electroporation with no double-strand RNAs or each of HST1, HST2, and HST3 double-strand RNAs as described above: of six cell suspension samples, three were for the RT-PCR assays at 1.5 days after the electroporation and the others for cell counting for 3 days after the electroporation. Note that after the cell counting, the cells were used for the RT-PCR assays to check the RNAi effect after 3 days from the electroporation.

## Supplementary information

**Additional file 1. Figure S1.** Plastid genome of chlamydomonad sp. NrCl902. Dark gray boxes show canonical plastid genes. Closed boxes show genes with no homolog in other organisms and intronic ORFs. Light gray boxes between dark gray ones show introns. Thin bars show tRNA genes.

**Additional file 2. Figure S2.** Predicted plastid metabolic map of chlamydomonad sp. NrCl902. 1. Heme synthesis, 2. branched chain amino acid synthesis, 3. sulfate assimilation, 4. Fe-S cluster synthesis, 5. aspartate-to-lysine conversion, 6. starch metabolism, 7. pentose phosphate pathway, 8. nitrite assimilation, 9. aromatic amino acid biosynthesis, 10. fatty acid synthesis, and 11. glycerolipid synthesis. Sequences in which plastid-targeting transit peptides were detected are shown by green circles with solid lines, while those lacking explicit plastid-targeting transit peptides are shown by green circle with no line. Details for isoprenoid (IPP), carotenoid, and plastoquinone syntheses are depicted in Fig. 3a. Substrate possibly imported from the cytosol and/or mitochondria are highlighted in blue, while those possibly exported to contribute to the cytosolic and mitochondrial functions are in red. Abbreviations of proteins are explained in Additional file 3 (Table S1).

**Additional file 3. Table S1.** Plastid-targeted protein sequences detected in the transcriptome data of chlamydomonad sp. NrCl902.

**Additional file 4. Figure S3.** Interaction between the non-photosynthetic plastid and other compartments. Protein sequences with clear plastid-targeting sequences are shown by light green circles enclosed by solid lines. Protein sequences with clear mitochondrial targeting sequences are shown by orange circles enclosed by red lines. Protein sequences with neither targeting sequence are shown by light blue circles. “?”: Aconitase that catalyzes the conversion from citrate to isocitrate outside mitochondria was not detected in the transcriptome data of chlamydomonad sp. NrCl902. Translation initiation from the 2nd methionine of the mitochondrial aconitase gene might express Aconitase functioning outside mitochondria. Abbreviations are explained in Tables S1 and S2.

**Additional file 5. Figure S4.** Mitochondrial genome of chlamydomonad sp. NrCl902. Conserved mitochondrial genes are shown by closed boxes, while intronic open reading frames are shown in gray. Intron regions are shown as open boxes. L1-L6 and S1-S3 show large subunit and small subunit rRNA gene fragments. Transfer RNA genes are shown by their amino acids and anticodons in parentheses. Given a variety of structures of mitochondrial genomes in Volvocales, i.e., circular genomes and tandem repeats of linear genome, it remains unclear whether the mitochondrial genome is a circularly mapping molecule or a linear, tandemly repeated molecule.

**Additional file 6. Table S2.** Mitochondrial and cytosolic protein sequences for carbon and energy metabolisms detected in the transcriptome data of chlamydomonad sp. NrCl902.

**Additional file 7. Figure S5.** Carotenoid biosynthesis in chlamydomonad sp. NrCl902. A. The detailed pathway for carotenoid biosynthesis and structures of carotenoids predicted to be synthesized in this pathway. B. HPLC profile for carotenoid detection by absorbance at 450 nm in chlamydomonad sp. NrCl902.

**Additional file 8. Table S3.** Detected sequences for electron transport systems in non-photosynthetic species of algal and plant lineages.

**Additional file 9. Table S4.** Detected sequences for representative plastid metabolic functions in the non-photosynthetic eudicot plant *Monotropa hypopytis*.

**Additional file 10. Figure S6.** Maximum likelihood tree of plastid terminal oxidase in eukaryotes. Non-photosynthetic algae are highlighted in black. Numbers on branches are bootstrap values equal to or higher than 80%. The dataset comprised of 67 taxa and 236 sites was analyzed with IQtree under the LG + I + Γ model selected with Bayesian Information Criterion.

**Additional file 11. Figure S7.** Maximum likelihood tree of Ferredoxin:NADP+ oxidoreductase in eukaryotes. Non-photosynthetic algae are highlighted in black. Numbers on branches are bootstrap values equal to or higher than 80%. The dataset comprised of 96 taxa and 303 sites was analyzed with IQtree under the LG + I + Γ model selected with Bayesian Information Criterion.

**Additional file 12. Figure S8.** Maximum likelihood tree of MPBQ/MSBQ methyltransferase in eukaryotes. A. Plant-type MMT. The dataset comprised of 17 taxa and 250 sites was analyzed with IQtree under the LG + Γ model selected with Bayesian Information Criterion. B. Divergent type MMT. The dataset comprised of 35 taxa and 292 sites was analyzed with IQtree under the LG + I + Γ model selected with Bayesian Information Criterion. Non-photosynthetic algae are highlighted in black. Numbers on branches are bootstrap values equal to or higher than 80%.

**Additional file 13.** Datasets for phylogenetic analyses.

**Additional file 14. Table S5.** Parameters of the multiple reaction monitoring (MRM) in the positive ion mode of the mass spectrometer used in the present study.

**Additional file 15. Table S6.** Parameters of the Q3 product ion scan in the positive ion mode of the mass spectrometer used in the present study.

## Data Availability

DNA and RNA sequencing data of chlamydomonad sp. NrCl902 were deposited to DDBJ (BioProject Accessions, PRJDB10134 [66] and PRJDB9052 [67], respectively). DDBJ accession numbers of the plastid and mitochondrial genomes are LC516060 [68] and LC516061 [69], respectively. The culture strain NrCl902 used in this study is available in National Institute for Environmental Studies (NIES [44]), Japan, as NIES-4405.
