# Supplementary figures and images for: A non-photosynthetic green alga illuminates the reductive evolution of plastid electron transport systems
Source: BMC Biol. 2020 Sep 16;18:126. doi: 10.1186/s12915-020-00853-w (PMC7495860; doi:10.1186/s12915-020-00853-w)

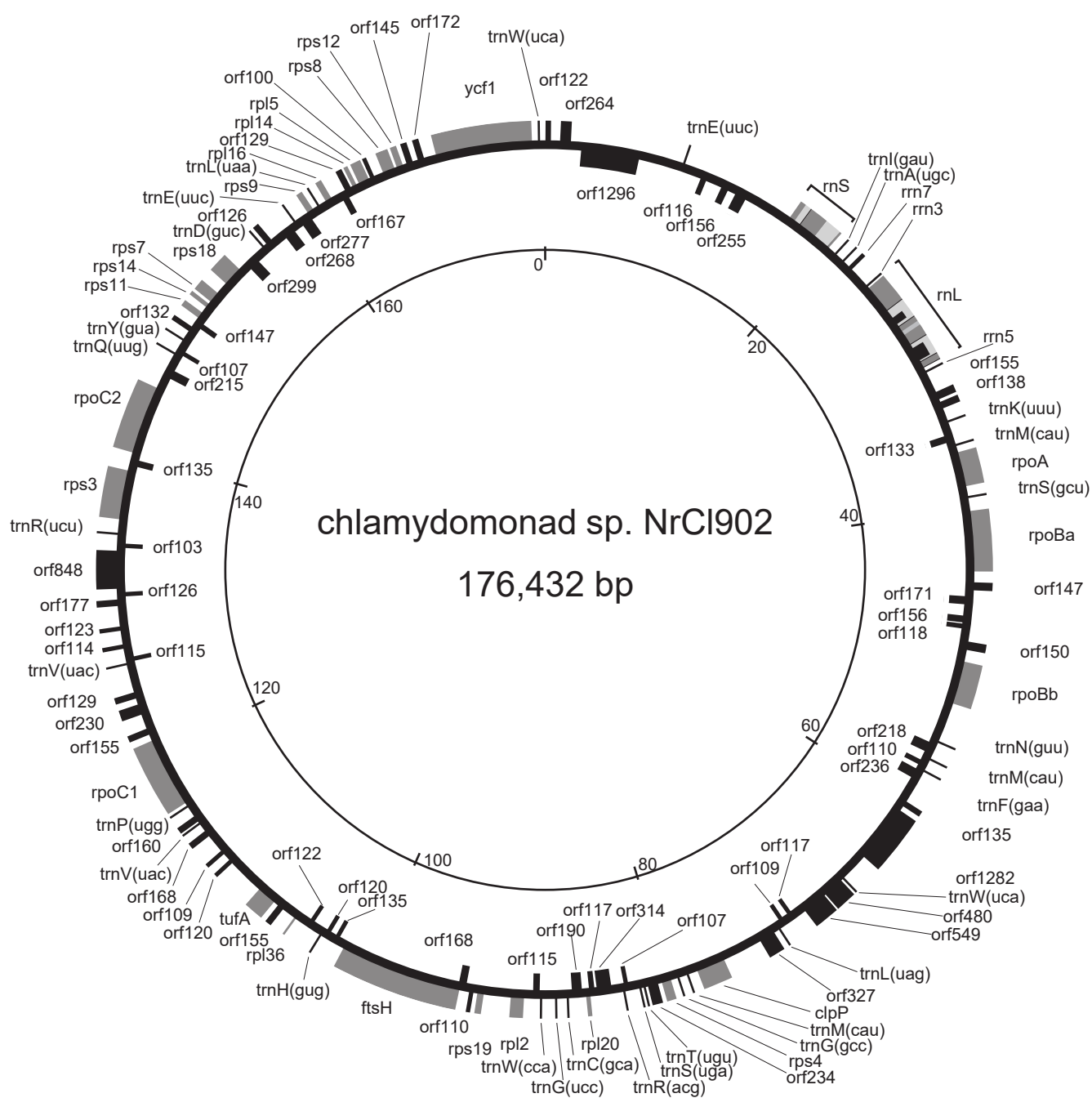

Fig. S1

Supplement: Supplementary file 1 — Additional file 1. Figure S1. Plastid genome of chlamydomonad sp. NrCl902. Dark gray boxes show canonical plastid genes. Closed boxes show genes with no homolog in other organisms and intronic ORFs. Light gray boxes between dark gray ones show introns. Thin bars show tRNA genes. [file 12915_2020_853_MOESM1_ESM.pdf]

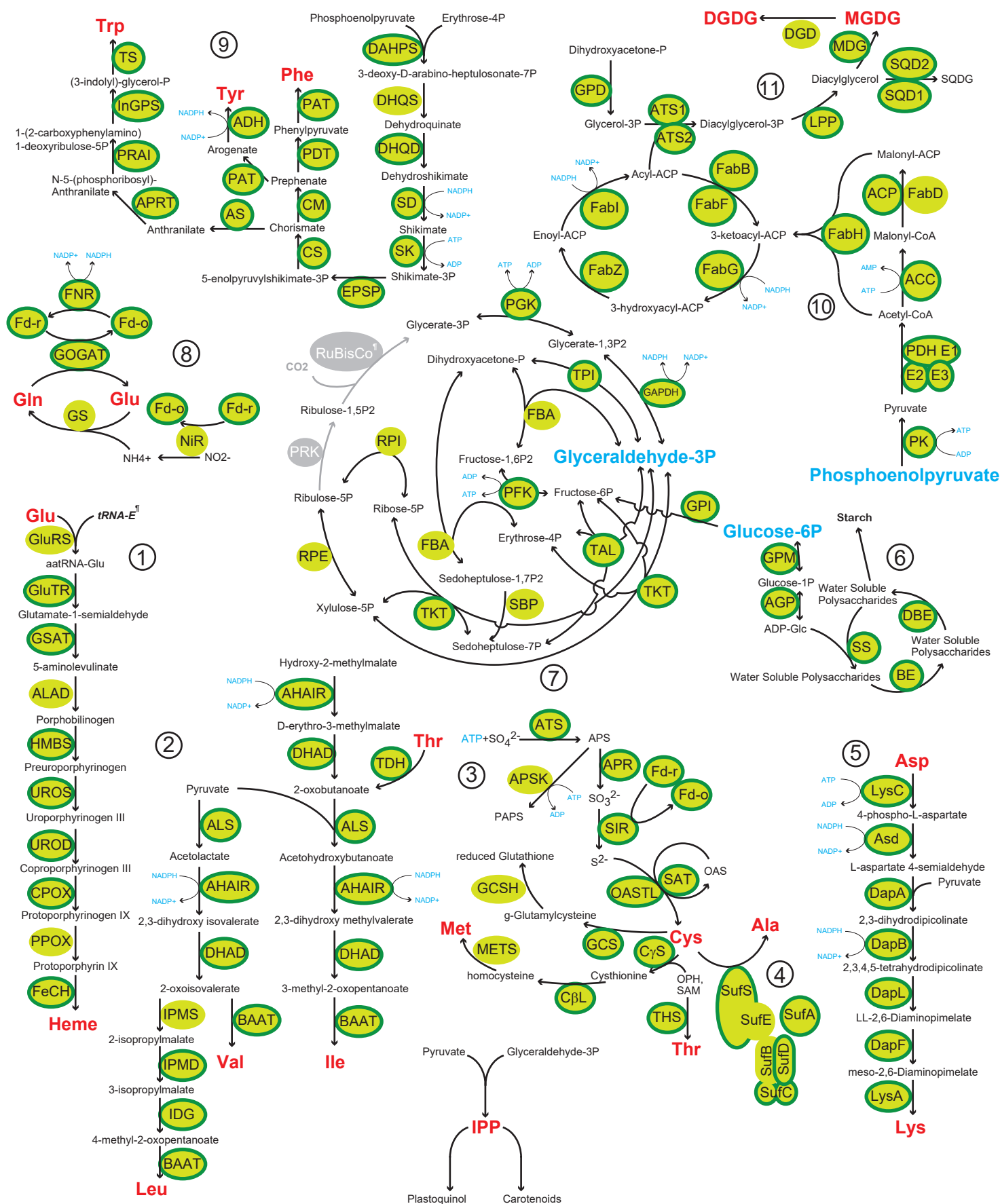

Supplement: Supplementary file 2 — Additional file 2. Figure S2. Predicted plastid metabolic map of chlamydomonad sp. NrCl902. 1. Heme synthesis, 2. branched chain amino acid synthesis, 3. sulfate assimilation, 4. Fe-S cluster synthesis, 5. aspartate-to-lysine conversion, 6. starch metabolism, 7. pentose phosphate pathway, 8. nitrite assimilation, 9. aromatic amino acid biosynthesis, 10. fatty acid synthesis, and 11. glycerolipid synthesis. Sequences in which plastid-targeting transit peptides were detected are shown by green circles with solid lines, while those lacking explicit plastid-targeting transit peptides are shown by green circle with no line. Details for isoprenoid (IPP), carotenoid, and plastoquinone syntheses are depicted in Fig. 3a. Substrate possibly imported from the cytosol and/or mitochondria are highlighted in blue, while those possibly exported to contribute to the cytosolic and mitochondrial functions are in red. Abbreviations of proteins are explained in Additional file 3 (Table S1). [file 12915_2020_853_MOESM2_ESM.pdf]

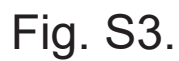

Fig. S3.

Supplement: Supplementary file 4 — Additional file 4. Figure S3. Interaction between the non-photosynthetic plastid and other compartments. Protein sequences with clear plastid-targeting sequences are shown by light green circles enclosed by solid lines. Protein sequences with clear mitochondrial targeting sequences are shown by orange circles enclosed by red lines. Protein sequences with neither targeting sequence are shown by light blue circles. “?”: Aconitase that catalyzes the conversion from citrate to isocitrate outside mitochondria was not detected in the transcriptome data of chlamydomonad sp. NrCl902. Translation initiation from the 2nd methionine of the mitochondrial aconitase gene might express Aconitase functioning outside mitochondria. Abbreviations are explained in Tables S1 and S2. [file 12915_2020_853_MOESM4_ESM.pdf]

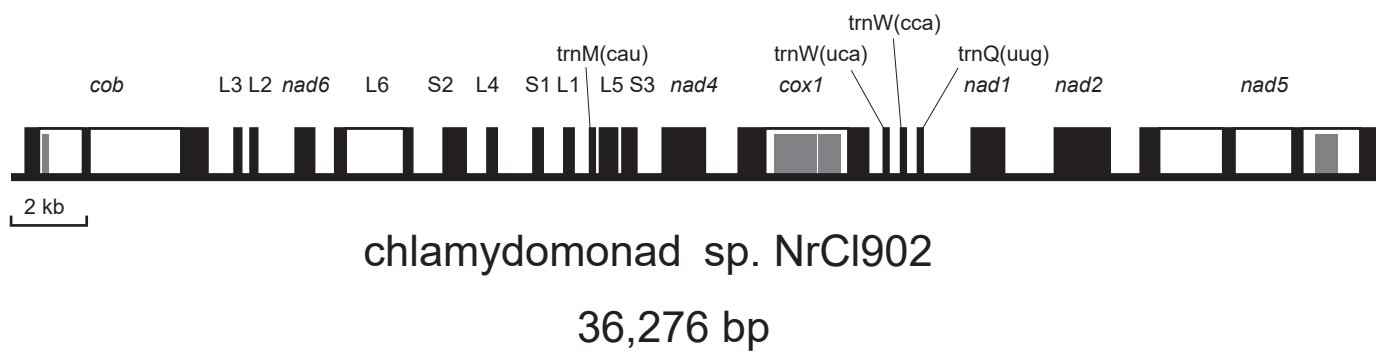

Fig. S4.

Supplement: Supplementary file 5 — Additional file 5. Figure S4. Mitochondrial genome of chlamydomonad sp. NrCl902. Conserved mitochondrial genes are shown by closed boxes, while intronic open reading frames are shown in gray. Intron regions are shown as open boxes. L1-L6 and S1-S3 show large subunit and small subunit rRNA gene fragments. Transfer RNA genes are shown by their amino acids and anticodons in parentheses. Given a variety of structures of mitochondrial genomes in Volvocales, i.e., circular genomes and tandem repeats of linear genome, it remains unclear whether the mitochondrial genome is a circularly mapping molecule or a linear, tandemly repeated molecule. [file 12915_2020_853_MOESM5_ESM.pdf]

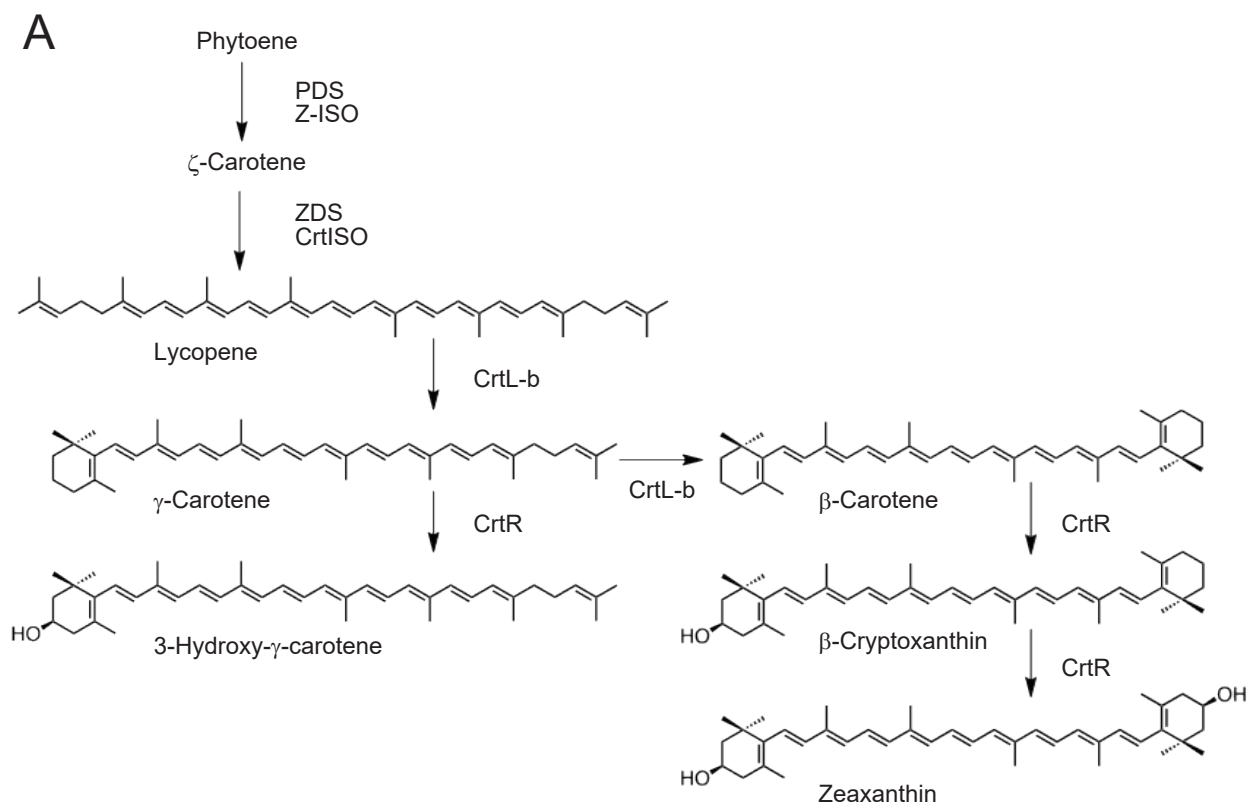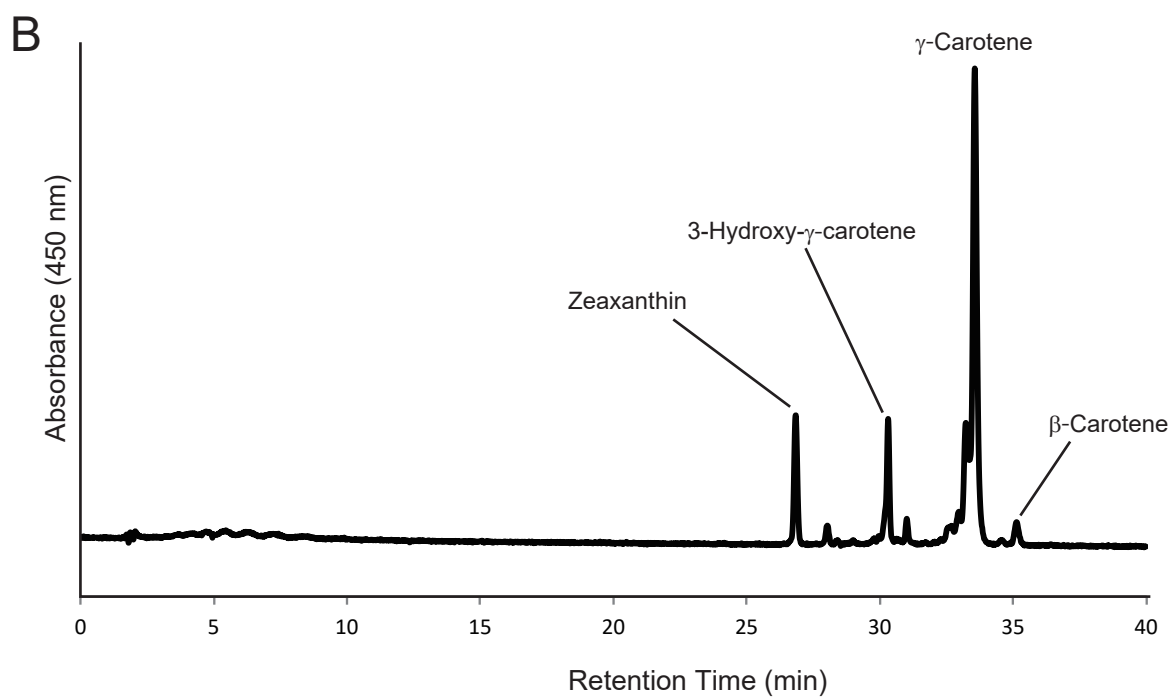

Figure S5

Supplement: Supplementary file 7 — Additional file 7. Figure S5. Carotenoid biosynthesis in chlamydomonad sp. NrCl902. A. The detailed pathway for carotenoid biosynthesis and structures of carotenoids predicted to be synthesized in this pathway. B. HPLC profile for carotenoid detection by absorbance at 450 nm in chlamydomonad sp. NrCl902. [file 12915_2020_853_MOESM7_ESM.pdf]

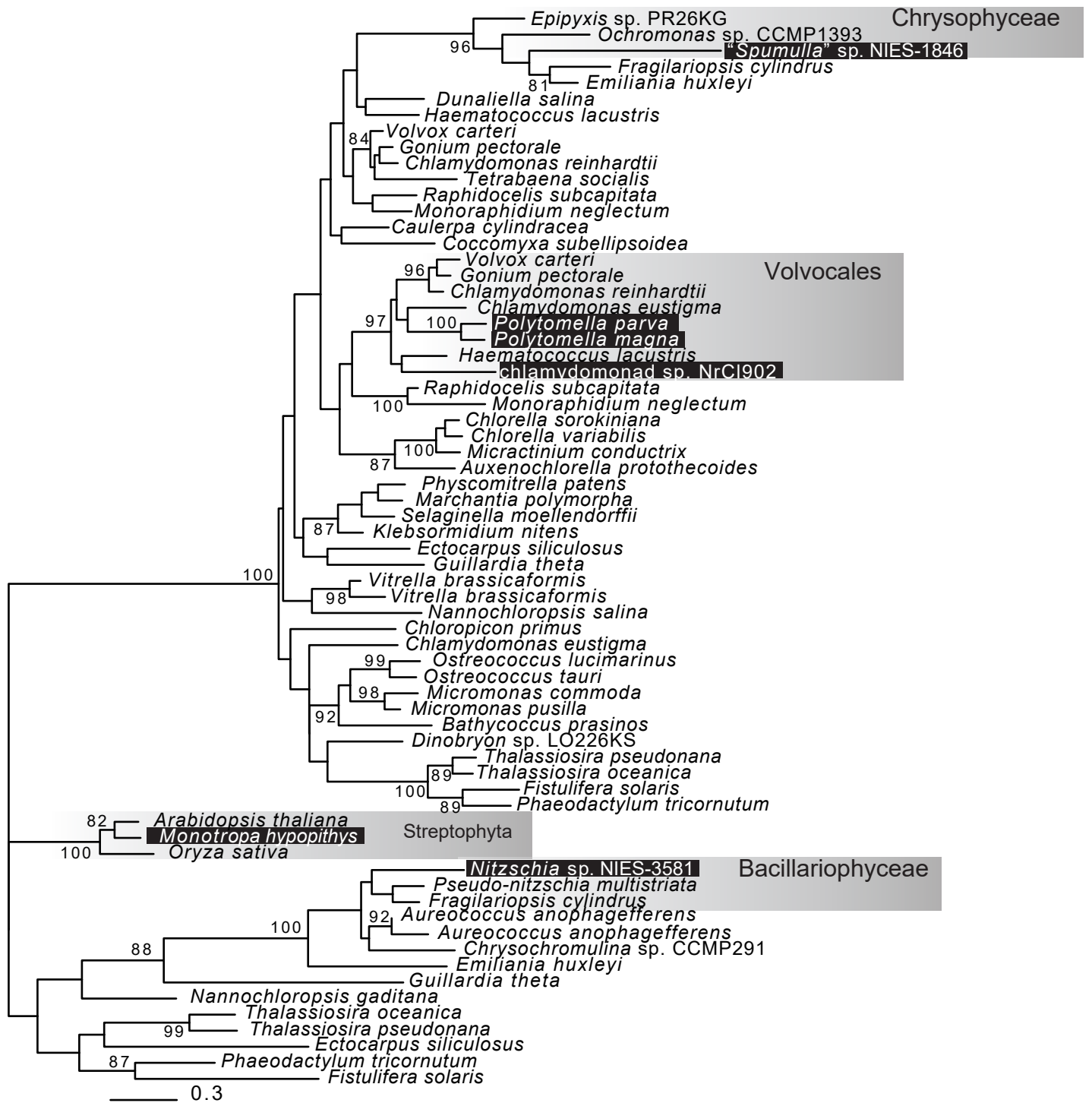

Fig. S6.

Supplement: Supplementary file 10 — Additional file 10. Figure S6. Maximum likelihood tree of plastid terminal oxidase in eukaryotes. Non-photosynthetic algae are highlighted in black. Numbers on branches are bootstrap values equal to or higher than 80%. The dataset comprised of 67 taxa and 236 sites was analyzed with IQtree under the LG + I + Γ model selected with Bayesian Information Criterion. [file 12915_2020_853_MOESM10_ESM.pdf]

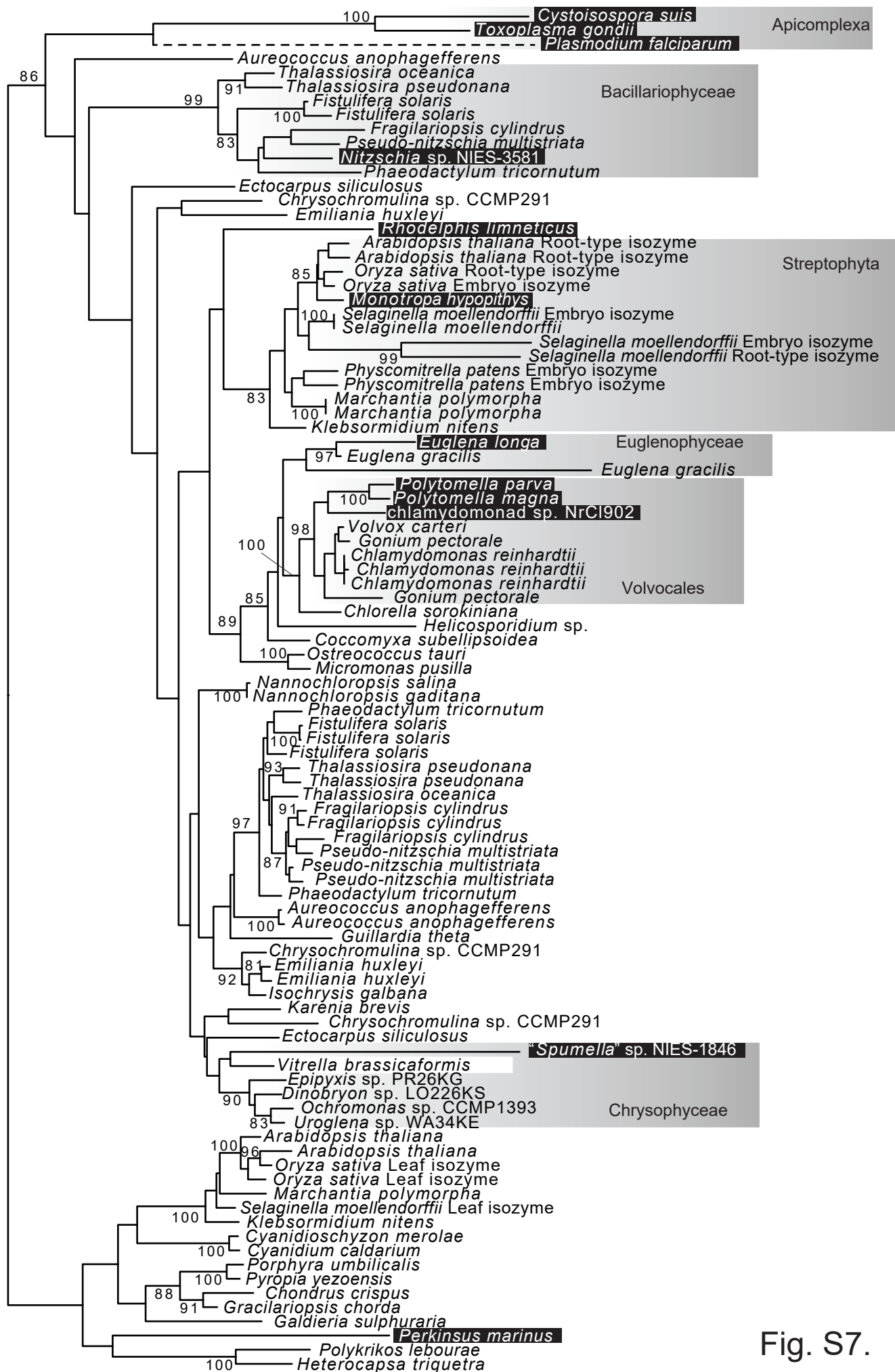

Fig. S7.

Supplement: Supplementary file 11 — Additional file 11. Figure S7. Maximum likelihood tree of Ferredoxin:NADP+ oxidoreductase in eukaryotes. Non-photosynthetic algae are highlighted in black. Numbers on branches are bootstrap values equal to or higher than 80%. The dataset comprised of 96 taxa and 303 sites was analyzed with IQtree under the LG + I + Γ model selected with Bayesian Information Criterion. [file 12915_2020_853_MOESM11_ESM.pdf]

A. Plant-type MMT

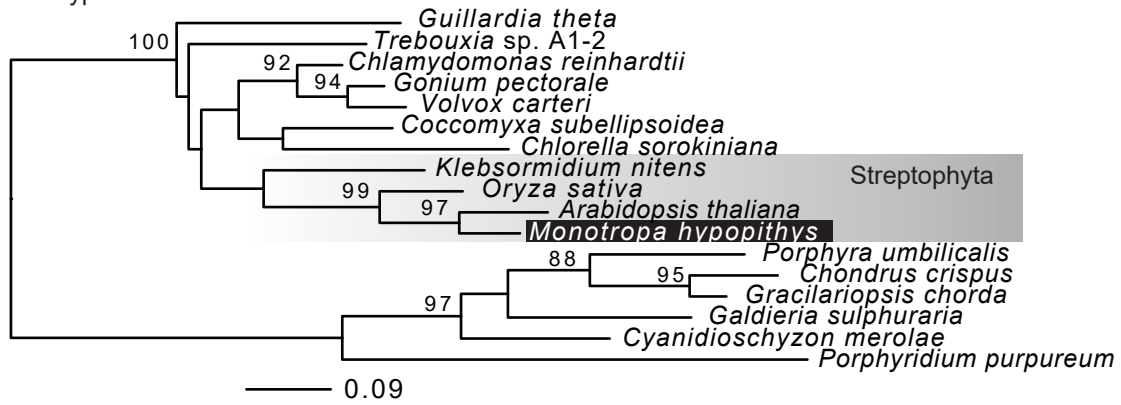

B. Divergent-type MMT

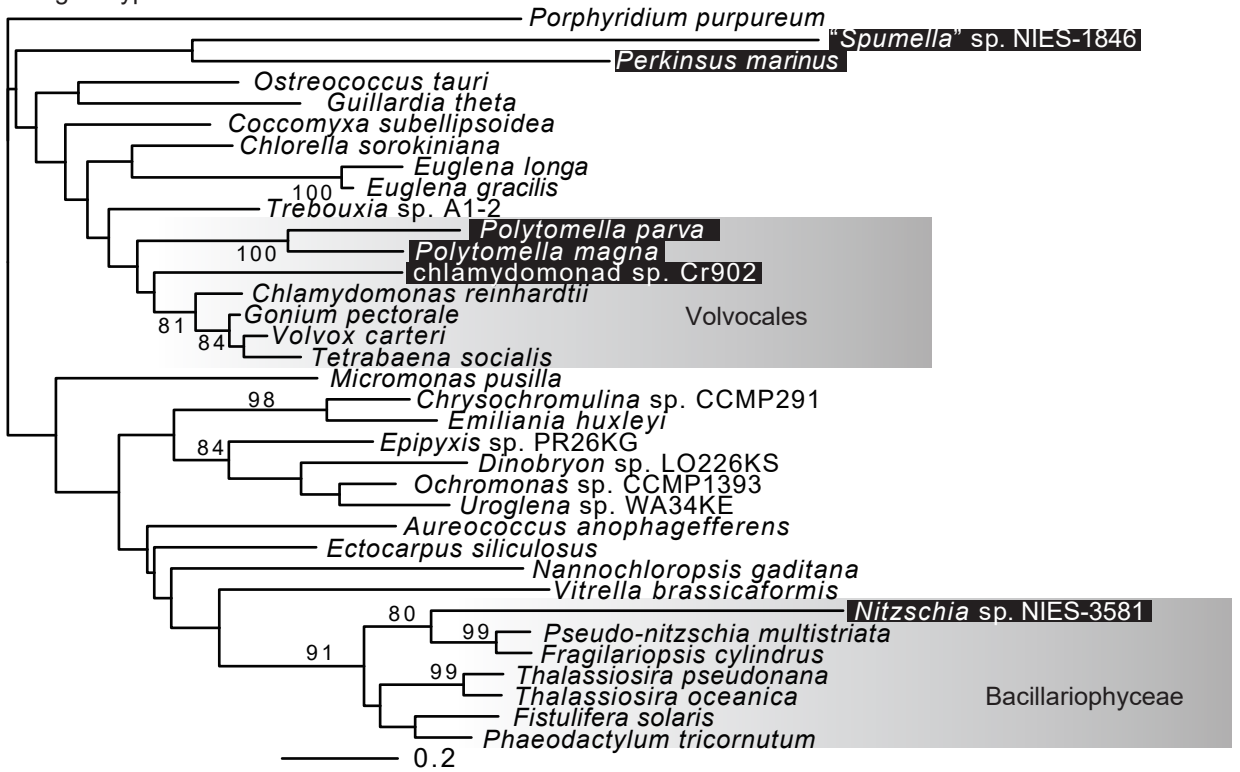

Fig. S8

Supplement: Supplementary file 12 — Additional file 12. Figure S8. Maximum likelihood tree of MPBQ/MSBQ methyltransferase in eukaryotes. A. Plant-type MMT. The dataset comprised of 17 taxa and 250 sites was analyzed with IQtree under the LG + Γ model selected with Bayesian Information Criterion. B. Divergent type MMT. The dataset comprised of 35 taxa and 292 sites was analyzed with IQtree under the LG + I + Γ model selected with Bayesian Information Criterion. Non-photosynthetic algae are highlighted in black. Numbers on branches are bootstrap values equal to or higher than 80%. [file 12915_2020_853_MOESM12_ESM.pdf]
