# Supplementary material for: A non-photosynthetic green alga illuminates the reductive evolution of plastid electron transport systems
Source: BMC Biol. 2020 Sep 16;18:126. doi: 10.1186/s12915-020-00853-w (PMC7495860; doi:10.1186/s12915-020-00853-w)
Supplement: Supplementary file 13 — Additional file 13. Datasets for phylogenetic analyses. [file 12915_2020_853_MOESM13_ESM.pdf]

>chlamydomonad sp. NrCl902 18S rRNA untrimmed sequence

GGGCTTGCCCGACTTTAGGCGAATCATGATAACTTCACGAATCGCACGGCCTCGTGCCGG
CGATGTTTCATTCAAATTTCTGCCCTATCAACTTTCGATGGTAGGATAGAGGCCTACCATG
GTGGTAACGGGTGACGGAGGATTAGGGTTCGATTCCGGAGAGGGTGCCTGAGAAACGG
CAACCACATCCAAGGAAGGCAGCAGGCGCGCAAATTACCCAATCCCAACACGGGGAGG
TAGTGACAATAAATAACAATACCGGGCATTTATGTCTGGTAATTGGAATGAGTACAATTTA
AATCCCTTAACGAGTACCAATTGGAGGGCAAGTCTGGTGCCAGCAGCCGCGGTAATTCC
AGCTCCAATAGCGTATATTTAAGTTGTTGCAGTTAAAAAGCTCGTAGTTGGATTTCGGGTG
GGTTCTAGCGGTCTGCCTCTGGTATGTACTGCTATGGCTCACCTTTCTGCCGGGGACGGG
CTCCTGGGCTTCACTGTCCGGGACTCGGAATCGGCGAGGTTACTTTGAGTAAATTAGAGT
GTTCAAAGCAAGCCTATGCTCTGAATATATTAGCATGGAATAACACGATAGGACTCTGGCC
TATCTTGTTGGTCTGTAGGACCGGAGTAATGATTAAGAGGGACAGTCGGGGGCATTCGTA
TTTCATTGTCAGAGGTGAAATTCTTGGATTTATGAAAGACGAACTTCTGCGAAAGCATTT
GCCAAGGATGTTTTCATTAATCAAGAACGAAAGTTGGGGGCTCGAAGACGATTAGATACC
GTCGTAGTCTCAACCATAAACGATGCCGACTAGGGATTGGCAGGTGTTTCGTTGATGACC
CTGCCAGCACCTTATGAGAAATCAAAGTTTTTGGGTTCCGGGGGGAGTATGGTCGCAAG
GCTGAAACTTAAAGGAATTGACGGAAGGGCACCACCAGGCGTGGAGCCTGCGGCTTAAT
TTGACTCAACACGGGAAAACTTACCAGGTCCAGACACGGGGAGGATTGACAGATTGAG
AGCTCTTTCTTGATTCTGTGGGTGGTGGTGCATGGCCGTTCTTAGTTGGTGGGTTGCCTTG
TCAGGTTGATTCCGGTAACGAACGAGACCTCAGCCTGCTAAATAGTCACGGGCACCTCG
GTGCACGCCTGACTTCTTAGAGGGACTATTGACGTTTAGTCAATGGAAGTGTGAGGCAAT
AACAGGTCTGTGATGCCCTTAGATGTTCTGGGCCGCACGCGCGCTACACTGATGCATTCA
ACGAGCCTATCCTTGGCCGAGAGGCCCGGGTAATCTTTGAAACTGCATCGTGATGGGGAT
AGATTATTGCAATTATTAGTCTTCAACGAGGAATGCCTAGTAAGCGCAAGTCATCAGCTTG
CGTTGATTACGTCCCTGCCCTTTGTACACACCGCCCGTCGCTCCTACCGATTGGGTGTGCT
GGTGAAGTGTTCGGATTGACCTTGGCTGATGGCAACATCGGCCTTGGTTGAGAAGATCAT
TGAACCCTCCCACCTAGAGGAAGGAGAAGTCGTAACAAGGTCTCCGTAGGTGAACCTGC
GGAGGGATCATTGAATCTATCAAT

18S rRNA dataset

>KR607489.1 Brachiomonas submarina strain SAG 7-2b -mating small subunit ribosomal RNA gene, partial sequence

ccatgcatgtctaagtatatatacggtgaaactgcgaatggctcattaaatcagttatagtttatttgatggtactttactcggataaccgtagtaattcta
gagctaatacgtgcgtaaatcccgacttctggaagggacgtatttattagataaaaggccagccgggcttgcccgacctatggcgaatcatgataa
cttcacgaatcgcacggccttgtgccggcgatgtttcattcaaatttctgccctatcaactttcgatggtaggatagaggcctaccatggtggtaacg
ggtgacggaggattagggttcgattccggagagggagcctgagaaacggctaccacatccaaggaaggcagcaggcgcgcaaattacccaat
cccgacacggggaggtagtgacaataaataacaataccgggcatgtctggtaattggaatgagtacaatttaaatcccttaacgagtatccattgg
agggcaagtctggtgccagcagccgcggtaattccagctccaatagcgtatatttaagttgttgcagttaaaaagctcgtagttggatttcgggcgg
gttccggcggtccgcctctggtgagcactgccgtggcccgcctttctgccggggaccgctcctgggcttaactgtccgggacgtggagtcggcg
atgttactttgagcaaaatagagtgttcaaagcaagcccgcgctctgaatacattagcatggaataacacgataggactctggcctatcttgttggtc
tgtaggaccggagtaatgattaagagggacagtcgggggcattcgtatttcattgtcagaggtgaaattcttggatttatgaaagacgaacttctgc
gaaagcatttgccaaggatgttttcattaatcaagaacgaaagttgggggctcgaagacgattagataccgtcgtagtctcaaccataaacgatgc
cgactagggattggcaggtgttttattgatgaccctgccagcaccttatgagaaatcaaagtttttgggttccgggggggagtatggtcgcaaggctg
aaacttaaaggaattgacggaagggcaccaccaggcgtggagcctgcggcttaatttgactcaacacgggaaaacttaccaggtccagacacg
ggaaggattgacagattgagagctctttcttgattctgtgggtggtggtgcatggccgttcttagttggtgggttgccttgtcaggttgattccggtaa
cgaacgagacctcagcctgctaaatagtcaagtgtactggtatacgtctgacttcttagagggactattggcgtttagccaatggaagtgtgaggca
ataacaggtctgtgatgcccttagatgttctgggccgcacgcgcgctacactgatgcattcaacgagcctatccttggccgagaggtccgggtaat
ctttgaaactgcatcgtgatggggatagattattgcaattattagtcttcaacgaggaatgcctagtaagcgcgagtcatcagctcgcgttgattacgt
ccctgccctttgtacacaccgcccgtcgctcctaccgattgggtgtgctggtgaagtgttcggattgacctcagccgtggaaccttggctgtggttg
agaagatcattaaaccctcccacctagaggaaggagaagtcgtaacaaggtctc---------------------------

>AF395437.1 Characiosiphon rivularis strain UTEX LB 1763 18S ribosomal RNA gene, partial sequence

ccatgcatgtctaagtatatatactgtgaaactgcgaatggctcattaaatcagttatagtttatttgatggtaccctactcggataaccgtagtaattcta
gagctaatacgtgcgtaaatcccgacttctggaagggacgtatttattagataaaaggccagccgggcttgcccgacctgcggtgaatcatgataa
cttcacgaatcgcacggcctcgtgccggcgatgtttcattcaaatttctgccctatcaactttcgatggtaggatagaggcctaccatggtggtaacg
ggtgacggaggattagggttcgattccggagagggagcctgagaaacggctaccacatccaaggaaggcagcaggcgcgcaaattacccaat
cccgacacggggaggtagtgacaataaataacaataccgggcatgtctggtaattggaatgagtacaatctaaatcccttaacgaggatccattgg
agggcaagtctggtgccagcagccgcggtaattccagctccaatagcgtatatttaagttgttgcagttaaaaagctcgtagttggatttcgggtgg
gttccagcggtccgcctctggtgtgcactgctgaggcctacctttctgccggggacggctcttgggcttaactgtccgggactcggaatcggcga
ggttactttgagtaaattagagtgttcaaagcaggcctacgctctgaatacattagcatggaataacacgataggactctggcctatcttgttggtctg
taggaccggagtaatgattaagagggacagtcgggggcattcgtatttcattgtcagaggtgaaattcttggatttatgaaagacgaacttctgcga
aagcatttgccaaggatgttttcattaatcaagaacgaaagttgggggctcgaagacgattagataccgtcgtagtctcaaccataaacgatgccg
actagggattggcaggtgttcctttgatgaccctgccagcaccttatgagaaatcaaagtttttgggttccgggggggagtatggtcgcaaggctgaa
acttaaaggaattgacggaagggcaccaccaggcgtggagcctgcggcttaatttgactcaacacgggaaaacttaccaggtccagacacggg
aaggattgacagattgagagctctttcttgattctgtgggtggtggtgcatggccgttcttagttggtgggttgccttgtcaggttgattccggtaacga

acgagacctcagcctgctaaatagtcactggcaccggtgcctgcttgacttcttagagggactattggcgtttagccagtggaagtgtgaggcaat
aacaggtctgtgatgcccttagatgttctgggccgcacgcgcgctacactgatgcattcaacgagcctatccttggccgagaggcccgggtaatct
ttgaaactgcatcgtgatggggatagattattgcaattattagtcttcaacgaggaatgcctagtaagcgcaagtcatcagcttgcgttgattacgtcc
ctgccctttgtacacaccgcccgtcgctcctaccgattgggtgtgctggtgaagtgtccggattggctttggctggtgaacatcgaccatggcgga
aaaggacattaaaccctcccaccta---

>AB360741.1 Characiochloris sasae gene for 18S ribosomal RNA, partial sequence, strain: NIES-567

ccatgcatgtctaagtatatatactgtgaaactgcgaatggctcattaaatcagttatagtttatttgatggtaccctactcggataaccgtagtaattcta
gagctaatacgtgcgtaaatcccgacttctggaagggacgtatttattagataaaaggccagccgggcttgcccgacctgcggtgaatcatgataa
cttcacgaatcgcacggcctcgcgccggcgatgtttcattcaaatttctgccctatcaactttcgatggtaggatagaggcctaccatggtggtaac
gggtgacggaggattagggttcgattccggagagggagcctgagaaacggctaccacatccaaggaaggcagcaggcgcgcaaattaccca
atcccgacacggggaggtagtgacaataaataacaataccgggcatgtctggtaattggaatgagtacaatctaaatcccttaacgaggatccatt
ggagggcaagtctggtgccagcagccgcggtaattccagctccaatagcgtatatttaagttgttgcagttaaaaagctcgtagttggatttcgggt
gggttccagcggtccgcctctggtgtgcactgctgaggcctacctttctgccggggacggctcttgggcttaactgtccgggactcggaatcggc
gaggttactttgagtaaattagagtgttcaaagcaggcctacgctctgaatacattagcatggaataacacgataggactctggcctatcttgttggtc
tgtaggaccggagtaatgattaagagggacagtcgggggcattcgtatttcattgtcagaggtgaaattcttggatttatgaaagacgaacttctgc
gaaagcatttgccaaggatgttttcattaatcaagaacgaaagttgggggctcgaagacgattagataccgtcgtagtctcaaccataaacgatgc
cgactagggattggcaggtgttcttttgatgaccctgccagcaccttatgagaaatcaaagtttttgggttccgggggagtatggtcgcaaggctg
aaacttaaaggaattgacggaagggcaccaccaggcgtggagcctgcggcttaatttgactcaacacgggaaaacttaccaggtccagacacg
ggaaggattgacagattgagagctctttcttgattctgtgggtggtggtgcatggccgttcttagttggtgggttgccttgtcaggttgattccggtaa
cgaacgagacctcagcctgctaaatagtcacgggcaccggtgcacgcttgacttcttagagggactattggcgtttagccagtggaagtgtgagg
caataacaggtctgtgatgcccttagatgttctgggccgcacgcgcgctacactgatgcattcaacgagcctatccttggccgagaggcccgggt
aatctttgaaactgcatcgtgatggggatagattattgcaattattagtcttcaacgaggaatgcctagtaagcgcaagtcatcagcttgcgttgatta
cgtccctgccctttgtacacaccgcccgtcgctcctaccgattgggtgtgctggtgaagtgtccggattggctttggttggtgaacatcgaccatgg
ccgaaaaggacattaaaccctcccacctagaggaaggagaagtcgtaacaaggtttcc--------------------------

>FR865616.1 Chlamydomonas applanata genomic DNA containing 18S rRNA gene, ITS1, 5.8S rRNA gene, ITS2, 28S rRNA gene, culture collection CCAP 11/9

ccatgcatgtctaagtatatatactgtgaaactgcgaatggctcattaaatcagttatagtttatttgatggtaccttactcggataaccgtagtaattcta
gagctaatacgtgcgtaaatcccgacttctggaagggacgtatttattagataaaaggccagccgggcttgcccgaccttaggcgaatcatgataa
cttcacgaatcgcacggccttgtgccggcgatgtttcattcaaatttctgccctatcaactttcgatggtaggatagaggcctaccatggtggtaacg
ggtgacggaggattagggttcgattccggagagggagcctgagaaacggctaccacatccaaggaaggcagcaggcgcgcaaattacccaat
cccgacacggggaggtagtgacaataaataacaataccgggcatgtctggtaattggaatgagtacaatctaaatcccttaacgagtatccattgg
agggcaagtctggtgccagcagccgcggtaattccagctccaatagcgtatatttaagttgttgcagttaaaaagctcgtagttggatttcgggtgg
gttctagcggtccgcctctggtgagtactgctcgggcctacctttctgccggggacggctcctgggcttcactgtccgggactcggaatcggcga
ggttactttgagtaaattagagtgttcaaagcaagcctacgctctgaatacattagcatggaataacacgataggactctggcctatcttgttggtctg
taggaccggagtaatgattaagagggacagtcgggggcattcgtatttcattgtcagaggtgaaattcttggatttatgaaagacgaacttctgcga
aagcatttgccaaggatgttttcattaatcaagaacgaaagttgggggctcgaagacgattagataccgtcgtagtctcaaccataaacgatgccg

actagggattggcaggtgtttcgttgatgaccctgccagcaccttatgagaaatcaaagtttttgggttccggggggagtatggtcgcaaggctga
aacttaaaggaattgacggaagggcaccaccaggcgtggagcctgcggcttaatttgactcaacacgggaaaacttaccaggtccagacacgg
ggaggattgacagattgagagctctttcttgattctgtgggtggtggtgcatggccgttcttagttggtgggttgccttgtcaggttgattccggtaac
gaacgagacctcagcctgctaaatagtcacgtccaccggtggatgcctgacttcttagagggactattggcgtttagccaatggaagtgtgaggc
gataacaggtctgtgatgcccttagatgttctgggccgcacgcgcgctacactgacgcattcaacgagcctatccttggccgagaggtccgggta
atctttgaaactgcgtcgtgatggggatagattattgcaattattagtcttcaacgaggaatgcctagtaagcgcaagtcatcagcttgcgttgattac
gtccctgccctttgtacacaccgcccgtcgctcctaccgattgggtgtgctggtgaagtgttcggattgacttcagcgggggcaactctgctgttgtt
gagaagatcattaaaccctcccacctagaggaaggagaagtcgtaacaaggtttccgtaggtgaacctgcggaaggatcattg
>AJ410442.1 Chlorogonium capillatum partial 18S rRNA gene, strain SAG 12-2e
ccatgcatgtctaagtatatatacggtgaaactgcgaatggctcattaaatcagttatagtttatttgatggtaccttactcggataaccgtagtaattct
agagctaatacgtgcgtaaaccccgacttctggaaggggcgtatttattagataaaaggccagccgggcttgcccgactcttggcgaatcatgata
acttcacgaatcgcacggcctcgtgccggcgatgtttcattcaaatttctgccctatcaactttcgatggtaggatagaggcctaccatggtggtaac
gggtgacggaggattagggttcgattccggagagggagcctgagaaacggctaccacatccaaggaaggcagcaggcgcgcaaattaccca
atcccgacacggggaggtagtgacaataaataacaataccgggcttgtctggtaattggaatgagtacaatttaaatcccttaacgagtatccattg
gagggcaagtctggtgccagcagccgcggtaattccagctccaatagcgtatatttaagttgttgcagttaaaaagctcgtagttggatttcgggtg
ggttccagcggtctgcctctggtatgtactgctgtggctcacctttctgccggggacggctcctgggcttcactgtccgggactcggaatcggcga
ggttactttgagtaaaatagagtgttcaaagcaagcctacgctctgaatacattagcatggaataacacgataggactctggcctatcttgttggtctg
taggaccggagtaatgattaagagggacagtcgggggcattcgtatttcattgtcagaggtgaaattcttggatttatgaaagacgaacttctgcga
aagcatttgccaaggatgttttcattaatcaagaacgaaagttgggggctcgaagacgattagataccgtcgtagtctcaaccataaacgatgccg
actagggattggcaggtgttttattgatgaccctgccagcaccttatgagaaatcaaagtttttgggttccggggggagtatggtcgcaaggctgaa
acttaaaggaattgacggaagggcaccaccaggcgtggagcctgcggcttaatttgactcaacacgggaaaacttaccaggtccagacacggg
aaggattgacagattgagagctctttcttgattctgtgggtggtggtgcatggccgttcttagttggtgggttgccttgtcaggttgattccggtaacga
acgagacctcagcctgctaaatagtcaagcgtaccggtacgcgcctgacttcttagagggactattggcgtttagccaatggaagtgtgaggcga
taacaggtctgtgatgcccttagatgttctgggccgcacgcgcgctacactgatgcattcaacgagcctatccttggccgagaggtccgggtaatc
tttgaatctgcatcgtgatggggatagattattgcaattattagtcttcaacgaggaatgcctagtaagcgcaagtcatcagcttgcgttgattacgtcc
ctgccctttgtacacaccgcccgtcgctcctaccgattgggtgtgctggtgaagtgttcggattggcttcagcagttgaacttctgctgttgctgaga
agatcattaaaccctcccacctagaggaaggagaagtcgtaacaaggtttccgtaggtgaacctgcggaaggatcattg
>AB477055.1 Chlorogonium complexum gene for 18S rRNA, partial sequence, strain: NIES-2296
ccatgcatgtctaagtatatatacggtgaaactgcgaatggctcattaaatcagttatagtttatttgatggtaccttactcggataaccgtagtaattct
agagctaatacgtgcgtaaaccccgacttctggaaggggcgtatttattagataaaaggccagccgggcttgcccgactctaggcgaatcatgat
aacttcacgaatcgcacggccttgtgccggcgatgtttcattcaaatttctgccctatcaactttcgatggtaggatagaggcctaccatggtggtaa
cgggtgacggaggattagggttcgattccggagagggagcctgagaaacggctaccacatccaaggaaggcagcaggcgcgcaaattaccc
aatcccgacacggggaggtagtgacaataaataacaataccgggcttgtctggtaattggaatgagtacaatttaaatcccttaacgagtatccatt
ggagggcaagtctggtgccagcagccgcggtaattccagctccaatagcgtatatttaagttgttgcagttaaaaagctcgtagttggatttcgggt
gggttccagcggtctgcctctggtatgtactgctgtggctcacctttctgccggggacggctcctgggcttaactgtccgggactcggaatcggcg
aggttactttgagtaaaatagagtgttcaaagcaagcctacgctctgaatacattagcatggaataacacgataggactctggcctatcttgttggtct

gtaggaccggagtaatgattaagagggacagtcgggggcattcgtatttcattgtcagaggtgaaattcttggatttatgaaagacgaacttctgcg
aaagcatttgccaaggatgttttcattaatcaagaacgaaagttgggggctcgaagacgattagataccgtcgtagtctcaaccataaacgatgcc
gactagggattggcaggtgttttattgatgaccctgccagcaccttatgagaaatcaaagtttttgggttccgggggggagtatggtcgcaaggctga
aacttaaaggaattgacggaagggcaccaccaggcgtggagcctgcggcttaatttgactcaacacgggaaaacttaccaggtccagacacgg
gaaggattgacagattgagagctctttcttgattctgtgggtggtggtgcatggccgttcttagttggtgggttgccttgtcaggttgattccggtaac
gaacgagacctcagcctgctaaatagtcaagcgtaccggtatgcgtctgacttcttagagggactattggcgtttagccaatggaagtgtgaggcg
ataacaggtctgtgatgcccttagatgttctgggccgcacgcgcgctacactgatgcattcaacgagcctatccttggccgagaggtccgggtaat
ctttgaatctgcatcgtgatggggatagattattgcaattattagtcttcaacgaggaatgcctagtaagcgcaagtcatcagcttgcgttgattacgtc
cctgccctttgtacacaccgcccgtcgctcctaccgattgggtgtgctggtgaagtgttcggattggcttcagcagttgaacttctgctgttgctgag
aagatcattaaaccctcccaccta---

>AJ410444.1 Chlorogonium elongatum partial 18S rRNA gene, strain UTEX 2571

ccatgcatgtctaagtatatatacggtgaaactgcgaatggctcattaaatcagttatagtttatttgatggtaccttactcggataaccgtagtaattct
agagctaatacgtgcgtaaaccccgacttctggaaggggcgtatttattagataaaaggccagccgggcttgcccgactctaggcgaatcatgat
aacttcacgaatcgcacggccttgtgccggcgatgtttcattcaaatttctgccctatcaactttcgatggtaggatagaggcctaccatggtggtaa
cgggtgacggaggattagggttcgattccggagagggagcctgagaaacggctaccacatccaaggaaggcagcaggcgcgcaaattaccc
aatcccgacacggggaggtagtgacaataaataacaataccgggcttgtctggtaattggaatgagtacaatttaaaccccttaacgagtatccatt
ggagggcaagtctggtgccagcagccgcggtaattccagctccaatagcgtatatttaagttgttgcagttaaaaagctcgtagttggatttcgggt
gggttccagcggtctgcctctggtatgtactgctgtggctcacctttctgccggggacggctcctgggcttaactgtccgggactcggaatcggcg
aggttactttgagtaaaatagagtgttcaaagcaagcctacgctctgaatacgttagcatggaataacacgataggactctggcctatcttgttggtct
gtaggaccggagtaatgattaagagggacagtcgggggcattcgtatttcattgtcagaggtgaaattcttggatttatgaaagacgaacttctgcg
aaagcatttgccaaggatgttttcattaatcaagaacgaaagttgggggctcgaagacgattagataccgtcgtagtctcaaccataaacgatgcc
gactagggattggcaggtgttttattgatgaccctgccagcaccttatgagaaatcaaagtttttgggttccgggggggagtatggtcgcaaggctga
aacttaaaggaattgacggaagggcaccaccaggcgtggagcctgcggcttaatttgactcaacacgggaaaacttaccaggtccagacacgg
gaaggattgacagattgagagctctttcttgattctgtgggtggtggtgcatggccgttcttagttggtgggttgccttgtcaggttgattccggtaac
gaacgagacctcagcctgctaaatagtcaagcgtaccggtatgcgtctgacttcttagagggactattggcgtttagccaatggaagtgtgaggcg
ataacaggtctgtgatgcccttagatgttctgggccgcacgcgcgctacactgatgcattcaacgagcctatccttggccgagaggtccgggtaat
ctttgaatctgcatcgtgatggggatagattattgcaattattagtcttcaacgaggaatgcctagtaagcgcaagtcatcagcttgcgttgattacgtc
cctgccctttgtacacaccgcccgtcgctcctaccgattgggtgtgctggtgaagtgttcggattgactccagcagttgaacttctgctgttgttgaga
agatcattaaaccctcccacctagaggaaggagaagtcgtaacaaggtttccgtaggtgaacctgcggaaggatcattg

>KY086471.1 Chlorosarcinopsis eremi strain ACSSI 132 small subunit ribosomal RNA gene, partial sequence

ccatgcatgtctaagtatatatactgtgaaactgcgaatggctcattaaatcagttatagtttatttgatggtactttactcggataaccgtagtaattcta
gagctaatacgtgcgtaaatcccgacttctggaagggacgtatttattagataaaaggccagccgggcttgcccgactcttggcgaatcatgataa
cttcacgaatcgcacggccttgtgccggcgatgtttcattcaaatttctgccctatcaactttcgatggtaggatagaggcctaccatggtggtaacg
ggtgacggaggattagggttcgattccggagagggagcctgagaaacggctaccacatccaaggaaggcagcaggcgcgcaaattacccaat
cccgacacggggaggtagtgacaataaataacaataccgggcc-

gtctggtaattggaatgagtacaatctaaatcccttaacgagtatccattggagggcaagtctggtgccagcagccgcggtaattccagctccaata
gcgtatatttaagttgttgcagttaaaaagctcgtagttggatttcgggtgggtgtcgccggtctgcctctggtatgtactggcgctgctcacctttctg
ccggggacggctcctgggcttaactgtctgggactcggagtcggcgttgttactttgagtaaattagagtgttcaaagcaagccttcgctctgaata
cattagcatggaataacacgataggactctggcctatcttgttggtctgtaggaccggagtaatgattaagagggacagtcgggggcattcgtattt
cattgtcagaggtgaaattcttggatttatgaaagacgaacttctgcgaaagcatttgccaaggatgttttcattgatcaagaacgaaagttgggggc
tcgaagacgattagataccgtcgtagtctcaaccataaacgatgccgactagggattggcaggtgtttcattgatgaccctgccagcaccttatgag
aaatcaaagtttttgggttccggggggagtatggtcgcaaggctgaaacttaaaggaattgacggaagggcaccaccaggcgtggagcctgcg
gcttaatttgactcaacacggggaaacttaccaggtccagacacggggaggattgacagattgagagctctttcttgattctgtgggtggtggtgca
tggccgttcttagttggtgggttgccttgtcaggttgattccggtaacgaacgagacctccgcctgctaaatagtcacgcgcaccggtgcacgcct
gacttcttagagggactaccggcgattagtcgttggaagtgggaggcaataacaggtctgtgatgcccttagatgttctgggccgcacgcgcgct
acactgatgcattcaacgagcctatccttggccgagaggtccgggtaatctttgaaactgcatcgtgatggggatagattattgcaattattagtcttc
aacgaggaatgcctagtaagcgcatgtcatcagcatgcgttgattacgtccctgccctttgtacacaccgcccgtcgctcctaccgattgggtgtgc
tggtgaagtgttcggactggcttcaatgggggaacccctgctgttgccgggaagaacattaaaccctcccacctagaggaaggagaagtcgtaa
caaggtctccgtaggtgaacctg--------------
>AJ410443.1 Chlorogonium euchlorum partial 18S rRNA gene, strain SAG 12-2d
ccatgcatgtctaagtatatatacggtgaaactgcgaatggctcattaaatcagttatagtttatttgatggtaccttactcggataaccgtagtaattct
agagctaatacgtgcgtaaaccccgacttctggaaggggcgtatttattagataaaaggccagccgggcttgcccgactctaggcgaatcatgat
aacttcacgaatcgcacggccttgtgccggcgatgtttcattcaaatttctgccctatcaactttcgatggtaggatagaggcctaccatggtggtaa
cgggtgacggaggattagggttcgattccggagagggagcctgagaaacggctaccacatccaaggaaggcagcaggcgcgcaaattaccc
aatcccgacacggggaggtagtgacaataaataacaataccgggcttgtctggtaattggaatgagtacaatttaaatcccttaacgagtatccatt
ggagggcaagtctggtgccagcagccgcggtaattccagctccaatagcgtatatttaagttgttgcagttaaaaagctcgtagttggatttcgggt
gggttccagcggtctgcctctggtatgtactgctgtggctcacctttctgccggggacggctcctgggcttcactgtccgggactcggaatcggcg
aggttactttgagtaaaatagagtgttcaaagcaagcctacgctctgaatacattagcatggaataacacgataggactctggcctatcttgttggtct
gtaggaccggagtaatgattaagagggacagtcgggggcattcgtatttcattgtcagaggtgaaattcttggatttatgaaagacgaacttctgcg
aaagcatttgccaaggatgttttcattaatcaagaacgaaagttgggggctcgaagacgattagataccgtcgtagtctcaaccataaacgatgcc
gactagggattggcaggtgttttattgatgaccctgccagcaccttatgagaaatcaaagtttttgggttccggggggagtatggtcgcaaggctga
aacttaaaggaattgacggaagggcaccaccaggcgtggagcctgcggcttaatttgactcaacacgggaaaacttaccaggtccagacacgg
gaaggattgacagattgagagctctttcttgattctgtgggtggtggtgcatggccgttcttagttggtgggttgccttgtcaggttgattccggtaac
gaacgagacctcagcctgctaaatagtcaagcgtaccggtacgcgcctgacttcttagagggactattggcgtttagccaatggaagtgtgaggc
gataacaggtctgtgatgcccttagatgttctgggccgcacgcgcgctacactgatgcattcaacgagcctatccttggccgagaggtccgggta
atctttgaatctgcatcgtgatggggatagattattgcaattattagtcttcaacgaggaatgcctagtaagcgcaagtcatcagcttgcgttgattacg
tccctgccctttgtacacaccgcccgtcgctcctaccgattgggtgtgctggtgaagtgttcggattggctccagcagttgaacttctgctgttgctga
gaagatcattaaaccctcccacctagaggaaggagaagtcgtaacaaggtttccgtaggtgaacctgcggaaggatcattg
>KJ635670.1 Chlamydomonas gloeophila strain UTEX_608 18S ribosomal RNA gene, partial sequence
ccatgcatgtctaagtatatatacggtgaaactgcgaatggctcattaaatcagttatagtttatttgatggtaccttactcggataaccgtagtaattct

agagctaatacgtgcgtaaaccccgacttctggaaggggcgtatttattagataaaaggccagccgggcttgcccgacctttggcgaatcatgata
acttcacgaatcgcacggcctcgtgccggcgatgtttcattcaaatttctgccctatcaactttcgatggtaggatagaggcctaccatggtggtaac
gggtgacggaggattagggttcgattccggagagggagcctgagaaacggctaccacatccaaggaaggcagcaggcgcgcaaattaccca
atcccgacacggggaggtagtgacaataaataacaataccgggcatgtctggtaattggaatgagtacaatctaaaccccttaacgagtatccatt
ggagggcaagtctggtgccagcagccgcggtaattccagctccaatagcgtatatttaagttgttgcagttaaaaagctcgtagttggatttcgggt
gggttccagcggtctgcctctggtatgtactgctgtggctcacctttctgccggggacggctcctgggctttactgtccgggactcggaatcggcgt
ggttactttgagtaaattagagtgttcaaagcaagcctacgctctgaatacattagcatggaataacacgataggactctggcctatcttgttggtctg
taggaccggagtaatgattaagagggacagtcgggggcattcgtatttcattgtcagaggtgaaattcttggatttatgaaagacgaacttctgcga
aagcatttgccaaggatgttttcattaatcaagaacgaaagttgggggctcgaagacgattagataccgtcgtagtctcaaccataaacgatgccg
actagggattggcaggtgttttattgatgaccctgccagcaccttatgagaaatcaaagtttttgggttccgggggggagtatggtcgcaaggctgaa
acttaaaggaattgacggaagggcaccaccaggcgtggagcctgcggcttaatttgactcaacacgggaaaacttaccaggtccagacacggg
aaggattgacagattgagagctctttcttgattctgtgggtggtggtgcatggccgttcttagttggtgggttgccttgtcaggttgattccggtaacga
acgagacctcagcctgctaaatagtcaagagtaccggtattcgcctgacttcttagagggactattggcgtttagccaatggaagtgtgaggcaat
aacaggtctgtgatgcccttagatgttctgggccgcacgcgcgctacactgatgcattcaacgagcctacccttggccgagaggtccgggtaatct
tcgaaactgcatcgtgatggggatagattattgcaattattagtcttcaacgaggaatgcctagtaagcgcaagtcatcagcttgcgttgattacgtcc
ctgccctttgtacacaccgcccgtcgctcctaccgattgggtgtgctggtgaagtgttcggattgactgcagcggtggaacctctgctgctgttgag
aagatcattaaaccctcccacctagaggaaggagaagtcgtaacaaggtttccgtag----------------------
>KM020099.1 Chlorococcum minutum strain SAG 213-7 18S ribosomal RNA gene, partial sequence
ccatgcatgtctaagtatatatacggtgaaactgcgaatggctcattaaatcagttatagtttatttgatggtaccctacttggataaccgtaggaaatct
agagctaatacatgcgtaaatcccgacttctggaagggacgtatttattagataaaaggccagccgggcttgcccgactttaggcgaatcatgata
acttcacgaatcgcacggcctcgtgccggcgatgtttcattcaaatttctgccctatcaactttcgatggtaggatagaggcctaccatggtggtaac
gggtgacggaggattagggttcgattccggagagggagcctgagaaacggcttccacatccaaggaaggcagcaggcgcgcaaattacccaa
tcccgacacggggaggtagtgacaataaataacaatactgggcatgtctggtaattggaatgagcacaatgtaaatatcttaacgagtatccattgg
agggcaagtctggtgccagcagccgcggtaattccagctccaatagcgtatatttaagttgttgcagttaaaaagctcgtagttggatttcgggtga
gtcgacgcggtctgcctctggtatgtactgcctcggttcacctttctgctggggacggctcctgggcttaactgcttgggacctggaatcagcgaag
tgaccttgagcaaacaagagtgttcaaagcaagccaacgctctgaattttttagcatggaatcacacgataggactctggcctatcttgttggtctgt
aggaccggagtaatgattaagagggacagtcgggggcattcgtatttcattgtcagaggtgaaattcttggatttatgaaagacgaacttctgcgaa
agcatttgccaaggatgttttcattgatcaagaacgaaagttgggggctcgaagacgattagataccgtcgtagtctcaaccataaacgatgccga
ctagggattggcaggtgttccattgatgaccctgccagcaccttatgagaaatcaaagtttttgggttccgggggggagtatggtcgcaaggctgaa
acttaaaggaattgacggaagggcaccaccaggcgtggagcctgcggcttaatttgactcaacacggggaaacttaccaggtccagacacggg
gaggattgacagattgagagctctttcttgattctgtgggtggtggtgcatggccgttcttagttggtgggttgccttgtcaggttgattccggtaacg
aacgagacctcagcctgctaaatagtcacgggtaccggtacacgcctgacttcttagagggactattggcgtctagtcaatggaagtgtgaggca
ataacaggtctgtgatgcccttagatgttctgggccgcacgcgcgctacactgatgcattcaacgagcctatccttggccgagaggtccgggtaat
ctttgaaactgcatcgtgatggggatagattattgcaattattagtcttcaacgaggaatgcctagtaagcgcaagtcatcagcttgcgttgattacgt
ccctgccctttgtacacaccgcccgtcgctcctaccgattgggtgtgctggtgaagtgttcggattggcttcaacgcaggaacctctgttgtggccg
agaagaacattaaaccctcccacctagaggaaggagaagtcgtaacaaggtttccgtaggtgaacctgcagaaggatca---

>AB602849.1 Chlamydomonas pseudoneoplanoconvexa gene for 18S rRNA, partial sequence
ccatgcatgtctaagtatatatactgtgaaactgcgaatggctcattaaatcagttatagtttatttgatggtaccttactcggataaccgtagtaattcta
gagctaatacgtgcgtaaatcccgacttctggaagggacgtatttattagataaaaggccagccgggcttgcccgacctcaggcgaatcatgata
acttcacgaatcgcatggcctcgtgccggcgatgtttcattcaaatttctgccctatcaactttcgatggtaggatagaggcctaccatggtggtaac
gggtgacggaggattagggttcgattccggagagggagcctgagaaacggctaccacatccaaggaaggcagcaggcgcgcaaattaccca
atcccgacacggggaggtagtgacaataaataacaataccgggcatgtctggtaattggaatgagtacaatttaaatcccttaacgagtaccaattg
gagggcaagtctggtgccagcagccgcggtaattccagctccaatagcgtatatttaagttgttgcagttaaaaagctcgtagttggatttcgggtg
ggttctagcggtctgcctctggtatgtactgctatggctcacctttctggcgggaaccgctcctgggcttcactgtccgggacgtggtatcgccgaa
gttactgtgagtaaaatagagtgttcaaagcaagcctatgctctgaatatattagcatggaataacacgataggactctggcctatcttgttggtctgt
aggaccggagtaatgattaagagggacagtcgggggcattcgtatttcattgtcagaggtgaaattcttggatttatgaaagacgaactactgcga
aagcatttgccaaggatgttttcattaatcaagaacgaaagttgggggctcgaagacgattagataccgtcgtagtctcaaccataaacgatgccg
actagggattggcaggtgtttcgttgatgaccctgccagcaccttatgagaaatcaaagtttttgggttccgggggggagtatggtcgcaaggctga
aacttaaaggaattgacggaagggcaccaccaggcgtggagcctgcggcttaatttgactcaacacggggaaacttaccaggtccagacacgg
ggaggattgacagattgagagctctttcttgattctgtgggtggtggtgcatggccgttcttagttggtgggttgccttgtcaggttgattccggtaac
gaacgagacctcagcctgctaaatagtcacgggcaccggtgcacgcctgacttcttagagggactattggcgtttagccaatggaagtgtgaggc
aataacaggtctgtgatgcccttagatgttctgggccgcacgcgcgctacactgatgcattcaacgagcctatccttcgccgagaggcgcgggta
atctttgaaactgcatcgtgatggggatagattattgcaattattagtcttcaacgaggaatgcctagtaagcgcgagtcatcagctcgcgttgattac
gtccctgccctttgtacacaccgcccgtcgctcctaccgattgggtgtgctggtgaagttttcggattgaccttggttgtggaacctcgaccttggttg
agaagaaaattaaaccctcccacctagaggaaggagaagtcgtaacaaggtctcc--------------------------
>FR865585.1 Chloromonas perforata genomic DNA containing 18S rRNA gene, ITS1, 5.8S rRNA gene, ITS2, 28S rRNA gene, culture collection CCAP 11/43
ccatgcatgtctaagtatatatactgtgaaactgcgaatggctcattaaatcagttatagtttatttgatggtaccctactcggataaccgtagtaattcta
gagctaatacgtgcgtaaatcccgacttctggaagggacgtatttattagataaaaggccagccgagcttgctcgacctgcggtgaatcatgataa
cttcacgaatcgcacggcctcgcgccggcgatgtttcattcaaatttctgccctatcaactttcgatggtaggatagaggcctaccatggtggtaac
gggtgacggaggattagggttcgattccggagagggagcctgagaaacggctaccacatccaaggaaggcagcaggcgcgcaaattaccca
atcccgacacggggaggtagtgacaataaataacaataccgggcatgtctggtaattggaatgagtacaatctaaatcccttaacgaggatccatt
ggagggcaagtctggtgccagcagccgcggtaattccagctccaatagcgtatatttaagttgttgcagttaaaaagctcgtagttggatttcgggt
gggttctagcggtccgcctctggtgagtactgctatggcctacctttctgccggggacggctcttggacttcactgtccgggactcggaatcggcg
aggttactttgagtaaattagagtgttcaaagcaggcctacgctctgaatacattagcatggaataacacgataggactctggcctatcttgttggtct
gtaggaccggagtaatgattaagagggacagtcgggggcattcgtatttcattgtcagaggtgaaattcttggatttatgaaagacgaacttctgcg
aaagcatttgccaaggatgttttcattaatcaagaacgaaagttgggggctcgaagacgattagataccgtcgtagtctcaaccataaacgatgcc
gactagggattggcaggtgtttcattgatgaccctgccagcaccttatgagaaatcaaagtttttgggttccgggggggagtatggtcgcaaggctg
aaacttaaaggaattgacggaagggcaccaccaggcgtggagcctgcggcttaatttgactcaacacgggaaaacttaccaggtccagacacg
gggaggattgacagattgagagctctttcttgattctgtgggtggtggtgcatggccgttcttagttggtgggttgccttgtcaggttgattccggtaa
cgaacgagacctcagcctgctaaatagtcacggctaccggtagtcgcctgacttcttagagggactattggcgtttagccaatggaagtgtgagg
caataacaggtctgtgatgcccttagatgttctgggccgcacgcgcgctacactgatgcattcaacgagcctatccttggccgagaggcccgggt

aatctttgaaactgcatcgtgatggggatagattattgcaattattagtcttcaacgaggaatgcctagtaagcgcgagtcatcagctcgcgttgatta
cgtccctgccctttgtacacaccgcccgtcgctcctaccgattgggtgtgctggtgaagtgttcggattggtttcagttgatgaacatcgactgttact
gaaaagatcattaaaccctcccacctagaggaaggagaagtcgtaacaaggtttccgtaggtgaacctgcggaaggatcattg
>AB001037.1 Chlamydomonas pulsatilla DNA for 18S rRNA, partial sequence
----
taagtatatatactgtgaaactgcgaatggctcattaaatcagttatagtttatttgatggtaccttactcggataaccgtagtaattctagagctaatac
gtgcgtaaatcccgacttctggaagggacgtatatattagataaaaggccagccgggcttgcccgaccttaggcgaatcatgatatcttcacgaat
cgcacggccttgtgccggcgatgtttcattcaaatttctgccctatcaactttcgatggtaggatagaggcctaccatggtggtaacgggtgacgga
ggattagggttcgattccggagagggagcctgagaaacggctaccacatccaaggaaggcagcaggcgcgcaaattacccaatcccgacacg
gggaggtagtgacaataaataacaataccgggcatgtctggtaattggaatgagtacaatctaaatcccttaacgagtatccattggagggcaagt
ctggtgccagcagccgcggtaattccagctccaatagcgtatatttaagttgttgcagttaaaaagctcgtagttggatttcgggtgggttctagcgg
tccgcctctggtgagtactgctaaggcctacctttctgccggggacggctcctgggattcatttctcgggactcggaatcggcgaggttactttgag
taaattagagtgttcaaagcaagccttcgctctgaatacattagcatggaataacacgataggactctggcttatcttgttggtctgtaagaccggagt
aatgattaagagggacagtcgggggcattcgtatttcattgtcagaggtgaaattcttggatttatgaaagacgaacttctgcgaaagcatttgccaa
ggatgttttcattaatcaagaacgaaagttgggggctcgaagacgattagataccgtcgtagtctcaaccataaacgatgccgactagggattggc
aggtgtttcgttgatgaccctgccagcaccttatgagaaatcaaagtttttgggttccgggggggagtatggtcgcaaggctgaaacttaaaggaatt
gacggaagggcaccaccaggcgtggagcctgcggcttaatttgactcaacacgggaaaacttaccaggtccagacacagggaggattgacag
attgagagctctttcttgattctgtgggtggtggtgcatggccgttcttagttggtgggttgccttgtcaggttgattccggtaacgaacgagacctca
gcctgctaaatagtcacgtccaccggtggacgcctgacttcttagagggactattggcgtttagccaatggaagtgtgaggcaataacaggtctgt
gatgcccttagatgttctgggccgcacgcgcgctacactgacgcattcaacgagcctatccttggccgagaggtccgggtaatctttgaaactgcg
tcgtgatggggatagattattgcaattattagtcttcaacgaggaatgcctagtaagcgcaagtcatcagcttgcgttgattacgtccctgccctttgta
cacaccgcccgtcgctcctaccgattgggtgtgctggtgaagtgttcggattgatttcagctggggcaactcggctgttgttgaaaagatcattaaa
ccctcccacctagaggaaggagaagtcgtaacaaggtttccgtaggtgaacctgcggaaggatcattg
>AB290340.1 Chlamydomonas pumilio var. pumilio gene for 18S ribosomal RNA, partial sequence
ccatgcatgtctaagtatatatactgtgaaactgcgaatggctcattaaatcagttatagtttatttgatggtaccttactcggataaccgtagtaattcta
gagctaatacgtgcgcaaatcccgacttctggaagggacgtatttattagataaaaggccagccgggcttgcccgaccttaggcgaatcatgata
acttcacgaatcgcacggcctcgtgccggcgatgtttcattcaaatttctgccctatcaactttcgatggtaggatagaggcctaccatggtggtaac
gggtgacggaggattagggttcgattccggagagggagcctgagaaacggctaccacatccaaggaaggcagcaggcgcgcaaattaccca
atcccgacacggggaggtagtgacaataaataacaataccgggcatgtctggtaattggaatgagtacaatctaaatcccttaacgagtatccattg
gagggcaagtctggtgccagcagccgcggtaattccagctccaatagcgtatatttaagttgttgcagttaaaaagctcgtagttggatttcgggtg
ggttctagcggtccgcctctggtgagtactgctaaggcctacctttctgccggggactgctcctgggattaatttctcgggacatggaatcggcgag
gttactttgagtaaattagagtgttcaaagcaagccttcgctctgaatacattagcatggaataacatgataggactctggcctatcttgttggtctgta
ggaccggagtaatgattaagagggacagtcgggggcattcgtatttcattgtcagaggtgaaattcttggatttatgaaagacgaacttctgcgaaa
gcatttgccaaggatgttttcattaatcaagaacgaaagttgggggctcgaagacgattagataccgtcgtagtctcaaccataaacgatgccgact
agggattggcaggtgtttcgttgatgaccctgccagcaccttatgagaaatcaaagtttttgggttccgggggggagtatggtcgcaaggctgaaac
ttaaaggaattgacggaagggcaccaccaggcgtggagcctgcggcttaatttgactcaacacgggaaaacttaccaggtccagacacgggga

ggattgacagattgagagctctttcttgattctgtgggtggtggtgcatggccgttcttagttggtgggttgccttgtcaggttgattccggtaacgaac
gagacctcagcctgctaaatagtcacgtccaccggtggatgcctgacttcttagagggactattggcgtttagccaatggaagtgtgaggcaataa
caggtctgtgatgcccttagatgttctgggccgcacgcgcgctacactgacgcattcaacgagcctatccttggccgagaggtccgggtaatcttt
gaaactgcgtcgtgatggggatagattattgcaattattagtcttcaacgaggaatgcctagtaagcgcaagtcatcagcttgcgttgattacgtccc
tgccctttgtacacaccgcccgtcgctcctaccgattgggtgtgctggtgaagtgttcggattggtttcagcgggggcgactctgctgttactgaga
agatcattaaaccctcccacctagaggaaggagaagtc---
>KX781334.1 Chlamydomonas reinhardtii isolate P5 18S ribosomal RNA gene and internal transcribed spacer 1, partial sequence
ccatgcatgtctaagtatatatactgtgaaactgcgaatggctcattaaatcagttatagtttatttgatggtaccctactcggataaccgtagtaattcta
gagctaatacgtgcgtaaatcccgacttctggaagggacgtatttattagataaaaggccagccgggttctcccgactcgcggtgaatcatgataa
cttcacgaatcgcatggccttgcgccggcgatgtttcattcaaatttctgccctatcaactttcgatggtaggatagaggcctaccatggtggtaacg
ggtgacggaggattagggttcgattccggagagggagcctgagagatggctaccacatccaaggaaggcagcaggcgcgcaaattacccaat
cccaacacggggaggtagtgacaataaataacaataccgggcatgtctggtaattggaatgagtacaatctaaatcccttaacgaggatccattgg
agggcaagtctggtgccagcagccgcggtaattccagctccaatagcgtatatttaagttgttgcagttaaaaagctcgtagttggatttcgggtgg
gtcttagcggtccgcctctggtgagtactgctaaggcctatctttctgccggggacggctcctgggtttaaccgcctgggactcggagtcggcgaa
gtgactttgagtaaattagagtgttcaaagcaagcctacgctctgaatacattagcatggaatcacacgataggactctggcctatcttgttggtctgt
aggaccggagtaatgattaagagggacagtcgggggcattcgtatttcattgtcagaggtgaaattcttggatttatgaaagacgaacttctgcgaa
agcatttgccaaggatgttttcattaatcaagaacgaaagttggggggctcgaagacgattagataccgtcgtagtctcaaccataaacgatgccga
ctagggattggcagatgtttcattgatgactctgccagcaccttatgagaaatcaaagtttttgggttccgggggggagtatggtcgcaaggctgaaa
cttaaaggaattgacggaagggcaccaccaggcgtggagcctgcggcttaatttgactcaacacgggaaaacttaccaggtccagacacggga
aggattgacagattgagagctctttcttgattctgtgggtggtggtgcatggccgttcttagttggtgggttgccttgtcaggttgattccggtaacgaa
cgagacctcagcctgctaaatagtcacgactgcttgcagttggccgacttcttagagggactattgtcgtgtaggcaatggaagtatgaggcaata
acaggtctgtgatgcccttagatgttctgggccgcacgcgcgctacactgacgcattcaacgagcctatccttggccgagaggcccgggtaatctt
tgaaactgcgtcgtgatggggatagattattgcaattattagtcttcaacgaggaatgcctagtaagcgcgagtcatcagctcgcgttgattacgtcc
ctgccctttgtacacaccgcccgtcgctcctaccgattgggtgtgctggtgaagtgttcggattggcttcaggtgatgaacatcgcttgttgctgaga
agttcattaaaccctcccacctagaggaaggagaagtcgtaacaaggtttccgtaggtgaacctgcggaaggatcattg
>U70791.1 Chloromonas reticulata 18S ribosomal RNA gene, partial sequence
ccatgcatgtctaagtatatatactgcgaaactgcgaatggctcattaaatcagttatagtttatttgatggtactctactcggataaccgtagtaattcta
gagctaatacgtgcgcacatcccgacttctggaagggacgtatttattagataaaaggccagccgggcttgcccgactttaggtgaatcatgataa
ctccacgaatcgcatggccttgtgccggcgatatttcattcaaatttctgccctatcaactttcgatggtaggatagaggcctaccatggtggtaacg
ggtgacggaggattagggttcgattccggagagggagcctgagaaatggctaccacatccaaggaaggcagcaggcgcgcaaattacccaat
cccgacacggggaggtagtgacaataaataacaataccgggcatgtctggtaattggaatgagcacaatctaaatcccttaacgaggatccattg
gagggcaagtctggtgccagcagccgcggtaattccagctccaatagcgtatatttaagttgttgcagttaaaaagctcgtagttggatttcggggg
ggtcttagcggtccggttc-
gctgtgtactgctagggccctcctttctgccggggacggctcttgggcttcactgtctgggacctggagtcggcgaggttactttgagtaaattaga
gtgttcaaagcaagcctacgctctgaatacattagcatggaataacacgataggactctggcctatcttgttggtctgtaggaccggagtaatgatta

agagggacagtcgggggcattcgtatttcattgtcagaggtgaaattcttggatttatgaaagacgaacttctgcgaaagcatttgccaaggatgttt
tcattaatcaagaacgaaagttgggggctcgaagacgattagataccgtcgtagtctcaaccataaacgatgccgactagggattggcaggtgttc
ttttgatgaccctgccagcaccttatgagaaatcaaagtttttgggttccggggggagtatggtcgcaaggctgaaacttaaaggaattgacggaa
gggcaccaccaggcgtggagcctgcggcttaatttgactcaacacgggaaaacttaccaggtccagacacgggaaggattgacagattgagag
ctctttcttgattctgtgggtggtggtgcatggccgttcttagttggtgggttgccttgtcaggttgattccggtaacgaacgagacctcagcctgctaa
atagtcacatctgcccgcagttggccgacttcttagagggactattgtcgtttaggcaatggaagtatgaggcaataacaggtctgtgatgccctta
gatgttctgggccgcacgcgcgctacactgatgcgttcaacgagcctatccttggccgagaggcccgggtaatcttggaaaccgcatcgtgatg
gggatagattattgcaattattagtcttcaacgaggaatgcctagtaagcgcgagtcatcagctcgcgttgattacgtccctgccctttgtacacacc
gcccgtcgctcctaccgattgggtgtgctggtgaagtgttcggatcggcttcatctggtgaacatcgggtgtcgctgagaagatcattaaaccctcc
cacctagaggaa--
>AJ781310.1 Chlamydomonas subcaudata partial 18S rRNA gene, isolate SAG 12.87
ccatgcatgtctaagtatatatactgtgaaactgcgaatggctcattaaatcagttatagtttatttgatggtacctacctcggataaccgtagtaattcta
gagctaatccgtccgtaaatcccgacttctggaagaaccgtatatattagataaaaggccagccggctt--
cccgaccttaggcgaatcatgatatcttcacgaatcgcacggccttgtgccggcgatgtttcattcaaatttctgccctatcaactttcgatggtagga
tagaggcctaccatggtggtaacgggtgacggaggattagggttcgattccggagagggagcctgagaaacggctaccacatccaaggaagg
cagcaggcgcgcaaattacccaatcccgacacggggaggtagtgacaataaataacaataccgggcatgtctggtaattggaatgagtacaatc
taaatcccttaacgagtatccattggagggcaagtctggtgccagcagccgcggtaattccagctccaatagcgtatatttaagttgttgcagttaaa
aagctcgtagttggatttcgggtgggttctagcggtccgcctctggtgagtactgctaaggcctacctttctgccggggacggctcctgggattcatt
tctcgggactcggaatcggcgaggttactttgagtaaattagagtgttcaaagcaagccttcgctctgaatacattagcatggaataacacgatagg
actctggcttatcttgttggtctgtaagaccggagtaatgattaagagggacagtcgggggcattcgtatttcattgtcagaggtgaaattcttggattt
atgaaagacgaacttctgcgaaagcatttgccaaggatgttttcattaatcaagaacgaaagttgggggctcgaagacgattagataccgtcgtagt
ctcaaccataaacgatgccgactagggattggcaggtgtttcgttgatgaccctgccagcaccttatgagaaatcaaagtttttgggttccgggggg
agtatggtcgcaaggctgaaacttaaaggaattgacggaagggcaccaccaggcgtggagcctgcggcttaatttgactcaacacgggaaaact
taccaggtccagacacagggaggattgacagattgagagctctttcttgattctgtgggtggtggtgcatggccgttcttagttggtgggttgccttg
tcaggttgattccggtaacgaacgagacctcagcctgctaaatagtcacgtccaccggtggacgcctgacttcttagagggactattggcgtttag
ccaatggaagtgtgaggcaataacaggtctgtgatgcccttagatgttctgggccgcacgcgcgctacactgacgcattcaacgagcctatccttg
gccgagaggtccgggtaatctttgaaactgcgtcgtgatggggatagattattgcaattattagtcttcaacgaggaatgcctagtaagcgcaagtc
atcagcttgcgttgattacgtccctgccctttgtacacaccgcccgtcgctcctaccgattgggtgtgctggtgaagtgttcggatcgatttcagctgg
ggcaacccggctgttgttgaaaagatcattaaaccctcccacctagaggaaggagaagtcgtaacaaggtttccgtaggtgaacctgcggaagg
atcattg
>AB007370.1 Chlamydomonas tetragama gene for 18S rRNA, partial sequence
ccatgcatgtctaagtatatatactgtgaaactgcgaatggctcattaaatcagttatagtttatttgatggtaccttactcggataaccgtagtaattcta
gagctaatacgtgcgtaaatcccgacttctggaagggacgtatttattagataaaaggccagccgagcttgctcgacccttggcgaatcatgataa
cttcacgaatcgcacggccttgtgccggcgatgtttcattcaaatttctgccctatcaactttcgatggtaggatagaggcctaccatggtggtaacg
ggtgacggaggattagggttcgattccggagagggagcctgagaaacggctaccacatccaaggaaggcagcaggcgcgcaaattacccaat
cccgacacggggaggtagtgacaataaataacaataccgggcatgtctggtaattggaatgagtacaatctaaatcccttaacgagtatccattgg

agggcaagtctggtgccagcagccgcggtaattccagctccaatagcgtatatttaagttgttgcagttaaaaagctcgtagttggatttcgggtgg
gttctagcggtccgcctctggtgagtactgctatggccttcctttctgtcggggaccgtggctgggcttcactgtccggtccgtggaatcgacgagg
ttactttgagtaaattagagtgttcaaagcaagcctacgctctgaatacattagcatggaataacacgataggactctggcctatcttgttggtctgtag
gaccggagtaatgattaagagggacagtcgggggcattcgtatttcattgtcagaggtgaaattcttggatttatgaaagacgaacttctgcgaaag
catttgccaaggatgttttcattaatcaagaacgaaagttgggggctcgaagacgattagataccgtcgtagtctcaaccataaacgatgccgacta
gggattggcaggtgttttattgatgaccctgccagcaccttatgagaaatcaaagtttttgggttccgggggggagtatggtcgcaaggctgaaactt
aaaggaattgacggaagggcaccaccaggcgtggagcctgcggcttaatttgactcaacacgggaaaacttaccaggtccagacacgggaag
gattgacagattgagagctctttcttgattctgtgggtggtggtgcatggccgttcttagttggtgggttgccttgtcaggttgattccggtaacgaacg
agacctcagcctgctaaatagtcacgtctaccggtagatgcctgacttcttagagggactattggcgtttagccaatggaagtgtgaggcaataaca
ggtctgtgatgcccttagatgttctgggccgcacgcgcgctacactgatgcattcaacgagcctatccttggccgagaggtccgggtaatctttgaa
actgcatcgtgatggggatagattattgcaattattagtcttcaacgaggaatgcctagtaagcgcgagtcatcagctcgcgttgattacgtccctgc
cctttgtacacaccgcccgtcgctcctaccgattgggtgtgctggtgaagtgttcggattagctttgacggttgaacttctgtcatggctgagaagat
cattaaaccctcccacctagaggaaggagaagtcgtaaca-----------------------------------
>KJ756821.1 Dunaliella polymorpha strain CCAP 19/7A 18S ribosomal RNA gene, partial sequence; internal transcribed spacer 1, 5.8S ribosomal RNA gene, and internal transcribed spacer 2, complete sequence; and 28S ribosomal RNA gene, partial sequence
ccatgcatgtctaagtatatatactgtgaaactgcgaatggctcattaaatcagttatagtttatttgatggtaccttactcggataaccgtagtaattcta
gagctaatacgtgcgtaaatcccgacttctggaagggacgtatttattagataaaaggccagccgggcttgcccgactcttggcgaatcatgataa
cttcacgaatcgcacggctttatgccggcgatgtttcattcaaatttctgccctatcaactttcgatggtaggatagaggcctaccatggtggtaacgg
gtgacggaggattagggttcgattccggagagggagcctgagaaacggctaccacatccaaggaaggcagcaggcgcgcaaattacccaatc
ccaacacggggaggtagtgacaataaataacaataccgggcatgtctggtaattggaatgagtacaatctaaatcccttaacgagtatccattgga
gggcaagtctggtgccagcagccgcggtaattccagctccaatagcgtatatttaagttgttgcagttaaaaagctcgtagttggatttcgggtggg
ttgtagcggtcagcctttggttagtactgctacggcctacctttctgccggggacagctcctgggcttaactgtccgggactcggaatcggcgaggt
tactttgagtaaattagagtgttcaaagcaagcctacgctctgaatacattagcatggaataacacgataggactctggcttatcttgttggtctgtaag
accggagtaatgattaagagggacagtcgggggcattcgtatttcattgtcagaggtgaaattcttggatttatgaaagacgaacttctgcgaaagc
atttgccaaggatgttttcattaatcaagaacgaaagttgggggctcgaagacgattagataccgtcgtagtctcaaccataaacgatgccgactag
ggattggcaggtgtttcgttgatgaccctgccagcaccttatgagaaatcaaagtttttgggttccgggggggagtatggtcgcaaggctgaaactta
aaggaattgacggaagggcaccaccaggcgtggagcctgcggcttaatttgactcaacacgggaaaacttaccaggtccagacacggggagg
attgacagattgagagctctttcttgattctgtgggtggtggtgcatggccgttcttagttggtgggttgccttgtcaggttgattccggtaacgaacga
gacctcagcctgctaaatagtcacgtctaccggtaggcgcctgacttcttagagggactattggcgtttagccaatggaagtgtgaggcaataaca
ggtctgtgatgcccttagatgttctgggccgcacgcgcgctacactgatgcattcaacgagcctatccttggccgagaggtccgggtaatctttgaa
actgcatcgtgatggggatagattattgcaattattagtcttcaacgaggaatgcctagtaagcgcgagtcatcagctcgcgttgattacgtccctgc
cctttgtacacaccgcccgtcgctcctaccgattgggtgtgctggtgaagtgtttggatcggtatcaatgggggaacctctgttggtactgagaaga
acattaaaccctcccacctagaggaaggagaagtcgtaacaaggtttccgtaggtgaacctgcggaaggatcattg
>KJ756819.1 Dunaliella primolecta strain CCAP 11/34 18S ribosomal RNA gene, partial sequence; internal transcribed spacer 1, 5.8S ribosomal RNA gene, and internal transcribed spacer 2, complete

sequence; and 28S ribosomal RNA gene, partial sequence

ccatgcatgtctaagtatatatactgtgaaactgcgaatggctcattaaatcagttatagtttatttgatggtaccttactcggataaccgtagtaattcta
gagctaatacgtgcgtaaatcccgacttctggaagggacgtatttattagataaaaggccagccgggcttgcccgactcttggcgaatcatgataa
cttcacgaatcgcacggctttatgccggcgatgtttcattcaaatttctgccctatcaactttcgatggtaggatagaggcctaccatggtggtaacgg
gtgacggaggattagggttcgattccggagagggagcctgagaaacggctaccacatccaaggaaggcagcaggcgcgcaaattacccaatc
ccaacacggggaggtagtgacaataaataacaataccgggcatgtctggtaattggaatgagtacaatctaaatcccttaacgagtatccattgga
gggcaagtctggtgccagcagccgcggtaattccagctccaatagcgtatatttaagttgttgcagttaaaaagctcgtagttggatttcgggtggg
ttgtagcggtcagcctttggttagtactgctacggcctacctttctgccggggacagctcctgggcttaactgtccgggactcggaatcggcgaggt
tactttgagtaaattagagtgttcaaagcaagcctacgctctgaatacattagcatggaataacacgataggactctggcttatcttgttggtctgtaag
accggagtaatgattaagagggacagtcgggggcattcgtatttcattgtcagaggtgaaattcttggatttatgaaagacgaacttctgcgaaagc
atttgccaaggatgttttcattaatcaagaacgaaagttgggggctcgaagacgattagataccgtcgtagtctcaaccataaacgatgccgactag
ggattggcaggtgtttcgttgatgaccctgccagcaccttatgagaaatcaaagtttttgggttccgggggagtatggtcgcaaggctgaaactta
aaggaattgacggaagggcaccaccaggcgtggagcctgcggcttaatttgactcaacacgggaaaacttaccaggtccagacacggggagg
attgacagattgagagctctttcttgattctgtgggtggtggtgcatggccgttcttagttggtgggttgccttgtcaggttgattccggtaacgaacga
gacctcagcctgctaaatagtcacgtctaccggtaggcgcctgacttcttagagggactattggcgtttagccaatggaagtgtgaggcaataaca
ggtctgtgatgcccttagatgttctgggccgcacgcgcgctacactgatgcattcaacgagcctatccttggccgagaggtccgggtaatctttgaa
actgcatcgtgatggggatagattattgcaattattagtcttcaacgaggaatgcctagtaagcgcgagtcatcagctcgcgttgattacgtccctgc
cctttgtacacaccgcccgtcgctcctaccgattgggtgtgctggtgaagtgtttggatcggtatcaatgggggaacctctgttggtactgagaaga
acattaaaccctcccacctagaggaaggagaagtcgtaacaaggtttccgtaggtgaacctgcggaaggatcattg

>KU641615.1 Dunaliella pseudosalina isolate MAH 18S ribosomal RNA gene, partial sequence; internal transcribed spacer 1, 5.8S ribosomal RNA gene, and internal transcribed spacer 2, complete sequence; and 28S ribosomal RNA gene, partial sequence

----

gtctaagtatatatactgtgaaactgcgaatggctcattaaatcagttatagtttatttgatggtaccttactcggataaccgtagtaattctagagctaat
acgtgcgtaaatcccgacttctggaagggacgtatttattagataaaaggccagccgggcttgcccgactcttggcgaatcatgataacttcacga
atcgcacggctttatgccggcgatgtttcattcaaatttctgccctatcaactttcgatggtaggatagaggcctaccatggtggtaacgggtgacgg
aggattagggttcgattccggagagggagcctgagaaacggctaccacatccaaggaaggcagcaggcgcgcaaattacccaatcccaacac
ggggaggtagtgacaataaataacaataccgggcatgtctggtaattggaatgagtacaatctaaatcccttaacgagtatccattggagggcaag
tctggtgccagcagccgcggtaattccagctccaatagcgtatatttaagttgttgcagttaaaaagctcgtagttggatttcgggtgggttgtagcg
gtcagcctttggttagtactgctacggcctacctttctgccggggacagctcctgggcttaactgtccgggactcggaatcggcgaggttactttga
gtaaattagagtgttcaaagcaagcctacgctctgaatacattagcatggaataacacgataggactctggcttatcttgttggtctgtaagaccgga
gtaatgattaagagggacagtcgggggcattcgtatttcattgtcagaggtgaaattcttggatttatgaaagacgaacttctgcgaaagcatttgcc
aaggatgttttcattaatcaagaacgaaagttgggggctcgaagacgattagataccgtcgtagtctcaaccataaacgatgccgactagggattg
gcaggtgtttcgttgatgaccctgccagcaccttatgagaaatcaaagtttttgggttccgggggagtatggtcgcaaggctgaaacttaaagga
attgacggaagggcaccaccaggcgtggagcctgcggcttaatttgactcaacacgggaaaacttaccaggtccagacacggggaggattgac
agattgagagctctttcttgattctgtgggtggtggtgcatggccgttcttagttggtgggttgccttgtcaggttgattccggtaacgaacgagacct

cagcctgctaaatagtcacgtctaccggtaggcgcctgacttcttagagggactattggcgtttagccaatggaagtgtgaggcaataacaggtct
gtgatgcccttagatgttctgggccgcacgcgcgctacactgatgcattcaacgagcctatccttggccgagaggtccgggtaatctttgaaactg
catcgtgatggggatagattattgcaattattagtcttcaacgaggaatgcctagtaagcgcgagtcatcagctcgcgttgattacgtccctgcccttt
gtacacaccgcccgtcgctcctaccgattgggtgtgctggtgaagtgtttggatcggtatcaatgggggaacctctgttggtactgagaagaacatt
aaaccctcccacctagaggaaggagaagtcgtaacaaggtttccgtaggtgaacctgcggaaggatcattg

>KJ756824.1 Dunaliella quartolecta strain CCAP 19/8 18S ribosomal RNA gene, partial sequence; internal transcribed spacer 1, 5.8S ribosomal RNA gene, and internal transcribed spacer 2, complete sequence; and 28S ribosomal RNA gene, partial sequence

ccatgcatgtctaagtatatatactgtgaaactgcgaatggctcattaaatcagttatagtttatttgatggtaccttactcggataaccgtagtaattcta
gagctaatacgtgcgtaaatcccgacttctggaagggacgtatttattagataaaaggccagccgggcttgcccgactcttggcgaatcatgataa
cttcacgaatcgcacggctttatgccggcgatgtttcattcaaatttctgccctatcaactttcgatggtaggatagaggcctaccatggtggtaacgg
gtgacggaggattagggttcgattccggagagggagcctgagaaacggctaccacatccaaggaaggcagcaggcgcgcaaattacccaatc
ccaacacggggaggtagtgacaataaataacaataccgggcatgtctggtaattggaatgagtacaatctaaatcccttaacgagtatccattgga
gggcaagtctggtgccagcagccgcggtaattccagctccaatagcgtatatttaagttgttgcagttaaaaagctcgtagttggatttcgggtggg
ttgtagcggtcagcctttggttagtactgctacggcctacctttctgccggggacagctcctgggcttaactgtccgggactcggaatcggcgaggt
tactttgagtaaattagagtgttcaaagcaagcctacgctctgaatacattagcatggaataacacgataggactctggcttatcttgttggtctgtaag
accggagtaatgattaagagggacagtcgggggcattcgtatttcattgtcagaggtgaaattcttggatttatgaaagacgaacttctgcgaaagc
atttgccaaggatgttttcattaatcaagaacgaaagttgggggctcgaagacgattagataccgtcgtagtctcaaccataaacgatgccgactag
ggattggcaggtgtttcgttgatgaccctgccagcaccttatgagaaatcaaagtttttgggttccgggggggagtatggtcgcaaggctgaaactta
aaggaattgacggaagggcaccaccaggcgtggagcctgcggcttaatttgactcaacacgggaaaacttaccaggtccagacacggggagg
attgacagattgagagctctttcttgattctgtgggtggtggtgcatggccgttcttagttggtgggttgccttgtcaggttgattccggtaacgaacga
gacctcagcctgctaaatagtcacgtctaccggtaggcgcctgacttcttagagggactattggcgtttagccaatggaagtgtgaggcaataaca
ggtctgtgatgcccttagatgttctgggccgcacgcgcgctacactgatgcattcaacgagcctatccttggccgagaggtccgggtaatctttgaa
actgcatcgtgatggggatagattattgcaattattagtcttcaacgaggaatgcctagtaagcgcgagtcatcagctcgcgttgattacgtccctgc
cctttgtacacaccgcccgtcgctcctaccgattgggtgtgctggtgaagtgtttggatcggtatcaatgggggaacctctgttggtactgagaaga
acattaaaccctcccacctagaggaaggagaagtcgtaacaaggtttccgtaggtgaacctgcggaaggatcattg

>JQ315781.1 Dunaliella salina strain KMMCC 1428 18S ribosomal RNA gene, internal transcribed spacer 1, 5.8S ribosomal RNA gene, internal transcribed spacer 2, and 28S ribosomal RNA gene region

--accgtagt-
attctagagctaatacgtgcgtaaatcccgacttctggaagggacgtatttattagataaaaggccagccgggcttgcccgactcttggcgaatcat
gataacttcacgaatcgcacggctttatgccggcgatgtttcattcaaatttctgccctatcaactttcgatggtaggatagaggcctaccatggtggt
aacgggtgacggaggattagggttcgattccggagagggagcctgagaaacggctaccacatccaaggaaggcagcaggcgcgcaaattac
ccaatcccaacacggggaggtagtgacaataaataacaataccgggcatgtctggtaattggaatgagtacaatctaaatcccttaacgagtatcc
attggagggcaagtctggtgccagcagccgcggtaattccagctccaatagcgtatatttaagttgttgcagttaaaaagctcgtagttggatttcgg
gtgggttgtagcggtcagcctttggttagtactgctacggcctacctttctgccggggacagctcctgggcttaactgtccgggactcggaatcgg
cgaggttactttgagtaaattagagtgttcaaagcaagcctacgctctgaatacattagcatggaataacacgataggactctggcttatcttgttggt

ctgtaagaccggagtaatgattaagagggacagtcgggggcattcgtatttcattgtcagaggtgaaattcttggatttatgaaagacgaacttctgc
gaaagcatttgccaaggatgttttcattaatcaagaacgaaagttgggggctcgaagacgattagataccgtcgtagtctcaaccataaacgatgc
cgactagggattggcaggtgtttcgttgatgaccctgccagcaccttatgagaaatcaaagtttttgggttccgggggggagtatggtcgcaaggct
gaaacttaaaggaattgacggaagggcaccaccaggcgtggagcctgcggcttaatttgactcaacacgggaaaacttaccaggtccagacac
ggggaggattgacagattgagagctctttcttgattctgtgggtggtggtgcatggccgttcttagttggtgggttgccttgtcaggttgattccggta
acgaacgagacctcagcctgctaaatagtcacgtctaccggtaggcgcctgacttcttagagggactattggcgtttagccaatggaagtgtgag
gcaataacaggtctgtgatgcccttagatgttctgggccgcacgcgcgctacactgatgcattcaacgagcctatccttggccgagaggtccggg
taatctttgaaactgcatcgtgatggggatagattattgcaattattagtcttcaacgaggaatgcctagtaagcgcgagtcatcagctcgcgttgatt
acgtccctgccctttgtacacaccgcccgtcgctcctaccgattgggtgtgctggtgaagtgtttggatcggtatcaatgggggaacctctgttggta
ctgagaagaacattaaaccctcccacctagaggaaggagaagtcgtaacaaggtttccgtaggtgaacctgcggaaggatcattg

>KJ756820.1 Dunaliella tertiolecta strain CCAP 19/6B 18S ribosomal RNA gene, partial sequence; internal transcribed spacer 1, 5.8S ribosomal RNA gene, and internal transcribed spacer 2, complete sequence; and 28S ribosomal RNA gene, partial sequence

ccatgcatgtctaagtatatatactgtgaaactgcgaatggctcattaaatcagttatagtttatttgatggtaccttactcggataaccgtagtaattcta
gagctaatacgtgcgtaaatcccgacttctggaagggacgtatttattagataaaaggccagccgggcttgcccgactcttggcgaatcatgataa
cttcacgaatcgcacggctttatgccggcgatgtttcattcaaatttctgccctatcaactttcgatggtaggatagaggcctaccatggtggtaacgg
gtgacggaggattagggttcgattccggagagggagcctgagaaacggctaccacatccaaggaaggcagcaggcgcgcaaattacccaatc
ccaacacggggaggtagtgacaataaataacaataccgggcatgtctggtaattggaatgagtacaatctaaatcccttaacgagtatccattgga
gggcaagtctggtgccagcagccgcggtaattccagctccaatagcgtatatttaagttgttgcagttaaaaagctcgtagttggatttcgggtggg
ttgtagcggtcagcctttggttagtactgctacggcctacctttctgccggggacagctcctgggcttaactgtccgggactcggaatcggcgaggt
tactttgagtaaattagagtgttcaaagcaagcctacgctctgaatacattagcatggaataacacgataggactctggcttatcttgttggtctgtaag
accggagtaatgattaagagggacagtcgggggcattcgtatttcattgtcagaggtgaaattcttggatttatgaaagacgaacttctgcgaaagc
atttgccaaggatgttttcattaatcaagaacgaaagttgggggctcgaagacgattagataccgtcgtagtctcaaccataaacgatgccgactag
ggattggcaggtgtttcgttgatgaccctgccagcaccttatgagaaatcaaagtttttgggttccgggggggagtatggtcgcaaggctgaaactta
aaggaattgacggaagggcaccaccaggcgtggagcctgcggcttaatttgactcaacacgggaaaacttaccaggtccagacacggggagg
attgacagattgagagctctttcttgattctgtgggtggtggtgcatggccgttcttagttggtgggttgccttgtcaggttgattccggtaacgaacga
gacctcagcctgctaaatagtcacgtctaccggtaggcgcctgacttcttagagggactattggcgtttagccaatggaagtgtgaggcaataaca
ggtctgtgatgcccttagatgttctgggccgcacgcgcgctacactgatgcattcaacgagcctatccttggccgagaggtccgggtaatctttgaa
actgcatcgtgatggggatagattattgcaattattagtcttcaacgaggaatgcctagtaagcgcgagtcatcagctcgcgttgattacgtccctgc
cctttgtacacaccgcccgtcgctcctaccgattgggtgtgctggtgaagtgtttggatcggtatcaatgggggaacctctgttggtactgagaaga
acattaaaccctcccacctagaggaaggagaagtcgtaacaaggtttccgtaggtgaacctgcggaaggatcattg

>GU292342.1 Ettlia carotinosa strain SAG 213-4 18S ribosomal RNA gene, partial sequence

ccatgcatgtctaagtatatatacggtgaaactgcgaatggctcattaaatcagttatagtttatttgatggtactttactcggataaccgtagtaattcta
gagctaatacgtgcgtaaatcccgacttctggaagggacgtatttattagataaaaggccagccgggcttgcccgacctatggcgaatcatgataa
cttcacgaatcgcacggccttgcgccggcgatgtttcattcaaatttctgccctatcaactttcgatggtaggatagaggcctaccatggtggtaacg
ggtgacggaggattagggttcgattccggagagggagcctgagaaacggctaccacatccaaggaaggcagcaggcgcgcaaattacccaat

cccgacacggggaggtagtgacaataaataacaataccgggcatgtctggtaattggaatgagaacaatttaaatcccttaacgagtatccattgg
agggcaagtctggtgccagcagccgcggtaattccagctccaatagcgtatatttaagttgttgcagttaaaaagctcgtagttggatttcgggtgg
gttccagcggtctgcctctggtatgtactgctgtggcctacctttctgccggggactgctcctgggcttaactgtccgggactcgaattcggcgagg
atactttgagtaaaacagcgtgttcaaagcaagcctacgctctgaatgcattagcatggaatatcacgataggactctggcctatcttgttggtctgta
ggaccggagtaatgattaagagggacagtcgggggcattcgtatttcattgtcagaggtgaaattcttggatttatgaaagacgaacttctgcgaaa
gcatttgccaaggatgttttcattaatcaagaacgaaagttgggggctcgaagacgattagataccgtcgtagtctcaaccataaacgatgccgact
agggattggcaggtgttttattgatgaccctgccagcaccttatgagaaatcaaagtttttgggttccggggggagtatggtcgcaaggctgaaact
taaaggaattgacggaagggcaccaccaggcgtggagcctgcggcttaatttgactcaacacgggaaaacttaccaggtccagacacgggaa
ggattgacagattgagagctctttcttgattctgtgggtggtggtgcatggccgttcttagttggtgggttgccttgtcaggttgattccggtaacgaac
gagacctcagcctgctaaatagtcaagcgtaccggtacgcgcctgacttcttagagggactattgacgtttagtcagtggaagtgtgaggcaataa
caggtctgtgatgcccttagatgttctgggccgcacgcgcgctacactgatgcattcagcgagcctatccttggccgagaggtccgggtaatcttt
gaaactgcatcgtgatggggatagattattgcaattattagtcttcaacgaggaatgcctagtaagcgcgagt-
atcagctcgcgttgattacgtccctgccctttgtacacaccgcccgtcgctcctaccgattgggtgtgctggtgaagtgttcggattgacttcagcgg
tggaacctctgctgttgttgagaagatcattaaaccctcccacctagaggaaggagaagtcgtaacaaggtctccgtaggtgaa-
ctgcggagggatca---

>AB360743.1 Gungnir kasakii gene for 18S ribosomal RNA, partial sequence, strain: NIES-1359 (= SkCl-5)

ccatgcatgtctaagtatatatactgtgaaactgcgaatggctcattaaatcagttatagtttatttgatggtaccttactcggataaccgtagtaattcta
gagctaatacgtgcgtaaatcccgacttctggaagggacgtatttattagataaaaggccagccgggcttgcccgactcttggcgaatcatgataa
cttcacgaatcgcacggctttatgccggcgatgtttcattcaaatttctgccctatcaactttcgatggtaggatagaggcctaccatggtggtaacgg
gtgacggaggattagggttcgattccggagagggagcctgagaaacggctaccacatccaaggaaggcagcaggcgcgcaaattacccaatc
ccgacacggggaggtagtgacaataaataacaataccgggcatgtctggtaattggaatgagtacaatctaaatcccttaacgagtaaccattgga
gggcaagtctggtgccagcagccgcggtaattccagctccaatagcgtatatttaagttgttgcagttaaaaagctcgtagttggatttcgggtggg
ttctagcggtccgcctctggtgagtactgctatggcctacctttctgccggggacggctcctgggcttaactgtccgggactcggaatcggcgagg
ttactttgagtaaattagagtgttcaaagcaagcctacgctctgaatacattagcatggaataacacgataggactctggcctatcttgttggtctgtag
gaccggagtaatgattaagagggacagtcgggggcattcgtatttcattgtcagaggtgaaattcttggatttatgaaagacgaacatctgcgaaa
gcatttgccaaggatgttttcattaatcaagaacgaaagttgggggctcgaagacgattagataccgtcgtagtctcaaccataaacgatgccgact
agggattggcaggtgttcttttgatgaccctgccagcaccttatgagaaatcaaagtttttgggttccggggggagtatggtcgcaaggctgaaact
taaaggaattgacggaagggcaccaccaggcgtggagcctgcggcttaatttgactcaacacgggaaaacttaccaggtccagacacgggga
ggattgacagattgagagctctttcttgattctgtgggtggtggtgcatggccgttcttagttggtgggttgccttgtcaggttgattccggtaacgaac
gagacctcagcctgctaaatagtcacggctacttgtaggcgcttgacttcttagagggactattgacgtttagtcaatggaagtgtgaggcaataac
aggtctgtgatgcccttagatgttctgggccgcacgcgcgctacactgatgcattcaacgagcctatccttggccgagaggtccgggtaatctttga
aactgcatcgtgatggggctagatcattgcaattattggtcttcaacgaggaatgcctagtaagcgcgagtcatcagctcgcgttgattacgtccctg
ccctttgtacacaccgcccgtcgctcctaccgattgggtgtgctggtgaagtgtccggatcggcttttgtgggggaacctctgcttttgctgaaaag
gtcattaaaccctcccacctagaggaaggagaagtcgtaacaaggtttcc---------------------------

>AF159369.1 Haematococcus lacustris strain SAG 34-1b culture-collection SAG:34-1b 18S

ribosomal RNA gene, complete sequence

ccatgcatgtctaagtatatatacggtgaaactgcgaatggctcattaaatcagttatagtttatttgatggtactttactcggataaccgtagtaattcta
gagctaatacgtgcgtatatcccgacttctggaagggacgtatttattagataaaaggccagccgggcttgcccgacctatggcgaatcatgataa
cttcacgaatcgcacggccttgcgccggcgatgtttcattcaaatttctgccctatcaactttcgatggtaggatagaggcctaccatggtggtaacg
ggtgacggaggattagggttcgattccggagagggagcctgagaaacggctaccacatccaaggaaggcagcaggcgcgcaaattacccaat
cccgacacggggaggtagtgacaataaataacaataccgggcatgtctggtaattggaatgagaacaatttaaatcccttaacgagtatccattgg
agggcaagtctggtgccagcagccgcggtaattccagctccaatagcgtatatttaagttgttgcagttaaaaagctcgtagttggatttcgggtgg
gttccagcggtctgcctctggtatgtactgctgtggcctacctttctgccggggactgttcctgggcttcattgtccgggactcgaattcggcgagga
tactttgagtaaaacagcgtgttcaaagcaagcctacgctctgaatgcattagcatggaatatcacgataggactctggcctatcttgttggtctgtag
gaccggagtaatgattaagagggacagtcgggggcattcgtatttcattgtcagaggtgaaattcttggatttatgaaagacgaacttctgcgaaag
catttgccaaggatgttttcattaatcaagaacgaaagttgggggctcgaagacgattagataccgtcgtagtctcaaccataaacgatgccgacta
gggattggcaggtgttttattgatgaccctgccagcaccttatgagaaatcaaagtttttgggttccggggggagtatggtcgcaaggctgaaactt
aaaggaattgacggaagggcaccaccaggcgtggagcctgcggcttaatttgactcaacacgggaaaacttaccaggtccagacacgggaag
gattgacagattgagagctctttcttgattctgtgggtggtggtgcatggccgttcttagttggtgggttgccttgtcaggttgattccggtaacgaacg
agacctcagcctgctaaatagtcaagcgtaccggtacgggctgaacttcttagagggactattgacgtttagtcagtggaagtgtgaggcaataac
aggtctgtgatgcccttagatgttctgggccgcacgcgcgctacactgatgcattcagcgagcctatccttggccgagaggtccgggtaatctttg
aaactgcatcgtgatggggatagattattgcaattattagtcttcaacgaggaatgcctagtaagcgcgagtcatcagctcgcgttgattacgtccct
gccctttgtacacaccgcccgtcgctcctaccgattgggtgtgctggtgaagtgttcggattgactttagcggtggaacctctgctgtcgttgagaa
gaacattaaaccctcccacctagaggaaggagaagtcgtaacaaggtctccgtaggtgaa-----------------

>AJ410446.1 Ixipapillifera deasonii (Chloromonas carrizoensis) partial 18S rRNA gene, strain SAG 46.72

ccatgcatgtctaagtatatatactgtgaaactgcgaatggctcattaaatcagttatagtttatttgatggtcttctacttggatacccgtagtaattctag
agctaatacatgcgcacatcccgacttctggaagggacgtatttattagataaaaggccagccgagcttgctcgactttcggtgaatcatgataact
ctacgaatcgcatggcctttggccggcgatgtttcattcaaatttctgccctatcaactttcgatggtaggatagaggcctaccatggtggtaacggg
tgacggaggattagggttcgattccggagagggagcctgagagatggctaccacatccaaggaaggcagcaggcgcgcaaattacccaatcc
cgacacggggaggtagtgacaataaataacaataccgggcatgtctggtaattggaatgagcacaatctaaatcccttaacgaggatccattgga
gggcaagtctggtgccagcagccgcggtaattccagctccaatagcgtatatttaagttgttgcagttaaaaagctcgtagttggatttcgggtggg
tcgcaacggtccggctctgctgtgcactgttgtggccttcctttctgccggtgacggctcttgggcttcactgtctgggacccggagtcggcgatgtt
actttgagtaaattagagtgttcaaagcaagcctacgctctgaatacattagcatggaataacacgataggactctggcctatcttgttggtctgtagg
accggagtaatgattaagagggacagtcgggggcattcgtatttcattgtcagaggtgaaattcttggatttatgaaagacgaacttctgcgaaagc
atttcgcaaggatgttttcattaatcaagaacgaaagttgggggctcgaagacgattagataccgtcgtagtctcaaccataaacgatgccgactag
ggattggcgggtgttcttttgatgaccccgccagcaccttatgagaaatcaaagtttttgggttccggggggagtatggtcgcaaggctgaaactta
aaggaattgacggaagggcaccaccaggtgtggagcctgcggcttaatttgactcaacacgggaaaacttaccaggtccagacacggggagg
attgacagattgagagctctttcttgattctgtgggtggtggtgcatggccgttcttagttggtgggttgccttgtcaggttgattccggtaacgaacga
gacctcagcctgctaaatagtcacatctgcccgcagctggccgacttcttagagggactattgtcgtttaggcaatggaagtatgaggcaataaca
ggtctgtgatgcccttagatgttctgggccgcacgcgcgctacactgatgcgttcaacaagcctatccttggccgagaggcccgggtaatcttaga

atccgcatcgtgatggggatagatcattgcaattgttggtcttcaacgaggaatacctagtaggcacaagtcatcagcttgtgccgattacgtccctg
cccttttgtacacaccgcccgtcgctcctaccgattgggtgtgctggtgaagtgtccggattggcttcagctggtgaacactggtcgtcgctgaaaa
ggtcattaaaccctcccacctagaggaaggagaagtcgtaacaaggtcttcgtaggtgaacctgcggaaggatcattg
>AF395436.1 Lobocharacium coloradoense strain Kugrens 18S ribosomal RNA gene, partial sequence
ccatgcatgtctaagtatatatactgtgaaactgcgaatggctcattaaatcagttatagtttatttgatggtaccctactcggataaccgtagtaattcta
gagctaatacgtgcgtaaatcccgacttctggaagggacgtatttattagataaaaggccagccgggcttgcccgacctgcggtgaatcatgataa
cttcacgaatcgcacggcctcgtgccggcgatgtttcattcaaatttctgccctatcaactttcgatggtaggatagaggcctaccatggtggtaacg
ggtgacggaggattagggttcgattccggagagggagcctgagaaacggctaccacatccaaggaaggcagcaggcgcgcaaattacccaat
cccgacacggggaggtagtgacaataaataacaataccgggcatgtctggtaattggaatgagtacaatctaaatcccttaacgaggatccattgg
agggcaagtctggtgccagcagccgcggtaattccagctccaatagcgtatatttaagttgttgcagttaaaaagctcgtagttggatttcgggtgg
gttccagcggtccgcctctggtgtgcactgctgaggcctacctttctgccggggacggctcttgggcttaactgtccgggactcggaatcggcga
ggttactttgagtaaattagagtgttcaaagcaggcctacgctctgaatacattagcatggaataacacgataggactctggcctatcttgttggtctg
taggaccggagtaatgattaagagggacagtcgggggcattcgtatttcattgtcagaggtgaaattcttggatttatgaaagacgaacttctgcga
aagcatttgccaaggatgttttcattaatcaagaacgaaagttgggggctcgaagacgattagataccgtcgtagtctcaaccataaacgatgccg
actagggattggcaggtgttcttttgatgaccctgccagcaccttatgagaaatcaaagtttttgggttccggggggagtatggtcgcaaggctgaa
acttaaaggaattgacggaagggcaccaccaggcgtggagcctgcggcttaatttgactcaacacgggaaaacttaccaggtccagacacggg
aaggattgacagattgagagctctttcttgattctgtgggtggtggtgcatggccgttcttagttggtgggttgccttgtcaggttgattccggtaacga
acgagacctcagcctgctaaatagtcacgggcaccggtgcacgcttgacttcttagagggactattggcgtttagccagtggaagtgtgaggcaa
taacaggtctgtgatgcccttagatgttctgggccgcacgcgcgctacactgatgcattcaacgagcctatccttggccgagaggcccgggtaatc
tttgaatctgcatcgtgatggggatagattattgcaattattagtcttcaacgaggaatgcctagtaagcgcaagtcatcagcttgcgttgattacgtcc
ctgccctttgtacacaccgcccgtcgctcctaccgattgggtgtgctggtgaagtgtccggattggctttggctggtgaacatcgaccatggccga
aaaggacattaaaccctcccaccta---
>FR854374.1 Microglena basinucleata genomic DNA containing 18S rRNA, ITS1, 5.8S rRNA, ITS2 and 28S rRNA, strain SAG 67.72
ccatgcatgtctaagtatatatactgtgaaactgcgaatggctcattaaatcagttatagtttatttgatggtacactactcggataaccgtagtaattcta
gagctaatacgtgcgtaaatcccgacttctggaagggacgtatttattagataaaaggccagccggagttctccgacctgcggtgaatcatgataa
cttcacgaatcgcacggccttgcgccggcgatgtttcattcaaatttctgccctatcaactttcgatggtaggatagaggcctaccatggtggtaacg
ggtgacggaggattagggttcgattccggagagggagcctgagaaacggctaccacatccaaggaaggcagcaggcgcgcaaattacccaat
cccgacacggggaggtagtgacaataaataacaataccgggcatgtctggtaattggaatgagtacaatctaaatcccttaacgaggatccattgg
agggcaagtctggtgccagcagccgcggtaattccagctccaatagcgtatatttaagttgttgcagttaaaaagctcgtagttggatttcgggtgg
gtgttagcggtccggctctgctgtgtactgctaatgcctacctttctgtcggggacggctcttggactttactgtccgggactcggagtcgacgaggt
tactttgagtaaattagagtgttcaaagcaggcgtacgctatgaatacattagcatggaataacacgataggactctggcttatcttgttggtctgtaa
gaccggagtaatgattaagagggacagtcgggggcattcgtatttcattgtcagaggtgaaattcttggatttatgaaagacgaacttctgcgaaag
catttgccaaggatgttttcattaatcaagaacgaaagttgggggctcgaagacgattagataccgtcgtagtctcaaccataaacgatgccgacta
gggattggcaggtgtttcgttgatgaccctgccagcaccttatgagaaatcaaagtttttgggttccggggggagtatggtcgcaaggctgaaactt

aaaggaattgacggaagggcaccaccaggcgtggagcctgcggcttaatttgactcaacacgggaaaacttaccaggtccagacacggggag
gattgacagattgagagctctttcttgattctgtgggtggtggtgcatggccgttcttagttggtgggttgccttgtcaggttgattccggtaacgaacg
agacctcagcctgctaaatagtcacggctacttgtagccgccagacttcttagagggactattgtcgtttaggcaatggaagtgtgaggcaataaca
ggtctgtgatgcccttagatgttctgggccgcacgcgcgctacactgttgcattcaacgagcctatccttggccgagaggtccgggtaatctttgaa
actgcaacgtgatggggatagattattgcaattattagtcttcaacgaggaatgcctagtaagcgcgagtcatcagctcgcgttgattacgtccctgc
cctttgtacacaccgcccgtcgctcctaccgattgggtgtgctggtgaagtgttcggattggtttcagttgatgaacatcggctgttgctgagaagat
cattaaaccctcccacctagaggaaggagaagtcgtaacaaggtttccgtaggtgaacctgcggaaggatcattg
>FR854385.1 Microglena monadina genomic DNA containing 18S rRNA, ITS1, 5.8S rRNA, ITS2 and 28S rRNA, strain SAG 55.72
ccatgcatgtctaagtatatatactgtgaaactgcgaatggctcattaaatcagttatagtttatttgatggtacactactcggataaccgtagtaattcta
gagctaatacgtgcgtaaatcccgacttctggaagggacgtatttattagataaaaggccagccggagttctccgacctgcggtgaatcatgataa
cttcacgaatcgcacggccttgcgccggcgatgtttcattcaaatttctgccctatcaactttcgatggtaggatagaggcctaccatggtggtaacg
ggtgacggaggattagggttcgattccggagagggagcctgagaaacggctaccacatccaaggaaggcagcaggcgcgcaaattacccaat
cccgacacggggaggtagtgacaataaataacaataccgggcatgtctggtaattggaatgagtacaatctaaatcccttaacgaggatccattgg
agggcaagtctggtgccagcagccgcggtaattccagctccaatagcgtatatttaagttgttgcagttaaaaagctcgtagttggatttcgggtgg
gtgttagcggtccggctctgctgtgtactgctagtgcctacctttctgtcggggacggctcttggacttaattgtccgggactcggagtcgacgagg
ttactttgagtaaattagagtgttcaaagcaggcgtacgctatgaatacattagcatggaataacacgataggactctggcttatcttgttggtctgtaa
gaccggagtaatgattaagagggacagtcgggggcattcgtatttcattgtcagaggtgaaattcttggatttatgaaagacgaacttctgcgaaag
catttgccaaggatgttttcattaatcaagaacgaaagttgggggctcgaagacgattagataccgtcgtagtctcaaccataaacgatgccgacta
gggattggcaggtgtttcgttgatgaccctgccagcaccttatgagaaatcaaagtttttgggttccgggggagtatggtcgcaaggctgaaactt
aaaggaattgacggaagggcaccaccaggcgtggagcctgcggcttaatttgactcaacacgggaaaacttaccaggtccagacacgggaat
gattgacagattgagagctctttcttgattctgtgggtggtggtgcatggccgttcttagttggtgggttgccttgtcaggttgattccggtaacgaacg
agacctcagcctgctaaatagtcacggctacttgtagccgccagacttcttagagggactattgtcgtttaggcaatggaagtgtgaggcaataaca
ggtctgtgatgcccttagatgttctgggccgcacgcgcgctacactgttgcattcaacgagcctatccttgaccgagaggtccgggtaatctttgaa
actgcaacgtgatggggatagattattgcaattattagtcttcaacgaggaatgcctagtaagcgcgagtcatcagctcgcgttgattacgtccctgc
cctttgtacacaccgcccgtcgctcctaccgattgagtgtgctggtgaagtgttcggattggtttcagtcgatgaacatctgctgttgctgagaagatc
attaaaccctcccacttagaggaaggagaagtcgtaacaaggtttccgtaggtgaacctgcggaaggatcattg
>chlamydomonad sp. NrCl902

----
gggcttgcccgactttaggcgaatcatgataacttcacgaatcgcacggcctcgtgccggcgatgtttcattcaaatttctgccctatcaactttcgat
ggtaggatagaggcctaccatggtggtaacgggtgacggaggattagggttcgattccggagagggtgcctgagaaacggcaaccacatcca
aggaaggcagcaggcgcgcaaattacccaatcccaacacggggaggtagtgacaataaataacaataccgggcatgtctggtaattggaatga
gtacaatttaaatcccttaacgagtaccaattggagggcaagtctggtgccagcagccgcggtaattccagctccaatagcgtatatttaagttgttg
cagttaaaaagctcgtagttggatttcgggtgggttctagcggtctgcctctggtatgtactgctatggctcacctttctgccggggacggctcctgg
gcttcactgtccgggactcggaatcggcgaggttactttgagtaaattagagtgttcaaagcaagcctatgctctgaatatattagcatggaataaca

cgataggactctggcctatcttgttggtctgtaggaccggagtaatgattaagagggacagtcgggggcattcgtatttcattgtcagaggtgaaatt
cttggatttatgaaagacgaacttctgcgaaagcatttgccaaggatgttttcattaatcaagaacgaaagttgggggctcgaagacgattagatac
cgtcgtagtctcaaccataaacgatgccgactagggattggcaggtgtttcgttgatgaccctgccagcaccttatgagaaatcaaagtttttgggtt
ccgggggggagtatggtcgcaaggctgaaacttaaaggaattgacggaagggcaccaccaggcgtggagcctgcggcttaatttgactcaacac
gggaaaacttaccaggtccagacacggggaggattgacagattgagagctctttcttgattctgtgggtggtggtgcatggccgttcttagttggtg
ggttgccttgtcaggttgattccggtaacgaacgagacctcagcctgctaaatagtcacgggcaccggtgcacgcctgacttcttagagggactat
tgacgtttagtcaatggaagtgtgaggcaataacaggtctgtgatgcccttagatgttctgggccgcacgcgcgctacactgatgcattcaacgag
cctatccttggccgagaggcccgggtaatctttgaaactgcatcgtgatggggatagattattgcaattattagtcttcaacgaggaatgcctagtaa
gcgcaagtcatcagcttgcgttgattacgtccctgccctttgtacacaccgcccgtcgctcctaccgattgggtgtgctggtgaagtgttcggattga
ccttggctgatgaacatcggccttggttgagaagatcattgaaccctcccacctagaggaaggagaagtcgtaacaaggtctccgtaggtgaacc
tgcggagggatcattg
>AJ410469.1 Oogamochlamys ettlii partial 18S rRNA gene, strain UTEX 2218
ccatgcatgtctaagtatatatactgtgaaactgcgaatggctcattaaatcagttatagtttatttgatggtaccctactcggataaccgtagtaattcta
gagctaatacgtgcgtaaatcccgacttctggaagggacgtatttattagataaaaggccagccgggctctgcccgactgcggtgaatcatgataa
cttcacgaatcgcacggcctcgtgccggcgatgtttcattcaaatttctgccctatcaactttcgatggtaggatagaggcctaccatggtggtaacg
ggtgacggaggattagggttcgattccggagagggagcctgagagatggctaccacatccaaggaaggcagcaggcgcgcaaattacccaat
cccgacacggggaggtagtgacaataaataacaataccgggcatgtctggtaattggaatgagtacaatctaaatcccttaacgaggatccattgg
agggcaagtctggtgccagcagccgcggtaattccagctccaatagcgtatatttaagttgttgcagttaaaaagctcgtagttggatttcggatgg
gtctcagcggtccgcctctggtgtgtactgctgcggcctatctttctgccggggacggctcctgggcttcactgtctgggactcggagtcggcgag
gttactttgagtaaattagagtgttcaaagcaagcatccgctctgaatacattagcatggaataacacgataggactctggcctatcttgttggtctgta
ggaccggagtaatgattaagagggacagtcgggggcattcgtatttcattgtcagaggtgaaattcttggatttatgaaagacgaacttctgcgaaa
gcatttgccaaggatgttttcattaatcaagaacgaaagttgggggctcgaagacgattagataccgtcgtagtctcaaccataaacgatgccgact
agggattggcagatgtttcattgatgactctgccagcaccttatgagaaatcaaagtttttgggttccgggggggagtatggtcgcaaggctgaaact
taaaggaattgacggaagggcaccaccaggcgtggagcctgcggcttaatttgactcaacacggggaaacttaccaggtccagacacgggaa
ggattgacagattgagagctctttcttgattctgtgggtggtggtgcatggccgttcttagttggtgggttgccttgtcaggttgattccggtaacgaac
gagacctcagcctgctaaatagtcacgactgccggcagttggcagacttcttagagggactattgtcgtttaggcaatggaagtatgaggcaataa
caggtctgtgatgcccttagatgttctgggccgcacgcgcgctacactgacgcattcaacgagcctatccttggtcgagagacccgggtaatcttt
gaaactgcgtcgtgatggggatagattattgcaattattagtcttcaacgaggaatgcctagtaagcgcgagtcatcagctcgcgttgattacgtccc
tgccctttgtacacaccgcccgtcgctcctaccgattgggtgtgctggtgaagtgttcggattggcgctaggggatgaacatcaccttgtgccgag
aagttcattaaaccctcccacctagaggaaggagaagtcgtaacaaggtttccgtaggtgaacctgcggaaggatcattg
>AJ410472.1 Oogamochlamys zimbabwiensis partial 18S rRNA gene, strain UTEX 2214
ccatgcatgtctaagtatatatactgtgaaactccgaatggctcattaaatcagttatagtttatttgatggtaccctactcggataaccgtagtaattcta
gagctaatacgtgcgtaaatcccgacttctggaagggacgtatttattagataaaaggccagccgggctctgcccgactgcggtgaatcatgataa
cttcacgaatcgcatggcctcgtgccggcgatgtttcattcaaatttctgccctatcaactttcgatggtaggatagaggcctaccatggtggtaacg
ggtgacggaggattagggttcgattccggagagggagcctgagagatggctaccacatccaaggaaggcagcaggcgcgcaaattacccaat
cccgacacggggaggtagtgacaataaataacaataccgggcatgtctggtaattggaatgagtacaatctaaatcccttaacgaggatccattgg

agggcaagtctggtgccagcagccgcggtaattccagctccaatagcgtatatttaagttgttgcagttaaaaagctcgtagttggatttcggatgg
gtctcagcggtccgcctcgtgtgagtactgctgcggcctatctttctgccggggacggctcctgggcttaactgttcgggactcggagtcggcga
ggttactttgagtaaattagagtgttcaaagcaagcctacgctctgaatacattagcatggaataacacgataggactctggcctatcttgttggtctg
taggaccggagtaatgattaagagggacagtcgggggcattcgtatttcattgtcagaggtgaaattcttggatttatgaaagacgaacttctgcga
aagcatttgccaaggatgttttcattaatcaagaacgaaagttgggggctcgaagacgattagataccgtcgtagtctcaaccataaacgatgccg
actagggattggcagatgtttcattgatgactctgccagcaccttatgagaaatcaaagtttttgggttccggggggagtatggtcgcaaggctgaa
acttaaaggaattgacggaagggcaccaccaggcgtggagcctgcggcttaatttgactcaacacggggaaacttaccaggtccagacacggg
aaggattgacagattgagagctctttcttgattctgtgggtggtggtgcatggccgttcttagttggtgggttgccttgtcaggttgattccggtaacga
acgagacctcagcctgctaaatagtcacgactgccggcagttggcagacttcttagagggactattgtcgtttaggcaatggaagtatgaggcaat
aacaggtctgtgatgcccttagatgttctgggccgcacgcgcgctacactgacgcattcaacgagcctatccttggccgagaggcccgggtaatc
tttgaaactgcgtcgtgatggggatagattattgcaattattagtcttcaacgaggaatgcctagtaagcgcgagtcatcagctcgcgttgattacgtc
cctgccctttgtacacaccgcccgtcgctcctaccgattgggtgtgctggtgaagtgttcggattggcccttggagatgaacatcgacttgggctga
gaagttcattaaaccctcccacctagaggaaggagaagtcgtaacaaggtttccgtaggtgaacctgcggaaggatcattg
>AY271673.1 Palmellopsis sp. BCP-EM1VF1 18S ribosomal RNA gene, partial sequence
ccatgcatgtctaagtatatatactgtgaaactgcgaatggctcattaaatcagttatagtttatttgatggtactttactcggataaccgtagtaattcta
gagctaatacgtgcgtaaatcccgacttctggaagggacgtatttattagataaaaggccagccgggcttgcccgactcttggcgaatcatgataa
cttcacgaatcgcacggccttgtgccggcgatgtttcattcaaatttctgccctatcaactttcgatggtaggatagaggcctaccatggtggtaacg
ggtgacggaggattagggttcgattccggagagggagcctgagaaacggctaccacatccaaggaaggcagcaggcgcgcaaattacccaat
cccgacacggggaggtagtgacaataaataacaataccgggcc-
gtctggtaattggaatgagtacaatctaaatcccttaacgagtatccattggagggcaagtctggtgccagcagccgcggtaattccagctccaata
gcgtatatttaagttgttgcagttaaaaagctcgtagttggatttcgggtgggtgtcgccggtctgcctctggtatgtactggcgctgctcacctttctg
ccggggacggctcctgggcttaactgtctgggactcggagtcggcgttgttactttgagtaaattagagtgttcaaagcaagccttcgctctgaata
cattagcatggaataacacgataggactctggcctatcttgttggtctgtaggaccggagtaatgattaagagggacagtcgggggcattcgtattt
cattgtcagaggtgaaattcttggatttatgaaagacgaacttctgcgaaagcatttgccaaggatgttttcattgatcaagaacgaaagttgggggc
tcgaagacgattagataccgtcgtagtctcaaccataaacgatgccgactagggattggcaggtgtttcattgatgaccctgccagcaccttatgag
aaatcaaagtttttgggttccggggggagtatggtcgcaaggctgaaacttaaaggaattgacggaagggcaccaccaggcgtggagcctgcg
gcttaatttgactcaacacggggaaacttaccaggtccagacacggggaggattgacagattgagagctctttcttgattctgtgggtggtggtgca
tggccgttcttagttggtgggttgccttgtcaggttgattccggtaacgaacgagacctccgcctgctaaatagtcacgcgcaccggtgcacgcct
gacttcttagagggactaccggcgattagtcgttggaagtgggaggcaataacaggtctgtgatgcccttagatgttctgggccgcacgcgcgct
acactgatgcattcaacgagcctatccttggccgagaggtccgggtaatctttgaaactgcatcgtgatggggatagattattgcaattattagtcttc
aacgaggaatgcctagtaagcgcatgtcatcagcatgcgttgattacgtccctgccctttgtacacaccgcccgtcgctcctaccgattggatgtgc
tggtgaagtgttcggactggcttcaatgggggaacccctgctgttgccgggaagaacattaaaccctcccatctagaggaaggagaagtcgtaac
aaggtctccgtaggtgaacctgcggaaggatca---
>U22931.1 Polytoma anomale strain SAG 62-21 18S ribosomal RNA (Rrn18) gene, complete sequence
ccatgcatgtctaagtatatatactgtgaaactgcgaatggctcattaaatcagttatagtttatttgatggtaccttactcggataaccgtagtaattcta

gagctaatacgtgcgtaaatcccgacttctggaagggacgtatttattagataaaaggccagccgggcttgcccgaccttaggcgaatcatgataa
cttcacgaatcgcacggccttgtgccggcgatgtttcattcaaatttctgccctatcaactttcgatggtaggatagaggcctaccatggtggtaacg
ggtgacggaggattagggttcgattccggagagggagcctgagaaacggctaccacatccaaggaaggcagcaggcgcgcaaattacccaat
cccgacacggggaggtagtgacaataaataacaataccgggcatgtctggtaattggaatgagtacaatctaaatcccttaacgagtatccattgg
agggcaagtctggtgccagcagccgcggtaattccagctccaatagcgtatatttaagttgttgcagttaaaaagctcgtagttggatttcgggtgg
gttcaagcggtccgcctctggtgagtactgcttaggcctacctttctgccggggacggctcctgggattcatttctcgggactcggaatcggcgag
gttactttgagtaaattagagtgttcaaagcaagcctacgctctgaatacattagcatggaataacacgataggactctggcctatcttgttggtctgta
ggaccggagtaatgattaagagggacagtcgggggcattcgtatttcattgtcagaggtgaaattcttggatttatgaaagacgaacttctgcgaaa
gcatttgccaaggatgttttcattaatcaagaacgaaagttgggggctcgaagacgattagataccgtcgtagtctcaaccataaacgatgccgact
agggattggcaggtgtttcgttgatgaccctgccagcaccttatgagaaatcaaagtttttgggttccgggggagtatggtcgcaaggctgaaac
ttaaaggaattgacggaagggcaccaccaggcgtggagcctgcggcttaatttgactcaacacgggaaaacttaccaggtccagacacaggga
ggattgacagattgagagctctttcttgattctgtgggtggtggtgcatggccgttcttagttggtgggttgccttgtcaggttgattccggtaacgaac
gagacctcagcctgctaaatagtcacgtccaccggtggatgcctgacttcttagagggactattggcgtttagccaatggaagtgtgaggcaataa
caggtctgtgatgcccttagatgttctgggccgcacgcgcgctacactgacgcattcaacgagcctatccttggccgagaggtccgggtaatcttt
gaacctgcgtcgtgatggggatagattattgcaattattagtcttcaacgaggaatgcctagtaagcgcgagtcatcagctcgcgttgattacgtccc
tgccctttgtacacaccg-
ccgtcgctcctaccgattgggtgtgctggtgaagtgttcggattgacttcagttggggcaactcgactgttgttgagaagaacattaaaccctccca
cctagaggaaggagaagtcgtaacaaggtttccgta-gtgaacct---------------
>U22938.1 Polytoma sp. strain ATCC 30963 18S ribosomal RNA (Rrn18) gene, complete sequence
ccatgcatgtctaagtatatatactgtgaaactgcgaatggctcattaaatcagttacagtttatttgatggtaccttactcggataaccgtagtaattcta
gagctaatacgtgcgtaaatcccgacttctggaagggacgtatttattagataaaaggccagccgggcttgcccgaccttaggcgaatcatgataa
cttcacgaatcgcacggccttgtgccggcgatgtttcattcaaatttctgccctatcaactttcgatggtaggatagaggcctaccatggtggtaacg
ggtgacggaggattagggttcgattccggagagggagcctgagaaacggctaccacatccaaggaaggcagcaggcgcgcaaattacccaat
cccgacacggggaggtagtgacaataaataacaataccgggcatgtctggtaattggaatgagtacaatctaaatcccttaacgagtatccattgg
agggcaagtctggtgccagcagccgcggtaattccagctccaatagcgtatatttaagttgttgcagttaaaaagctcgtagttggatttcgggtgg
gttcaagcggtccgcctctggtgagtactgcttaggcctacctttctgccggggacggctcctgggattcatttctcgggactcggaatcggcgag
gttactttgagtaaattagagtgttcaaagcaagcctacgctctgaatacattagcatggaataacacgataggactctggcctatcttgttggtctgta
ggaccggagtaatgattaagagggacagtcgggggcattcgtatttcattgtcagaggtgaaattcttggatttatgaaagacgaacttctgcgaaa
gcatttgccaaggatgttttcattaatcaagaacgaaagttgggggctcgaagacgattagataccgtcgtagtctcaaccataaacgatgccgact
agggattggcaggtgtttcgttgatgaccctgccagcaccttatgagaaatcaaagtttttgggttccgggggagtatggtcgcaaggctgaaac
ttaaaggaattgacggaagggcaccaccaggcgtggagcctgcggcttaatttgactcaacacgggaaaacttaccaggtccagacacaggga
ggattgacagattgagagctctttcttgattctgtgggtggtggtgcatggccgttcttagttggtgggttgccttgtcaggttgattccggtaacgaac
gagacctcagcctgctaaatagtcacgtccaccggtggatgcctgacttcttagagggactattggcgtttagccaatggaagtgtgaggcaataa
caggtctgtgatgcccttagatgttctgggccgcacgcgcgctacactgacgcattcaacgagcctatccttggccgagaggtccgggtaatcttt
gaacctgcgtcgtgatggggatagattattgcaattattagtcttcaacgaggaatgcctagtaagcgcgagtcatcagctcgcgttgattacgtccc
tgccctttgtacacaccgcccgtcgctcctaccgattgggtgtgctggtgaagtgttcggattgacttcagttggggcaactcgactgttgttgagaa

gaacattaaaccctcccacctagaggaaggagaagtcgtaacaaggtttccgta-gtgaacc----------------

>KP299174.1 Polytomella capuana strain SAG 63-5 18S ribosomal RNA gene, partial sequence

ccatgcatgtctaagtatatatactgtgaaactgcgaatggctcattaaatcagttataatttatttgatggtaccctactcggataaccgtagtaattcta
gagctaatacgtgcgtaaatcccgacttctggaagggacgtatttattagataaaagaccagccggactcgtctgtctctcggtgactcatgataact
ttgcgaatcgctgatctt-----
cagcgatgtttcattcaaatttctgccctatcaactttcgatggtaggatagaggcctaccatggtggtaacgggtgacggaggattagggttcgatt
ccggagagggagcctgagagatggctaccacatccaaggaaggcagcaggcgcgcaaattacccaatcccgacacggggaggtagtgaca
ataaataacaataccgggcgcgtctggtaattggaatgagtacaatctaaatcccttaacgaggatccattggagggcaagtctggtgccagcag
ccgcggtaattccagctccaatagcgtatatttaagttgttgcagttaaaaagctcgtagttggatttcggatagtccggcgcggtcctctcttagtgc
gcactgcctcgcattatcttcctgccggggacggcttctgtcttcatgattagggaactcggagtcggcgtggttactttgagtaaattagagtgttca
aagcaagctttcgctctgaatacgttagcatggaataacgcgataggactctggcctat-
tcgttggtctgtgggactggagtaatgattaagagggacagtcgggggcattcgtattccgttgtcagaggtgaaattcttggatttacggaagacg
aacatctgcgaaagcatttgccaaggatgttttcattaatcaagaacgaaagttgggggctcgaagacgattagataccgtcgtagtctcaaccata
aacgatgccgactagggattggcggatggtcttttaatgcctccgccagcaccttatgagaaatcaaagtctttgggttccggggggagtatggtc
gcaaggctgaaacttaaaggaattgacggaagggcaccaccaggcgtggagcctgcggcttaatttgactcaacacgggaaaacttaccaggt
ccagacacgggaaggattgacagattgatagctctttcttgattctgtgggtggtggtgcatggccgttcttagttggtgggttgccttgtcaggttga
ttccggtaacgaacgagacctcagcctgctaaatagttctctccgccttggcggagctaacttcttagagggactattggcgtttagccaatggaag
tatgaggcaataacaggtctgtgatgcccttagatgttctgggccgcacgcgcgctacactgacgcgaccaacgagcctatcctttgccgagagg
cacgggtaatcttgtaaaccgcgtcgtgatggggatagatcattgcaattattggtcttcaacgaggaatgcctagtaagcgcgagtcatcagctcg
cgttgattacgtccctgccctttgtacacaccgcccgtcgctcctaccgattgggtgtgctggtgagatgtcgggattggctttagcttgggcaactc
aactagagctgagaacttcatcaaaccctcccacctagaggaaggagaagtcgtaacaaggtttccgtaggtgaacctgcggaaggatcattg

>U22932.1 Polytoma difficile strain SAG 62-16 18S ribosomal RNA (Rrn18) gene, complete sequence

ccatgcatgtctaagtatatatactgtgaaactgcgaatggctcattaaatcagttatagtttatttgatggtaccttactcggataaccgtagtaattcta
gagctaatacgtgcgtaaatcccgacttctggaagggacgtatttattagataaaaggccagccgggcttgcccgaccttaggcgaatcatgataa
cttcacgaatcgcacggccttgtgccggcgatgtttcattcaaatttctgccctatcaactttcgatggtaggatagaggcctaccatggtggtaacg
ggtgacggaggattagggttcgattccggagagggagcctgagaaacggctaccacatccaaggaaggcagcaggcgcgcaaattacccaat
cccgacacggggaggtagtgacaataaataacaataccgggcatgtctggtaattggaatgagtacaatctaaatcccttaacgagtatccattgg
agggcaagtctggtgccagcagccgcggtaattccagctccaatagcgtatatttaagttgttgcagttaaaaagctcgtagttggatttcgggtgg
gttcaagcggtccgcctctggtgagtactgcttaggcctacctttctgccggggacggctcctgggattcatttctcgggactcggaatcggcgag
gttactttgagtaaattagagtgttcaaagcaagcctacgctctgaatacattagcatggaataacacgataggactctggcctatcttgttggtctgta
ggaccggagtaatgattaagagggacagtcgggggcattcgtatttcattgtcagaggtgaaattcttggatttatgaaagacgaacttctgcgaaa
gcatttgccaaggatgttttcattaatcaagaacgaaagttgggggctcgaagacgattagataccgtcgtagtctcaaccataaacgatgccgact
agggattggcaggtgtttcgttgatgaccctgccagcaccttatgagaaatcaaagtttttgggttccggggggagtatggtcgcaaggctgaaac
ttaaaggaattgacggaagggcaccaccaggcgtggagcctgcggcttaatttgactcaacacgggaaaacttaccaggtccagacacaggga
ggattgacagattgagagctctttcttgattctgtgggtggtggtgcatggccgttcttagttggtgggttgccttgtcaggttgattccggtaacgaac

gagacctcagcctgctaaatagtcacgtccaccggtg-
atgcctgacttcttagagggactattggcgtttagccaatggaagtgtgaggcaataacaggtctgtgatgcccttagatgttctgggccgcacgcg
cgctacactgacgcattcaacgagcctatccttggccgagaggtccgggtaatctttgaacctgcgtcgtgatggggatagattattgcaattattag
tcttcaacgaggaatgcctagtaagcgcgagtcatcagctcgcgttgattacgtccctgccctttgtacacaccgcccgtcgctcctaccgattggg
tgtgctggtgaagtgttcggattgacttcagttggggcaactcgactgttgttgagaagaacattaaaccctcccacctagaggaaggagaagtcg
taacaaggtttccgta-gtgaacc----------------
>U22933.1 Polytoma ellipticum strain SAG 62-18 18S ribosomal RNA (Rrn18) gene, complete sequence
ccatgcatgtctaagtatatatactgtgaaactgcgaatggctcattaaatcagttatagtttatttgatggtaccttactcggataaccgtagtaattcta
gagctaatacgtgcgtaaatcccgacttctggaagggacgtatttattagataaaaggccagccgggcttgcccgaccttaggcgaatcatgataa
cttcacgaatcgcacggccttgtgccggcgatgtttcattcaaatttctgccctatcaactttcgatggtaggatagaggcctaccatggtggtaacg
ggtgacggaggattagggttcgattccggagagggagcctgagaaacggctaccacatccaaggaaggcagcaggcgcgcaaattacccaat
cccgacacggggaggtagtgacaataaataacaataccgggcatgtctggtaattggaatgagtacaatctaaatcccttaacgagtatccattgg
agggcaagtctggtgccagcagccgcggtaattccagctccaatagcgtatatttaagttgttgcagttaaaaagctcgtagttggatttcgggtgg
gttcaagcggtccgcctctggtgagtactgcttaggcctacctttctgccggggacggctcctgggattcatttctcgggactcggaatcggcgag
gttactttgagtaaattagagtgttcaaagcaagcctacgctctgaatacattagcatggaataacacgataggactctggcctatcttgttggtctgta
ggaccggagtaatgattaagagggacagtcgggggcattcgtatttcattgtcagaggtgaaattcttggatttatgaaagacgaacttctgcgaaa
gcatttgccaaggatgttttcattaatcaagaacgaaagttgggggctcgaagacgattagataccgtcgtagtctcaaccataaacgatgccgact
agggattggcaggtgtttcgttgatgaccctgccagcaccttatgagaaatcaaagtttttgggttccgggggggagtatggtcgcaaggctgaaac
ttaaaggaattgacggaagggcaccaccaggcgtggagcctgcggcttaatttgactcaacacgggaaaacttaccaggtccagacacaggga
ggattgacagattgagagctctttcttgattctgtgggtggtggtgcatggccgttcttagttggtgggttgccttgtcaggttgattccggtaacgaac
gagacctcagcctgctaaatagtcacgtccaccggtggatgcctgacttcttagagggactattggcgtttagccaatggaagtgtgaggcaataa
caggtctgtgatgcccttagatgttctgggccgcacgcgcgctacactgacgcattcaacgagcctatccttggccgagaggtccgggtaatcttt
gaacctgcgtcgtgatggggatagattattgcaattattagtcttcaacgaggaatgcctagtaagcgcgagtcatcagctcgcgttgattacgtccc
tgccctttgtacacaccgcccgtcgctcctaccgattgggtgtgctggtgaagtgttcggattgacttcagttggggcaactcgactgttgttgagaa
gaacattaaaccctcccacctagaggaaggagaagtcgtaacaaggtttccgtaggtgaacc----------------
>KP299175.1 Polytomella magna strain SAG 63-9 18S ribosomal RNA gene, partial sequence
ccatgcatgtctaagtatatatactgtgaaactgcgaatggctcattaaatcagttataatttatttgatgataccctactcggataaccgtagtaattcta
gagctaatacgtgcgtaaatcccgacttctggaagggacgtatttattagataaaaggccagccggacttgtccgaccagcggtgactcatgataa
ctttgcgaatcgctgcttgc------
agcgatgtttcattcaaatttctgccctatcaactttcgatggtaggatagaggcctaccatggtggtaacgggtgacggaggattagggttcgattc
cggagagggagcctgagagatggctaccacatccaaggaaggcagcaggcgcgcaaattacccaatcccgacacggggaggtagtgacaat
aaataacaataccgggcgcgtctggtaattggaatgagtacaatctaaatcccttaacgaggatccattggagggcaagtctggtgccagcagcc
gcggtaattccagctccaatagcgtatatttaagttgttgcagttaaaaagctcgtagttggatttcgggtagttcgtcgcggtcttctcttagaacgca
ctgcctcgcgctatcttcctgccggggacggttcctgtgctttcgggtatgggactcggagtcggcgtggttactttgagtaaattagagtgttcaaa
gcaagctttcgctctgaatacgttagcatggaataacgcgataggactctggcctat-

tcgttggtctgtgggactggagtaatgattaagagggacagtcgggggcattcgtattccgttgtcagaggtgaaattcttggatttacggaagacg
aacatctgcgaaagcatttgccaaggatgttttcattaatcaagaacgaaagttgggggctcgaagacgattagataccgtcgtagtctcaaccata
aacgatgccgactagggattggccgatggttatttaatgcctcggccagcaccttatgagaaatcaaagtctttgggttccgggggggagtatggtc
gcaaggctgaaacttaaaggaattgacggaagggcaccaccaggcgtggagcctgcggcttaatttgacccaacacgggaaaacttaccaggt
ccagacacgggaaggattgacagattgatagctctttcttgattctgtgggtggtggtgcatggccgttcttagttggtgggttgccttgtcaggttga
ttccggtaacgaacgagacctcagcctgctaaatagtctgcatcgctgcggtgtacttgacttcttagagggactattggcgtttagccaatggaagt
atgaggcgataacaggtctgtgatgcccttagatgttctgggccgcacgcgcgctacactgacgcgaccaacgagcctatcctttaccgagaggt
acgggtaatcttgtaaaccgcgtcgtgatggggatagatcattgcaattattggtcttcaacgaggaatgcctagtaagcgcgagtcatcagctcgc
gttgattacgtccctgccctttgtacacaccgcccgtcgctcctaccgattgggtgtgctggtgagatgtcgggatcagctctaggtgaagcaattc
ctctagagccgagaacttcatcaaaccctcccacctagaggaaggagaagtcgtaacaaggtttccgtaggtgaacctgcggaaggatcattg
>U22934.1 Polytoma mirum strain SAG 62-13 18S ribosomal RNA (Rrn18) gene, complete sequence
ccatgcatgtctaagtatatatactgtgaaactgcgaatggctcattaaatcagttatagtttatttgatggtaccttactcggataaccgtagtaattcta
gagctaatacgtgcgtaaatcccgacttctggaagggacgtatttattagataaaaggccagccgggcttgcccgaccttaggcgaatcatgataa
cttcacgaatcgcacggccttgtgccggcgatgtttcattcaaatttctgccctatcaactttcgatggtaggatagaggcctaccatggtggtaacg
ggtgacggaggattagggttcgattccggagagggagcctgagaaacggctaccacatccaaggaaggcagcaggcgcgcaaattacccaat
cccgacacggggaggtagtgacaataaataacaataccgggcatgtctggtaattggaatgagtacaatctaaatcccttaacgagtatccattgg
agggcaagtctggtgccagcagccgcggtaattccagctccaatagcgtatatttaagttgttgcagttaaaaagctcgtagttggatttcgggtgg
gttcaagcggtccgcctctggtgagtactgcttaggcctacctttctgccggggacggctcctgggattcatttctcgggactcggaatcggcgag
gttactttgagtaaattagagtgttcaaagcaagcctacgctctgaatacattagcatggaataacacgataggactctggcctatcttgttggtctgta
ggaccggagtaatgattaagagggacagtcgggggcattcgtatttcattgtcagaggtgaaattcttggatttatgaaagacgaacttctgcgaaa
gcatttgccaaggatgttttcattaatcaagaacgaaagttgggggctcgaagacgattagataccgtcgtagtctcaaccataaacgatgccgact
agggattggcaggtgtttcgttgatgaccctgccagcaccttatgagaaatcaaagtttttgggttccgggggggagtatggtcgcaaggctgaaac
ttaaaggaattgacggaagggcaccaccaggcgtggagcctgcggcttaatttgactcaacacgggaaaacttaccaggtccagacacaggga
ggattgacagattgagagctctttcttgattctgtgggtggtggtgcatggccgttcttagttggtgggttgccttgtcaggttgattccggtaacgaac
gagacctcagcctgctaaatagtcacgtccaccggtggatgcctgacttcttagagggactattggcgtttagccaatggaagtgtgaggcaataa
caggtctgtgatgcccttagatgttctgggccgcacgcgcgctacactgacgcattcaacgagcctatccttggccgagaggtccgggtaatcttt
gaacctgcgtcgtgatggggatagattattgcaattattagtcttcaacgaggaatgcctagtaagcgcgagtcatcagctcgcgttgattacgtccc
tgccctttgtacacaccgcccgtcgctcctaccgattgggtgtgctggtgaagtgttcggattgacttcagttggggcaactcgactgttgttgagaa
gaacattaaaccctcccacctagaggaaggagaagtcgtaacaaggtttccgtaggtgaacc----------------
>U22935.1 Polytoma obtusum 18S ribosomal RNA (Rrn18) gene, complete sequence
ccatgcatgtctaagtatatatactgtgaaactgcgaatggctcattaaatcagttatagtttatttgatggtaccttactcggataaccgtagtaattcta
gagctaatacgtgcgtaaatcccgacttctggaagggacgtatttattagataaaaggccagccgggcttgcccgaccttaggcgaatcatgataa
cttcacgaatcgcacggccttgtgccggcgatgtttcattcaaatttctgccctatcaactttcgatggtaggatagaggcctaccatggtggtaacg
ggtgacggaggattagggttcgattccggagagggagcctgagaaacggctaccacatccaaggaaggcagcaggcgcgcaaattacccaat
cccgacacggggaggtagtgacaataaataacaataccgggcatgtctggtaattggaatgagtacaatctaaatcccttaacgagtatccattgg
agggcaagtctggtgccagcagccgcggtaattccagctccaatagcgtatatttaagttgttgcagttaaaaagctcgtagttggatttcgggtgg

gttcaagcggtccgcctctggtgagtactgcttaggcctacctttctgccggggacggctcctgggattcatttctcgggactcggaatcggcgag
gttactttgagtaaattagagtgttcaaagcaagcctacgctctgaatacattagcatggaataacacgataggactctggcctatcttgttggtctgta
ggaccggagtaatgattaagagggacagtcgggggcattcgtatttcattgtcagaggtgaaattcttggatttatgaaagacgaacttctgcgaaa
gcatttgccaaggatgttttcattaatcaagaacgaaagttgggggctcgaagacgattagataccgtcgtagtctcaaccataaacgatgccgact
agggattggcaggtgtttcgttgatgaccctgccagcaccttatgagaaatcaaagtttttgggttccgggggagtatggtcgcaaggctgaaac
ttaaaggaattgacggaagggcaccaccaggcgtggagcctgcggcttaatttgactcaacacgggaaaacttaccaggtccagacacaggga
ggattgacagattgagagctctttcttgattctgtgggtggtggtgcatggccgttcttagttggtgggttgccttgtcaggttgattccggtaacgaac
gagacctcagcctgctaaatagtcacgtccaccggtggatgcctgacttcttagagggactattggcgtttagccaatggaagtgtgaggcaataa
caggtctgtgatgcccttagatgttctgggccgcacgcgcgctacactgacgcattcaacgagcctatccttggccgagaggtccgggtaatcttt
gaacctgcgtcgtgatggggatagattattgcaattattagtcttcaacgaggaatgcctagtaagcgcgagtcatcagctcgcgttgattacgtccc
tg-cctttgtacacaccg-
ccgtcgctcctaccgattgggtgtgctggtgaagtgttcggattgacttcagttggggcaactcgactgttgttgagaagaacattaaaccctccca
cctagaggaaggagaagtcgtaacaaggtttccgtagtgaaccaagcaggaaggatca--
>HQ584989.1 Polytomella papillata strain SAG 63-2 18S ribosomal RNA gene, partial sequence
----
---gattcatctgtcctttggtgattcataataactttacgaatcgctgttttt----
tcagcgatgtttcattcaaatttctgccctatcaactttcgatggtaggatagaggcctaccatggtggtaacgggtgacggagaattagggttcgatt
ccggagagggagcctgagagatggctaccacatccaaggaaggcagcaggcgcgcaaattacccaatcccaacacggggaggtagtgaca
ataaataacaataccgggcgcgtctggtaattggaatgagtacaatctaaatcccttaacgaggatccattggagggcaagtctggtgccagcag
ccgcggtaattccagctccaatagcgtatatttaagttgttgcagttaaaaagctcgtagttggatttcggatgatttatcgcggtccttatttttgcgta
ctgccttgaattatctttcttttgttggcaggggtaggactcgtcttgct--
ccttcaaattcaacgtggttactttgagtaaattagagtgttcaaagcaagctatcgctctgaatacgttagcatggaataacgcgataggactctgg
cctat-
tcgttggtctgtgggactggagtaatgattaagagggacagtcgggggcatttgtattccgttgtcagaggtgaaattcttggatttacggaagaca
aacatctgcgaaagcatttgccaaggatgttttcattaatcaagaacgaaagttgggggctcgaagacgattagataccgtcgtagtctcaaccata
aacgatgccgactagggattggcagatggtttttaatgcctctgccagcaccttatgagaaatcaaagtctttgggttccgggggagtatggtcg
caaggctgaaacttaaaggaattgacggaagggcaccaccaggcgtggagcctgcggcttaatttgactcaacacgggaaaacttaccaggtc
cagacacgggaaggat--

>KP299173.1 Polytomella parva strain SAG 63-3 clone 7.2.4 18S ribosomal RNA gene, partial sequence
ccatgcatgtctaagtatatatactgtgaaactgcgaatggctcattaaatcagttataatttatttgatgataccctacttggataaccgtagtaattcta
gagctaatacatgcgtaaatcccgacttctggaagggacgtatttattagataaaagaccatccggattcatctgtcctttggtgattcataataacttt

acgaatcgctgttttt----
tcagcgatgtttcattcaaatttctgccctatcaactttcgatggtaggatagaggcctaccatggtggtaacgggtgacggagaattagggttcgatt
ccggagagggagcctgagagatggctaccacatccaaggaaggcagcaggcgcgcaaattacccaatcccaacacggggaggtagtgaca
ataaataacaataccgggcgcgtctggtaattggaatgagtacaatctaaatcccttaacgaggatccattggagggcaagtctggtgccagcag
ccgcggtaattccagctccaatagcgtatatttaagttgttgcagttaaaaagctcgtagttggatttcggatgatttatcgcggtccttattttttgcgta
ctgccttgaattatctttcttttgttggcaggggtaggactcgtcttgct--
ccttcaaattcaacgtggttactttgagtaaattagagtgttcaaagcaagctatcgctctgaatacgttagcatggaataacgcgataggactctgg
cctat-
tcgttggtctgtgggactggagtaatgattaagagggacagtcgggggcatttgtattccgttgtcagaggtgaaattcttggatttacggaagaca
aacatctgcgaaagcatttgccaaggatgttttcattaatcaagaacgaaagttgggggctcgaagacgattagataccgtcgtagtctcaaccata
aacgatgccgactagggattggcagatggtttttaatgcctctgccagcaccttatgagaaatcaaagtctttgggttccgggggagtatggtcg
caaggctgaaacttaaaggaattgacggaagggcaccaccaggcgtggagcctgcggcttaatttgacccaacacgggaaaacttaccaggtc
cagacacgggaaggattgacagattgatagctctttcttgattctgtgggtggtggtgcatggccgttcttagttggtgggttgccttgtcaggttgatt
ccggtaacgaacgagacctcagcctgctaaatagtttgctttacctattggtagagaacttcttagagggactattggcgtttagccaatggaaggat
gaggcaataacaggtctgtgatgcccttagatgttctgggccgcacgcgcgctacactgacgcaaccaacgagtttatcctttaccgagaggtac
gggtaatcttgtaaactgcgtcgtgatggggatagatcattgcaattattggtcttcaacgaggaatgcctagtaagcgcgagtcatcagctcgcgtt
gattacgtccctgccctttgtacacaccgcccgtcgctcctaccgattgggtgtgctggtgagatgttgggattggtttcaagttaggaacttgatttg
agactgaaaacttcatcaaaccctcccacctagaggaaggagaagtcgtaacaaggtttccgtaggtgaacctgcggaaggatcattg
>KP299172.1 Polytomella piriformis strain SAG 63-10 18S ribosomal RNA gene, partial sequence
ccatgcatgtctaagtatatatactgtgaaactgcgaatggctcattaaatcagttataatttatttgatgataccctacttggataaccgtagtaattcta
gagctaatacatgcgtaaatcccgacttctggaagggacgtatttattagataaaagaccatccagattcatctgtcctttggtgattcataataacttt
acgaatcgctgttttt----
tcagcgatgtttcattcaaatttctgccctatcaactttcgatggtaggatagaggcctaccatggtggtaacgggtgacggagaattagggttcgatt
ccggagagggagcctgagagatggctaccacatccaaggaaggcagcaggcgcgcaaattacccaatcccaacacggggaggtagtgaca
ataaataacaataccgggcgcgtctggtaattggaatgagtacaatctaaatcccttaacgaggatccattggagggcaagtctggtgccagcag
ccgcggtaattccagctccaatagcgtatatttaagttgttgcagttaaaaagctcgtagttggatttcggatgatttatcgcggtccttattttatgcgta
ctgccttgaattatcttctttttgttggcaggaggtaggtttattcttactttcttcaaattcaacgtggttactttgagtaaattagagtgttcaaagcaagc
tatcgctctgaatacgttagcatggaataacgcgataggactctggcctat-
tcgttggtctgtgggactggagtaatgattaagagggacagtcgggggcatttgtattccgttgtcagaggtgaaattcttggatttacggaagaca
aacatctgcgaaagcatttgccaaggatgttttcattaatcaagaacgaaagttgggggctcgaagacgattagataccgtcgtagtctcaaccata
aacgatgccgactagggattggcagatggtttttaatgcctctgccagcaccttatgagaaatcaaagtctttgggttccgggggagtatggtcg
caaggctgaaacttaaaggaattgacggaagggcaccaccaggcgtggagcctgcggcttaatttgactcaacacgggaaaacttaccaggtc
cagacacgggaaggattgacagattgatagctctttcttgattctgtgggtggtggtgcatggccgttcttagttggtgggttgccttgtcaggttgatt
ccggtaacgaacgagacctcagcctgctaaatagtttgctttacctattggtagagaacttcttagagggactattggcgtttagccaatggaaggat
gaggcaataacaggtctgtgatgcccttagatgttctgggccgcacgcgcgctacactgacgcaaccaacgagtttatcctttaccgagaggtac
gggtaatcttgtaaactgcgtcgtgatggggatagatcattgcaattattggtcttcaacgaggaatgcctagtaagcgcgagtcatcagctcgcgtt

gattacgtccctgccctttgtacacaccgcccgtcgctcctaccgattgggtgtgctggtgagatgttgggattggtatcaagttggcaacctgactt
gaaactgaaaacttcatcaaaccctcccacctagaggaaggagaagtcgtaacaaggtttccgtaggtgaacctgcggaaggatcattg
>U22937.1 Polytoma sp. SAG 195.80 18S ribosomal RNA (Rrn18) gene, complete sequence
ccatgcatgtctaagtatatatactgtgaaactgcgaatggctcattaaatcagttatagtttatttgatggtaccttactcggataaccgtagtaattcta
gagctaatacgtgcgtaaatcccgacttctggaagggacgtatttattagataaaaggccagccgggcttgcccgaccttaggcgaatcatgataa
cttcacgaatcgcacggccttgtgccggcgatgtttcattcaaatttctgccctatcaactttcgatggtaggatagaggcctaccatggtggtaacg
ggtgacggaggattagggttcgattccggagagggagcctgagaaacggctaccacatccaaggaaggcagcaggcgcgcaaattacccaat
cccgacacggggaggtagtgacaataaataacaataccgggcatgtctggtaattggaatgagtacaatctaaatcccttaacgagtatccattgg
agggcaagtctggtgccagcagccgcggtaattccagctccaatagcgtatatttaagttgttgcagttaaaaagctcgtagttggatttcgggtgg
gttcaagcggtccgcctctggtgagtactgcttaggcctacctttctgccggggacggctcctgggattcatttctcgggactcggaatcggcgag
gttactttgagtaaattagagtgttcaaagcaagcctacgctctgaatacattagcatggaataacacgataggactctggcctatcttgttggtctgta
ggaccggagtaatgattaagagggacagtcgggggcattcgtatttcattgtcagaggtgaaattcttggatttatgaaagacgaacttctgcgaaa
gcatttgccaaggatgttttcattaatcaagaacgaaagttgggggctcgaagacgattagataccgtcgtagtctcaaccataaacgatgccgact
agggattggcaggtgtttcgttgatgaccctgccagcaccttatgagaaatcaaagtttttgggttccgggggagtatggtcgcaaggctgaaac
ttaaaggaattgacggaagggcaccaccaggcgtggagcctgcggcttaatttgactcaacacgggaaaacttaccaggtccagacacaggga
ggattgacagattgagagctctttcttgattctgtgggtggtggtgcatggccgttcttagttggtgggttgccttgtcaggttgattccggtaacgaac
gagacctcagcctgctaaatagtcacgtccaccggtggatgcctgacttcttagagggactattggcgtttagccaatggaagtgtgaggcaataa
caggtctgtgatgcccttagatgttctgggccgcacgcgcgctacactgacgcattcaacgagcctatccttggccgagaggtccgggtaatcttt
gaacctgcgtcgtgatggggatagattattgcaattattagtcttcaacgaggaatgcctagtaagcgcgagtcatcagctcgcgttgattacgtccc
tgccctttgtacacaccg-
ccgtcgctcctaccgattgggtgtgctggtgaagtgttcggattgacttcagttggggcaactcgactgttgttgagaagaacattaaaccctccca
cctagtggaaggagaagtcgtaacaaggtttccgtaggtgaacctg--------------
>U22942.1 Polytoma uvella strain SAG 62-2M 18S ribosomal RNA (Rrn18) gene, complete sequence
ccatgcatgtctaagtatatatactgtgaaactgcgaatggctcattaaatcagttatagtttatttgatggtaccttactcggataaccgtagtaattcta
gagctaatacgtgcgtaaatcccgacttctggaagggacgtatttattagataaaaggccagccgggcttgcccgaccttaggcgaatcatgataa
cttcacgaatcgcacggccttgtgccggcgatgtttcattcaaatttctgccctatcaactttcgatggtaggatagaggcctaccatggtggtaacg
ggtgacggaggattagggttcgattccggagagggagcctgagaaacggctaccacatccaaggaaggcagcaggcgcgcaaattacccaat
cccgacacggggaggtagtgacaataaataacaataccgggcatgtctggtaattggaatgagtacaatctaaatcccttaacgagtatccattgg
agggcaagtctggtgccagcagccgcggtaattccagctccaatagcgtatatttaagttgttggagttaaaaagctcgtagttggatttcgggtgg
gttcaagcggtccgcctctggtgagtactgcttaggcctacctttctgccggggacggctcctgggattcatttctcgggactcggaatcggcgag
gttactttgagtaaattagagtgttcaaagcaagcctacgctctgaatacattagcatggaataacacgataggactctggcctatcttgttggtctgta
ggaccggagtaatgattaagagggacagtcgggggcattcgtatttcattgtcagaggtgaaattcttggatttatgaaagacgaacttctgcgaaa
gcatttgccaaggatgttttcattaatcaagaacgaaagttgggggctcgaagacgattagataccgtcgtagtctcaaccataaacgatgccgact
agggattggcaggtgtttcgttgatgaccctgccagcaccttatgagaaatcaaagtttttgggttccgggggagtatggtcgcaaggctgaaac
ttaaaggaattgaccgaagggcaccaccaggcgtggagcctgcggcttaatttgactcaacacgggaaaacttaccaggtccagacacaggga
ggattgacagattgagagctctttcttgattctgtgggtggtggtgcatggccgttcttagttggtgggttgccttgtcaggttgattccggtaacgaac

gagacctcagcctgctaaatagtcacgtccaccggtggatgcctgacttcttagagggactattggcgtttagccaatggaagtgtgaggcaataa
caggtctgtgatgcccttagatgttctgggccgcacgcgcgctacactgacgcattcaacgagcctatccttggccgagaggtccgggtaatcttt
gaacctgcgtcgtgatggggatagattattgcaattattagtcttcaacgaggaatgcctagtaagcgcgagtcatcagctcgcgttgattacgtccc
tgccctttgtacacaccg-
ccgtcgctcctaccgattgggtgtgctggtgaagtgttcggattgacttcagttggggcaactcgactgttgttgagaagaacattaaaccctccca
cctagaggaaggagaagtcgtaacaaggtttccgtaggtgaacctgcaggaaggatca--
>LC093465.1 Pyrobotrys casinoensis gene for 18S ribosomal RNA, partial sequence
---
tgcatgtctaagtatatatactgtgaaactgcgaatggctcattaaatcagttatagtttatttgatggtaccttactcggataaccgtagtaattctagag
ctaatacgtgcgtaaatcccgacttctggaagggacgtatttattagataaaaggccagccgagcttgctcgaccccaggcgaatcatgataacttc
acgaatcgcacggccttgtgccggcgatgtttcattcaaatttctgccctatcaactttcgatggtaggatagaggcctaccatggtggtaacgggta
acggaggattagggttcgattccggagagggagcctgagaaacggctaccacatccaaggaaggcagcaggcgcgcaaattacccaatcccg
acacggggaggtagtgacaataaataacaataccgggcatgtctggtaattggaatgagtacaatctaaatcccttaacgagtatccattggaggg
caagtctggtgccagcagccgcggtaattccagctccaatagcgtatatttaagttgttgcagttaaaaagctcgtagttggatttcgggtgggttcta
gcggtccggctctgctgtgtactgctaaggctcacctttctgccggggacggctcctgggcttcactgtccgggactcggaatcggcgaggttact
ttgagtaaatcaaggcgttcaaagcaggcatacgctctgaatgcattagcatggaataacacgataggactctggcctatcttgttggtctgtagga
ccggagtaatgattaagagggacagtcgggggcattcgtatttcattgtcagaggtgaaattcttggatttatgaaagacgaacttctgcgaaagca
tttgccaaggatgttttcattaatcaagaacgaaagttgggggctcgaagacgattagataccgtcgtagtctcaaccataaacgatgccgactagg
gattggcaggtgtttcgttgatgaccctgccagcaccttatgagaaatcaaagtttttgggttccggggggagtatggtcgcaaggctgaaacttaa
aggaattgacggaagggcaccaccaggcgtggagcctgcggcttaatttgactcaacacgggaaaacttaccaggtccagacacggggagga
ttgacagattgagagctctttcttgattctgtgggtggtggtgcatggccgttcttagttggtgggttgccttgtcaggttgattccggtaacgaacgag
acctcagcctgctaaatagtcacgtctaccggtaggcgcctgacttcttagagggactattggcgtttagccaatggaagtgtgaggcgataacag
gtctgtgatgcccttagatgttctgggccgcacgcgcgctacactgatgcattcaacgagcctatccttggccgagaggtccgggtaatctttgaaa
ctgcatcgtgatggggatagattattgcaattattagtcttcaacgaggaatgcctagtaagcgcgagtcatcagctcgcgttgattacgtccctgcc
ctttgtacacaccgcccgtcgctcctaccgattgggtgtgctggtgaagtgttcggatcgactccagcggtggaacctctgctgttgttgagaagat
cattaaaccctcccacctagaggaaggagaagtcg--------------------------------------
>KM020019.1 Tetracystis excentrica strain SAG 92.80 18S ribosomal RNA gene, partial sequence; internal transcribed spacer 1, 5.8S ribosomal RNA gene, and internal transcribed spacer 2, complete sequence; and 26S ribosomal RNA gene, partial sequence
ccatgcatgtctaagtatatatactgtgaaactgcgaatggctcattaaatcagttatagtttatttgatggtaccttactcggataaccgtagtaattcta
gagctaatacgtgcgtaaatcccgacttctggaagggacgtatttattagataaaaggccagccgggcttgcccgactcttggcgaatcatgataa
cttcacgaatcgcacggcttcacgccggcgatgtttcattcaaatttctgccctatcaactttcgatggtaggatagaggcctaccatggtggtaacg
ggtgacggaggattagggttcgattccggagagggagcctgagaaacggctaccacatccaaggaaggcagcaggcgcgcaaattacccaat
cccgacacggggaggtagtgacaataaataacaataccgggcatgtctggtaattggaatgagtacaatctaaatcccttaacgagtatccattgg
agggcaagtctggtgccagcagccgcggtaattccagctccaatagcgtatatttaagttgttgcagttaaaaagctcgtagttggatttcgggtgg
gttctagcggtccgcctctggtgagtactgctatggcctacctttctgtcggggacggtctctgggcttcactgtccggagctcggaatcgacgag

gttactttgagtaaattagagtgttcaaagcaagcctacgctctgaatacattagcatggaataacacgataggactctggcctatcttgttggtctgta
ggaccggagtaatgattaagagggacagtcgggggcattcgtatttcattgtcagaggtgaaattcttggatttatgaaagacgaacatctgcgaa
agcatttgccaaggatgttttcattaatcaagaacgaaagttgggggctcgaagacgattagataccgtcgtagtctcaaccataaacgatgccga
ctagggattggcaggtgtttcgttgatgaccctgccagcaccttatgagaaatcaaagtttttgggttccgggggagtatggtcgcaaggctgaa
acttaaaggaattgacggaagggcaccaccaggcgtggagcctgcggcttaatttgactcaacacgggaaaacttaccaggtccagacacggg
gaggattgacagattgagagctctttcttgattctgtgggtggtggtgcatggccgttcttagttggtgggttgccttgtcaggttgattccggtaacg
aacgagacctcagcctgctaaatagtcacggctaccggtagtcgcttgacttcttagagggactattggcgtttagccaatggaagtgtgaggcaa
taacaggtctgtgatgcccttagatgttctgggccgcacgcgcgctacactgatgcattcaacgagcctatccttggccgagaggtccgggtaatc
ttcgaaactgcatcgtgatggggctagatcattgcaattattggtcttcaacgaggaatgcctagtaagcgcgagtcatcagctcgcgttgattacgt
ccctgccctttgtacacaccgcccgtcgctcctaccgattgggtgtgctggtgaagtgtccggattgactacagcgggggaacctctgctgttgttg
aaaaggtcattaaaccctcccacctagaggaaggagaagtcgtaacaaggtttccgtaggtgaacctgcggaaggatcattg

>KM020021.1 Tetracystis intermedia strain SAG 94.80 18S ribosomal RNA gene, partial sequence; internal transcribed spacer 1, 5.8S ribosomal RNA gene, and internal transcribed spacer 2, complete sequence; and 26S ribosomal RNA gene, partial sequence

ccatgcatgtctaagtatatatactgtgaaactgcgaatggctcattaaatcagttatagtttatttgatggtaccttactcggataaccgtagtaattcta
gagctaatacgtgcgtaaatcccgacttctggaagggacgtatttattagataaaaggccagccgggcttgcccgactcatggcgaatcatgataa
cttcacgaatcgcacggcttcatgccggcgatgtttcattcaaatttctgccctatcaactttcgatggtaggatagaggcctaccatggtggtaacg
ggtgacggaggattagggttcgattccggagagggagcctgagaaacggctaccacatccaaggaaggcagcaggcgcgcaaattacccaat
cccgacacggggaggtagtgacaataaataacaataccgggcatgtctggtaattggaatgagtacaatctaaatcccttaacgagtatccattgg
agggcaagtctggtgccagcagccgcggtaattccagctccaatagcgtatatttaagttgttgcagttaaaaagctcgtagttggatttcggatgg
gttctagcggtccgcctctggtgagtactgctatggcctatctttctgccggggacggtctctgggcttaactgtccggagtccggaatcggcgag
gttactttgagtaaattagagtgttcaaagcaagcctacgctctgaatacattagcatggaataacacgataggactctggcctatcttgttggtctgta
ggaccggagtaatgattaagagggacagtcgggggcattcgtatttcattgtcagaggtgaaattcttggatttatgaaagacgaacatctgcgaa
agcatttgccaaggatgttttcattaatcaagaacgaaagttgggggctcgaagacgattagataccgtcgtagtctcaaccataaacgatgccga
ctagggattggcaggtgtttcgttgatgaccctgccagcaccttatgagaaatcaaagtttttgggttccgggggagtatggtcgcaaggctgaa
acttaaaggaattgacggaagggcaccaccaggcgtggagcctgcggcttaatttgactcaacacgggaaaacttaccaggtccagacacggg
gaggattgacagattgagagctctttcttgattctgtgggtggtggtgcatggccgttcttagttggtgggttgccttgtcaggttgattccggtaacg
aacgagacctcagcctgctaaatagtcacggctaccggtagtcgcttgacttcttagagggactattggcgtttagccaatggaagtgtgaggcaa
taacaggtctgtgatgcccttagatgttctgggccgcacgcgcgctacactgatgcattcaacgagcctatccttggccgagaggtccgggtaatc
ttcgaaactgcatcgtgatggggctagatcattgcaattattggtcttcaacgaggaatgcctagtaagcgcgagtcatcagctcgcgttgattacgt
ccctgccctttgtacacaccgcccgtcgctcctaccgattgggtgtgctggtgaagtgtccggattggctccagcgggggaacctctgctgttgct
gagaaggtcattaaaccctcccacctagaggaaggagaagtcgtaacaaggtttccgtaggtgaacctgcggaaggatcattg

>KM020022.1 Tetracystis pulchra strain SAG 97.80 18S ribosomal RNA gene, partial sequence; internal transcribed spacer 1, 5.8S ribosomal RNA gene, and internal transcribed spacer 2, complete sequence; and 26S ribosomal RNA gene, partial sequence

ccatgcatgtctaagtatatatactgtgaaactgcgaatggctcattaaatcagttatagtttatttgatggtaccttactcggataaccgtagtaattcta

gagctaatacgtgcgtaaatcccgacttctggaagggacgtatttattagataaaaggccagccgggcttgcccgactcatggcgaatcatgataa
cttcacgaatcgcacggcttcatgccggcgatgtttcattcaaatttctgccctatcaactttcgatggtaggatagaggcctaccatggtggtaacg
ggtgacggaggattagggttcgattccggagagggagcctgagaaacggctaccacatccaaggaaggcagcaggcgcgcaaattacccaat
cccgacacggggaggtagtgacaataaataacaataccgggcatgtctggtaattggaatgagtacaatctaaatcccttaacgagtatccattgg
agggcaagtctggtgccagcagccgcggtaattccagctccaatagcgtatatttaagttgttgcagttaaaaagctcgtagttggatttcggatgg
gttctagcggtccgcctctggtgagtactgctatggcctatctttctgccggggacggtctctgggcttaactgtccggagtccggaatcggcgag
gttactttgagtaaattagagtgttcaaagcaagcctacgctctgaatacattagcatggaataacacgataggactctggcctatcttgttggtctgta
ggaccggagtaatgattaagagggacagtcgggggcattcgtatttcattgtcagaggtgaaattcttggatttatgaaagacgaacatctgcgaa
agcatttgccaaggatgttttcattaatcaagaacgaaagttgggggctcgaagacgattagataccgtcgtagtctcaaccataaacgatgccga
ctagggattggcaggtgtttcgttgatgaccctgccagcaccttatgagaaatcaaagtttttgggttccgggggggagtatggtcgcaaggctgaa
acttaaaggaattgacggaagggcaccaccaggcgtggagcctgcggcttaatttgactcaacacgggaaaacttaccaggtccagacacggg
gaggattgacagattgagagctctttcttgattctgtgggtggtggtgcatggccgttcttagttggtgggttgccttgtcaggttgattccggtaacg
aacgagacctcagcctgctaaatagtcacggctaccggtagtcgcttgacttcttagagggactattggcgtttagccaatggaagtgtgaggcaa
taacaggtctgtgatgcccttagatgttctgggccgcacgcgcgctacactgatgcattcaacgagcctatccttggccgagaggtccgggtaatc
ttcgaaactgcatcgtgatggggctagatcattgcaattattggtcttcaacgaggaatgcctagtaagcgcgagtcatcagctcgcgttgattacgt
ccctgccctttgtacacaccgcccgtcgctcctaccgattgggtgtgctggtgaagtgtccggattggctccagcgggggaacctctgctgttgct
gagaaggtcattaaaccctcccacctagaggaaggagaagtcgtaacaaggtttccgtaggtgaacctgcggaaggatcattg

>KM020020.1 Tetracystis texensis strain SAG 99.80 18S ribosomal RNA gene, partial sequence; internal transcribed spacer 1, 5.8S ribosomal RNA gene, and internal transcribed spacer 2, complete sequence; and 26S ribosomal RNA gene, partial sequence

ccatgcatgtctaagtatatatactgtgaaactgcgaatggctcattaaatcagttatagtttatttgatggtaccttactcggataaccgtagtaattcta
gagctaatacgtgcgtaaatcccgacttctggaagggacgtatttattagataaaaggccagccgggcttgcccgactcttggcgaatcatgataa
cttcacgaatcgcacggcttcacgccggcgatgtttcattcaaatttctgccctatcaactttcgatggtaggatagaggcctaccatggtggtaacg
ggtgacggaggattagggttcgattccggagagggagcctgagaaacggctaccacatccaaggaaggcagcaggcgcgcaaattacccaat
cccgacacggggaggtagtgacaataaataacaataccgggcatgtctggtaattggaatgagtacaatctaaatcccttaacgagtatccattgg
agggcaagtctggtgccagcagccgcggtaattccagctccaatagcgtatatttaagttgttgcagttaaaaagctcgtagttggatttcgggtgg
gttctagcggtccgcctctggtgagtactgctatggcctacctttctgtcggggacggtctctgggcttcactgtccggagctcggaatcgacgag
gttactttgagtaaattagagtgttcaaagcaagcctacgctctgaatacattagcatggaataacacgataggactctggcctatcttgttggtctgta
ggaccggagtaatgattaagagggacagtcgggggcattcgtatttcattgtcagaggtgaaattcttggatttatgaaagacgaacatctgcgaa
agcatttgccaaggatgttttcattaatcaagaacgaaagttgggggctcgaagacgattagataccgtcgtagtctcaaccataaacgatgccga
ctagggattggcaggtgtttcgttgatgaccctgccagcaccttatgagaaatcaaagtttttgggttccgggggggagtatggtcgcaaggctgaa
acttaaaggaattgacggaagggcaccaccaggcgtggagcctgcggcttaatttgactcaacacgggaaaacttaccaggtccagacacggg
gaggattgacagattgagagctctttcttgattctgtgggtggtggtgcatggccgttcttagttggtgggttgccttgtcaggttgattccggtaacg
aacgagacctcagcctgctaaatagtcacggctaccggtagtcgcttgacttcttagagggactattggcgtttagccaatggaagtgtgaggcaa
taacaggtctgtgatgcccttagatgttctgggccgcacgcgcgctacactgatgcattcaacgagcctatccttggccgagaggtccgggtaatc
ttcgaaactgcatcgtgatggggctagatcattgcaattattggtcttcaacgaggaatgcctagtaagcgcgagtcatcagctcgcgttgattacgt

ccctgcccctttgtacacaccgcccgtcgctcctaccgattgggtgtgctggtgaagtgtccggattgactacagcgggggaacctctgctgttgttg
aaaaggacattaaaccctcccacctagaggaaggagaagtcgtaacaaggtttccgtaggtgaacctgcggaaggatcattg

PTOX dataset
>ENA|HAGC01036235|HAGC01036235.1 TSA: Dinobryon sp. LO226KS, strain LO226KS, contig comp32964_c1_seq2, transcribed RNA sequence.
TLKDEARWKLSQLKLSNDAIWEREKSRKAPWIIKGPYLILCFLLDALFDNKPISRFYFLE
TVARMPYFSYITMLHTYETLGWWRRSAEVKRIHFAEEYNEYHHLLIWESLGGDQDWSVRF
FAQHSAIVYFFVLIVMWIFSPSLAYNFSELIEAHAVDTYAEFAESNKEILQSMDAPPVAK
AYYEAPDMYVFDEFQTSRAPRRPVINSLYDVVCNIRDDEQEHVATMAQCQDPQVLV
>ENA|HAGF01022324|HAGF01022324.1 TSA: Epipyxis sp. PR26KG, strain PR26KG, contig comp28076_c0_seq3, transcribed RNA sequence.
NRLKDELPTSLKLKLTNDAVKQTELTREAHPIARSLYDAGCTAIDYLFDNRPIERFWFLE
TVARMPYFVCVSMLHLYESFGWLRA-PSLRKVHEAEEWNELHHLLIMESLGGDKNWFDRF
MGYHSAIAAYWLLIGTYLYSPQIAYQFMELLETHAVDTYGTFVEENVEILRSLPPPDIAV
AYYTGNDLYTFDDFQVSRGSRRPPCDNLYDVFCNIRDDEKEHVSTMVACQDYSTIG
>CAMPEP_0174979518 /NCGR_PEP_ID=MMETSP0004_2-20121128|14827_1 /TAXON_ID=420556 /ORGANISM=Ochromonas sp., Strain CCMP1393 /LENGTH=562 /DNA_ID=CAMNT_0016231057 /DNA_START=1 /DNA_END=1687 /DNA_ORIENTATION=-
GQQEQSLPGSLGFKLSNKAVAEAERIRDVHPIAQILYDVGCYCIDEYFDERPIPRFWLLE
TIARIPYFSFVSMLHLYESFGWLRQ-PSMRKVHNAEEWNELHHLLIMESLGGDKEWKDRF
IGYHAAIVYYWLIIAVYVLSPRIAYQCMELIECHAADTYATFLRENAATLRSLPAPNVAR
SYYLSEDLYFFDDFQVTRGTRRPPCDNMYDVFKNILDDETEHISTMQACQEYCLSG
>XP_002946776.1 hypothetical protein VOLCADRAFT_79367 [Volvox carteri f. nagariensis]
QEFKEIRARLDQLRLDADDVIAVERERESPLWVKAPFYALCWLLDIMYDNKPIEKFWVLE
TVARIPYFAYISILHLYESLGFWRAGAELRKIHFAEEWNEMHHLQIMESLGGDRAWMDRF
IAEHSAVFYYWVLILFYLVSPRMAYNFMQRVELHAADTYTAFLQRNAAVLESIPPPMVAL
QYYYSEDLYLFDEFQTASPPRRPRCETLLDVFKNIRDDEMEHVKTMIACQNSTIAK
>KXZ56602.1 hypothetical protein GPECTOR_1g541 [Gonium pectorale]
QEFKQIRAKLDRLELDVADVVAVERRRSSPLWVRAPFYALCWVLDVMYDSKPIEKFWVLE
TVARIPYFAYISILHLYESLGFWRAGAELRKIHFAEEWNEMHHLQIMESLGGDRAWVDRF
IAEHAAVLYYWVLIGFYLVSPRMAYNFMQRVELHAADTYTAFLERNAELLSSIPPPLVAL
QYYCSEDLYLFDEFQTASPPRRPRCESLLDVFTNIRDDELEHVKTMVACQNSGIAR
>XP_001703466.1 plastid terminal oxidase [Chlamydomonas reinhardtii]
QEFKQIRAKLDKLELSVSDVVAVEHARTSPAWVKAPFYALCFVLDVMYKNKAIEKFWVLE
TVARIPYFAYISILHLYESLGFWRAGAELRKIHFAEEWNEMHHLQIMESLGGDRAWFDRF
IAEHAAVFYYWVLIAFYLVSPRMAYNFMQRVELHAADTYSAFVERNRTALADIPPPLVAL
QYYYSDDLYLFDEFQTASPPRRPPCENLLDVFTNIRDDELEHVKTMVACQESTIAK
>ABF85790.1 plastid terminal oxidase [Haematococcus lacustris]

AEFDEIKDKLSKLTICNEAVARREAARATPIAVKLPFLALCWVLDVVYDKRPIQKFWVLE
TVARIPYFAYISILHLYESLGFWRAGAELRKIHFAEEWNELHHLQIMESLGGDQAWFDRF
LAEHAAVLYYWLLIAFYLVSPKVAYNFMQRVEHHAADTYCEFLESNRELLASIPPPVVAL
NYYRNQDLYLFDSFQTSSVQRRPDCNTLLDVFINVRDDELEHVATMFAMQNEEIAK
>GAX78356.1 hypothetical protein CEUSTIGMA_g5798.t1 [Chlamydomonas eustigma]
EEWEEIREKLTKLKLDNQAIVEVEKARYTPFWVKLPFLFLCLVLDVLYEGKPIQKFWVLE
TVARIPYFAYISILHLYESIGFWRAGAELRKIHFAEEWNEMHHLQIMESLGGDQSWFDRF
VAEHSAIAYYWLLIFFYLVSPKLAYNFMQRVELHARDTYAVFVENNKELLETIPPPKVAL
SYYLNQDLYLFDEFQQGSTPRRPSCNNLYEVFNNIRDDENEHVKTMDACEKETIAL
>PRW56883.1 plastid terminal oxidase [Chlorella sorokiniana]
PEQKEVLAKVRALKLDDAKVSAREQRRKTNWGIQAAYYSLCWVLDVVYANRPIERFWFLE
TVARMPYFVYISMLHLYESLGWWRAGAELRKVHFAEEWNELHHLQIMEALGGDLRWGDR
F
LAEHAAVFYYWVLVAIYLVSPSASYQFMEMVEEHAADTYAEFAEQNREQLQGIPPPLVAL
NYYKSGDLYLFDEFQTSWELRRPPCNNLYDVFINIRDDELEHVKTMAACQDGSVAL
>AFR58668.1 plastid terminal oxidase [Dunaliella salina]
SECRKIRQKLQQLTLSNKAVAEREHTRETTWYIRLLYDAVCLMLDVVYNNRPLQRFWFLE
TVARMPYFSYISCIHLYETLGWWRAAAELRKIHFAEEWNELHHLQIMEALGGDCMWFDRF
LAYHSAIAYYWVLVALYIFSPRLSYNFSELLEAHAVDTYSEFVSSNEVLLKSLEPPLVAA
QYYRSPDLYMFDEFQTDIEKRTPKCDNLYDVFVNIRDDEAEHVKTMNACQDAEIMD
>GBF89019.1 plastid terminal oxidase [Raphidocelis subcapitata]
EDCQNIRQRLSDLKLSDAAVWAREAARPAPWWIKAPFWALCVVLDLLFANRPIQRFWVLE
TVARIPYFSAISLLHLYESLGFWRAGAELRRIHFAEEWNELHHLQIMEALGGDLLWFDRF
VAEHAALVYYWVCIAFYMAAPKNAYQFSELVEWHAVDTYAQFCETNEALLKSIPPPRVAV
AYYRNEDLYMFDEMQTCSEPRRPSCNNLYDVFSNIRDDEVEHVRTMRACQSPAIAV
>AXB99491.1 plastid alternative oxidase [Caulerpa cylindracea]
DECMQIRAKLNGLKLSNDAVWDRERARTAPWYINLAYYTLCYLLDVLFNNRPLQRFWFLE
TVARMPYFSYISMLHLYESLGWWRQGSILRKVHLAEEWNELHHLQIMESLGGDQYWSDRF
LGQHAAIFYYWILLLFYVFSPRLAYNFSELIEAHAVDTYGEFVDANKELLQSLPPPFVAV
QYYRGDDLYLFDEFQTSGETRRPECNNLYDVFVNIRDDEGEHVKTMKGCQDETIVR
>XP_002950383.1 hypothetical protein VOLCADRAFT_90709 [Volvox carteri f. nagariensis]
NECKAIRSKLKQLKLSNQAVWDREHARETPWFIKGVYLSLCVLLDVLFNNRPIQRFWFLE
TVARMPYFSYISVLHLYESFGWWRAGAELRKIHFAEEWNELHHLQIMESLGGDKLWFDRF
CALHAAVVYYWILLALYVFSPELAYNFSELIEAHAVDTYGEFVDANEELLKSLPPPLVAA
MYYRSQDLYMFDSFQTSQNPRRPPCKNLHDVFCNIRDDELEHVKTMRACQDATVQQ
>XP_005649673.1 hypothetical protein COCSUDRAFT_46597 [Coccomyxa subellipsoidea C-169]

SDCSDVREKLKMLRLENAPVWEREKRRKAPFIINVAYTTLCVVMDVLYNKRPIQRFWFLE
VVARMPYFSYISMLHLYESLGWWRAGAELRRIHFAEEWNELHHLQIMESLGGDQFWIDRF
AAQHAAVFYYWVIVGFFAFSPQLAYVFSELVEGHAVDTYEEFVEENAELLKTLPPPVVAL
EYYKNGDLYLFDELQTTGPKRRPSCNNLYDVFCNIAGDEREHVKTMRACRTYEIVS
>KXZ53636.1 hypothetical protein GPECTOR_6g553 [Gonium pectorale]
NECQEIRVKLKQLKLSNKAVWDREHARKTPWFIKGVYLSLCYLLDFLFDNRPVQRFWFLE
TVARMPYFSYITMLHLYESFGWWRAGAELRKIHFAEEWNELHHLQIMESLGGDQLWFDRF
AALHAAVVYYWILVALYVFSPELAYNFSELIEYHAVDTYGEFVDANEELLKSLPPPLVAA
VYYRSPDLYMFDSFQTSQNPRRPTCKSLYDVFRNICDDELEHVKTMRACQDSTVQQ
>XP_011402281.1 Ubiquinol oxidase 4, chloroplastic/chromoplastic [Auxenochlorella protothecoides]
PKERKVLEKLQALRLDDRRVIAREHKRKAPWLIKGSYVFLCWVLDILYANRPIQRFWVLE
TVARMPYFAYISMLHLYESLGWWRAGAELRRIHFAEEWNELHHLQIMETLGGDHLWIDRF
FAEHAAVFYYWVIILMFFASPSAAYVFSELVENHAVDTYSEFLEENKEVLQGIPPPLVAV
TYYKSGDLYLFDALHTEWSIRRPECNNLYDVFVNIRDDELEHVATMKACAENTIKP
>AAM12876.1 quinol-to-oxygen oxidoreductase [Chlamydomonas reinhardtii]
NNCAEIRSKLKQLKLSNKAMWDREHARETPWLIKGVYLSLCLLLDFLYENRPIQRFWFLE
TVARMPYFSYISMLHLYESLGWWRAGAELRKIHFAEEWNELHHLQIMESLGGDQLWFDRF
AAQHAAILYYWILLGLYVFSPRLAYNFSELIEYHAVDTYGEFWDANEELLKSLPPPLVAA
VYYRSQDLYMFDSFQTSQNPRRPSCKTLYDVFKNICDDEMEHVKTMKACQDETVSQ
>GBF99932.1 plastid terminal oxidase [Raphidocelis subcapitata]
KQCREVRARLKQLKLDNGAVWAREHARETPWLVKGVYLALCVFLDKAYDNRPIQRFWFLE
TVARMPYFVYISCLHLAESMGWWRAGADLRIVHAAEEWNELHHLQVMESLGGDVLWWD
RF
LGTHAAFIYYWVLVGLFVASPSLAYNFSELIEAHAVDTYGEFVDANEELLKSLPPPLVAA
EYYCGQDLYMFDAFQTSDTPRRPKVKNMYDVFANIRDDEKEHVKTMQACQDFTVAG
>ABF85789.1 plastid terminal oxidase [Haematococcus lacustris]
EECAEIRQRLSRLILSNHAVSERERVREAPWYVRGVYNILCLLIDVLFENRPIQRFWFLE
TVARMPYFSYISCLHLYESLGWWRAAAELRKLHFAEEWNELHHLQIMESLGGDQLWFDRF
VGYHSAIVYYWVLVLLYVASPSLAYTFSELLEAHAVDTYGEFLDANEPLLKSLAPPLVAA
EYYRSCDLYLFDEFQTSNTPRNPPLENLYDVFCAIKEDEGEHVKTMHACVDSSLVA
>PSC72506.1 plastid terminal oxidase isoform A [Micractinium conductrix]
EEQRQVLAQVRALSLDDSLVSARERARGASLPITAAYVSLCWVLDFLYAGRPIQRFWILE
TVARMPYFVYISMLHLYESLGWWRAGAELRKVHFAEEWNELHHLQIMEALGGDLRWIDRF
VAEHAAVFYYWVLVVIYLISPSASYQFMEMVEGHAADTYAEFAAQNKEPMQAIPPPLVAL
NYYKSGDLYLFDEFQTSWERRRPACNNLYDVFVNIRDDELEHVKTMVACQDGTVAL

>XP_013906748.1 Alternative oxidase 4 [Monoraphidium neglectum]
AQCREVRAKLQQLTLSNAKVWEREHKRETPWVVKAVYLALCVFLDVAYDKRPIQRFWFLE
TVARMPYFSYLTMLHLLETLGWWRAGAELRKVHFAEEWNELHHLQIMEALGGDQLWIDRF
MAQ-------------------LAYNFSELIEAHAVDTYSEFADANEELLKSLPPPLVAA
EYYTGPDLYLFDSFQTSQQPRRPSVNTLHDVFLNIAADEGEHVKTMRACQDYSVAG
>XP_005850335.1 hypothetical protein CHLNCDRAFT_12129, partial [Chlorella variabilis]
--------------------------------IRAAYASLCWALDVMYAGRPIERFWILE
TVARMPYFVYISMLHLYESLGWWRAGAELRKVHFAEEWNELHHLQIMEALGGDLRWGDR
F
LAEHAAVFYYWALVLIYLISPAASYQFMEMVEGHAADTYAEFAEQNRERLQAIPPPLVAL
AYYKSGDLYLFDQFQTSWELRRPRCNNLYDVFINIRDDELEHVKTMAACQ------
>XP_001421713.1 predicted protein, partial [Ostreococcus lucimarinus CCE9901]
------RAKMDALRLDADAIWARERARSAPWILKVPYVALCVMLDKLFETQPVQRFWFLE
TVARMPYFSYTSMLTFYEILGWWRRSSELRKVHFAEEWNEYHHLLVMESLGGDACWRDRF
LGQHAAIVYYFVLVALWLISPALAYNFSELIEGHAVDTYGQFVDQNAELLKSMPAPRIAV
EYYEAADLYLFDEFQTARRLRRPQIRSLFDVFSNIRDDEGEHVNTMNACQREDSDI
>XP_003083541.1 Alternative oxidase [Ostreococcus tauri]
TMPKELRVKLEALRLDSDAIWEREKNRPAPWLLKAPYLALCFMLDKLFETKPVQRFWFLE
TVARMPYYSYTAALTFYEILGWYRGGAELRKIHFAEEWNEYHHLLIMESLGGDVSWRDRF
LGQHAALVYYGVLILLWFMSPALAYNFSELIEAHAVDTYAQFVDQNAELLKTMPAPRIAV
EYYEGADLYLFDEFQTARRTRRPRIRTLYDVFSNIRDDEGEHVSTMNACQKEDAAV
>GAX73707.1 hypothetical protein CEUSTIGMA_g1160.t1 [Chlamydomonas eustigma]
PEVQARRDLLGKLTLSNQAVWDREHARKSPWVIKVPYYVLCWVLDVLFDGRPIQRFWLLE
TVARMPYFSYINLLHLYETLGWWRRSLEARKVHFAEEWNEAHHLLIMESLGGDQRWADRF
IAQHAAVVYYYVVLNILWFLSPTLAYNFSELIEAHAVDTYGQFVDENEAILKQLPPPRMAR
LYYESEDMYLFEKFQTERGSRHVAVMSLYDVFCNIRDDEAEHVATMHAMQRQDVLD
>QDZ20645.1 ubiquinol oxidase [Chloropicon primus]
ELCVTFEDQLKKLTLDAEGIYERELKRKKSWIIKGPYIVLCWALDRLYKDRPIQRFWLLE
TVARMPYFSYISMLHLYETLGWWRKGAAVKSVHFAEEWNEFNHLLIMESLGGDDSWIDRF
AGYHGAIVYYWVLNLLWLISPYLAYQFSELLENHAVDTYSQFVDENEELLRTLPAPAVAK
NYYQNEDLYMFDEFQTSRSRRRPQINTLYDTFSNIRDDELEHVKTMVACQNTNKSV
>XP_002509380.1 quinol-to-oxygen oxidoreductase [Micromonas commoda]
ELMSELRDQLATLTLSNDAIWAREKARPAPWIIKAPYLALCVFLDLVFENRPIQRFWFLE
TVARMPYFSYNTMLTVYELLGWWRRSSELRRVHFAEEWNEYHHLLIHESLGGDAAWRDRF
LGFHSALLYYGALNVAWLLSPALAYNFSELIEAHAVDTYAQFAEENKATLRKLPAPRIAR
KYYEEGDLYLFDEFQTARSSRRVRVTTLYDAFCAIRDDEAEHVATMAECQMEDSAM

>XP_003061387.1 predicted protein, partial [Micromonas pusilla CCMP1545]
------RDQLATLTLSNAAIWERERSRPAPWIIKAPYFALCTFLDLVFEDRPIQRFWFLE
SVARMPYFSYNTMLTLYELLGWWRRSSELRRVHFAEEWNEYHHLLIMESLGGDALWRDRF
LAQHAALAYYLVLIALWLISPALAYNFSELIEAHAVDTYAQFADENKEKLRTLPAPRIAR
AYYENDDMYLFDEFQTARSSRRPRVRTLHDTFCAIRDDENEHVATMKECQMEDSAM
>XP_007508715.1 predicted protein [Bathycoccus prasinos]
RQGELLLEYLERMKLSNAKIWEREKRLESPWIIEGPYYLLCKVLDLWFENKPVQRFWFLE
TVARMPYFSYTTMLTLYELLGWWRRSSELRKVHFAEEWNEYHHLLIMESLGGDRRWSDRF
LAQHAALVYYFGLVVVWLLSPKLAYNFSEKIETHAVATYAQFTEENKELLESLPAPEVAK
KYYEAEDLYLFDEFQTTTSVRRPKIETLYDVFSNICEDEKEHVGTMNACQIEGVTL
>XP_013899483.1 Alternative oxidase 4, chloroplastic/chromoplastic [Monoraphidium neglectum]
EECKEIRANLAQLKLDDATVWAREEERPAPWWIKAPFWGLCVVLDLLFANRPIQRFWVLE
TVARIPYFSAISLLHFYESLGFWRAGAELRKVHFAEEWNELHHLQIMESLGGDLLWFDRF
VAEHAALAYYWVCT--
----AEGVWLAGALG---RRGAP--------------------SPILVC-------
>PNH11360.1 Ubiquinol oxidase 4, chloroplastic/chromoplastic [Tetrabaena socialis]
--------RNAPFRISAVAQSPEASSPRKPEFIRGVYLSLCLLLDVLYENRPIQRFWFLE
TVARMPYFSYISILHLYESLGWWRAGAELRKIHFAEEWNELHHLQIMESLGGDLLWFDRF
AALHSAIAYYWILLGLYVFSPRLAYNFSELIEYHAVDT----------------------
-------------FQSSRAPRRPPCRSLLDVFTNIRDDEGEHVQTMRACQDSSVRS
>chlamydomonad sp. NrCl902
EQFEEIKAQLDSLRLSNEAVAARERARETPMCIKLPFLLLCWILDSIYEDRPIAKFWVLE
TVARIPYFAYLSCLHLYETLGLWTAGAELRKVHFAEEWNELHHLQIMECLGGDQSWFDRC
IAHNAALLYYWVLVLFYLVSPKWAYNFMQRVELHASDTYTEFIESNRATLAGLPPPRVAL
EYYLGEDTYLFDQFQSGNPKRRPRCESLLDVFINIRDDENEHVKTMEACQGSAQGQ
>Polytomella parva
PEFQKELEHIESLTLTSHQVEEVEHARDSPLWVKVPFFALCWLLDALYENRPIQKFWVLE
TVARIPYFAYISILHLYESLGLWRAGAELRKVHFAEEWNEMHHLQIMESLGGDQHWFDRF
FGQWAAVGYYWLIIAFYMFSPKIAYNFMQRVELHAYDTYAAFVEVNRDLLKQIPPPMVAL
SYYRTKDLYLFDEFQQNPQKRRPECSDLYDTFVNIRDDEFEHVRTMIACQDDTVAK
>CAA06190.1 Immutans protein [Arabidopsis thaliana]
--QATILQDDEEKVVVEES-FKAETSTLEQGVNVFLTDSVIKILDTLYRDRTYARFFVLE
TIARVPYFAFMSVLHMYETFGWWRR-ADYLKVHFAESWNEMHHLLIMEELGGNSWWFDRF
LAQHIATFYYFMTVFLYILSPRMAYHFSECVESHAYETYDKFLKASGEELKNMPAPDIAV
KYYTGGDLYLFDEFQTSRNTRRPVIENLYDVFVNIRDDEAEHCKTMRACQTLGSLR
>XP_015633460.1 ubiquinol oxidase 4, chloroplastic/chromoplastic [Oryza sativa Japonica Group]

EAMRTQREKEQTEVAVEESFPFRETAPLEQSVNIFLTESVITILDGLYRDRNYARFFVLE
TIARVPYFAFISVLHMYETFGWWRR-ADYIKVHFAESWNEFHHLLIMEELGGNSLWVDRF
LARFAAFFYYFMTVAMYMVSPRMAYHFSECVERHAYSTYDKFIKLHEDELKKLPAPEAAL
NYYLNEDLYLFDEFQTARCSRRPKIDNLYDVFVNIRDDEAEHCKTMKACQTHGNLR
>XP_024367795.1 uncharacterized protein LOC112278476 [Physcomitrella patens]
LEQDELRAALKKLTLSNQAVWDRENAREAPWWILGPYYLLCLMLDVIFNDRPIQRFWFLE
TVARMPYFSYISMLHLYETLGWWRIGAEVRKVHFAEEWNEMHHLRIMESLGGDLEWGDRF
FAQHAAFFYYWVLNLMFLISPKVAYNFSELIEMHAVDTYGEFADANEELLKTLPPPPAAL
EYYESEDLYMYDEFQTSQETRRPKINNLYDVFKAISGDELEHVKTMSACQTLDMPI
>PTQ49270.1 hypothetical protein MARPO_0003s0157 [Marchantia polymorpha]
IEQDELREQLKKLKLSNKKVWEREKAREAPWWILGPYYFLCWMLDVIFEDRPIQRFWFLE
TVARMPYFSYISMLHLYETLGWWRSGAEVRKVHFAEEWNEMHHLKIMESLGGDLEWGDR
F
FAQHAAFFYYWTLNAMFLISPTVAYNFSELIESHAVDTYGEFADENEELLKTLPPSPVAV
AYYESGDLYMYDEFQTSRESRRPKMGSLYDVFMAICGDEGEHVKTMVACQQLDTQV
>XP_002984492.2 uncharacterized protein LOC9644228 [Selaginella moellendorffii]
TEQEDLRKALAKLTLNRDAVWERERNREAPWWILGPYYALCWMLDVIFEGRPIQRFWFLE
TVARMPYFSYISMLHLYETLGWWRVGADVRKVHFAEEWNEMHHLKIMESLGGDLLWGDR
F
FGQHAAFFYYWILNFMFFVSPKVAYNFSELIEMHAVDTYGQFFDENEELLKQLPPSPEAV
AYYENEDLYMFDEFQTSRESRRPKVDSLYDVFVAIKGDEFEHVKTMAACQRAETVA
>GAQ78184.1 Hypothetical protein KFL_000090280 [Klebsormidium nitens]
AEMDELRKKLSQLKLSNDKVWERERSREAPWYILGPYYFLCLILDVIFNGRPIQRFWFLE
TVARMPYFSYISMLHLYETLGWWRVGAEVRKVHFAEEWNEMHHLKIMESLGGDNQWVDR
F
FGQHAAFFYYWILNFLFLVSPKVAYNFSELIEFHAVDTYEEFAEANKELLMSLPPSPVAV
EYYRGEDLYMFDEFQTSRATRRPKVDNLYDVFCNIRDDEFEHVKTMVACQDGGQPI
> XP_028109225.1 ubiquinol oxidase 4, chloroplastic/chromoplastic isoform X1 [Camellia sinensis]
--QATVLQDDEQKAAVENS-FQRKTIVFEQSVNILLTDSVIKILDVLYRDRDYASFFVLE
TIARVPYFAFMSVLHLYESFGWWRR-TDYLKVHFAESWNEMHHLLIMEELGGNSWWFDRF
LAQHIAVFYYFMSVFMYALSPRMAYHFSECVESHAFETYDKFIKTRGEELKKLPAPEVAV
KYYTGGDLYLFDEFQTSRKSRRPKIENLYDVFVNIREDEGEHCKTMKACQTHGNLR
>Polytomella magna
PEFQKELEKVESLTLTNKQVQDVQHARESPVWVKIPFCVLCWLLDVLYDDRPIQKFWVLE
TVARIPYFAYISILHLYESLGFWRAGAELRKVHFAEEWNEMHHLQIMESLGGDQHWFDRF
LAQWSAVGYYWLIILFYMFSPKAAYNFMQRVELHAYDTYSAFVEVNRELLEDLPPPLVAL

NYYRTKDLYLFDEFHQRAEPRRPKCDNLFDTFVNVRDDEYEHVKTMIACQDDTVAK
>Nitzschia sp.
RLEMMASPQDSSPTVLSSSNSTLLPSTFNKNLIDTLYKIMCILYPVKGNDRDFVRFYILE
TVARVPYFAYLSVMHLRETFGVRFESSDRMRTHYAEADNELHHLLIMESLGGNSSCVDRT
LAQTAAFLYYWYVVVIYMWDEAAAYHLSELIEDHAYETYDAFLKEHGERLKTMPVPEIAR
QYYEVGDPCFFDQFSTVPHRIRPPLQSLYDVFVLVRDDEKEHWRTLCNLVQFQEMN
>Spumella sp.
SFDSDVQRKIGTLTLSNRAVEAAERFRQVNFISRLLYSIGCKLLDVLFESKPIQRFWFLE
TIARIPYFSYLTTLHLYETLGLWRK-HALRRVHNAQEWNEHHHLLIMETLGGNKSLMDRL
IAYAAAIWYYWTITMFYMISPFVAYQFMELLEGHAVDSYAAFLIENKETLQQLPAPRVAQ
EYYTGKDLYYFDEFQVFTGTRRPKIKTLFDVFQNICLDEKEHVKTMQACQTYSTDG
>CEM05282.1 unnamed protein product [Vitrella brassicaformis CCMP3155]
ENTIRLRQELSRLRLDNDAVWRREEERNSPLLLKIPYLFLCWLLDVLYNERPIQRFWFLE
TVARMPYFAYISMLHLYETLGWWRVGTEVRKVHFAEEWNEMHHLFIMESLGGDQAWLDRF
LARHAAIVYYWVLILLFLISPTWAYNFSELIEWHAVDTYGQFVSQNKQILKRLPAPAVAM
EYYKSGDLYMFDEFQTSRQSRRPQINNLYDVFTTIRDDELEHVKTMSACQRLEAAM
>CEM04265.1 unnamed protein product [Vitrella brassicaformis CCMP3155]
EETIRLRQELSRLSLDDDGVIRREEERNAPLLIKVPYLFLCWLLDRLYAGRPIQRFWTLE
TVARIPYMSYISMLHLYETLGWWRVGVEVRRVHFAEEWNEMHHLFIMESLGGDQAWFDRF
LARHAAIVYYWVVIFLFLISPTWAYNFSELVEWHATDTYAEFVEQNKDRLKRLPAPAVAL
EYYNGGDLYLFDEFQSSRESRRPNIDNLYDVFVTIRDDELEHVKTMSACQRLESAM
>VEU38912.1 unnamed protein product [Pseudo-nitzschia multistriata]
GGEPEITDMTAASPQXSLSTPAEIEPVFNKLLIDTVYDVICFLYPVKGTERDFARFYVLE
TVARVPYFAYLSVMHLRETFGERYGSSERMRTHYAEADNELHHLLIMESLGGNSNIIDRT
LAQTMAFFYYWYVIAIYVWNEPAAYHLSELIEDHAFNTYTKFTAEYGDRLKNEPVPDIAR
KYYERDNPFLFDLFCTVKSERRPQLSSLYDVFVNIRDDEKEHWKTLCNLVQYDDMN
>OEU10955.1 plastid terminal oxidase-like protein [Fragilariopsis cylindrus CCMP1102]
AAESSTTTTTTIPFPSSTSNGAVTPDIFNKAVIDTVYDIICFLYPVKGTERDYARFYVLE
TVARVPYFAYLSVMHLRETFGERYPKSDRMRTHYAEADNELHHLLIMESLGGNSNIIDRT
LAQTMAFGYYWYVIVIYTFNESAAYHLSELIEDHAYNTYSKFTEEYEERLKLEPVPDVAR
KYYELDNPFLFDLFCTVKSSRRPQLTSLYDVFINIRDDEKEHWKTLCNLVQYDDMN
>XP_009033364.1 hypothetical protein AURANDRAFT_20495 [Aureococcus anophagefferens]
--------------------------FNKVVIDTVYDVICLLYPVTGSERDFARFYVLE
TVARVPYFAYLSVMHLRETFGDRDPGSERMRTHYAEADNELHHLLIMESLGGNSSAVDRT
LAQTMAFFYYWYVTVVYSFSEPAAYHLSELIEDHAFNTYDGFLRDHGPKLKGMPVPDIAR
KYYERDDPFLFDQFCTVKSRSRPPLESLYDVFVNIRNDEKEHWKTLCNLVQLDDTQ

>XP_009034449.1 hypothetical protein AURANDRAFT_14460, partial [Aureococcus anophagefferens]
--------------------------ATKGAIDALYE-----------GRDFARFYVLE
TLARVPYFAYLSVMHLRETFGDRRPGSERMRTHYAEADNELHHLLIMESLGGNSSAVDRT
LAQSMAFFYFWYVTVVYSFSEPAAYHLSELIEDHAFNTYDDFIKDHGPKLKGMPVPDIAR
KYYERDDPYLRDQFLTVKSRSRPPLESLYDVFVNIRDDEKEHWKTL----------
>EWM22362.1 immutans protein [Nannochloropsis gaditana]
PIFKSMAANQDGELQRDNSDSSEQAKVANKAIVSSIKD----ALVAVYGDRHYARFHALE
TIARVPYFAYTSVLHLYETLGWRRR-ADLMKIHFAESWNELHHLLIMEELGGNAEWLDRL
VAVHLAFFYYWMAIALYMASPETAYNLNEMVERHAYDTYDKFLETHEEELKALPPPQIAK
EYYQTGDLYMFDEFHTNSERRRPKIESLYDVFVNIRNDEREHFMTMAVMQGHSGDA
>EJK63007.1 hypothetical protein THAOC_16363 [Thalassiosira oceanica]
----SARRTSLGSAIAPPDDPDRQLGSLNKSSIDFLKST---VFDSFFEDRAYARFYALE
TIARVPYFSYLSVLHLYETLGKWRR-AKYLKLHFAESWNELHHLLIMEELGGSERFLDRF
LAQHIAFGYYAVVILLYVLNPVQAYNLNQDVEEHAFETYDKYLKDNEEKLKNLPAPKAAI
DYYVDGDMYMFDEFQTGCEFRRPKIENLYDVFVAIRDDEAAHVQTMEQLQTELDVA
>XP_002293901.1 predicted protein [Thalassiosira pseudonana CCMP1335]
PITTAVRTTSHHSSLFASSTAPENERAANRFAIGFLKGT---IFDTFFEDRAYARFYALE
TIARVPYFSYLSVLHLYETLGKWRR-VKYLKLHFAESWNEMHHLLIMEELGGSERFFDRF
LAQHCAFGYFLIVITLYLINPVQAYNLNQDVEEHAFATYDTFLKENAEMLKTKPAPKVAI
EYYRHGDMYMFDEFQTECELRRPEINNLYDVFVAIRDDEMAHVKTMEKLQTELDVS
>XP_002182610.1 predicted protein [Phaeodactylum tricornutum CCAP 1055/1]
GVSNPLRTERTQPVVSANTVSTLPETKRNRQLVHQLKSV---LFDQVYMDRAFARFYALE
TIARMPYFSYLSVLHLWETLGMWRR-AEYLQVHFAESWNELHHLLIMEELGGNGRWGDRF
VAQHIAFFYYWIVVTLYAVNPTMAYNLNQAVEEEAYETYDGFLQTHAEYLQSQPAPQAAI
RYYTGDDLYLFHAMHDTREQRRPTCETLYDTIRNIRDDELEHVKTMACLQDESV--
>OEU09946.1 plastoquinol terminal oxidase-like protein [Fragilariopsis cylindrus CCMP1102]
VDKEDRLPDCLNFILTNESVKNAEIERDSHPVSQTLYDLGCLVLDNLFDNRPIQRFWFLE
TIARIPYFSYVSMLHLYESFGWFRA-VELRKIHSAEDWNELHHLLIMESLGGNTKWSDRF
VAYHTAFLYYWFLILVYLGSPRIAYQFMELLEAHAVDTYTTFVKVNKQRLSELPPPSVAV
SYYKYGDLYMFDDFQTTRGSRRPPCDTLLDVFTNIAIDEGEHVKTMQACQDYAKFG
>GAX22341.1 hypothetical protein FisN_3Hh453 [Fistulifera solaris]
PTLAGIRRDASSILKSSSTADNSDTK-GHRQVVKTIKSI---LFDNLYLDRSYARFYALE
TIARMPYFAYTSVLHFLETTGKWRQ-ADRLALHFAETWNELHHLLIMEELLTQSKFRDRF
VAQHVAFFYFWFAVGLYLVRPDAAYSLNQAVEEEAYATYDAFLKEREDYLKSQPAPEVAI
NYYNGSGQDMFDRMSKALSSRRVECKTLYDCFVAIRDDELEHSQTMELLKREPFEL

>TFJ81505.1 hypothetical protein NSK_007176 [Nannochloropsis salina CCMP1776]
------RPPKASINRSPEAITRREEIRETPWFVLLPYYALCAFLDAVYEGRPLARFWFLE
TVARMPYFSYISMLHLYETLGWWTIGTDVRRVHFAEEWNELHHLQIMESLGGDARWLDRF
LARHSAIAYYWVLNLMFLISPRLAYNFSELIESHAVDTYSQFLDENEDLLRRLPAPRVAL
DYYVMGDLYMFDEFQTTRQTRRPLIRSLYDVFSAIRDDESEHVKTINACQVADEVI
>CBJ31032.1 conserved unknown protein [Ectocarpus siliculosus]
PKGRPLLDEQSPTSASDSSATVVSPGRFMNDFNAGVVDFLKGLMVQYYGERTYARFYALE
TIARVPYFGYLCVLHLYETLGKWRQ-AEYLKVHFAESYNELHHLLIMEELGGNVLFRDRW
FAQHAAFFYFFVVVGLYLSNPRNAYNLNQHVEEHAFSTYDSFLSDNQEALKQQPAPEASL
G------------------------------CLVRNDEWEHVKTMKAMQL-----
>XP_002296917.1 predicted protein [Thalassiosira pseudonana CCMP1335]
PRQRELQAKLNELTLSSDAIWERERARVAPYIIKIPYYALCFMLDVVFEGRPFSRFFLLE
TVARMPYFSYITMLHLYETMGFWRRSSDIKRIHFAEEWNEFHHLLIMESLGGDQPYWVRL
MAQHSALAYYIALCMLWMLSPTLSYKFSEMLETHAVDTYGQFVDENEDKLKELPPSIAAV
EYYTGVSDPMFGEYQTASRVRVPNLRSLYDVFVAIRNDEGDHVSTMKSCLDPKEAT
>CBJ25453.1 alternative oxidase, mitochondrial protein [Ectocarpus siliculosus]
PEVVDLQNRLNELKLSNKAIEDRETARQTPWYVMAPYKALCWFLDVVFEDRPIQRFWFLE
TVARMPYFSYLSMLFLYETLGWWSGAAEVRKVHFAEEYNEMQHLRIMESLGGDTRWSDRF
LARHAAIIYFSVLILGYLVSPFLAYNFSELIESHAVDTYTEFAEANEELLKSLPPTPQAL
DYYHGGDMYLFDEFQTSRFSRRPRIQNLYDVFSCIRDDELEHVKTMFACERGTGAL
>GAX11985.1 hypothetical protein FisN_8Lh079 [Fistulifera solaris]
QQQRDFRDELEQLTLNSTEIWAKEFADVAPWIIKIPYLVVCYMLDVLFEKEVFARFFLLE
TVARMPYFSYITMLHLYETLGFWRRSADMKRVHFAEELNEFRHLLIMESLGGDQKWWVRF
LAQHSAIVYYVVLCVLWAVSPSLSYRFSELLETHAVNTYSTFLEENEARLRQLPPSMAAV
EYYAGSSDSFYAEFQTSAEIRRPQIRSLYDVFHTIRDDESDHASTMASCLNPDSNL
>EJK59088.1 hypothetical protein THAOC_20735 [Thalassiosira oceanica]
PRQKELQAKLGQLTLNSTAIWERERARVAPLVIKFPYYALCFLLDVVFEGRAFSRFFLLE
TVARMPYFSYITMLHLYESLGFWRRSSDIKRIHFAEEWNEFHHLLIMESMGGDQPYWVRF
LAQHSAVAYYVALCLLWIASPSLSYKFSEMLETHAVDTYGQFVDENESKLKELPPSLVAV
EYYTGLSDPMFGEYQTASGVRKPSMNSLYDVFCAIRNDEGDHVQTMSSCLDPKVST
>XP_002185130.1 predicted protein [Phaeodactylum tricornutum CCAP 1055/1]
DSQKELHAQLDALTLNATGIWDREMQNEAPFVIKIPYFGLCYMLDEVFDGKIPSRFFLLE
TVARMPYFSYITMLHLYETLGFWRRSAGMKRIHFAEELNEFHHLLIMESLGGDQAWWVRF
LAQHSAIVYYVALCLLWGISPSLSYRFSELLETHAVSTYGQFLDENEEALKKLPPPLPAI
EYYAGSSDPFYAEFQTTAPLRRPSMLSLYEVFQAIKADELDHVSTMEACLDPEANT
>KOO33882.1 plastoquinol oxidase immutan [Chrysochromulina sp. CCMP291]

-------------------------FNKFLIDTVYDVICLIYSKHSIEQCASRFYVLE
TVARVPYFGYLSVMHLKETFGNRAVGKDRMRTHYAEADNELHHLLIMESLGGNSSLFDRT
LAQSMAFGYYWYVVGVYSFSEQAAYHLSELIEDHAYNTYDKFLSTNEDTLRKMPVPAIAR
KYYEEENPFMFDLFCTVKLASRPKLESLYDVFVNVRNDEREHWKTLCNLVQYEDMR
>XP_005794311.1 hypothetical protein EMIHUDRAFT_447503 [Emiliania huxleyi CCMP1516]
AAADALGAAVPSPQLDPALVAELSEAVFNTAIVDSVKG----LIDLAYPDRPFARFYVLE
TVARVPYFAYLSVMHLRETWGERDASRERMRVHYAEADNELHHLLIMESLGGNSSAVDRA
VAQGLATAYYWWVVFVFAHDERAAYHLSELVEDHAYRTYDAFLASHEVELRALPVPEVAR
RYYEQENPFLFDLLCTIGCPRRPRLRSLYDVFVCVRNDEREHWGAMSSLVSTGTVR
>XP_005787845.1 hypothetical protein EMIHUDRAFT_462437 [Emiliania huxleyi CCMP1516]
PGDPLRPPACLGLSLSNEAVKEAERRRDAHPVSRTLYDVGCLLLDNLFDQRPIQRFWFLE
IIARIPYFSYVSMLHLYESFGWWRG-PELRKVHNAEEWNELHHLLIMEALGGNAQWSDRF
LGYHVAFAYYWFLIGVYLCSPRIAYQFMELLEAHAVDTYSTFVKENRERLSQLPPPAVAR
SYYTEGDLYYFDDFQVDRGSRRPACDSLLDVFENIAIDEGEHVKTMRACQDYAVLG
>XP_005840480.1 plastid terminal oxidase [Guillardia theta CCMP2712]
EDSPVISENFDSMFRKDGTTWGSKSYPVTKSIAQALVTVLKGSLDWYYHKKDFARFFVLE
TVARVPYFAYMSVLHLYESFGYHDR-AHWIKIHYAEADNELHHLLIMEALGGNKEFSDRW
IAQHAAFAYYWFCVTFYFLHPRGAYYVMSLIENHAYHTYSKYIEANKSWLASQPSPAIAK
EYYEGGDLYLFDAFHTTRQERRPRVQSLLDTFESIRDDESQHHRTMLSLVERGRP-
>XP_005824706.1 centrin/plastid terminal oxidase fusion protein [Guillardia theta CCMP2712]
PEARRLQEKVARLVLDNDAVAKREEIRATPALIMVPYTFLCWLIDVLFVNRPIQRFWFLE
TVARMPYFSYITMLTLYESLGWWRSSMDSRRVHFAEEWNEVQHLKIMEALGGDRSWFDRF
MGRHAAIFYFIILNHIWLLSPSLAYNFSELIEFHAVDTYGEFVDANEELLKSLPPPQEAV
EYYNSKDLYLFDEFQTGRKSRRPVIRSLYDVFCNIRDDELEHVKTMFQCQTSLQQI
>Monotropa hypopitys
LQPRKLFRVQATVLQDDEQKAAVENSFFEQSVNILLTDSVIKILDVLYRDRDYASFFVLE
TIARVPYFAFMSVLHLYESFGWWRR-TDYLKVHFAESWNEMHHLLIMEELGGNSWWFDRF
LAQHIAVFYYFMSVFMYALSPRMAYHFSECVESHAFETYDKFIKTRGEELKKLPAPEVAV
KYYTGGDLYLFDEFQTSRKSRRPKIENLYDVFVNIREDEGEHCKTMKACQTHGNLR

FNR dataset

>CAMPEP_0175010974 /NCGR_PEP_ID=MMETSP0005-20121125|8393_1 /TAXON_ID=420556 /ORGANISM=Ochromonas sp., Strain CCMP1393 /LENGTH=372 /DNA_ID=CAMNT_0016266843 /DNA_START=64 /DNA_END=1182 /DNA_ORIENTATION=+
QTFLENDPYYDMSNLPMQLFKAKEPHVGKIISVKRIVGADATGETCDIIIDHFGKMPYWE
GQSYGVIPPGTNPKGKPYTNRLYSIASTRYGDDMKGTTTTLCVRRATYWDELGREPAKKG
VCSNFLCDAKPGDEVMLTGPSGKVMLMPATPEADIIMVATGTGIAPYRSFLRRLFVEATP
AGKFKGLAWLFLGVANTDSLLYDADWQKIKEKYPENFRVDYALSREQSNKSGGKMYIQDK
VEEYSDEVFDRMSKGAHMYFCGLKGMMPGILDMLKGVAEKKGLVWADQLKEWKEAGQ
WHV
EVY

>ENA|HAGC01007494|HAGC01007494.1 TSA: Dinobryon sp. LO226KS, strain LO226KS, contig comp21561_c0_seq1, transcribed RNA sequence.
QDFLEADPYYDQSNIPLQLFKAKEPLIGKIISVKRIVGAQATGETCDIIIDHFGKMPYWE
GQSYGVIPPGINPKGKPYTNRLYSIASTRYGDDFKGTTTTLCVRRAVYYDELGREPAKKG
VCSNFLCDAKPGDEIALTGPTGKVMLMPKNPNADIIMVATGTGIAPYRAFIRRLFVEKTP
AAAFKGLAWLFLGVANTDSLLYDEDWQKIKAKYPNNFRLDYALSREQNNKKGGKMYIQD
K
VEEYADEIFERLEKGAHIYFCGLKGMMPGILEMLESVSVKKGIDWKEKLTHLKEEGQWHV
EVY

>ENA|HAGF01002525|HAGF01002525.1 TSA: Epipyxis sp. PR26KG, strain PR26KG, contig comp12208_c0_seq1, transcribed RNA sequence.
QDFLEGNPYYDTSFMPMQTYKATAPYIAKIISVKRIVGAQATGETCDVVIDHSGKLPYWE
GQSLGVIPPGINAKGKPHTNRLYSIASTRYGDDMKGTSVTLCVRRATYFDELGREPAKKG
VCSNFLCDAKPGDPVTCTGPTGKVMLMPKTPEADIIMVATGTGIAPYRSFLRRLFVEKTP
AGKFKGTAWLFLGVANVDALLYDEEWQKIKTAYPNNFKLDYALSREQTNKKGGKMYIQDK
VEEYADDIFGRMEKGAHIYFCGLKGMMPGILEMLEAVSKKKGIVWADKLKHWKEQGQWH
V
EVY

>ENA|HAFO01017510|HAFO01017510.1 TSA: Uroglena sp. WA34KE, contig comp195067_c0_seq1, transcribed RNA sequence.
QNFLEADPYWDNSNIPLQTFKAKEPLAGKIISVKRIVGAQATGETCDIIIDHFGKMPYWE
GQSYGVIPPGINPKGKPHTNRLYSIASTRYGDDMKGTTTTLCVRRATYWDELGKDPAKKG
VCSNFLCDAKPGDEVMLTGPSGKVMLIPKTPEADIIMVATGTGIAPYRSFLRRLFVEATP
AAKFKGLAWLFLGVANTDSLLYDADWQKLKEKFPKNFRLDYALSREQNNKQGGKMYIQD
K

VEEYADEVFDRMSKGAHMYFCGLKGMMPGIIEMLEGVCQKKGLVWADTLKQWKEAGQW
HV
EVY
>TRINITY_DN19663_c0_g1::TRINITY_DN19663_c0_g1_i1::g.3235::m.3235 TRINITY_DN19663_c0_g1::TRINITY_DN19663_c0_g1_i1::g.3235 ORF type:complete len:391 (+),score=78.61,sp|Q9S9P8|FNRR2_ARATH|60.63|1e-138,NAD_binding_1|PF00175.16|2.7e-23,FAD_binding_6|PF00 [Rhodelphis limneticus]
EKVTLAPTHLENGKVPMNTWRNEKPYKGKILSIEKIVGPKAPGEIYHIVIDHRGEMPYWE
GQSFGVLPPGTDPKGKPYGVRLYSIASSRYGDHFDGRTASFCIRRAVYVDKTGKEPSKKG
VCSNYICDLKPGDEVSLAGPTGKVLLMQEECTKPQIMIATGTGIAPFRSFVRRLFLENVP
RSVEQPLAWLFMGVPNKDGLLYQEEFDHMQRRNASRFRIDYAISREPKNKHGGKMYVQDK
VEEHADELWNLMEKGAHFYFCGLKGMIPGITKMFARVAKERGESWDNKLKMLKKNKQWH
V
EVY
>GGOE01006969.1 TSA: Euglena longa strain CCAP 1204-17a Contig6985, transcribed RNA sequence FNR
ISRKKVLVNLEEGVVPLNTFGPKAPFVGKVQSVEHIVGPDAANKIWNVVIETNGKMKYWE
GQSCGVIPPGTNSKEVPHGTRLYSIAASRYGDNFDGNTMTLCVRQATYFDELGSEPAKKG
ICSDFLCNATPGTEVAMTGPTGKSLLLVKDPLATFICVATGTGIAPFRSFWRRMFYEDIP
NYNFHGFFWLFMGVANHDALLYDNEIQEITLTYPKNFRVDYAFSREETNKSGGKMYIQDK
VEENAEQIFDLFENGAHIYFCGKKEMMHGMRHVFQRVANQKGLDWEEYFSSLKHNNRWH
T
EVY
>GEFR01008144.1 TSA: Euglena gracilis EG_transcript_8143 transcribed RNA sequence
ISRKKVPLKLEEGALPMNTFGPKAPFIGKIRSVERIVGPKATGETCNIVIETNGKIPYWE
GQSYGVIPPGTNSKEVPHGTRLYSIAASRYGDTFDGNTTTLCVRRATYWDELGREPAKKG
ICSNFLCDAKPGTEVVMTGPTGKILLLDNDPCAAHICVATGTGIAPFRSFWRRMFYEEIP
NYKFNGIFWLFMGVANRDALLYDNEIQEIAATYPKNFRVDYAMSREETNKRGGKMYIQDK
VEEYADQVFGLLEGGAHIYFCGLKGMMPGIQEMLERVAKERGLNWEEYFSKLKHNNQWH
V
EVY
>GEFR01013430.1 TSA: Euglena gracilis EG_transcript_13435 transcribed RNA sequence
ISRREVPLNLEEPAVPSNTFTKDQPFVAVVESKRLLTGPKALGETYHIVLRTNGAIPCRE
GQCYGVIPPGTGPDPKAHYPRLYTIATSRYGDHCDGQTTSLCVKKIAWWDQVGGL--KKE
LCSHFLCDAEPGTEVTMIGPTANSMLLDPAPDAVHICVATGTGVAPYRAFWRRLFCERGP
GEVFRGRLWLFFGGANPDELLYADELADLQATYPDQVQLVTAFSEVEQTASGEKMFVQDK

LALHAEEVFRLLQSGAHLYCSGSKFMMPGIQSTL-------------------------

>Polytomella parva
VSKKEVSKGLEGGKLPLNTFSTKKPYTAKVLSVEKIVGPKATGETFHVVLDTQG-VKYHE
GQSLGVIPPGTNSKEVPHGTRLYSIASSRYGDNFEGNTTTLCIRRAVYTDKTGKEPAKQG
ICSNYLTKAAPGTEVTVTGPTGKVLLLPEDASQTVICVATGTGIAPFRSFWRRCFVENVP
GYKFNGKLWLFMGVANSDSKLYDEELQALKKAYPEQFRLDYALSREQKNSKGQKMYIQDK
VEEYADEVFSLLDNGAHIYFCGLKGMMPGIQGMLERVAKSKGLVYADWVEKLKHKSQWH
V
EVY
>XP_005644145.1 ferredoxin-NADP+ reductase [Coccomyxa subellipsoidea C-169]
LKKNKVPAELEKGDLPMNTFNNKKPFKATVKSVERIVGPKATGETCHIVIETRGEIPYWE
GQSYGVIPPGTNSKEVPHGTRLYSIAATRYGDTFDGKTTSLCVRRAEYWCEMKANPAKKG
ICSNFLCDAKPGDEITMTGPTGKILLLPEDKNAAIIMVATGTGIAPYRAFWRRFFLEEIE
GYKYTGLAWLFMGVANSDAKLYDDELQAILKAHPDQFRVDYALSREQTNKNGGKMYIQD
K
VEEYSDEVFDLLDNGAHIYFCGLKGMMPGIQEMLERVASEKGMVWEEFFQKLKKNNQWH
V
EVY
>XP_002954986.1 ferredoxin-NADP+ reductase [Volvox carteri f. nagariensis]
VSKRTVPTALEEGEMPLNTYSNKAPFKAKIRSVETITGPKATGETCHIIIETEGKIPFWE
GQSYGVIPPGTNSKEVPHGTRLYSIASSRYGDDFDGKTASLCVRRAVYVDETGKEPAKKG
ICSNYLCDATPGTEIVMTGPTGKVLLLPADANAPLICVATGTGIAPFRSFWRRCFMENVP
SYKFTGLFWLFMGVANSDAKLYDEELQALAKAYPSQFRLDYALSREQKNRKGGKMYIQDK
VEEYSDEIFDLLDNGAHMYFCGLKGMMPGIQEMLERVAKSKGLNYEEWVEGLKHRNQWH
V
EVY
>KXZ46591.1 hypothetical protein GPECTOR_42g802 [Gonium pectorale]
VTKRTVPTQLEEGEMPLNTYGNKAPFKAKVRSVETITGPKATGETCHIIIETEGKIPFWE
GQSYGVIPPGTNSKEVPHGTRLYSIASSRYGDDFDGKTASLCVRRAVYVDETGKEPAKKG
ICSNFLCDAKPGTEIAMTGPTGKVLLLPADANAPLICVATGTGIAPFRSFWRRCFMENVP
SYKFGGLFWLFMGVANSDAKLYDEELKALAAAYPSQFRVDYALSREQKNRKGGKMYIQDK
VEEYADEIFGLLDGGAHMYFCGLKGMMPGIQEMLERVAKSKGLNYEEWVEGLKHKNQWH
V
EVY
>XP_003084170.1 Ferredoxin-NADP+ reductase [Ostreococcus tauri]

NGRAKVPLEMEKMELPLNTYKNKEPFVGTIRSVERIVGPNATGETCHIIIEHGGKMPFWE
GQSYGVIPPGTNSKEVPHGVRLYSIASSRYGDSYDGLTATLCVRRATYWDEMNAEPAKKG
ICSNFLCDAKPGQEVMMTGPTGQVMLLPKDPATPVIMVATGTGIAPMRSYLRRFFLEDIP
SWEFKGLAWLFMGVANSDAKLYDDEFQEMVKRFPDQFRIDYALSREDTNKNGGKMYIQDK
VEEYKDQVFQLLDGGAHMYFCGLKGMMPGILSMLEGVCKEKGISYEEWLEGLKKNGQWH
V
EVY
>XP_001697352.1 ferredoxin-nadp reductase [Chlamydomonas reinhardtii]
MSKRTVPTKLEEGEMPLNTYSNKAPFKAKVRSVEKITGPKATGETCHIIIETEGKIPFWE
GQSYGVIPPGTNSKEVPHGTRLYSIASSRYGDDFDGQTASLCVRRAVYVDETGKEPAKKG
LCSNFLCDATPGTEISMTGPTGKVLLLPADANAPLICVATGTGIAPFRSFWRRCFIENVP
SYKFTGLFWLFMGVANSDAKLYDEELQAIAKAYPGQFRLDYALSREQNNRKGGKMYIQDK
VEEYADEIFDLLDNGAHMYFCGLKGMMPGIQDMLERVAKEKGLNYEEWVEGLKHKNQWH
V
EVY
>XP_003064407.1 ferredoxin-NADP oxidoreductase [Micromonas pusilla CCMP1545]
GGRAPVPLELEKMPLPLNTFKNKAPFTGKIKSVERIVGPNATGETCHIIIEHGGKMPFWE
GQSYGVIPPGTNSKEVPHGVRLYSIASTRYGDEFDGNTATLCVRRATYWDEKNAEPAKKG
ICSNFLCDAKPGAEVMMTGPTGQVMLLPEDPATPVIMVATGTGIAPMRSYIRRFFVEDVK
NWEFKGLAWLFMGVANSDAKLYDDEFSECIKRFPGQFRVDYALSRESQNRKGGKMYIQDK
VEEYKDQVFQLLDGGAHMYFCGLKGMMPGILEMLEGVCKEKGIDYEEWLEGLKKKGQW
HV
EVY
>P53991.1 RecName: Full=Ferredoxin--NADP reductase, chloroplastic; Short=FNR; Flags: Precursor [Chlamydomonas reinhardtii]
MSKRTVPTKLEEGEMPLNTYSNKAPFKAKVRSVEKITGPKATGETCHIIIETEGKIPFWE
GQSYGVIPPGTNSKEVP-TARLYSIASSRYGDDGDGQTASLCVRRAVYVDETGKEPAKKG
LCSNFLCDATPGTEISMTGPTGKVLLLPADANAPLICVATGTGIAPFRSFWRRCFIENVP
SYKFTGLFWLFMGVGNSDAKLYDEELQAIAKAYPGQFRLDYALSREQNNRKGGKMYIQDK
VEEYADEIFDLLDNGAHMYFCGLKGMMPGIQDMLERVAKEKGLNYEEWVEGLKHKNQWH
V
EVY
>KXZ48298.1 hypothetical protein GPECTOR_29g73 [Gonium pectorale]
VSKRTVPTKLEEGQLPMNTFSPKATLKARIKSVEKLTGPKATGETYHIIIETEGKIPYWE
GQSFGVIPPGTNSKEVPHGTRLYSIASSRYGDYFDGATATLCVRRAVYVDETGAEPAKKG
VCSNFLCDAAPGTEINMTGPTGKVLLLPSDPNATLICVATGTGIAPFRSFWRRLFMEDTG

RPAFGGLFWLFMGAANADATLYDAELQALAAAHPEHFRLSYALSREQKNKRGGKLYIQDK
VEEYADEVFSLLDGGAHMYFCGLKGMMPGIQEMLSRVAGEKGLTYEHWVEGLRARGQWH
V
EVY
>PRW50852.1 ferredoxin-NADP+ reductase [Chlorella sorokiniana]
LKRADVPLELEEIGLPMNTFGPKNPFIGKIVSVETITGPKATGETCHIIIQTDKKIPFVE
GQSYGVIPPGTNSKEVPHGTRLYSIAATRYGDSFDGMTTSLCVRRAVYVDETGKEPAKKG
ICSNFLCDAKPGQEITMTGPTGKVLLLPEDPNAVIICVATGTGIAPFRTFYRRMFLENVP
NYKFTGLFWMFMGVANSDNKLYDEEMQAIAKAYPEQFRLDYALSREQKNRNGGKMYIQD
K
VEEYADEVFDLLNNGAHIYFCGLKGMMPGILEMLERVAKSKGLEYSEWFEGLKHKNQVHV
EVY
>CAA55406.1 ferredoxin NADP reductase, partial [Chlamydomonas reinhardtii]
--KIPFWE
GQSYGVIPPGTNSKEVPHGTRLYSIASSRYGDDFDGQTASLCVRRAVYVDETGKEPAKKG
LCSNFLCDATPGTEISMTGPTGKVLLLPADANAPLICVATGTGIAPFRSFWRRCFIENVP
SYKFTGLFWLFMGVANSDAKLYDEELQAIAKAYPGQFRLDYALSREQNNRKGGKMYIQDK
VEEYADEIFDLLDNGAHMYFCGLKGMMPGIQDMLERVAKEKGLNYEEWVEGLKHKNQWH
V
EVY
>KDD75893.1 hypothetical protein H632_c449p0, partial [Helicosporidium sp. ATCC 50920]
VVRRPVPTELEGIGVPMNTYSMKKPFKARVMSVDKLVGPRATGETYHVVIETRGEIPFAE
GQSYGVVPPGSNSRTVPHATRLYSIASTRYGDRFDGRTASFCIRRATFWDELGREPEKKG
ICSNFLCDAAPGTEIDLSGPTGKLLLMPDDPHADVITVATGTGIAPFRAFWRRRFFEAVP
SEQGRGHMWLFMGVANSDAKLYASEIEAVEQAHPTRFRVDYALSREQTNASGGKMYVQDR
LEQHADEVFDRLSAGAHIYFCGLKGMLPGILETLERVAGEKGIDWPAFLEGLKKEHRWHV
EVY
>OAO96703.1 RFNR1 [Arabidopsis thaliana]
KSKVLVTPLLEDKETPLNLFRPKEPYTATIVSVERIVGPQAPGETCHIVIDHDGNVPYWE
GQSYGVIPPGENPKGAPHNVRLYSIASTRYGDSFDGKTASLCVRRAIYYDETGKEPSKAG
VCSNFLCNAKPGDKVKITGPSGKVMLLPDDPKATHIMIATGTGVAPYRGYLRRMFMENVP
NFKFDGLAWLFLGVANSDSLLYDEEFAGYRKDYPENFRYDKALSREEKNKKGGKMYVQDK
IEEYSDEIFKLLDNGAHIYFCGLKGMMPGIQDTLKRVAEERGESWEQKLTQLRKNKQWHV
EVY
>OAP18471.1 RFNR2 [Arabidopsis thaliana]
SSKVTVSPILEDKDPPLNLYKPKESYTAKIVSVERVVGPKAPGETCHIVIDHDGNLPYWE

GQSYGVIPPGENPKGAPHNVRLYSIASTRYGDFFDGKTASLCVRRAVYYDETGKEPSKNG
VCSNFLCDSKPGDKIQITGPSGKVMLLPSDPNATHIMIATGTGVAPYRGYLRRMFMENVP
NKTFSGLAWLFLGVANTDSLLYDDEFTKYLKDHPDNFRFDKALSREEKNKKGGKMYVQDK
IEEYSDEIFKLLDNGAHIYFCGLKGMMPGIQDTLKRVAEERGESWDLKLSQLRKNKQWHV
EVY
>BAE98556.1 ferredoxin-NADP+ reductase [Arabidopsis thaliana]
PVKVVKESKKQEEGIVVNKFKPKNPYTGRCLLNTKITGDDAPGETWHIVFTTEGEVPYRE
GQSIGVIPEGIDKNGKPHKLRLYSIASSAIGDFGDSKTVSLCVKRLVYTNDGGEI--VKG
VCSNFLCDLKPGDEAKITGPVGKEMLMPKDPNATIIMLGTGTGIAPFRSFLWKMFFEEHE
DYKFNGLAWLFLGVPTSSSLLYKEEFEKMKEKNPDNFRLDFAVSREQTNEKGEKMYIQTR
MAEYAEELWELLKDNTFVYMCGLKGMEKGIDDIMVSLAAKDGIDWLEYKKQLKRSEQWN
V
EVY
>NP_001077566.1 ferredoxin-NADP[+]-oxidoreductase 2 [Arabidopsis thaliana]
AKKVEKVSKKNEEGVIVNRYRPKEPYTGKCLLNTKITADDAPGETWHMVFSHQGKIPYRE
GQSVGVIADGIDKNGKPHKVRLYSIASSALGDLGNSETVSLCVKRLVYTNDQGET--VKG
VCSNFLCDLAPGSDVKLTGPVGKEMLMPKDPNATVIMLATGTGIAPFRSFLWKMFFEKHD
DYKFNGLAWLFLGVPTTSSLLYQEEFDKMKAKAPENFRVDYAISREQANDKGEKMYIQTR
MAQYAAELWELLKDNTFVYMCGLKGMEKGIDDIMVSLAANDGIDWFDYKKQLKKAEQW
NV
EVY
>XP_015629836.1 ferredoxin--NADP reductase, root isozyme, chloroplastic [Oryza sativa Japonica Group]
ESKVAVKPLLESNEPPLNTYKPKEPYTATIVSVERIVGPKAPGETCHIVIDHGGNVPYWE
GQSYGIIPPGENPKGAPHNVRLYSIASTRYGDSFDGRTTSLCVRRAVYYDETGKEPSKNG
VCSNFLCNSKPGDKVKVTGPSGKIMLLPEDPNATHIMIATGTGVAPFRGYLRRMFMEDVP
KYRFGGLAWLFLGVANTDSLLYDEEFTSYLKQYPDNFRYDKALSREQKNKNAGKMYVQD
K
IEEYSDEIFKLLDGGAHIYFCGLKGMMPGIQDTLKKVAEQRGESWEQKLSQLKKNKQWHV
EVY
>XP_015646844.1 ferredoxin--NADP reductase, embryo isozyme, chloroplastic [Oryza sativa Japonica Group]
KSKVAVKPLLDNKEPPLNLYKPKEPYTATIVSVERLVGPKAPGETCHIVIDHGGNVPYWE
GQSYGVIPPGENPKGSPNTVRLYSIASTRYGDSFDGKTASLCVRRAVYYDETGKEPTKKG
ICSNFLCDSKPGDKVQITGPSGKIMLLPDDPNATHIMIATGTGVAPYRGYLRRMFMEDVP
SFKFGGLAWLFLGVANTDSLLYDEEFTNYLQQYPDNFRYDKALSREQKNKNGGKMYVQDK

IEEYSDEIFKLLDGGAHIYFCGLKGMMPGIQDTLKRVAEQRGESWEQKLSQLKKNKQWHV
EVY
>XP_015625198.1 ferredoxin--NADP reductase, leaf isozyme 2, chloroplastic-like [Oryza sativa Japonica Group]
TTKVEKVSKKQVDGVVTNKYRPKEPYTGRCLLNTRITGDDAPGETWHMVFSTDGEIPYRE
GQSIGVIPDGIDKNGKPHKLRLYSIASSAIGDFADSKTVSLCVKRLVYTNDQGEI--VKG
VCSNFLCDLKPGSDVKITGPVGKEMLMPKDPNATIIMLGTGTGIAPFRSFLWKMFFEEHD
DYKFNGLAWLFLGVPTSSTLLYREEFERMKEIAPERFRLDFAVSREQTNAAGEKMYIQTR
MAEYKDELWELLKDNTYVYMCGLKGMEKGIDDIMIDLAAKDGIDWLDYKKQLKKSEQW
NV
EVY
>XP_015640980.1 ferredoxin--NADP reductase, leaf isozyme 1, chloroplastic [Oryza sativa Japonica Group]
PAKKEKISKKHDEGVVTNKYRPKEPYVGKCLLNTKITADDAPGETWHMVFSTEGEIPYRE
GQSIGVIADGVDKNGKPHKLRLYSIASSALGDFGDSKTVSLCVKRLVYTNDQGEI--VKG
VCSNFLCDLKPGSDVKITGPVGKEMLMPKDPNANIIMLATGTGIAPFRSFLWKMFFEKYD
DYKFNGLAWLFLGVPTSSSLLYKEEFDKMKAKAPENFRVDYAVSREQTNAQGEKMYIQTR
MAEYKEELWELLKDHTYVYMCGLKGMEKGIDDIMVSLAAKDGIDWADYKKQLKKGEQW
NV
EVY
>XP_024384495.1 ferredoxin--NADP reductase, embryo isozyme, chloroplastic-like [Physcomitrella patens]
ASKVALTADLEGPEPPMHLFKNKEPFIGTVKSVERIVGPNATGETCHIVIDHGGQMPYWE
GQSYGIIPPGENPKGQPNTVRLYSIASTRYGDEFDGKTASLCVRRAVYWCELQAEPAKKG
ICSNFLCDCKPGDKVQITGPSGKVMLLPSDPNATHIMVATGTGIAPYRGFLRRMFMEDVP
TFKFGGLAWLFLGVANSDSLLYHDEFTKYKEAFPENFRYDTALSREEKNSKGGKMYVQDK
IEEYSEELFNLLDKGAHIYFCGLRGMMPGIQDTLKRVAEARGENWEEKLAKLKKNKQWHV
EVY
>XP_024356607.1 ferredoxin--NADP reductase, embryo isozyme, chloroplastic-like [Physcomitrella patens]
TPQVATGPKLEAKEPPMHLFKNKEPFIGTIKSVERIVGPKAPGETCHIVIDHEGNVPYWE
GQSYGIIPPGENPKGQPNSVRLYSIASTRYGDDFDGRTASFCVRRAVYWDETGKEPAKKG
ICSNFLCDSKPGDKVQIVGPSGKVLLLPEDPSATHIMVATGTGIAPYRGYLRRMFMEDT-
EFKFNGLAWLFMGVANTDSLLYHDEFNTYLKEYPDNFRYDIALSREQKNSRGGKLYVQDK
MEEYSEELFDKLDKGAHIYFCGLRGMMPGIQDMLKRVAESRGESWETKLAALKKNKQWH
V

EVY
>PTQ28452.1 hypothetical protein MARPO_0163s0011 [Marchantia polymorpha]
VSKVKV-PLLEEKEPPLNLWKNKAPYKGVIKSVERIVGPNATGETCHIVIDHDGNVPYWE
GQSYGIIAPGENPKGTPNTVRLYSIASTRYGDNFDGKTTSLCVRRAVYWDETQKEPAKKG
VCSNFLCDAKPGDEVQITGPSGKVLLLPEDPNAVHIMVATGTGIAPYRAYLRRMFMEDVP
NFQFGGLAWLFLGVANTDSLLYHDEFSSYKEQYPENFRYDLALSREQKNQRGGKMYVQDK
IEEYSSELFDLLDKGAHIYFCGLRGMMPGIQDTLKRVAEEQGIVWEEKLSQLKKNKQWHV
EVY
>OAE30389.1 hypothetical protein AXG93_3612s1120 [Marchantia polymorpha subsp. ruderalis]
AVKEAKVSKKNEHGLVTNVYKPKTPYEGICLTNGKIVGDDAPGETFHMVFSTEGKIPYRE
GQSVGVVPPGVDEKGKPHKLRLYSIASSAPGDFGDYKTVSLCVKRLVYTNDKGEE--VKG
VCSNFLCDLKAGDKVNLTGPVGKEMLMPTDEKATVIMLATGTGIAPFRGFLWRMFFEKHD
DYKFQGLAWLFLGVPTSSSLLYREEFEKMQQDYPDNFRLDFAVSREQTNAKGERMYIQTR
MADYAEELWQMLQDNTFVYMCGLKGMEKGIDDIMVSLAARDGIDWVEYKKTLKKGEQW
NV
EVY
>XP_024542927.1 ferredoxin--NADP reductase, embryo isozyme, chloroplastic [Selaginella moellendorffii]
AKVATTAAPLDTSDPPLNLFKPKTPYTATIKSVERIVGDKAPGETCHIVIDHGGNVPYWE
GQSYGVIPPGENPKGTPNAVRLYSIASTRYGDDFDGKTASLCVRRAVYWDETGKEPAKKG
VCSNFLCDRKPGDKVQITGPSGKIMLLPSNPKAAHIMIATGTGIAPFRGYLRRMFMEDV-
SFKFGGLAWLFLGVANRDSLLYHDEFEGYLKEYPDNFRYDIALSREQNNKRGGKMYVQDK
IEEYSEEVFKLLDEGAHIYFCGLKGMMPGIQDTLKRVAEERGESWEEKLSMLKKKKQWHV
EVY
>EFJ16776.1 hypothetical protein SELMODRAFT_179376 [Selaginella moellendorffii]
AKVATTAAPLDTSDPPLNLFKPKTPYTATIKSVERIVGDKAPGETCHIVIDHGGNVPYWE
GQSYGVIPPGENPKGTPNAVRLYSIASTRYGDDFDGKTASLCVRRAVYWDETGKEPAKKG
VCSNFLCDRKPGDKVQITGPSGKIMLLPSNPKAAHIMIATGTGIAPFRGYLRRMFMEDV-
SFKFGGLAWLFLGVANRDSLLYHDEFEGYLKEYPDNFRYDIALSREQNNKRGGKMYVQDK
IEEYSEEVFKLLDEGAHIYFCGLKGMMPGIQDTLKRVAEERGESWEEKLSMLKKKKQWHV
EVY
>XP_002985709.1 ferredoxin--NADP reductase, embryo isozyme, chloroplastic [Selaginella moellendorffii]
--MSRLLKLLERNKPPFHLYTSQLPYTATVSSIQRLT---RDGQVSHIVIDHGGNVPFWE
GQSYGILPPGENSKGTRHPYHLYSIASSRYGDDLSGRSASLCVKRAIYVDQTGEEPSKKG
VCSNFLCDCKPGDKVDLVGPFGKLMLLNSNPSDSHIMVATGTGVAPFRGFLQRLLEDKMR

PRKFEGSAWLFMGAPTAGRLLYNEEFERYARDLPWSFRYDTALSRESCNKRGGRFYVQDR
MEEHGEEIFKLLDGGSHIYFCGRKDMLVGVEAVFEEVARRMGEDWRGKLAKLKKNRQWH
V
EVY
>XP_002977684.1 ferredoxin--NADP reductase, root-type isozyme, chloroplastic [Selaginella moellendorffii]
MRRLVLPRSLELKKAPTNLYTRKSPYIATIESVHRLTTGENGGDTYHIVIDHGGNVPFWE
GQSYGILPPGTNPKGAPPPNRLYSLASSRYGDDLSGRTASLCVKRIVSYD----NPNSTG
ICSNFLCDARPGDQVRIVGPFGSSLLLNENPRGAQIMVGTGTGVAPFRGFLRRMFVEEVP
-FKFDGLAWLFLGVASSKSLLYHDEFERIARDFPSSFRYDLALSREMVDRSGGKFYVQHR
IKERGKEVLELLESGGHIYFCGREEMMEGIQETFRKLC---GDSWHEKLSGWKRNKQWHV
DVY
>XP_002993724.1 ferredoxin--NADP reductase, leaf isozyme 1, chloroplastic [Selaginella moellendorffii]
TETVEKVKKKDEEGVVTNLFRPKEPYVGRCLLNTKIVGDDAPGETWHMVFTTEGKIPYRE
GQSIGIVPPGLDAKGKPQKLRLYSIASSAPGDFGDYKTVSLCVKRLVYVNDKGEE--VKG
VCSNFLCDLKPGDEVSITGPVGKEMLMPVDPNATIIMLGTGTGIAPFRGFLWRMFFEKHD
DYKFNGLAWLFLGVPTSSSLLYKEEFEKMKEKFPNNFNLDFAVSREQTNAKGEKMYIQTR
MAEYAEQLWDLLKDNTYVYMCGLKGMEKGIDDIMTSLAAKEGIDWAEYKKQLKKGEQW
NV
EVY
>GAQ80250.1 ferredoxin-NADP reductase [Klebsormidium nitens]
QTATEVFPALEESDPPMNTAKNNAPFTGTIKSVERIVGPNATGETKHIVIDHEGKLPYWE
GQSYGIIPPGENPKGKPHTVRLYSIASTRYGDDFDGKTASLCVRRAVYVDETGKEPEKAG
ICSNFLCDSKPGDKVQITGPSGKVMLIPEDPNATHIMIATGTGIAPFRAYLRRFFMEDVP
SFKFGGLAWLFLGVANTDSLLYHDEFSKYKEEFPDNFRYDLALSREQKNKEGGKMYIQNK
VAEYGNEVFDMLDKGAHIYFCGLKGMMPGIQDSLKQIAESRGESWEEKLTQLKKNKQWHV
EVY
>GAQ83927.1 ferredoxin-NADP reductase [Klebsormidium nitens]
KAKAKKESKKDDEGISVNLFKPKEPYTGRTLLNTKIVGDDAPGETWHMVFSHEGKVPYRE
GQSIGIVPPGMDANGRPHKLRLYSIASSAPGDFGDYKTVSLCVKRLVYTNDKGEE--VKG
VCSNFLCDLKAGDEVQITGPVGKEMLMPKDPNANVIMLGTGTGIAPFRGFLWKMFFEKRS
DYKFNGLAWLFLGVPTSSSLLYREEFELMQKKHPKNFRLDFAVSREQNNAKGEKMYIQTR
MAEYAEELWGLLKDNTYTYMCGLRGMEKGIDDIMTSLAQRDGMDWAEYKKELKKDERW
SV
ETY

>EJK44714.1 hypothetical protein THAOC_36724 [Thalassiosira oceanica]
AAVRDAQHFLE-GELPLNFAKPNQPVTATVLGRTKLIDDDAPGDIEHVILKLPEGFHYVE
GQSLSLIPPGVDAKGRKHKPRLYSIASTRYGDVLDGNTISLCVRRAEYVDVTGEKPAKQG
VCSNFLCDVRAGDEVSVAGPVGKTMLLPKDSNTDIIMIATGTGIAPFRGFMHRLFMENTL
ARHFGGRAWLVLGVPVTGGLLYKEEFDCMQRNGADQLRIDYAISREMTAKTGGKMYVQN
V
IAENGREVFDRLDNGAVIYFCGLKGMMPGILDSLEEVAASQGIVWSEKLAELKKNHQWHV
EVY
>GAX27166.1 ferredoxin--NADP+ reductase [Fistulifera solaris]
SVLDDAQHWLEDIGLPSNTVKPKEPLPAQVLGRARLIGDDAPGDIQHIVLQLPAAFHYVE
GQSLSVIPPGLDTTGKPHKPRLYSIASTRYGDLLDGRTVSLCVRRAEYYDITGVAPEKQG
VCSNFLCNTQVGDVVQVAGPVGKTMLLPEDPSKDIIMVATGTGIAPFRGFLHRLFMENTV
SRHFNAKAWLILGVPVRSGLLYQEELQAMQQNSVADLEVTYAISREMKNAQGGKLYVQDV
LAARADELFSKLDHGAVIYFCGLKGMMPGILTALEQVATRQGIDWSTKLKEWQANHQWHV
EVY
>OEU11321.1 ferredoxin NADP reductase [Fragilariopsis cylindrus CCMP1102]
EMLYSSQCWLE-PYVAPTFAKATNPVKAIVLGRTSLITEDAPGDIQHIVLRLPEGFHYVE
GQSLSVIPPGLTEKGKKQKPRLYSIASTRYGDLLDGTTVSLCVRKAEYYDITGIIDSKAG
ICSKFLCSATPGTEIDVAGPIGKTMILPKDSTKDIIMVATGTGIAPFRSFMHRLFVEQTT
ARHYTGNAWLILGVPTSGSLLYKTEFDSMLQQQLNNLKINYAISREMTNKEGGKLYVQDV
LKENSIELFSKLEAGAVIYFCGLKGMMPGILLALEEVATSQGIDWSTTLKKYQSNHQWHV
EVY
>XP_002291633.1 predicted protein, partial [Thalassiosira pseudonana CCMP1335]
--------------PLNFAKPQAPATATVLGRTKLIADDAPGDIEHVIMKLPEGFHYVE
GQSLSVIPPGTDAKGRNHKPRLYSIASTRYGDTLDGTTISLCVRRAEFIDVTGEKPEKQG
VCSNFLCNVNPGDTVSVAGPVGKTMLLPEDPTKDVIMVATGTGIAPFRGFMHRLFMENTL
ARHFGGSAWLVLGVPVSGGLLYKEEFDCMLRNSPNQLRIDYAISREMTNTIGGKLYVQHV
IAQNGKELFNRLDNGAHIYFCGLKGMLPGILESLEGVAKEQGVDWTAKLSELKKNNQWHV
EVY
>VEU44392.1 unnamed protein product [Pseudo-nitzschia multistriata]
EMLHAAQCWLEDSPVPPPFCTAKSPVTATVLGRTPLIESDAPGDIQHIVLRLPEGFRYVE
GQSISVIPPGTDPSGRPHKPRLYSIASTRYGDLLDGTTVSLCVRRAEYTDATGKVPSKAG
VCSGFLCDAVPGQKVSVAGPVGKTMLLPHSGSVDLIMVATGTGIAPFRSFLHRLFTEHTP
ANHFNGKAWLILGVPTSGSLLYKPEFDAMLRTPTPSLRIDYAISREMTNASGGKLYVQDV
LRENAGELMERLENGAVIYFCGLRGMMPGILEALEEVATASGMDWAETLKKYKANHQWHV
EVY

>XP_002183949.1 predicted protein, partial [Phaeodactylum tricornutum CCAP 1055/1]
-------------------KPAAPVPAKVLSRRRLIADDAPGDVQHIIMSLPKGLHYVE
GQSLSVIPPGTNPQGRPHKPRLYSIASTRYGDDLRGNTVSLCVRRAEYVDQTRKVPTKAG
VCSNFLCDMVPGTIVQVAGPVGKTMLLPKANQQDIIMVATGTGIAPFRAFLRRLFLENTV
AKQYQGQAWLILGVSVTGGLLYADEFERMQQPWPGQLRVDYAISREMKNQQGGKLYVQD
V
LSEEAEMLWTKLEDGAIIYFCGLKGMMPGILEALEDVAKARGKVWSQTLRRLKANGQWHV
EVY
>GAX27626.1 hypothetical protein FisN_13Hh283 [Fistulifera solaris]
SVLDDAQHWLEDIGLPSNTIKPKEPVLAQVLGRARLIGEDAPGDIQHIVLQLPAEFHYVE
GQSLSVIPPGVD-QGKPHKPRLYSIASTRYGDLLDGQTVSLCVRRAEFYDITGLAPEKQG
VCSNLLCNTQVGDMVQVAGPVGKTMLLPDDPSKDIIMVATGTGIAPFRGFLHRLFIENTV
SRHFNAKAWLILGVPVRSGLLYEEELQAMQQNSAADLEVTYAISREMKNTAGGKLYVQDV
LAAGPMNCFSN--

>TFJ81270.1 hypothetical protein NSK_007231 [Nannochloropsis salina CCMP1776]
QDFLEANPYSDQSNIPLNTYKNKEPHTGKIVSVKRIVGPKATGETCHIIIDHGGAMPYWE
GQSYGVVPPGINPKGKPNNVRLYSIASTRYGDDMKGQTASLCVRRATYWCELNAEPAKKG
ICSNYLCDAKPGDSVALAGPTGKVMLIPKTPEADLIMVATGTGIAPYRTFVRRLFVEDTP
ARKFKGLAWLFLGVANKDSLLYDDEWQEVKKAYPNNFRVDYALSREQENKKGGKMYIQD
K
MEEYADEIFDRLSKGAHIYFCGLKGMMPGIQDTLERVAQEKGIEWKEMLEGLKKNHQWHV
EVY
>EWM23857.1 Ferredoxin reductase-type FAD-binding domain protein [Nannochloropsis gaditana]
QDFLEANPYFDQSNIPLNTYKNKEPHTGKIVSVKRIVGPKATGETCHIIIDHGGAMPYWE
GQSYGVVPPGINPKGKPNNVRLYSIASTRYGDDMKGQTASLCVRRATYWCELKAEPAKKG
ICSNYLCDAKPGDSVALAGPTGKVMLIPKTPEADLIMVATGTGIAPYRTFVRRLFVEDTP
ARKFKGLAWLFLGVANKDSLLYDDEWQEVKKAYPNNFRVDYALSREQENKKGGKMYIQD
K
MEEYADEIFDRLSKGAHIYFCGLKGMMPGIQDTLERVAQEKGIEWKDMLEGLKKNHQWHV
EVY
>XP_002180502.1 predicted protein [Phaeodactylum tricornutum CCAP 1055/1]
QDFLEAEPYWDQSSVPVNVYKNKAPFTGKVVSTKRIVGPLATGETCHVVIDHEGNFPYWE
GQSWGVIPPGVREKGKPHSVRLYSIASTRYGDDMTGKTGSLCVRRATYWCELKAEPTKKG
VCSNFLCDTRPGEEVQMTGPAGKVMLMPENPDTDYIMVATGTGIAPYRGFVRRLFTEKTP
AAEYKGQAWLFLGVANSDALLYDDEWQEVKTNNPNQFRLDYALSREQENKKGGKMYIQD

K
VEEYADEIFQKLDAGAHIYFCGLKGMMPGIQEMLQTVCTQKGVEYDEWLKGLKAKKQWH
V
EVY
>XP_009034033.1 hypothetical protein AURANDRAFT_52453 [Aureococcus anophagefferens]
APAAPTLDFLEYAEIPKTAFKPTAPYKSAIRSVKRAIGPDAPGEICHVVMTTEGKLPYVE
GQSVGVVPPGN--KGKPHQQRLYSIASTRYGDDGAGDSVSLCVRRAVYVDETGEEPAKKG
ICSNFLCDGSPGDVVALTGPVGKGLLLPESPDADVIMVATGTGVAPYRGFVKRLFDEQTP
ANDFTGRAWLFFGGPTSDSILYPELWDAAKASKPDQFDLTLAISREQTNEDGGRMYVQHR
IVEHADEIFDRLDNGAHFYLCGLKGMQPGIEAALEEVCDKKGLVFKDWVKALKKDKRYHV
EVY
>GAX13172.1 ferredoxin--NADP+ reductase [Fistulifera solaris]
QDFLEAEPYWDQSTVPVNVYKNKAPFTGKVVSTKRIVGPKATGETCHIIIDHNGDFPYWE
GQSWGVIPPGVREKGKPHSVRLYSIASSRYGDDMTGKTGSLCVRRATYWCELKAEPAKKG
ICSNFLCDTKPGDEIQMTGPAGKVMLIPENPNTDLIMVATGTGIAPYRGFIRRLFFEDTP
AANYKGTAWLFMGVANSDALLYDDELQEVKKKFPNNFRLDYALSREQENKKGGKMYVQD
K
VEEYADEIFTKLDNGAHIYFCGLKGMMPGIQDMLKSVAESKKIDYEEWVKELKAKKQWHV
EVY
>XP_002184856.1 predicted protein [Phaeodactylum tricornutum CCAP 1055/1]
QNFLEAEPYFDQSTVPVNVYKNKAPFTGKVVSSKRIVGPKATGETCHIIIDHNGDFPFWE
GQSWGVIPPGTREKGKPHSVRLYSIASTRYGDDMTGKTGSLCVRRATYWCDLKADPAKKG
ICSNFLCDTKPGDEVNMTGPAGKVMLLPEEPDTDYIMVATGTGIAPYRGFVRRLFTEETP
AGEYKGQAWLFLGVANSDALLYDDEWQTVLKEYPENFRLDYALSREQENKNGGKMYIQD
K
VEEYADEIFAKLDSGAHIYFCGLKGMMPGIQDMLKSVCEEKKVDYDEWLKGLKSKKQWH
V
EVY
>XP_002295321.1 ferredoxin-nadp reductase [Thalassiosira pseudonana CCMP1335]
QDFLEAEPYYSQDTVKTNTYKNKAPFTGKVVSTKRIVGPKATGETCHIIIDHNGDFPYWE
GQSWGVIPPGTREKGKPHSVRLYSIASSRYGDDMTGKTGSLCVRRATYWCELKADPAKKG
ICSNFLCDTEPGAEVMMTGPAGKVMLMPEDPKTDYIMVATGTGIAPFRSFVRRLFFEDTP
AAAYKGEAWLFLGVANSDALLYDDEFQDAKARYPENFRLDYALSREQENKNGGKMYIQDK
VEEYADEVFNKLDNGAHIYFCGLKGMMPGIQDMLAEVCKSKGLDYEEWIKELKGKKQWH
V
EVY

>EJK72001.1 hypothetical protein THAOC_06509 [Thalassiosira oceanica]
QDFLEYTPYYDHSAVKVNTHKNKAPFTGKVVSTKRIVGPKATGETCHIIIDHEGDFPYIE
GQSWGVIPPGTREKGKPHAVRLYSIASSRYGDDMTGKTGSLCVRRATYWCELQADPAKKG
VCSNFLCDTTPGDELKMTGPSGKVMLMPEDPNTDYIMVATGTGIAPYRGFIRRLFFEDTP
AADYKGQAWLFLGVANSDALLYDDEFQDAKARYPDNFRIDYALSREQENKKGGKMYIQDK
VEEYADEIFNKLDSGAHIYFCGLKGMMPGIQDMLKAVCEEKGISYDEWLKGLKQAKQWHV
EVY
>OEU22459.1 chloroplast ferredoxin dependent NADH oxireductase [Fragilariopsis cylindrus CCMP1102]
QNFLEAEPYYDQSTVPVNVYKNKAPFTGKVVSTKRIVGPKATGETCHIVIDHKGDFPYWE
GQSWGVMPPGNREKGKPHSVRLYSIASSRYGDDMTGQTGSLCVRRATYWDTLKADPAKKG
ICSNFLCDTQPGDDVAMTGPAGKVMLMPEDPSTDYIMVATGTGIAPYRGFIRRLFNEDTP
AGRYKGEAWLFLGVANSDALLYDDEFQEAKSRYPENLRIDYALSREQNNTKGGKMYIQDK
VEEYADEVFNKLESGAHIYFCGLKGMMPGIQDMLKGVAEKKGLNYDEWLKGLKKAKQWH
V
EVY
>VEU37418.1 unnamed protein product [Pseudo-nitzschia multistriata]
QDFLEAEPYWDQSTVPVNVYKNKAPFTGKVVSTKRIVGPKATGETCHIIIDHNGDFPYWE
GQSWGVIPPGTREKGKPHAVRLYSIASSRYGDDMTGKTGSLCVRRATYWCELKADPAKKG
ICSNFLCDTNPGDEITMTGPAGKVMLMPEDPNTDYIMVATGTGIAPYRGFIRRLFNEDTP
AARYKGEAWLFLGVANSDALLYDDEFQEAKSRFPDNLRIDYALSREQTNAKGGKMYIQDK
VEEYADEVFTKLENGAHIYFCGLKGMMPGIQDMLKGVAEKKGIDYDEWLKKLKKAKQWH
V
EVY
>OEU15208.1 chloroplast ferredoxin dependent NADH oxireductase [Fragilariopsis cylindrus CCMP1102]
EDFLEAEPFYDQSTVPVNVYKNKSPFTGKVVSTKRIVGPQATGETCHIIIDHDGDFPYWE
GQSWGVMPPGTREKGKPHSVRLYSIASSRYGDDMTGKTGSLCVRRANYWCELKADPAKA
G
ICSNFLCDSVPGTEVKMTGPAGKVMLMPEDPTIDYIMVATGTGIAPYRGFVRRLYAEDTP
AARYKGEAWLFLGVANTDALLYDDEFQLAAKK---GLRIDYALSREQENTKGGKMYIQDK
VEEYADEVFAKLEGGAHIYFCGLKGMMPGIQEMLKGVAEKKNVDYDAWLKGLKKAKQW
HV
EVY
>XP_002290014.1 ferredoxin--NADP+ reductase [Thalassiosira pseudonana CCMP1335]
QNFLEAEPYWDQSNVPVNVYKNKAPFTGKVVSTKRIVGPQATGETCHIVIDHQGNFPYWE

GQSWGVIPPGTREKGKPHSVRLYSIASSRYGDDFTGNTGSLCVRRATYWCELKADPAKKG
ICSNFLCDTTAGDEVMMTGPAGKVMLMPEDPKTDYIMVATGTGIAPFRGFVRRLFFESTP
AAKYQGQAWLFLGVANSDALLYDDEFQEAKSKFPDNFRLDYALSREQNNKKGGKMYIQDK
VEEYADEVFNKLNNGAHIYFCGLKGMMPGIQDMLAEVCKSKGLDYDEWIKELKGKKQWH
V
EVY
>CBJ31206.1 hypothetical protein Esi_0238_0041 [Ectocarpus siliculosus]
TRSLEREPYWDMSELPMHTFKAKAPYTGTIETVEKLVQPGASGEVFHVKLRHEGNMPYWE
GQSLGVTPPGLDAKGKPHKVRLYSIASTRYGDDGDGRTVSLCVRRATVIDETGKEPSKEG
VCSNFLCRARAGQEVTLTGPSGKIMLLPETPDADVIMVGTGTGIAPYRGFLQRLFKEDTP
AARFTGTAWLFLGVATTEGLLYHDDWMEMLRKFPFNFRCE---GREEKNASGGKMYIQDR
VEQYADEVFERLDGGAHIYFCGLKGMMPGITDMLARVAGERGISWDAKLKELKSKGQWH
V
EVY
>VEU42932.1 unnamed protein product [Pseudo-nitzschia multistriata]
EDFLEAEPYYDQSTVPVNVWKNKAPFTGKIVSTKRIVGPQATGETCHIIIDHDGDFPYWE
GQSWGVIPPGTREKGKPHSVRLYSIASSRYGDDKTGKTGSLCVRRATYWCELKAEPAKKG
ICSNFLCDTQPGDEVTMTGPAGKVMLMPEDPKTDLIMVATGTGIAPYRGFIRRLFAEDTP
AAEYKGQAWLFLGVANSDALLYDDEFQAAKKAFPENFRLDYALSREQTNVKGGKMYIQDK
VEEYADEVFEKLENGAHIYFCGLKGMMPGIQDMLKGVAEKKDIDYDEWLKKLKKAKQWH
V
EVY
>GAX17478.1 ferredoxin--NADP+ reductase [Fistulifera solaris]
QDFLEAEPYWDQTNVPVNVYKNKAPFVGKVISTKRIVGPKATGETCHIIIDHKGDFPYWE
GQSWGVIPPGVREKGKPHSVRLYSIASSRYGDDMTGKTGSLCVRRATYWCELKADPAKKG
ICSNFLCDTKPGDEVQMTGPAGKVMLLPENPSTDYIMVGTGTGVAPYRGFIRRLFVENTP
AAAYKGQAWLFLGVANSDALLYDDEWQAVKKDHPEQFRLDYALSREQTNAKGGKMYIQD
K
IEEYADEVFNKLENGAVIYFCGLKGMMPGIQEMLETVAKKKGIDYEEWIKGLKAKKQWRV
EVY
>GAX13963.1 ferredoxin--NADP+ reductase [Fistulifera solaris]
QDFLEAEPYWDQTNVSVNVYKNKAPFVGKVISTKRIVGPKATGETCHIIIDHKGDFPYWE
GQSWGVIPPGVREKGKPHSVRLYSIASSRYGDDMTGKTGSLCVRRATYWCELKADPAKKG
ICSNFLCDTKPGDEVQMTGPAGKVMLLPENPNTDYIMVGTGTGVAPYRGFIRRLFVENTP
AAEYKGQAWLFLGVANSDALLYDDEWQAVKKEHPEQFRLDYALSREQTNAKGGKMYIQD
K

IEEYADEVFNKLENGAVIYFCGLKGMMPGIQEMLETVAKKKGIDYEEWIKGLKAKKQWRV
EVY
>OEU17081.1 ferredoxin NADP reductase [Fragilariopsis cylindrus CCMP1102]
QEFLEATPYYDQSSVPVNVYKNKAPFTGKVVSTKRIVGPKATGETCHIIVDHKGDFPYWE
GQSWGVIPPGTREKGKPHSVRLYSIASSRYGDDMTGKTGSLCVRRATYWDELKADPAKKG
ICSNFLCDTQPGDDVVMTGPAGKVMLMPEDPSTNYIMVATGTGIAPYRGFIRRLFNEDTP
AARYKGEAWLFLGVANSDALLYDDEFQEAKTRFPENLRIDYALSREQNNTKGGKMYIQDK
VEEYADEVFNKLESGAHIYFCGLKGMMPGIQDMLKGVAEKKGLNYDEWLKGLKKAKQWH
V
EVY
>VEU42375.1 unnamed protein product [Pseudo-nitzschia multistriata]
QDFLEADPYWDQSTVPVNTFKNKAPFTGKVVSTKRIVGPKATGETCHIVIDHNGDFPYWE
GQSWGVIPPGTREKGKPHAVRLYSIASSRYGDDMTGKTGSLCVRRATYWCELKADPAKKG
ICSNFLCDTKTGDEVTMTGPAGKVMLMPQDPTTBYIMVATGTGIAPYRGFIRRLFNEDTP
AARYKGEAWLFLGVANSDALLYDDEFQEAKSRFPENLRIDYALSREQTNSKGGKMYIQDK
VEEYADEVFSKLENGAHIYFCGLKGMMPGIQDMLKGVADKKGLNYDEWLKKLKKNKQW
HV
EVY
>XP_009040433.1 hypothetical protein AURANDRAFT_31888 [Aureococcus anophagefferens]
QDFLEASPYWDQSTVPVNTYKNKSPYTTKVVSCKRIVGPEATGETCHIIFDHQGKMPYWE
GQSFGVIADGTNPKGKPHTVRLYSIAASRYGDDMTGKTTSLCVRRATYWDEMGKDPAKKG
VCSNFICDSKPGDAVKMTGPSGKVMLMPEKADTDYIMVATGTGIAPYRSFIRRLFTETTP
AGEYKGTAWLFLGVANSDALLYDEEWQATLAKYPDNFKLDYALSREQSNSKGGKMYIQDK
VEEYADEIFDRLGKGAVMYFCGLKGMMPGIQDMLKSVCDKKGLDYDEYIKDLKKKGQWR
V
EVY
>XP_009035092.1 hypothetical protein AURANDRAFT_23206 [Aureococcus anophagefferens]
QDFLEASPYWDQSTVPVNTYKNKSPYTTKVVSCKRIVGPEATGETCHIIFDHQGKMPYWE
GQSFGVIADGTNPKGKPHTVRLYSIAASRYGDDMTGQTTSLCVRRATYWDEMGKDPAKKG
VCSNFICDSKPGDPIKMTGPSGKVMLMPEKADTDYIMVATGTGIAPYRSFIRRLFTETTP
AGEYKGTAWLFLGVANSDALLYDEEWQATLAKYPDNFKLDYALSREQTNTKGGKMYIQDK
VEEYADEIFDRLGKGAVMYFCGLKGMMPGIQDMLKGVCDKKGLDYDEYIKGLKKAGQWR
V
EVY
>CBN78345.1 ferredoxin-NADP oxidoreductase [Ectocarpus siliculosus]
LDFLEADPYFDQSNVPVNTYKAKDPMIGKVVSVKRIVGPEATGETCDVVISHGGKMPYWE

GQSYGVIPPGNNWKGKPNGVRLYSIASSRYGDDMTGTTTTLCVRRATYWDEMGKEPAKKG
VCSNYLCDAEPGAKLKLTGPSGKVMLMPDKPETDLIMIATGTGIAPYRSFVRRLFAEATP
AKEYKGQAWLFLGVANSDALLYDAEWQQVLKEFPDNFRLDYALSREQENKSGGKMYIQD
K
VEEYGDEVFQKLSKGAHIYFCGLKGMMPGILNMLEKVATKKKMNWETTLKDLKKKEKWM
N
KRT
>Polytomella magna
VSKRDVAPGLEGGKLPLNTYSPKKPFKAKILSVEKIVGPKATGETFHVVLDTQG-VKYQE
GQSFGIIPPGTNSREVAHGTRLYSIASSRYGDNFEGNTATLCVRRAVYVDKTGKEPEKKG
ICSNYLTSTPSGTEVTLTGPTGKVLLLPEDPSVPVICVATGTGIAPFRSFWRRCFVENIP
GYKFTGKLWLFMGVANSDSKLYDDELQALSKAHPEQFRLDYALSREQQNKKGQKMYIQDK
VEEYADELFNLLDNGAHIYFCGLKGMMPGIQGMLERVAKEKNLVYAEWVEKLKHKNQWH
V
EVY
>chlamydomonad sp. NrCl902
IVKSDVPLELEKGPMPLNTYNNKKPFKATIKSVQKIVGPKATGETYHIVINHDGKLPFWE
GQSYGIIPPGSNSKEVAHGVRLYSIASSRYGDNFDGKTTTLCVRRAVYTDETGKEPAKKG
LCSNFLADAAPGTELNVTGPTGKILLMPEDPNAVLICVATGTGIAPFRSFYRRCFYENVP
NYKFNGTFWLFMGVANSDSKLYDDELKALLATHPKQFRLDYALSREQKNLKGGKMYIQDK
IEEYADEVFGLLDKGAHMYFCGLKGMMPGITDMLERVAKEKGIDYAQWQEKLKHNNQWH
V
EVY
>Monotropa hypopitys
KPKVSISPLLEDNEPPLHLHKPKEPYTATIVSVERLVGTKAPGETCHIVIDHGGNVPYWE
GQSYGVIPPGENPKGTPQNVRLYSIASTRYGDFFDGKTASLCVRRAVYYDETGKEPSKKG
VCSNFLCDSMPGDKIKITGPSGKIMLLPEDPNATHIMIATGTGVAPFRGYLRRMFMESVS
TFKFGGLAWLFLGVANTDSLLYDEEFTKYLQDYPNNFRFDRALSREQKNKGGGKMYVQDK
IKEYSDEIFKRLDGGAHIYFCGLKGMMPGIQDTLKRVAEERGESWENKLSQLKKNKQWHV
EVY
>Nitzschia sp.
GGTVSAQAWLEDLGVPSPFAKNTKPVTATVLGRAKLISDDAPGDIQHVLLKLPEGMHYVE
GQSISVIPPGVDEKGKAHKPRLYSIASTRYGDLLDGNTVSLCVRRAQYKDATGQIDSKAG
VCSNFLCNSEPGTTVQVAGPTGKTMLLPEDPNTDVIMVATGTGIAPFRSFLHRLFVENTP
ARHFNGHAWLILGVPVTGGLLYPEELDAMKTTAGSQLDVTYAISREMQNKQGGKLYVQDV
LSEQADVLFAKLQAGANIYFCGLKGMMPGILESLEKVATEKGLDWSKTLTHYKNNHQWHV

EVY
>Spumella sp.
SPSLEADPYWDQMRIPMNTYTIAQPFQAKVAAVRPLVNANATAEFYEMVIEHGGRMPFWE
GQNVGIIPPGTDPEGLPLPTRLYTVASTRYGDDTRGTSMTLCFKRCLREKTEPIEPAKYG
VCSDYMSNLRPGDSVLMTGPAGREILLRDKPESDIIMVATGTGVAPFRAFVRRLLVEDNP
ASRFSGLAWLFLGATTQDSVLYHDLWTSLTAAHPDRFRYTLALSRDQTNAQGGKMYIQDQ
VQLHSEEIFERMAKGAVLYCCGRRAMMPSIMANLDLHATSRGLKWQKVFESWVRNEQLKI
EVY
>CEM11286.1 unnamed protein product [Vitrella brassicaformis CCMP3155]
QDFLEASPYYDVSNIPVNTYGKKAPCIGKVVSVKRIVGEKATGETCHIIIDHDGKMPYWE
GQSYGVIPPGINPKGKPQGVRLYSIASTRYGDDMTGNTASLCVRRAVYNDETGEEPEKKG
VCSNYLCDAQPGDELKLTGPAGKVMLMPDKPAADLIMVATGTGIAPYRGFLRRLFVEQTP
ARDFKGLAWLFLGVANSDSLLYDEEWQSILKEHPDKFRLDYALSREQNNKKGGKMYIQDK
MEEYAEEIFDRMDKGAHMYFCGLKGMMPGILEMLKGVAESKGKTWEDVLKSWKENKQW
HV
EVY
>ABF73016.1 plastid ferredoxin NADPH reductase protein precursor [Karenia brevis]
QDFLEFEPYTDRSTMPDNTFKPKSPFEGKIISVERIVGPKATGETCNVVIDHFGKMPYIE
GQSYGVIPPGLNPKGKPNKVRLYSIASSRYGDDTKGTTTTLCVRRATYWCEMEKEPAKKG
VCSNYLCDASAGDVVKLTGPTGKVMLLPDKPETDIIMVATGTGIAPYRSFLKRMFIEKTP
FAKFKGLAWLFLGVANSDALLYDEDWKAIEKENPDNFRYDVALSREMTNKDGGKMYIQDK
VEEYGDEVFDRLDKGAHIYFCGLKGMMPGIQGMLEKVAGAKGLNYEEFIKKLKKNGQWH
V
EVY
>XP_002788137.1 ferredoxin--NADP reductase, putative [Perkinsus marinus ATCC 50983]
ARRKPKIISSRGRSVEVNKHKLKHPLQAVCLSNTPATREAKPADVRHIVLDTGGDLEYLE
GQSVGIIPPGA-------KTKVYSIASAGQGDLSDGTTVSLCVKRLVEVDDYGEVEDYRG
VGSNYLCDLKPGDTVAITGPTGKELLLPDDPDSKVLMLATGTGIAPFRGFLTSEFCRGID
---GEGKLWLILGVADRSSILYPQELSDCVSANSDRLRVDYALSREEKDEQGRKMYIQSK
MRQCGEDIWRWLQPNFHLYMCGMKAMESGVHEALSEICASHGSDWKEILARMRKEGRYH
A
EVY
>AKR52926.1 chloroplast ferredoxin-NADP+ reductase [Polykrikos lebourae]
TKAPAGTRVVEGRSIPWNMWSVKAPLPADVVENTTTQTGDANWETCHITFNHRGEYSYVE
GQSLGIIAPGPDKKESPAKIRLYSIASSATGDDETSKTVSLCVKRVVEVDEVGEDPDYRG
VASNHICDARPGDTVQITGPTGAEMLLPEIDDPNIVMLATGTGIAPMRSYLRYLFHDKAG

GERFKGLAWLFLGVPYKASLLYDAEHQDYVKRFPDQFRFDYAVSREDKNAAGQKMYIQTK
MAEYGEELWELLKPKTHFYLCGLKGMEAGMAEVFGPIAEKNGIVWADYFKQLKKEHRMH
V
EVY
>AAW79314.1 chloroplast ferredoxin-NADP{+) reductase, partial [Heterocapsa triquetra]
ATKKKGVKVVHGKEIPWNIFMPKAPYTGKVVANDTEDTGDANWETTHLTFDHDGKVPYLE
GQSIGIIAPGPDKKETPAKIRLYSIASSAVGDDQTSKTVSLCVKRVVELDEVGEDPDYRG
VCSNHICDMSVGDDVLITGPTGAEMLLPEDPKANIIMLATGTGIAPMRSYMRLLFHDKAG
GSRFQGLAWLFMGVPYSKSLLYDDEHLAYVKNYPDQFRFDYAVSREQTNAAGQKMYIQTK
MAEYTDELWELMQENTHIYMCGLKGMEAGMEECFSAKAEAAGLVWKEFAKSMKKADRY
HV
EVY
>PHJ21926.1 ferredoxin nadp+ oxidoreductase fnr [Cystoisospora suis]
ESSTFQSLEHLDSDVPINTFRPSAPLLCRVVSVLPATGEGRAPEIYTVVLHHACRLRFVE
GQSIAIRPKPSGRARRRDSPRIYSIASSRYGDDGTGSTLTLCVKKHVYTDLTGERPSKDG
VCSTFICNAKPGDEFEVTGPMGKTLLLPKQEDAPLVMLATGTGVAPFRGHIQRLRRMAAG
HTSRQPRILLFVGARTAEAVPYLNEWKACSSDESSGVELHLALSRQMHREDGKRLYIQDL
VWRERAKVWKALQDGGHLYVCGLKSMLSGVEDVLARVARECAGSGMDFVRQLKQERRW
HV
EVY
>XP_018638092.1 ferredoxin NADP+ oxidoreductase FNR [Toxoplasma gondii ME49]
TDQTSVDAKADELRVAVNTFRPASPLICRVVSVTPVTSKDSSPQVFSIVLHHGKQLPFVE
GQGIGIMPPSRAAQKRRLLPRIYSIASSRDGDDGCGSTLTLCVKKHIYADVTGKRRQKDG
ICSTYICDAKCGDEVEVTGPVGKTLLLPTSTETPLVMLATGTGVAPFRSHLQRLLSAGGP
AQPNRPKVLLFIGARTAAAVPYMNEWRDIEAQRDGNFDIHFALSRQMKNPQGKKLYIQDV
VWQEREKVWKALDDGGHLYACGLKNMMVGVHEVLGNMAEEKGLPRDHLASLLKHQRR
WHV
EVY
>XP_966214.1 ferredoxin--NADP reductase [Plasmodium falciparum 3D7]
RNKNFKLKNNKEENNFINLYTVKNPLKCKIVDKINLVRPNSPNEVYHLEINHNGLFKYLE
GHTCGIIPYYNDNNKKQRCARLYSISSSNNMEN-----LSVAIKIHKYEQTENAPITNYG
YCSGFIKNLKINDDIYLTGAHGYFNLPNIQKNTNFIFIATGTGISPYISFLKKLFAYDKN
RNSYTGYITIYYGVYNEDSILYLNELEYFQKMYPNNINIHYVFSYKQ-NSDATSFYVQDE
IYKRKTEFLNLFNYKCELYICGHKSIRYKVMDILKSHDQ----------FDEKKKKRVHV
EVY
>XP_005835894.1 hypothetical protein GUITHDRAFT_162347 [Guillardia theta CCMP2712]

LEFLEAKPYWDQSGVQVNIAKQKNPLIGKIISVQRIVGPNSPGETCNIIIDHQGQAPLLG
GPVLRCRPPGIDPKNKPYGVRLYSIASTRYGDDKTGKTTTLCVRRATYWCELKAEPAKKG
VCSNYLCDSKPGDEISLTGPSGKVMLMPDDPNMTYIMVATGTGIAPFRSFLRRLFGEGNP
AGKFKGLAWLFLGVANKDSLLYDEEFQVYLRQNPDKMRLDYALSREPLNKKGGKMYIQDK
VEEYADEVFDALDKGAHIYFCGLKGMMPGIQDMLRGVCESKGLNFEEYLEGLKKKGQWH
V
EVY
>KOO53489.1 ferredoxin-NADP reductase [Chrysochromulina sp. CCMP291]
QNFLEADPYWDQTNIPLNTYKNKAPFVSKVISVKRIVGPQATGETCHIIMNHGGKMPYWE
GQSYGVIPPGINPKGKPNTVRLYSIASSRYGDDMTGTTTSLCVRRATYWCELKADPAKKG
VCSNFLCDAKPGDELMLTGPSGKVMLIPKDPNVDLIMVATGTGIAPYRSFIRRLFVEKTP
YGEYKGLAWLFLGVANADALLYDDEWQEVLKKYPKNFKVDYALSREQKNVDGGKMYIQD
K
VKEYADEVFTRMDNGAHMYFCGLKGMMPGITEMLEEVSSKKGLVWEDKLKEWKEKGQW
HV
EVY
>XP_005790923.1 hypothetical protein EMIHUDRAFT_361737 [Emiliania huxleyi CCMP1516]
QDFLEAEPYWDQSTIPVNTYKNKAPFVGKIVSTKRIVGPEATGETCDVVMSHGGKMPYWE
GQSYGVIPPGTNPKGKPNSVRLYSIASSRYGDDMTGTTTTLCVRRATYWCELKADPAKKG
VCSNYLCDSKPGDDVKLTGPSGKVMLIPKDPNVDLIMVATGTGIAPYRSFIRRLFVEETP
FGEYSGLAWLFLGVANSDALLYDDEWQSVLKAHPKNFRLDYALSREQKNKDGGKMYIQDK
VAEYSDEIFTRMDNGAHMYFCGLKGMMPGITEMLEGVCKEKGLKWEDKLTQWKKAGQW
HV
EVY
>XP_005770769.1 hypothetical protein EMIHUDRAFT_432385 [Emiliania huxleyi CCMP1516]
QDFLEAEPYWDQSTIPVNTYKNKAPFTGKIVSTKRIVGPEATGETCDIVIGHEGKMPYWE
GQSYGVIPPGTNPKGKPNSVRLYSIASSRYGDDMTGTTTTLCVRRATYWCELKADPAKKG
VCSNYLCDSKPGDDVKLTGPSGKVMLIPKDPNVDLIMVATGTGIAPYRSFIRRLFVEKTP
FGEYTGQAWLFLGVANSDALLYDDEWQEVLKKYPDNFRLDYALSREQKNKDGGKMYIQD
K
VAEYSDEIFSKMDNGAHMYFCGLKGMMPGITEMLEGVCKDKGLVWEDKLSEWKKAGQW
HV
EVY
>AAW79315.1 chloroplast ferredoxin NADP(+) reductase, partial [Isochrysis galbana]
QDFLEASPFWDQSNIPINTYKNKAPFTGKIISVKRIVGAAATGETCDIVMSHGGKMPYWE
GQSYGVIPPGTNPKGKPNSVRLYSIASSRYGDDMTGTTTTLCVRRATYWCELKADPAKKG

VCSNFLCDSKPGDEVKLTGPSGKVMLIPQDATADLIMVATGTGIAPYRSFIRRLFVEKTP
YGEYKGLAWLFLGVANKDALLYDDEWQSVLKSYPKNFRVDYALSREQTNKDGGKMYIQD
K
VAEYSDEIFTRMDNGAHMYFCGLKGMMPGITEMLEGVSKAKGIVWEDKLKEWKSKGQWH
V
EVY
>KOO34261.1 hypothetical protein Ctob_009005 [Chrysochromulina sp. CCMP291]
QGFLEFDPYFDKTNLPKNTFKAKAPFVAKVISTKKISGPKANGETWDVVLSHGGDMPYIE
GQSYGVIPPGVNPKGKPNSVRLYSIASSRYGDDMTGKTTTLCVKRALYWDELKAFPAKKG
ICSNYLCDLKPGDEVLLTGPAGDVMLLPENPNADIIMLATGTGIAPMRTFLRRLFFEDTP
YARFKGLAWLFFGVYNSDALLYEDEWQTILKKHPGRFRYDKAISEEMKNKDGGNMFVQHK
MEEYADEIFDRLEKGAHMYLCGLRGMLPGVQESLKKVAEAKGIDYDEFLKNLKEKGQWH
V
EVY
>KOO34449.1 ferredoxin--NADP+ reductase [Chrysochromulina sp. CCMP291]
PSHLEFEPYFDISTMPLNTFKNKAPHTGTIVSAKRIVGDKAPGEVCHVHIKSGAVFKYIE
GQSLGVIPPGNCPKGKPNTVRLYSIASTRYGDDLDGESVSLCVRRAVYWDDLGREPAKKG
VCSNYLCDAKPGQLVTLTGPTGKVMLMPATPEADLIMVATGTGIAPYRGFLRRLFIEQTP
AAAFRGLAWLVLGVPTSDGLLYDEDWQAIAKRHPKHFRVTYAISREQETADGRKMYVQDR
LAESAQELFERLDKGAHIYFCGLKGMMPGIVETLEKVAASKGVDWDEKLEHLKKNGQWH
V
EVY
>XP_005789289.1 hypothetical protein EMIHUDRAFT_42969, partial [Emiliania huxleyi CCMP1516]
---LECDPYHAAANLPLNTYKNKAPLAGAIISAKRIVGPAAPGEVCHIRIGTGDNFRYWE
GQSLGVIPPG------PNAVRLYSIASTRYGDDAKGSSVSLCVRRALYWDDTGAHPAKKG
VCSNFLCDSSPGDEVHLTGPTGKVMLMPHEPATDLIMLATGTGIAPYRGFLRRLFLEPTP
AADFRGLAWLFLGVANRDALLYDDEWQAILQRSAAQGREDGARRRSATRPVGGKMYIQDK
VAQHADEVFERMDNGAHMYFCGLKGMMPGITEMLEGVCKEKGLVWEDKLSEWKKAGQ
WHV
EVY
>OSX71405.1 hypothetical protein BU14_0537s0012 [Porphyra umbilicalis]
GSATLRMAATKRANVPLNLFRPKTPYEATVLYNARIVGKDAPGETVHLIFQHDGNVPYLE
GQSIGVMAPGLDAKGKPHKVRLYSIASTRYGDFGDGKTVSLSVKRLLYNDDEGKE--VKG
VCSNFLCDLAPGDKVSITGPVGTAMLMPEDPNATVIMLATGTGIAPFRTYMRRAFTEKHS
DYKFTGKMWLFLGVPTSSTLLYQTEFEEMMHNYPDQLRCDWAISREQTDADGNKMYLQT

R
MKEYAEELYQLVTSKAYIFLCGLKGMTAGIDEMFGELFKKDGLDWNEYRKAMKKEGRYEA
EVY
>ADM64306.2 ferredoxin-NADP+ reductase [Pyropia yezoensis]
------MAATKRTDVPLNLFRPKAPYEATVLYNARIVGKDAPGETMHMIFNHDGNVPYME
GQSIGVIAPGVDAKGKPHKVRLYSIASTRYGDFGDGKTVSLSVKRLVYEDADGKE--VKG
VCSNHLCDLTPGDKVHITGPVGTAMLMPEDPNATIIMLATGTGIAPFRTYMRRAFTEKHA
DYKFTGKMWLFLGVPTSSTLLYQTEFEEMMHNHPDQLRCDWAISREQTDAEGNKMYLQTR
MKEYAQELYELVTAKAHIFLCGLKGMTAGIDEMFGELFEKDGLNWNDYRKAMKKEGRYEA
EVY
>XP_005706517.1 ferredoxin--NADP+ reductase [Galdieria sulphuraria]
YGVRMAAPASSKREVPLNIFRPNKPFEATCLSNELIVDKDAPGETWHMVFNTDGALRYIE
GQSIGVIPPGSDDKGKPHKVRLYSIASTSHGDHKDDKTLSLCVKRLVYTDSTGEE--RRG
VCSNYLCDLKANDKVNISGPVGTVMLMPEDQKANIIMLATGTGIAPFRAFLLRAFMENNP
DYRFEGKMWLFFGVPTTSSLLYYDELEKMAKENPNHLKIDYAISREQKDANGKKMYIQNR
MAEYKEELYELFHPNTYIYMCGLKGMESGLDEVMGPVFEERGQDWQEFRKQLKKEKHLLI
ETY
>XP_005719376.1 Ferredoxin-NADP+ oxidoreductase [Chondrus crispus]
PAARINMATIDKSKIPLNVYRPKNPYTAKVLYNERIVGEDAPGETMHMIFNHDGNVPYLE
GQSIGIVAPGTDKKGRPHKPRLYSIASTQHGDFGDGKTVSLSVKRLVYQDADGNE--VKG
VCSNHLCDLKAGDDVKISGPVGVTMLMPTDPNATLIMLATGTGVAPFRAFMRRAFSEDNP
DYKFTGTMWLFLGVPTASTLLYQQEFEEMKANFPENVRLDYAISREQTDPDGNKMYLQNR
MKEYEDELYELFQDNTYVYMCGLKGMESGIDGFMTSRFERDGKDWIAYRKSMKKAKRWE
V
ETY
>PXF41619.1 Ferredoxin--NADP reductase, cyanelle [Gracilariopsis chorda]
RAATIRMASRDRSKIPINLFRPKNPYVATVVYNERIVGEDAPGETKHMIFNHDGNVPYLE
GQSIGVIAPGLDGKGKPHKVRLYSIASTRHGDFGDGKTVSLSVKRLVYTDDDGNE--VKG
VCSNHLCDLKAGDKVQISGPVGTAMLMPEDPNATIIMLATGTGVAPFRTFMRRAFAENNS
DFKFTGTMWLFLGVPTSSTLLYQEEFEEMKANYPGQVRLDYAISREQQDADGKKMYLQNR
MKEYEEELYELFQENTYVYMCGLAGMEGGIDEFMSARFEKDGRDWNEYRRSMKKAGRW
EV
ETY
>XP_005538366.1 ferredoxin-NADP+ reductase [Cyanidioschyzon merolae strain 10D]
RPATLRMVAAE-KKVPVNLYKPNDPLIGTCIYNKKIVGDDAPGDTCHVIIHHDGKLPYLE
GQSVGIIPEGTDEKGRPHKLRLYSIASTAAGDFGDYKTLSLVVKRLVYTNEKGEE--VRG

VCSNYLNDIKPGTPVKMTGPVGKEMLMPDDPNATIIMLATGTGIAPFRAFMRKAFVEKHA
DYQFKGKMILYLGVPTSSSLLYRDELEEMKANAPDQVELHYAISREMKNKQGGKYYLQDA
MAERGEEIWQLLQDNTYVYMCGLKGMDSGIDAFMKDLAAKDGVDWATFKKQLKQQHRY
NV
EVY
>BAF42337.1 ferredoxin-NADP+ oxidoreductase [Cyanidium caldarium]
GSATLHMVAAE-KKVPVNLFKPSSPLVGTCIYNKKIVGDDAPGDTCHVIIHHDGKLPYLE
GQSVGIIPEGTDDKGRPHKLRLYSIASTAAGDFGDYKTLSLVVKRLVYTNEKGEE--VRG
VCSNFLNDIKPGEPIKMTGPVGKEMLMPDDPNATIIMLATGTGIAPFRAFMRKAFVEKHA
DYQFKGKMILYLGVPTSSSLLYRDELEEMKANFPDQVELHYAISREMKNKQGGRYYLQDS
MAERGEEIWQLLRDNTYVYMCGLKGMDSGIDSFMTDLAAKDGVNWADFKKQLKQQHRY
NV
EVY

Dataset of plant type MMT

>BAB62076.1 APG1 [Arabidopsis thaliana]
RFIQHKKEAYWFYRFLSIVYDHVINPGHWTEDMRDDALEPADLSHPDMRVVDVGGGTGFT
TLGIVKTVKAKNVTILDQSPHQLAKAKQKEPLKECKIVEGDAEDLPFPTDYADRYVSAGS
IEYWPDPQRGIREAYRVLKIGGKACLIGPVYPTFWLSRFFSDVWMLFPKEEEYIEWFKNA
GFKDVQLKRIGPKWYRGVRRHGLIMGCSVTGVKPASGDSPLQLGPKEFLLGTLAAAWFVL
IPIYMWIKDQ

>XP_015647024.1 2-methyl-6-phytyl-1,4-hydroquinone methyltransferase 1, chloroplastic [Oryza sativa Japonica Group]
RFIQHKKEAFWFYRFLSIVYDHVINPGHWTEDMRDDALEPAELYHHGLKVVDVGGGTGFT
TLGIVKHVDNENVTLLDQSPHQLEKARQKVALNGVNIIEGDAEDLPYPTDTFDRYVSAGS
IEYWPDPQRGIREAYRVLKLGGVACLIGPVHPTFWLSRFFADMWMLFPKEEEYIEWFQKA
GFQDVKIKRIGPKWYRGVRRHGLIMGCSVTGVKRSSGDSPLQLGPKAFVMGTICASYYVL
VPIYMWMKDQ

>GAQ88660.1 MPBQ/MSBQ methyltransferase [Klebsormidium nitens]
RFIQHKQEAFWFYRFLSIVYDHVINPGHWTEDMREDALEVADLNDPNLKVVDVGAGTGFC
TQGVVRTIPGKNVTMIDQSPHQLAKARKKPELQDVTILEGDAEDLPFATDTFDRYVSAGS
IEYWPDPQRGIVEAYRVIKPGAKACLIGPVHPTHPISRFFADQWMLFPTEAEYMEWFTKA
GFEDVQIKRIGPSWYRGVRRHGLIMGCSVTGVKSNPGESPLNLGPKALILGSLAGAYYVI
IPIYMWLKNL

>XP_001692712.1 MPBQ/MSBQ methyltransferase [Chlamydomonas reinhardtii]
RLVQHKSEAYWFYAGLSQVYDHIVNPGHWTEDMRDDALAPAKLDDPNLKVVDVGGGTGF
C
TLGVVKTVKPENVTLMDQSPHQLAKAKAKPALKGVTILEGDAEDLPFPTDTFDRYVSAGS
IEYWPEPQRGIREAYRVVKEGGLACMIGPVHPTHPVSRFFADAWMLFPTEEEYIEWFTKA
GFTDVKMTRIGPKWYRGVRRHGLIMGCSVTGVKPKAGDSPLVMGPKALILGTAAGFYYFC
LPIYMYIKNL

>XP_005650939.1 S-adenosyl-L-methionine-dependent methyltransferase [Coccomyxa subellipsoidea C-169]
RFIQHKKEALFFYAFLSQVYDYIVNPGHWTVDMRTEALEPAKLDNPKLKVVDVGGGTGFC
TQGIVKTISPINVVLMDQSPQQLAKAKKKKDLQGVTIVEGDAEDLPFPTDSFDRYVSAGS
IEYWPEPQRGIKEAYRVIKEGGLACMIGPVHPTFWLSRFFADMWMLFPTEDEYREWFTKA
GFTDVKIKRIGPKWYRGVRRHGLIMGCSVTGIKPKAGDSPLEMGPKVLIIGSLAGFYYFV
LPVYMWLKNL

>KXZ49108.1 hypothetical protein GPECTOR_23g39 [Gonium pectorale]
RFIQHKNEAKAFYAGLSQVYDHIVNPGHWTEDMREDALVPAKLDDPNLKVVDVGGGTGFC

TLGIVKSINPSNVTLIDQSPHQLAKAKAKPALKEVTILEGDAEDLPFATDSFDRYVSAGS
IEYWPEPQRGIREAYRVIKEGGVACVIGPVYPTFWLSRFFADVWMLFPKEEEYIEWFTKA
GFTDIKITRIGPKWYRGVRRHGLIMGCSVTGVKPKAGDSPLVMGPKALILGTAAGFYYFC
VPVYMYLKNL
>PRW18401.1 MPBQ MSBQ methyltransferase [Chlorella sorokiniana]
RLIQHKNEAKAFYAFLSQVYDYIVNPGHWTTDMREDALQPAQLDSPDLKVVDVGGGTGFC
TQGIVKAVLPTNITLIDQSPQQLDKARGKADLQGVTILEGDAEDLPFPADSFDRYVSAGS
IEYWPEPQRGICEAYRVIKPGGLACMIGPVHPTHPVSRAMADLWMLFPTEEEYLQWFKAA
GFEDVQLKRIGPSWYRGVRRHGLIMGCSVTARKPAGGDSPLQLGPKAVVLGSVGGFWYFL
LPVYMWLKNL
>XP_002952409.1 hypothetical protein VOLCADRAFT_81811 [Volvox carteri f. nagariensis]
RLIQHKNEAYWFYAGLSQVYDHIVNPGHWTEDMREAALVPAMLDNPDLKVVDVGGGTGF
T
TLGIVKAIKPGNVTLIDQSPHQLAKAKAKPALKGVTILEGDAEDLPFRTDSFDRYVSAGS
IEYWPEPQRGIKEAYRVIKEGGVACVIGPVHPSFWLSRFFADVWMLFPKEEEYIEWFTKA
GFVDVKITRIGPKWYRGVRRHGLIMGCSVTGVKPKAGESPLVMGPKVLIMGTAAGFYYFC
VPIYMYLKNL
>KAA6422115.1 MPBQ MSBQ methyltransferase [Trebouxia sp. A1-2]
RLIQHKEEAFWFYRFLSIFYDTIVNPGHWTEDMRTDALAVAELNDANLKVCDVGGGTGFC
TLGVVETVKPENVTLVDQSPHQLSKAKAKPALQRVTIKEGDAEDLPLPTDGFDRYVSAGS
IEYWPDPQRGIKEAYRVVKSGGLACLIGP--------------------------WFTRA
GFEDVQIKRIGPKWYRGVRRHGLIMGCSVTGRKTKDGDSPLQLPPKPLLLGSTAGFYYFV
LPVYMWIKDK
>Monotropa hypopitys
RFIQHKKEAFWFYRFLSIVYDHIINPGHWTDDMRDDALEPADLTDRNLVVVDVGGGTGFT
TLGIVKHVDAKSVTILDQSPHQLAKAKEKEPLKECTIIEGDAEDLPFPTDYADRYVSAGS
IEYWPDPQRGIREAYRVLKLGGKACLIGPVYPTFWLSRFFADVWMLFPKEEEYIEWFEKA
GFKDVKLKRIGPKWYRGVRRHGLIMGCSVTGVKSASGDSPLQLGPKVFVLGATAAMYYVL
VPVYMWLKDQ
>OSX72407.1 hypothetical protein BU14_0438s0002 [Porphyra umbilicalis]
GLIQHKREAFWFYRFLSIVYDTIVNPFHWTVGMRDKSLAQAGLASRNLVTVDVGGGTGFN
TEGVLAYVDPQYVTLLDQSPHQMAKAKAKPALQGVTFVEGDAENLPFATGYADRYTSAGS
IEYWPEPQRGIAEAYRVLKPGGKATIIGPVRATNWFSRFWCDLWMLFPMESEYRKWYEAA
GFYDIQVGYIGPEAYKGVRSHGLIMGLTITGTKPMNGEPLCQLGPMEWIIGLIAGGYYFV
LPFCVMLYAA
>XP_005711647.1 Putative uncharacterized protein [Chondrus crispus]

GLIQHKREAFWFYRFLSIVYDTIVNPFHWTKEMRDRSLTQAMLECRDLKTVDVGGGTGFC
TEGVAQYIDTKNITLLDQSPHQMAKAKAKESLKGVTFVEGDAENLPFQSGEFDRYTSAGS
IEYWPEPQRGIKEAYRVLKPGGVATMIGPVRATNWFSRFWCDLWMLFPMESEYIKWFTAA
GFTNLEVSYIGPSAYKGVRQHGLIMGLTVTGTKPAQGESPLQMGEMLWFIGVLAGGYYFV
LPFLIILYAA
>PXF43311.1 2-methyl-6-phytyl-1,4-hydroquinone methyltransferase 2, chloroplastic [Gracilariopsis chorda]
GLIQHKREAFWFYRFLSIVYDTIVNPFHWTKEMRDRSLAQAQLECRDLKTIDVGGGTGFC
TEGVANYIDTKHITLLDQSPHQMAKAKAKKTLQGVTFVEGDAENLPFNSAQFDRYTSAGS
IEYWPEPQRGIAEAYRVLKPGGVATLIGPVRATNWFSRFWCDLWMLFPMETEYRKWFTAA
GFKDLKISYIGPNAYKGVRQHGLIMGLTITGTKPADGESPVQLGEMLWMLGVIAGGYYFI
LPFLIILYAA
>XP_005704060.1 MPBQ/MSBQ methyltransferase [Galdieria sulphuraria]
GLIQHKKEAFWFYRFLSIVYDTIVNPFHWTVEMRDTSLKQAELVSRDLKVLDAGGGTGFT
TEGIVQYVDAHNITLLDQSPHQMAKAKKKPKLSSVNFVEGDAENLPFPSNSFDRYISAGS
IEYWPEPQRGISEAYRVLRCGGVATVIGPVRATHWFSRFWCDLWMLFPTEEEYRYWFQKA
GFEDIRVSYIGPAAYKGIREHGLIMGLTISGKKPVNGEPVCRLGPMQWLLGCIAGFYYFL
LPFFIMFYAA
>XP_005535393.1 probable MPBQ/MSBQ methyltransferase [Cyanidioschyzon merolae strain 10D]
GLIQHKREAFWFYRFLSLVYDTVVNPFHWTTEMRDASLRQAGLEEPDFKVLDVGGGTGFC
TEGIVQYVSPSQVTLLDQSPHQMQVAKRKPSLQGVTFVQGDAEALAFPTDSFDRVVSAGS
IEYWPEPQRGIAEAYRVLKPGGLATIIGPVRATNPVSRFFCDLWMLFPMEEEYRVWFTRA
GFTDLKVSYIGPPAYKGIRQHGLIMGLTITGRKPAPGEAKIQLGPMRWILGCIAGFYYFV
LPFAIMLYAA
>KAA8494213.1 2-methyl-6-phytyl-1,4-hydroquinone methyltransferase, chloroplastic [Porphyridium purpureum]
GMMQHKREAFWFYRWLSFFYDVVVNPFHWTREMRDEALLRAQLDSPCLRVVDVGGGTGF
S
TLGIVRYVAARYVVLMDQSAAQMSYAAQKRELSGVTFIEGDAENIPIRTNYADRYVSCGS
IEYWPNPQQGIGEAYRILKPGGLACMVGPVRATNPLSRFFCDTWMLFPTEQEYIYWFAAA
GFRNIKVSEITPAAYKGVRRHGLIMGLVVTGEKPLNGDLPLTMEDRVQMLGTVAGFYYFV
LPIAIMLYAA
>XP_005838443.1 hypothetical protein GUITHDRAFT_65997 [Guillardia theta CCMP2712]
RLIQHKAEAFWFYRYLSIVYDKIVNPGHWTEDMREDALKPAQLTSPDLDVVDVGGGTGFC
TLGIVKAVRPEKIVLMDQSPHQLEKARAKKGLEGVTIMEGDAEDLPFETDSKDRYVSAGS
IEYWPDPQRGICEAYRVVKPGGIACCIGPVHPTFPLSRIFADLWMLFPTEEEYIEWFTKA

GFEDVKITRIGPWWYHGVRRHGLIMGCSVTGRKPEAGLPKLQLGPKELILGTLGGFYYFL
VPVYMFLKHL

Dataset of divergent type MMT
>XP_002952366.1 hypothetical protein VOLCADRAFT_105494 [Volvox carteri f. nagariensis]
GLGLTLFALKRVLDTPSRKYDNNVGKEYDAWTEEGVLEYYWGEHIHLGYYSDEEGYFKQA
KFDFVDEMLKFSGAQDPKKILDVGCGFGGTSRHLAKKFKEASVTGITLSPKQVARGTELA
QQQGVNNVQFQVMDALAMEFPDDTFDLVWACESGEHMPDKKKYVEEMTRVLKPGGTLVI
A
CWCQREETAQDKEDLQFLYDEWAHPYFISIQEFERLMKGTGKLQNVHTDNWNKNTLASWR
HSIWVGVFDPWIVVSKPRIWYKTVREIVTIERMHQAFAKGLMEYGMMAGSKA
>KXZ49093.1 hypothetical protein GPECTOR_23g25 [Gonium pectorale]
GLGLTAYTLKRILDTPSRKYDNNVGKEYDAWTEEGVLEYYWGEHIHLGYYSDEEGYFKQA
KFDFVDEMLKFSGAQNPKKILDVGCGFGGTSRHLAKKFKDASVTGITLSPKQVQRGTELA
KEQGVGNVQFQVMDALAMEFPDDTFDLVWACESGEHMPDKKKYVEEMTRVLKPGGTLVI
A
CWCQREETAQDKEDLQFLYDEWAHPYFISIEEFGRLMNGTGKLEKVHTANWNKNTLASWR
HSIWVGVFDPWIVVFKPRIWYKTVREIVTIERMHQAFAKELMQYGMMAGTKA
>XP_001692723.1 predicted protein [Chlamydomonas reinhardtii]
GLGLSLFALKRILDTPSRKYDNNVGQEYDAWTEEGVLEYYWGEHIHLGYYSDEEGYFKQA
KFDFVDEMLRFSGAKNPATILDVGCGFGGTSRHLAKKFRDANVTGITLSPKQVQRGTELA
KEQGVGNVKFQVMDALAMEFPDNSFDLVWACESGEHMPDKRKYIEEMTRVLKPGGTLVIA
CWCQREEGPQDKEDLQFLYDEWAHPYFISIAEFGRLMNGTGKLDGVKLEDWNKNTISSWR
HSIWVGVFDPWVVVFKPRIWYKTVREIVTLERMHQAFEKGLMEYGMMTATKK
>PNH02628.1 putative tocopherol O-methyltransferase, chloroplastic [Tetrabaena socialis]
GLGLTAFTIKRILDTPSRTYDNNVGQEYDAWTEEGVLEYYWGEHIHLGYYSDEEGYFKQA
KFDFVDEMLGFSGAKAPQKILDVGCGFGGTSRHLAKKFRDASVTGITLSPKQVARGTELA
QQQGVTNVKFQVMDALAMDIPDDTYDLVWACESGEHMPDKKKYVEEMTRVLKPGGTLVI
A
CWCQREETPADKADLQFLYDEWAHPYFISIEEFGRLMNGTGKLQAVTTANWNQNTLASWR
HSIWVGVFDPWIVISKPRIWYKTVREIVTIERMHQAFATGLMEYGMMTGTKS
>KAA6422171.1 MPBQ MSBQ transferase cyanobacterial type (ISS) [Trebouxia sp. A1-2]
GVALTFFALKRVFDTPSRAYKENVGDEYDSWTEDGVLEYYWGEHIHLGYYTEQEGYFKQA
KLDFVDQMLKWSGAKSPSRVLDVGCGIGGTTRILAKNFPDANTQGITLSKSQVKRGTELA
AEQGLSNCSFQVMDALHMDFPDDTFDLVWACESGEHMPDKKLYIEEMTRVLKPGGTLVIA
CWCQREESSEEKDQLQFLYDEWAHPYFISNLEFGRLMEGTGKLQAVEVEDWTQPTIDSWR
HSIWVGVYDPWIVVFKPVVWYRTMREIVTLERMHRAFDKGLMQYGMMKAVKS
>PRW61310.1 S-adenosyl-L-methionine-dependent methyltransferase [Chlorella sorokiniana]
GAVLAGYGIKKVFDTPSRSYDQNVGQEYDAWTEEGVLEYYWGEHIHLGYYTEEEGYFIQA

KYDFIDEMFKWSGSDTPQKVLDVGCGIGGTSRFLAAKFPQASVTGITLSPNQVKRGTELA
AERGLGNVKFQVMDALKMEFPDNSFDLVWACESGEHMPDKKAYVDEMVRVLKPGGTLVI
A
TWCQREETESDRERLQFLYEEWAHPYFVSKEEYGRIMEGTGQLDNVATADWTAPTINSWR
HSIWVGVWDPWIVVFKPRIWYKTVREIVTLERMHRAFDDGLMEYGMMKAKKK
>XP_005650891.1 S-adenosyl-L-methionine-dependent methyltransferase [Coccomyxa subellipsoidea C-169]
GVAVVVLAVKKLFDTPSRTYDPNVGDEYDSWTEEGILEHYWGEHIHLGYYTEEEGYFKQA
KFDFVDEMLRWSGAEQPKRILDVGCGIGGTSRHLAAKFPGAQVQGITLSSKQVARGTELA
KERGLTNVNFQVMNALAMEFEDDTFDLVWACESGEHMPDKKAYVEEMARVLKPGGHMVI
A
TWCQREETDKERADLQFLYDEWAHPYFVSVQEYGRLLEGTGKMESVDIDDWTPQTLPTWR
HSNWVGVWDPWPVIFKPFVWYKVLREIVTLERMHRAFDSGLMEYGMAKAVKK
>Polytomella parva
GLAASAVAIKNVFDNPSRKYHGNVGQEYDAWTSEGILEHYWGDNIHLGYYSEKEGYFKKA
KVDFIDEMFRFSGSQSPRAVLDVGCGFGGSSRHLARKFPDSTIIGITLSAEQVKRARELA
AEQGLTNVHFFVMDALYMSLPAETFDLVWACESGEHMPDKRRFVSQMSRVLRPAGNLVIA
CWCQRDSSAQEQKELDFLYHEWAHPFFVSKEAFGRMLSEMGKFKNIQIQNWTRFTLPTWH
HSILVGVWDPWIVLSKPRIWYKTLREVVMIQRMHKAFAKGLMEYGMISAVKA
>Polytomella magna
GLAVGATAVIKVFNNPSRKYNNNVGQEYDAWTSEGVLEHYWGDNIHLGYYSEEDGFFKYA
KVEFIDRMLEFSKSESPKEILDVGCGFGGSSRHLAKKFPAASVQGITLSPKQVERGIELA
AEQGLTNVTLQVMDALKMDFPDNSFDLVWACESGEHMPDKRQFVEEMLRVLRPGGRLVIA
CWCQRDAGKQEQRELNFLYDEWAHPFFVSKEAFGRIMQDTGSLGD--------------

>Nitzschia sp.
AAIAGIGFTKAVLDKPSRPYKGSVGEEYDAWTSQGIVEYYWGEHIHLGYYPMDVGQISDA
QVLLMEQLCQFGGVQGNPKILDVGCGFGGTTRFLARQFSGADVTGISLSKEQVQRASEIS
SEQGVTNTKFVVEDAMELSFDDNSIDLVWACESGEHMPDKKRYVEQMVRVLKPGGRLIIA
TWCQRDDSTRDRRDLDYLYGEWSHPYFVSKELYAGLLKGTTVMQNIKVDNWAQETLPTW
R
KTIQLAFLNPWGWLFKPTTYMRCLRDAYCMERMHRAFHRGLMEYGVLCATKQ
>Spumella sp.
VYLLYLMKLSEISTKRSRKFEQTVAREYDQWTREGLVEYYWGEHIHLGYYERPPTDFVEA
KVRFVDRLLDVSGLRQPLRVLDVGCGIGGTTRHLARCLSGVSVTGIAISGAQIERAEALS
REQQVAGTAFARMDAQEMTFPDESFNVVWVCESSEHMPDKAKLVSEMMRVLKPGGRLVL

A
AWCQRDDRAEDSAQLQTLYDDWAHPHFVSIERFTELLASNGA-ADIVAQDWTAPTLPSWL
HSITVGILDPRPFLRQPGLIFGALREARTLALMHRAFRSGLMRYGVLTATKP
>CAMPEP_0174960874 /NCGR_PEP_ID=MMETSP0004_2-20121128|3933_1 /TAXON_ID=420556 /ORGANISM=Ochromonas sp., Strain CCMP1393 /LENGTH=400 /DNA_ID=CAMNT_0016209269 /DNA_START=89 /DNA_END=1291 /DNA_ORIENTATION=+
LFTALKSWADKALWTPSRTYSNSVGKEYDAWEEEGILEYYWGEHIHLGYYGEKESQFIQA
KYDFIDEMLKFGQFDVPLQILDVGCGIGGTSRYLAKKFQDTTVVGITLSQNQVNRATALA
KEQDVPNARFEVMDALDMTFPDNSFDYVWACESGEHMPDKKKYVEQMTRVLKPGGRIVI
A
TWCQRDEGDEEKRMLNFLYSEWTHPFFISISDYKKLMDGTGQLEDLATDDWTPQTIASWL
HSIWVGVFDPWPVFSKPKLWWKTFRDGLTLVRMHKSFNSKLMEYGMMTGTKK
>ENA|HAFO01015914|HAFO01015914.1 TSA: Uroglena sp. WA34KE, contig comp194936_c1_seq2, transcribed RNA sequence.
AILAIGKWVDEKLWTPSRTYNNTVGKEYDAWEEEGILEYYWGEHIHLGYYAEKDSLFIEA
KYDFIDEMLNFGRINAPAQILDVGCGIGGTSRYLAKKFTDTKVIGITLSNNQMKRATELA
KEQGVTNAEFLVMDALDMQFPDNSFDYVWACESGEHMPDKKRYIEEMTRVLKPGGKIVVA
TWCQRDEGNDEKRMLNFLYSEWTHPFFISISDYKKLMESTEVLNDIITDDWTPQTIASWL
HSIWAGVFDPWPVFSRPHLWWKTFRDGLTLVRMHKSFDSKLMEYGMMAAVKS
>ENA|HAGC01013791|HAGC01013791.1 TSA: Dinobryon sp. LO226KS, strain LO226KS, contig comp25212_c0_seq1, transcribed RNA sequence.
AFSAFARWFNRMLWTPSRVYNNTVGKEYDAWQEEGILEAYWGEHIHLGYYEPEYKAFIQA
KYDFIDAMLQFSGFDVPVKILDVGCGIGGTSRYLAKKFRQVEVTGITLSGNQKRRATELA
TEQQCDNAVFQVMDALQMEFEDESFDLVWACESGEHMPDKKRYVEEMTRVLKPGGQLVIA
TWCQRDEGESENRMLNFLYSEWTHPFFISISDYAQLMLGTGSLTSIRTDDWTPQTIASWL
HSIWVGVFNPWPVFRQPRLWWKTFRDGLTLYRMHNSFASGLMQYGMMTATKT
>ENA|HAGF01018081|HAGF01018081.1 TSA: Epipyxis sp. PR26KG, strain PR26KG, contig comp25997_c0_seq1, transcribed RNA sequence.

----------------------CGIGGTSRYLAKKFENAQVTGITLSPFQVKRATQLA
KEQFVSNAEFKVMDALNMNFPDNSFDYVWACESGEHMPDKKKYVEEMTRVLKPGGRIVIA
TWCQRDEGAQDRRMLDFLYSEWSHPYFISINDYKSLMLGTQQLGEVGTDDWTVPTIPSWR
HTLWAGVFDPRP--
>GGOE01001753.1 TSA: Euglena longa strain CCAP 1204-17a Contig1765, transcribed RNA sequence MPBQ/MSBQ methyltransferase
MLGVLFLALKKLFDTPSRKYNVNVGREYDAWTEEGVLEYYWGEHIHLGYYSPELGNFIEA

KVEFVNKMFDWSGAKGPRTILDVGCGIGGTSRQLAERLPAAQVTGITLSPKQVERGMELV
KERGIKNCELKVMDALKMDFPDNSFDLVWGCESGEHMPDKKKYVEEMVRVLKPGGTLVIA
TWCQREETQKEKGKLQFLYDEWAHPYFISKEAYVRIMHGTGQLECVGCEDWNEYTIDSWR
HSIWVGVRDPMGLFCKPAVLYKCLRDAITLEKMHRAFACGLMEYGMLKAKKK
>GEFR01007101.1 TSA: Euglena gracilis EG_transcript_7105 transcribed RNA sequence
VLSVVALGVKKVLDTPSRKYNVNVGKEYDAWTEEGVLEYYWGEHIHLGYYSPELGNFIEA
KINFVNQMFDWSGAKEPRSILDVGCGIGGTSRQLAERFPAAQVTGITLSPKQVERGTELV
KERGIKNCDLRVMDALKMDFPDNSFDLVWGCESGEHMPDKKKYVEEMVRVLKPGGTLVIA
TWCQREETQKEKDKLQFLYDEWAHPYFISKEEYVRIMNGTGQLQSVVTEDWNEYTIDSWR
HSIWVGVRDPMGLFCKPAVLYKCLRDAITLERMHRAFASGLMEYGMLKAQKK
>VEU41615.1 unnamed protein product [Pseudo-nitzschia multistriata]
TALAGTGFVKLFLDKPSRTYGGTVANEYDEWTEEGILEYYWGEHIHLGYYGPDVKYLNEA
QYTFIDEMMKFGGIDPAAKVLDVGCGFGGTSRYLARALPEAEVTAITLSPKQVERAQELA
IEQDTHNVNFMVEDALEMSFPDNSFDIVWACESGEHMPDKKKYIEQMMRVLKPGGKFVMA
TWCQRDDRKRDKRDLQYLYEEWTHPYFISKEEYTQLIDNTGVMNKVTAANWVRETIASWR
HTIFKGAKDPRGFIFKPKKYIKTLRDAYCIERMHRAFKRGLMEYGMLTATKK
>OEU07966.1 MPBQ/MSBQ methyltransferase [Fragilariopsis cylindrus CCMP1102]
AAMASTGFVKLFLDKPSRTYGGTVAQEYDEWTEEGILEYYWGEHIHLGYYGPDVKYLKDA
QYAFIDEMMKFGGIDPKAKVLDVGCGFGGTSRYLARALPESSVTAITLSPKQVERAKELA
IEQDTPNVKFMVEDALEMSFPDNSFDIVWACESGEHMPDKKKYIDQMMRVLKPGGKFVMA
TWCQRDDRKRDKRDLQYLYEEWTHPYFISKEAYAELIAETGVMNKATTADWVKETIATWR
HTILKGIKDPWGFIFKPKKYIKTLRDAYCIERMHRAFKRGLMEYGMLTATKK
>XP_002293723.1 sterol-c-methyltransferase [Thalassiosira pseudonana CCMP1335]
GAILTTATIK-YLDKPSRTYSGSVAREYDAWTQDGILEYYWGEHIHLGYYNEEEGYFIQA
KYDFIDEMMKFGGIDAGAKVLDVGCGFGGTSRYLADKLPKAEVTGITLSPNQVKRGTELA
MERNLPNAKFTVMNALEMDFPDNTFDIVWACESGEHMPDKEAYINEMMRVLKPGGKFVM
A
TWCQRDDRKRDKRDLRFLYEEWTHPYFISIEAYKELIDATTLMSNVKTADWVTPTIASWR
HSIWVGVYDPMGWIFKPTKYVKCARDAYCLERMHRAFKRGLMEYGMFAAVKK
>GAX14460.1 hypothetical protein FisN_11Hh079 [Fistulifera solaris]
AALAGTAGVKLILDKPSRTYAGSVGDEYDAWTSEGILEYYWGEHIHLGYYTPEEGYFIQA
KYDFIDKMMEFGDIDPKAKVLDVGCGFGGTSRYLAKALPSSHVTGITLSPNQVKRGTELA
VEQGVANTKFMVMDALKMDFPDNSFDIVWACESGEHMPDKKTYIEQMMRVLKPGGKFVM
A
CWSQREEGKRDKRDLQYLYEEWSHPYFVSINYFKKLIDDTGVMGEVKTANWVDETIASWR
HSVWVGIFDPRGWIFKPKQYYKCVRDAYCLERMHRAFKRGLMEYGMWAATKK

>XP_002186194.1 predicted protein [Phaeodactylum tricornutum CCAP 1055/1]
AAVAGTATVKLVLDKPSRTYGDTVAKEYDAWADDGILEYYWGEHIHLGYYSPEEGYFVQA
KYDFIDEMMTFGGIDAKAKVLDVGCGFGGTSRYLAKKLSDAHVTGITLSPKQVQRGTELA
VEQGVANTRFTVMDALQMDFPDNSFDIVWACESGEHMPDKKAYISEMMRVLKPGGTFVM
A
CWSQRDDSQRDKRDLQYLYEEWTHPYFISIKDFRKLIDDTDVMNPVTTANWVDETIASWR
HSIWVGVFDPRGWIFKPKTYVKCFRDAYCLERMHRAFKRGLMEYGMWTATKK
>EJK64762.1 hypothetical protein THAOC_14472 [Thalassiosira oceanica]
AAVAGTAGVKFILDRPSRAYNDSVAKEYDEWTQDGILEYYWGEHIHLGYYSEEEGYFIQA
KYDFIDEMMKFGGIDAGAKVLDVGCGFGGTSRYLADKLPKAEVTGITLSPNQVKRGTELA
EERGLPNAKFQVMNALEMDFPDNSFDIVWACESGEHMPDKEAYINEMMRVLKPGGKFVM
A
TWCQRDDRKKDKRDLRFLYEEWTHPYFISIEAYEELIDATKLMGGVKTADWCEPTIASWR
HSIWVGVYDPRGFIFKPTKYLKCARDAYCLERMHRAFKRGLMEYGMFAATKK
>CBJ31646.1 MPBQ/MSBQ transferase [Ectocarpus siliculosus]
AVGLLGAVAIKVLDTPSRPYDNTVGNEYDAWTEDGILESYWGEHIHLGYYNEEEGAFIQA
KYDFIDEMAKWGGVVAPKKVLDVGCGVGGTSRYLAKKLPETSVTGITLSPKQVERATQLA
EEQGVPNAKFQVTNALDMTFEDESFDLVWACESGEHMPDKGKYIEEMTRVLKPGGQLVVA
TWCQRDNSPEEERKLDFLYSEWTHPHFISINDYAKLMEGTGQLEQVETDDWAEQTTPTWR
LSIWVGVVNPWPWLRVPRSYYKTVRDAWCIERMHQAFKKGLMQYGMIKSVKK
>CEM04269.1 unnamed protein product [Vitrella brassicaformis CCMP3155]
AALAAGVAVKKVLDTPSRPYDGSVAREYDAWTEEGILEHYWGEHVHLGYYDDEDGSFKQA
KDDFVLKMLEFAKVDVPKDTLDLGCGIGGTARILAKAFPQSRILGIAISPNQIARANELA
TEQGVSSCKFMVMDGMKMTVPDNTFDFVWVCESTEHMPDKQKAIQEMTRVLKPGGRLVV
A
VWSQRDDTKRERRRLDYLYSEWSHPFFTSVPQFVEMLQDTGVMENIETADWAQQTLPSWR
HQIWLGIKDPLPWLTKPKFYWKCVRDAWCLNRMHQAFKEGLMQYGMITATKK
>KOO24628.1 hypothetical protein Ctob_000658 [Chrysochromulina tobinii]
VVALAFAWLARVLNTPSRVYDNSVGREYDAWTSEGILEYYWGEHIHLGYYGEKEPFFIEA
KYDFIDKMLEFSQAQAPLKVLDVGCGIGGTSRYIAKKFPKATVTGITISPEQQRRATALA
AERGVSNAKFEVCDALDMTYEDNTFDLVWACESGEHMPDKVKYVEEMARVLKPGGRIVIA
TWCQREEGPKERDTLDYLYGEWTHPYFISIEEYGRIMQRTGKLQKIVTADWATETIPAWR
HSVWVGVWDPWPVIRRPKLWWKVIRDAWCLEIMHRAFTNGLMQYGMMTATKP
>XP_022840839.1 Methyltransferase type 11 [Ostreococcus tauri]
GIVALAYGTKTIFDTPSRTYVNTVGTEYDAWTEEGILEYYWGEHIHLGWYSDEDGAFIQA
KFDFVDQMADWSEARAPARVLDVGCGIGGTSRHLARRFVGTEVTGITLSPNQVKRATELA

SEQGVTNANFQVMNALEMTFEDDTFDLVWACESGEHMPDKKKYVEEMVRVLKPGGKIVIA
TWCQRETPQKEKDNLQFLYEEWAHPYFISYEEYERLLRGTGSMESAASEDWVKNTLVSWR
HSIWVGVWDPWPVVFAPRQWYKVVREIVTLERMARAFESGLMTYGMIKGTKK
>EWM22420.1 Methyltransferase type 11 [Nannochloropsis gaditana]
VAGLLAARLPGVIARGWRSYNNSVGREYDAWTREKILEHYWGEHIHLGYYTEEDGYFIGA
KYDFIDRMMAFAKLDGPAKVLDVGCGIGGTTRYIAKKLTTSQVSGITLSQEQVRRAKELA
EEQDVTNAEFQVMDALHMSYPDNSFDLVWACESGEHMPDKKAYVEEMVRVLKPGGTLVIA
CWCQRDAKRSEQKRVDFMCTEWSHPYFISIQDFERLALGTGVMENVRTEDWAKFTLPSWR
HSVWVGVFDPFFWMLRPHLWVKILRDAFTLNVFHNAFKDGLMGYGMIYAQKK
>XP_009039696.1 hypothetical protein AURANDRAFT_12910, partial [Aureococcus anophagefferens]
-----------------RPYDNSVGREYDAWTKEGILEHYWGEHIHLGYYTDAEGYFIEA
KYNFTQRMMDWGGVTTDVKILDVGCGIGGTSRYMATTLPESSVTGITLSGEQRDRATKLA
AERDIPNAKFQVMDALNMDFEDNSFDVVWGCESGEHMPDKKKYVTEMARVLKPGGKMVI
A
TWCQRDNAAEEEQALDFLYSEWTHPYFISIPDYAKSLKKDAAMVDVETDDWNKNTIASWR
HSIWVGVFDPWPVIRAPRMWYKCLRDGICLERMHRAFKRGLMEYGMIKGTK-
>XP_003055537.1 predicted protein [Micromonas pusilla CCMP1545]
SLAFAFCKLKKNLDTPGRPW-TTVGKEYDAWTDEKILEYYWGEHIHLGYYKDEDGAFVEA
KLDFVDEMLAWSRFNGPRKVLDVGCGIGGATRHLANKFSNTRVTGVTLSPKQARRAGELA
LKQGVPNAEFLVMDALEMDFPDDHFDVVWACESGEHMPDKGKYVEEMVRVLKPGGTLVI
A
TWCQRSTPPREVVNLNYLYEEWAHPYFISINDYAMLLKGTMKMDTVETDDWTRQTIASWR
HSIWVGVWDPMPVFSRPRIWYKTLRDIVCLERMRRAFGCGLMQYGMIKGTKS
>XP_005841094.1 hypothetical protein GUITHDRAFT_91786 [Guillardia theta CCMP2712]
-------VAAFLLNKPSRAYQNTVGKEYDAWTEEGILEYYWGEHIHLGFYNKSDNPFKET
KYKFIDEMYKWSGAEAPLKVLDVGCGIGGTSRYLAKKLGDTKVTGITLSPNQVQRATELA
KEKGLDNVEFKVMDALKMEFPDNTFDLVWGCESGEHMPDKYKYVEEMTRVLKPGGTLVI
A
TWCQRETPESEKDKLKFLYEEWAHPYFISIEEYERHCKKTGKLEAISGANWVKETIHAWR
HSIWVGIWDPWIVIWKPWIWWKVIREIVTLERMHRAFDSGLMTYGMIRAKKQ
>XP_005787794.1 hypothetical protein EMIHUDRAFT_98396 [Emiliania huxleyi CCMP1516]
LVATIFAWVLRVFNTPSRSYRNSVGEEYDAWTSEGILEYYWGEHIHLGYYEEGQPLF---
----------------PSKVLDVGCGIGGTSRYIAKKFPSADVTGITISPEQRRRAEQLA
TERGIGNAKFELVDALNMTFADNSFDLVWACESGEHMPDKERYVQEMSRVLKPGGRIVIA
T-------AKERATLDYLYGEWSHPYFISIEEYGRIMERTGELDQVVTDDWAKQTIPAWR

HSIWAGVWDPWPVFRRPRLWYKVLRDSWCLEVMHRAFTNGLMRYGMMTATKK
>KAA8496924.1 2-methyl-6-phytyl-1,4-hydroquinone methyltransferase [Porphyridium purpureum]
VIVVLALIIKKDLDTPKRPYTDGVGAEYDKWTMDGILEYYWGEHIHLGYYSKEEGAFKQA
KIDFTKQMLYWTGVKAPRRILDVGCGIGGTSRILAKEFPHAEVIGISLSSEQVQRATALA
EKEGLDNVTFRLMDALHMDFDDDTFDLVWGCESGEHMPDKAAYVNEMTRVLAPQGTLAI
A
TWCQRDTPQTEKDRLQYLYDEWSHPHFISYQEYMRIMQRTNVMGDVIGEDWNDVTLPSW
R
HSNWVGVVDPWPVIFKPALWYWVIREIVCLERMHRAFRDGLMQYGMIKGVKK
>XP_002777996.1 3-demethylubiquinone-9 3-methyltransferase, putative [Perkinsus marinus ATCC 50983]
---------TGTQTQPERYRPGGVGNEYDKWTNDGLVETYWGEHIHLGAYTTDEWFFKEQ
KFTFVDDLLSWGGFDATLRILDVGCGIGGSSRIMAKRY--EAVTGITLSDAQVERASELS
REAGLNNVTFKKMDALRMEFPDASYDLIWSCECGEHVPDKAKYIEEMCRVLKPGGRLIVA
TWCEKDDRDHQKWLLRFLYEEWSHPMFISIEKYEDILRKNSLMEGVESADWTPQTLPSWR
HSIFVGVWWPWPVIIRPRVWWSTIREIVTIERMHEAFRDGVMRYGVFRATK-
>chlamydomonad sp. NrCl902
GLCLAAAGYMLFQKGSRAYKGSTVGAEYDAWTEEGILEHYWGEHIHLGYYSEAEGYFKAA
KYDFVDAMLEFSGVKS-ASILDVGCGFGGTSRHLAKKFPDAKVTGITLSPKQVERGTQLA
HEQGVGNAEFKVMDALAMTLPSDSFDLVWACESGEHMPDKRRYVEEMVRVLKPGGTLVIA
CWCQREETPQETRELRFLYDEWAHPHFISIQEFTRIMEGTGQLAGVTGEDWTAHTLPSWR
HSLWVGVDDPWPVIAKPRIWYKTLREAVTLERMHRAFASGLMEYGLMRATKL
